# Automated glycan assembly of Lewis type I and II oligosaccharide antigens†
†Electronic supplementary information (ESI) available: Full experimental information, HPLC chromatograms for AGA syntheses, ^1^H, ^13^C, COSY and HSQC NMR spectra of all new compounds. See DOI: 10.1039/c9sc00768g


**DOI:** 10.1039/c9sc00768g

**Published:** 2019-04-29

**Authors:** Mónica Guberman, Maria Bräutigam, Peter H. Seeberger

**Affiliations:** a Department of Biomolecular Systems , Max Planck Institute of Colloids and Interfaces , Am Mühlenberg 1 , 14476 Potsdam , Germany . Email: Peter.Seeberger@mpikg.mpg.de; b Department of Chemistry and Biochemistry , Freie Universität Berlin , Arnimalle 22 , 14195 Berlin , Germany

## Abstract

Human blood group related glycan antigens are fucosylated (neo-)lactoseries oligosaccharides that play crucial roles in pathogenic processes. Lewis type-II-chain antigens mark the surface of cancer cells, but are also mediators of bacterial infections. To investigate the biological roles of Lewis type glycans a host of synthetic approaches has been developed. Here, we illustrate how automated glycan assembly (AGA) using a set of six monosaccharide building blocks provides quick access to a series of more than ten defined Lewis type-I and type-II antigens, including Le^x^, Le^y^, Le^a^, Le^b^ and KH-1. Glycans with up to three α-fucose branches were assembled following a strictly linear approach and obtained in excellent stereoselectivity and purity.

## Abstract 2

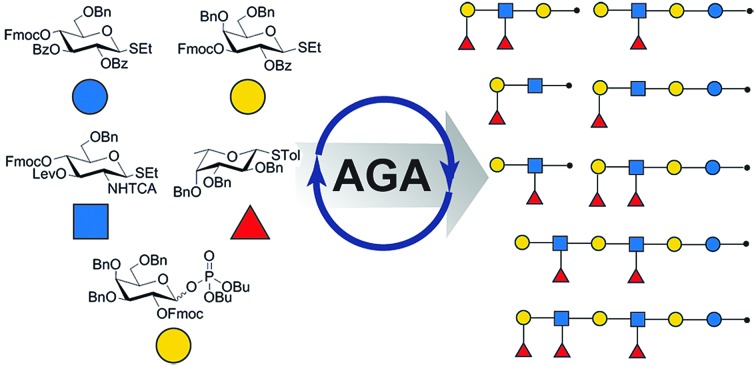
Lewis antigens are fucosylated oligosaccharides that play crucial roles in various biological processes. Here, we illustrate how automated glycan assembly (AGA) provides quick access to a series of more than ten defined Lewis type-I and type-II antigens.

## Introduction

**Fig. 1:**
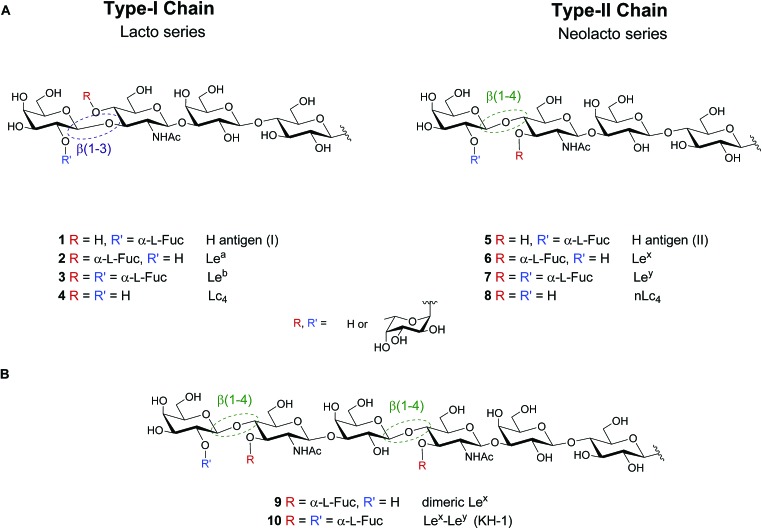
(A) Lewis type-I and type-II chain blood group related oligosaccharide antigens and (B) dimeric structures of the Lewis type-II chain that are exclusively found on cancer tissue.

Lewis antigens are lacto- or neolacto-series oligosaccharides that are commonly found as part of glycoproteins or glycolipids on the eukaryotic cell surface.^1^–^3^ These antigens are related to the ABO blood-group system and are implicated in developmental processes, reproductive physiology, oncogenic transformations, cell–cell communication and pathogen–host interactions.^2^,^4^–^11^ Lewis lacto-series (type-I-chain) glycans **1–3** are fucosylated versions of a lactotetraosyl (Lc_4_) core **4**. Similarly, Lewis neolacto (type-II-chain) antigens **5–7** result from fucosylation of neolactotetraosyl (nLc_4_) core **8** (Fig. 1A).

While the importance of blood group antigens for blood transfusions is established, their involvement in infectious diseases and cancer development is still emerging. Lewis^b^ (Le^b^) **3** expressed on gastric epithelium is the receptor for *Helicobacter pylori*, the cause chronic gastritis and peptic ulcers.^6^ Type-II chain analogue Lewis^y^ (Le^y^) **7** is a tumor-associated carbohydrate antigen (TACA), that is overexpressed on the cell surface of several types of cancer.^12^–^14^ Le^b^ and Le^y^ vary minimally in the regiochemistry of the glycosidic linkage in the terminal *N*-acetyllactosamine subunit of the Lc_4_ (**4**)/nLc_4_ (**8**) core (β(1–3) *vs.* β(1–4), respectively). Extended chain versions of Lewis antigens such as Le^x^ dimer (Le^x^–Le^x^, **9**) and KH-1 (Le^x^–Le^y^, **10**) are attractive targets for tumor immunotherapy (Fig. 1B). These TACAs are overexpressed in colorectal cancer and overcome the low immunogenicity observed in human trials when using shorter antigens such as Le^y^.^15^–^20^

Synthetic access to oligosaccharide antigens is essential as isolation of useful amounts of pure glycans from biological sources is difficult.^21^,^22^ Numerous syntheses of Lewis antigens that serve as tools *e.g.* for immunotherapy^20^,^23^,^24^ have been reported, including solution-phase, solid-phase and automated syntheses.^19^,^25^–^28^ Despite the structural similarity of Lewis antigens, typically total syntheses produce single structures.^29^–^31^

Here, we present a general method to assemble the entire class of Lewis antigens. The lacto- and neolacto-series target molecules differ in three structural aspects: the presence or absence of fucose on the terminal galactose; the presence or absence of fucose on GlcNAc, and the β(1–3) or β(1–4) linkage in the lactosamine subunit. The logic of automated glycan assembly (AGA) is based on the selection of a minimum set of monosaccharide building blocks to assemble all targeted glycans *via* a linear glycosylation and deprotection sequence.^32^,^33^ AGA reduces the synthesis time considerably^34^,^35^ and facilitates the assembly of large, complex antigens such as **9** or **10**.^36^ Access to lacto- and neolacto-series variants that carry a linker for conjugation allows them to be used as tools for diverse biological applications, in particular those where minor structural differences between type-I- and type-II-chain antigens,^37^ are concerned.

## Results and discussion

**Fig. 2:**
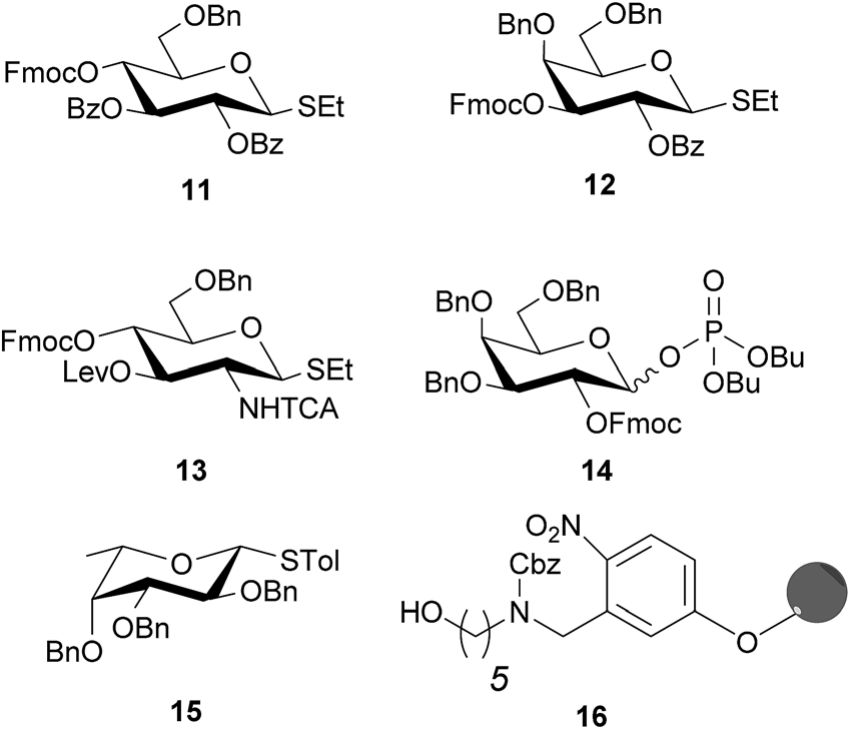
Solid support and building blocks used for the AGA of protected Lewis type-II chain oligosaccharides.

“Approved building blocks”, referring to monomers that can be prepared in large amounts, are shelf stable and upon activation result in reliable and selective glycosidic bond formation, are key to AGA.^35^ The five monosaccharide building blocks **11–15**, which suffice to access all Le type-II blood group related antigen targets (Fig. 2), are either commercially available or were synthesized in multi-gram scale in a few steps starting from commercial intermediates (see ESI† for details). Hydroxyl groups of building blocks **11–14** that engage in chain elongation are temporarily protected as 9-fluorenylmethoxycarbonyl (Fmoc) carbonates that are stable under the acidic glycosylation conditions, but can be easily removed under mild basic conditions. Levulinoyl ester (Lev) marks the C3-hydroxyl in glucosamine (GlcN) **13** as orthogonal temporary protecting group as illustrated for AGA before.^38^,^39^ Benzyl ether (Bn) and benzoyl esters (Bz), when C2 participation is needed, are used as permanent protecting groups. The amine of GlcN **13** was protected as an *N*-trichloroacetyl (TCA) group to ensure β-selectivity. The permanent protecting groups should be removed after AGA and light-induced cleavage of the solid support by methanolysis (Bz) and hydrogenolysis (TCA and Bn). Ready synthetic access and high stability, prompted us to use thioglycosides as anomeric leaving groups of all building blocks except galactose **14**. The C2-OFmoc in **14** resulted in low stereoselectivity for the thioglycoside, while the dibutyl phosphate building block ensured excellent stereoselectivity (see ESI†). The selectivity differences may be a result of a change in solvent used during the glycosylation, since dioxane is added to ensure solubility of NIS/TfOH required for thioglycoside activation. Dioxane coordinates the β-face of the oxocarbenium ion that forms during glycosylation and favors the formation of the α-glycosylation product.^40^ Dibutyl phosphates can be activated by TMSOTf in DCM, hence solvent coordination does not influence the reaction.

Before synthesizing the target molecules, the five building blocks **11–15** were tested for their performance in AGA by establishing optimal glycosylation conditions in the context of dimer or trimer syntheses (Table 1). All AGA syntheses were executed using an oligosaccharide synthesizer and Merrifield resin equipped with photolabile linker **16** as solid support.^26^,^39^,^41^,^42^ After each glycosylation, the temporary protecting group was removed to allow for further chain elongation. In the case of Fmoc, 10 min incubation with 20% piperidine in DMF was sufficient; Lev was selectively cleaved by treatment with a hydrazine acetate solution (0.15 M), liberating a hydroxyl group for the subsequent glycosylation step. After complete assembly, the desired oligosaccharide was cleaved from the solid support in a continuous flow photoreactor and analyzed by HPLC.^39^

**Table 1:** Optimized glycosylation conditions for AGA using building blocks **11–15**^*a*^

Entry	Glycosyl donor	*t*_1_ (min)	*T*_1_ (°C)	*t*_2_ (min)	*T*_2_ (°C)
1	**11**	5	–20	20	0
2	**12**	5	–20	20	0
3	**13**	5	–20	40	0
4	**14**	5	–35	30	–15
5	**15**	5	–40	20	–20

^*a*^ *t*_1_: incubation time, *T*_1_: incubation temperature, *t*_2_: glycosylation time, *T*_2_: glycosylation temperature.

Full conversion and excellent stereoselectivity were achieved for thioglycosides **11** and **12** employing eight equivalents of building block and a glycosylation time of 20 min at 0 °C, after a short incubation (incubation time, *t*_1_) at lower temperatures (Table 1). Glycosylation reagents are added dropwise to the reaction vessel at a controlled incubation temperature (*T*_1_). *T*_1_ is typically 20 °C below the glycosylation temperature (*T*_2_), to minimize reactivity before the reagent delivery process is completed. Afterwards the reaction vessel is warmed up to *T*_2_ to perform the coupling. Highly reactive perbenzylated fucose **15** was activated at –20 °C to avoid hydrolysis. Glucosamine **13** proved less reactive than the other building blocks and required 40 min glycosylation time to achieve full conversion. Dibutyl phosphate **14** was coupled at –15 °C for 30 min using five equivalents of building block.

**Fig. 3:**
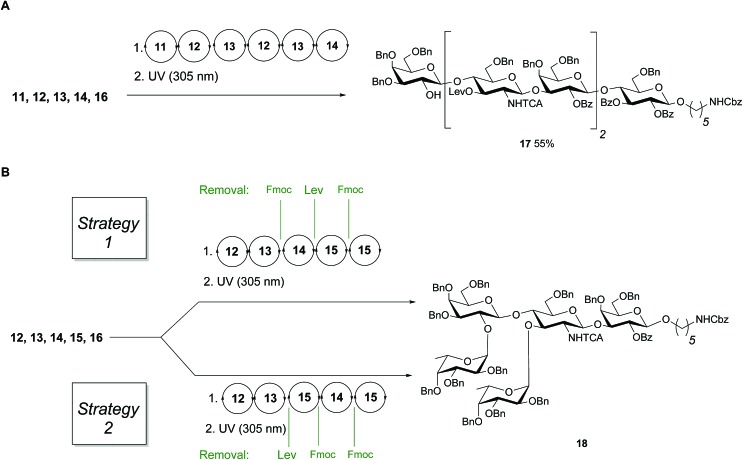
(A) AGA of fully protected linear hexamer **17** (B) different assembly strategies for the synthesis of **18**. Conditions for AGA: (1) 8 equiv. of **11**, **12**, **13** or **15** and NIS/TfOH in DCM/dioxane for –20 °C (5 min) → 0 °C (20 min or 40 min) or –40 °C (5 min) → –20 °C (20 min), or **14** (5 equiv.) and 5 equiv. of TMSOTf in DCM at –35 °C (5 min) → –15 °C (30 min). Fmoc removal in 20% piperidine in DMF at 25 °C for 10 min. Lev removal in 0.15 M hydrazine in py/AcOH/H_2_O for 2 × 30 min pulsed bubbling. (2) Photocleavage: *hν* (305 nm).

Linear hexasaccharide nLc_6_**17**, the unbranched backbone of KH-1 and Le^x^-dimer, served as a first test for the optimized conditions. HPLC analysis of the crude products after AGA and light-induced cleavage showed that just one product was formed (ESI†) before protected nLc_6_ was isolated in 55% yield by preparative HPLC (Fig. 3A).

Next, two branching strategies for Le type-II antigens Le^y^, Le^x^, Le^x^-dimer and KH-1 were evaluated. After introduction of building block **13** into an oligosaccharide, the Fmoc group can be removed and a galactose can be attached at the C4 hydroxyl group of GlcN before the Lev at the C3 hydroxyl is cleaved and a fucose is attached; subsequently chain elongation continues (‘strategy 1’). Alternatively, this process can be inversed (‘strategy 2’, Fig. 3B). Both branching strategies were tested using **18** as a model to ensure that all glycosylation cycles result in full conversion and excellent stereoselectivity. After light-induced cleavage and preparative HPLC, **18** was isolated in 39% yield using strategy 1. Strategy 2 resulted in an improved result as judged by analytical HPLC and 51% isolated yield.

**Fig. 4:**
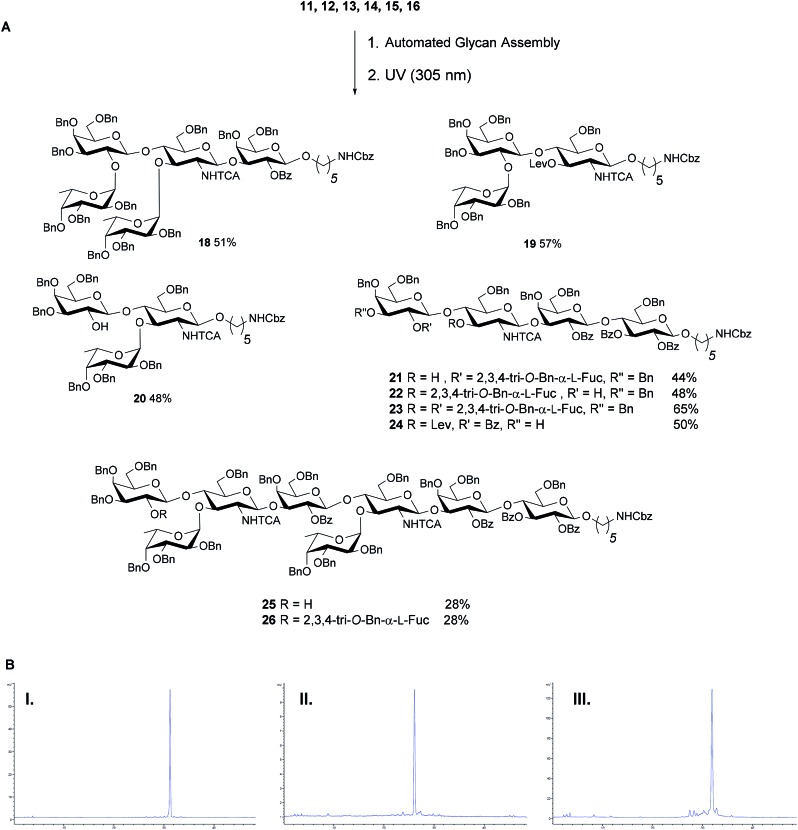
(A) AGA of fully protected Lewis type-II oligosaccharides. Reaction conditions for the AGA: (1) 8 equiv. of **11**, **12**, **13** or **15** and NIS/TfOH in DCM/dioxane for –20 °C (5 min) → 0 °C (20 min or 40 min) or –40 °C (5 min) → –20 °C (20 min), or **14** (5 equiv.) and 5 equiv. of TMSOTf in DCM at –35 °C (5 min) → –15 °C (30 min). Fmoc removal in 20% piperidine in DMF at 25 °C for 10 min. Lev removal in 0.15 M Hydrazine in py/AcOH/H_2_O for 2 × 30 min pulsed bubbling. (2) Photocleavage: *hν* (305 nm). The exact reaction sequences can be found in the ESI.† (B) Analytical NP-HPLC of protected oligosaccharides Le^x^**22** (I), Le^y^**23** (II) and KH-1 **26** (III) after AGA and photo-induced cleavage from the resin. HPLC was performed using YMC-Pack 5 μm (150 × 4.6 mm i.d.), detection by ELSD.

With a set of building blocks in hand, a series of protected Lewis type-II-chain blood group related antigens **19–26** (28–65% yield) was synthesized *via* strategy 2 with excellent stereoselectivity (Fig. 4). The syntheses of **19–24** yielded no significant amounts of deletion sequences. The AGA protocol was adjusted slightly for the extended chain antigens **25** and **26** to achieve full conversion in every glycosylation cycle. Two glycosylation cycles were performed when introducing the second GlcN building block since the GlcN building block is less reactive and the branched acceptor is sterically more hindered (Fig. 4). Antigens **17–26** were assembled using five building blocks. Le^x^ and H-antigen were assembled with the initial lactose unit (**21** and **22**) or without (**19** and **20**) to provide access to all variants of these structures for biological studies.^43^ Lewis type-chain ceramides on the surface of human cells carry the initial lactose unit,^44^ while many biological studies only consider the terminal fucosylated epitope.^45^,^46^ Streamlined coupling cycles rendered the assembly of KH-1 nonasaccharide **26** (15 h) significantly faster than an earlier AGA version that required 23 h.^25^

**Fig. 5:**
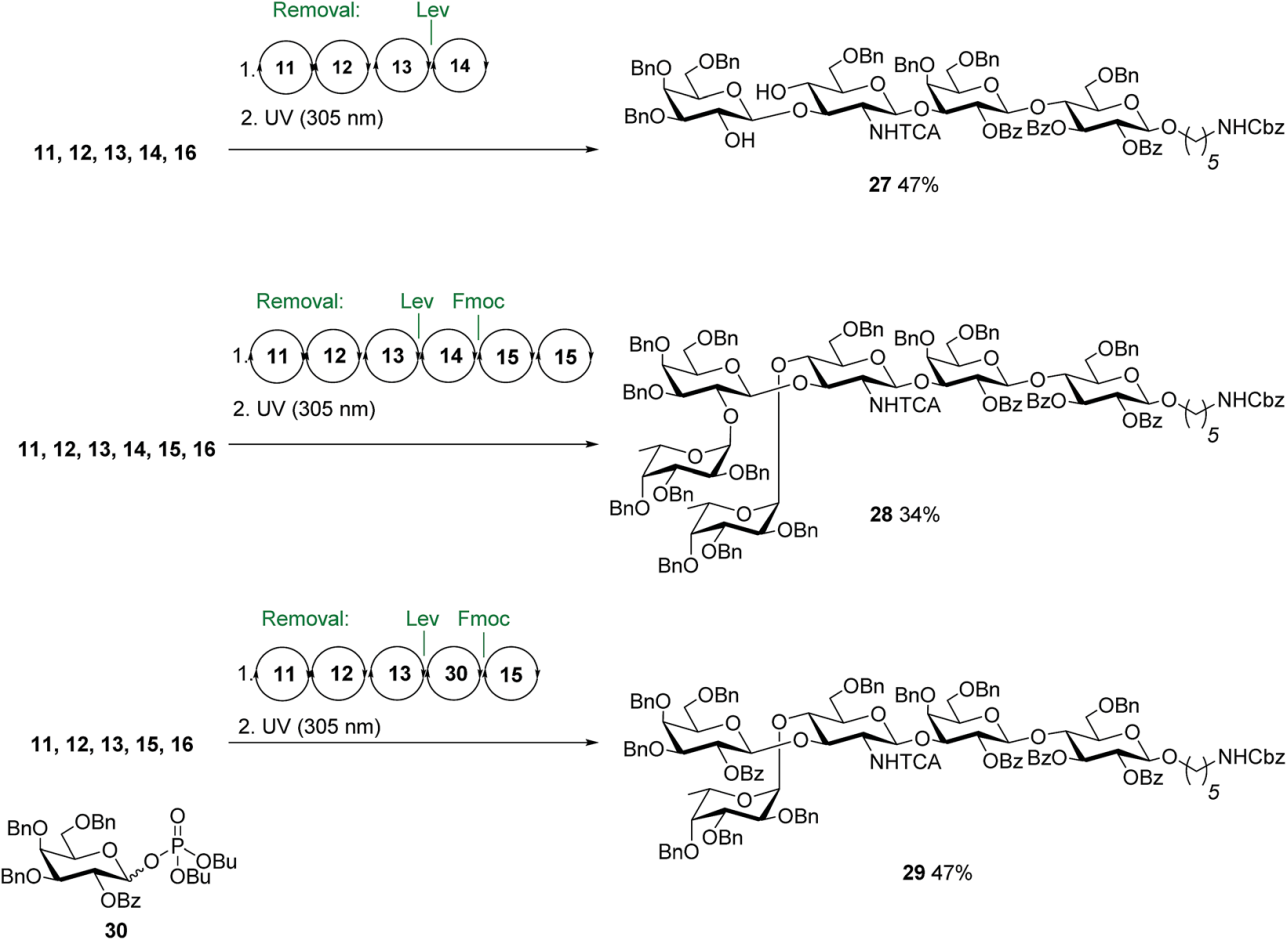
Syntheses of the protected Lewis type-I chain antigens **27**, **28** and **29**. Conditions for AGA: (1) 8 eq. of **11**, **12**, **13** or **15** and NIS/TfOH in DCM/dioxane for –20 °C (5 min) → 0 °C (20 min or 40 min) or –40 °C (5 min) → –20 °C (20 min), or **14** or **30** (5 equiv.) and 5 equiv. of TMSOTf in DCM at –35 °C (5 min) → –15 °C (30 min). Fmoc removal in 20% piperidine in DMF at 25 °C for 10 min. Lev removal in 0.15 M hydrazine in Py/AcOH/H_2_O for 2 × 30 min pulsed bubbling. (2) Photocleavage: *hν* (305 nm).

After preparing all Lewis type-II-chain antigens, we set out to assemble the Lewis type-I-chain antigens Lewis^a^ (Le^a^, **2**) and Le^b^**3**. Both contain a β(1–3) instead of a β(1–4) linkage between GlcN and the terminal Gal, with the C4 GlcN fucosylated (Fig. 1). Since type-I- and type-II-chain antigens differ only in the substituents attached at the non-reducing end of the GlcN unit, AGA of type-I-chain structures can rely on the sequential cycles developed for type-II chains, as simply the order of Fmoc and Lev cleavage in the GlcN unit has to be inverted. Protected Lc_4_ tetrasaccharide **27** was obtained in a yield comparable to that for type-II-chain analogue nLc_4_ (Fig. 5). However, the approach failed to afford branched structures since under these conditions, after C4 fucosylation of GlcN, Lev was not properly cleaved from the C3 hydroxyl. Therefore, assembly strategies where galactosylation of GlcN precedes Fmoc deprotection and fucosylation were used for the AGA of Le^b^**28** and Le^a^**29** (Fig. 5).

Replacement of **14** by **30** that only bears permanent protecting groups allowed for the assembly of Le^a^**29**. A difucosylation strategy, where the Fmoc groups at C2 of galactose **14** and C4 of the GlcN **13** were removed simultaneously furnished **28** (Fig. 5).

**Fig. 6:**
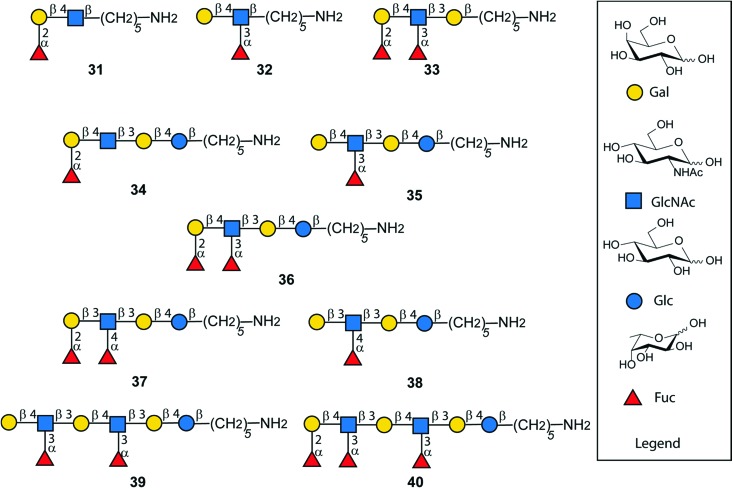
Synthetic Lewis type-I- and type-II-chain antigens **31–40**.

Following AGA and photo-induced cleavage from the solid support, all permanent protecting groups have to be removed, to furnish the desired antigens. Global deprotection relied on two steps. Benzoyl groups were cleaved with sodium methoxide in MeOH/DCM as reaction progress was monitored by MALDI. In the second step, benzyl ethers, TCA and the carboxybenzyl group at the amino linker were removed using hydrogenolysis catalyzed by Pd/C to afford the deprotected, *N*-acetylated oligosaccharides. Reversed phase HPLC yielded a series of fully deprotected Lewis antigens **31–38** that carry a C5-amino linker at their reducing end for immobilization on glycan array surfaces or conjugation to carrier proteins (Fig. 6).^47^,^48^ Rather low isolated yields over the two deprotection steps (17–54%) may be a result of the poor solubility of partially and fully deprotected glycans during and after deprotection.^49^ In addition, partial cleavage of fucose and TCA was observed. For the extended chain antigens **25** and **26** the combination of methanolysis and hydrogenolysis did not lead to the desired products Le^x^ dimer (**39**) and KH-1 (**40**). Severe solubility issues for the partially-deptrotected oligosaccharides formed during the deprotection process prevented complete hydrogenolysis. Birch reduction followed by peracetylation to facilitate purification was the endgame during the total synthesis of KH-1.^30^ This procedure was not applicable as it would result in the irreversible acetylation of the C5-amino linker. Finally, compounds **25** and **26** were deprotected using sodium in liquefied ammonia gas followed by methanolysis. The final oligosaccharides were purified using a Sephadex-G25 column to yield **39** (13%) and **40** (19%). The bottleneck of chemical glycan synthesis is the removal of protective groups from the final molecules and is common to AGA and other modes of glycan construction. Alternatives such as a combination of AGA with enzymatic glycosylations may circumvent deprotection issues but these methods have not yet been established for complex, branched oligosaccharides such as KH-1.^50^,^51^

## Conclusions

A set of Lewis type- I and type-II-chain antigens was synthesized *via* automated glycan assembly in a fast, reliable and reproducible fashion. Five building blocks were sufficient to synthesize all Lewis type-II antigens including H-antigen II, Le^x^, Le^y^ and KH-1 and the Lewis type-I-chain-related structures Lc_4_ and Le^b^. For the assembly of Le^a^ an additional building block was necessary, since the temporary Lev protecting group was difficult to cleave. Optimized glycosylation cycles led to full conversion and excellent stereoselectivity during AGA and minimized the formation of side products. Global deprotection of the target molecules provided sufficient quantities of the target glycans for biological studies.

## Conflicts of interest

There are no conflicts to declare.

## Supplementary Material

Supplementary information
