# Supplementary material for: Automated glycan assembly of Lewis type I and II oligosaccharide antigens
Source: Chem Sci. 2019 Apr 29;10(21):5634–40. doi: 10.1039/c9sc00768g (PMC6552968; doi:10.1039/c9sc00768g)

Electronic Supplementary Material (ESI) for Chemical Science.
This journal is © The Royal Society of Chemistry 2019

Automated Glycan Assembly of Lewis Type I and II Oligosaccharide Antigens

Mónica Guberman,[a, b, †] Maria Bräutigam[a, †] and Peter H. Seeberger[a, b, *]

[a] Department of Biomolecular Systems, Max Planck Institute of Colloids and Interfaces, Am Mühlenberg 1, 14476 Potsdam, Germany

[b] Department of Chemistry and Biochemistry, Freie Universität Berlin, Arnimalle 22, 14195 Berlin, Germany

Supporting Information

Table of Contents

1. Experimental Section S2

General Methods S2

Synthesis of Building Blocks S2

Synthesis of Protected Oligosaccharides S3

General Procedure for Automated Glycan Assembly S3

β-Stereoselectivity for the Nonreducing end Gal-GlcNAc Linkage S7

Experimental Data for Protected Oligosaccharides 17-29 .... S8

Deprotection of Oligosaccharides 31-40 .... S26

Global Deprotection: Methanolysis - Hydrogenolysis S26

Experimental Data for Deprotected Oligosaccharides 31-38 .... S27

Global Deprotection: Birch Reduction - Methanolysis S33

Experimental Data for Deprotected Oligosaccharides 39-40 .... S33

2. NMR spectra of all new compounds S36

1. Experimental Section

General Methods

All reagents and solvents were acquired from commercial sources, unless stated otherwise. Monosaccharide building blocks were purchased from GlycoUniverse. Anhydrous solvents were obtained from a Solvent Dispensing System (J.C. Meyer). TLC was performed on 0.25 mm Kieselgel 60 F_{254} glass-supported plates (Macherey-Nagel), with detection via UV light (254 nm) and sugar stain (3.70 mL of *p*-anisaldehyde in 140 mL of a solution 3.5% H_2SO_4 in ethanol). For flash chromatography purifications, a Reveleris X2 Flash Chromatography System (GRACE Discovery Sciences) and Reveleris Silica Gel columns were used. Amberlite IR-120 (Across Organics) protonic exchange resin was rinsed with THF, water, methanol and dichloromethane before use. Palladium on carbon was removed from reaction mixtures by filtration with Rotilabo syringe filters (Roth), PTFE filters (pore size: 0.45 µm). Sephadex G25 resin was used for size-exclusion chromatography. NMR spectra were obtained using Ascend 400 (Bruker) and Agilent 400 MHz NMR Magnet (Agilent Technologies) spectrometers at 400 MHz (^{1}H) and 100 MHz (^{13}C) or a Varian 600 (Agilent) at 600 MHz (^{1}H) and 150 MHz (^{13}C), or a Ascend 700 (Bruker) at 700 MHz (^{1}H) and 176 MHz (^{13}C). $CDCl_3$, CD_3OD or D_2O were used as solvents and chemical shifts (δ) referenced to internal standards ($CDCl_3$: 7.26 ppm ^{1}H, 77.16 ppm ^{13}C; CD_3OD: 4.87 or 3.31 ppm ^{1}H, 49.0 ppm ^{13}C; D_2O: 4.79 ppm ^{1}H) unless stated otherwise. Assignments were supported by COSY and HSQC experiments. IR spectra were measured with a Spectrum 100 FT-IR Spectrometer (Perkin Elmer). Only diagnostic signals are listed. Specific rotations were measured using a UniPol L 1000 polarimeter (Schmidt + Haensch). For monitoring reactions by mass spectrometry, an Agilent 1100 Series LC/MSD mass spectrometer was used. MALDI spectra were obtained with a Daltonics Autoflex Speed spectrometer (Bruker). ESI-HRMS were performed with a Xevo G2-XS Q-Tof (Waters). HPLCs were performed on Agilent 1200 Series systems.

Building Block Syntheses

Building blocks **12**, **13**, **15** were acquired from GlycoUniverse (product codes Gal32.11140202, GlcN30.15131402, and Fuc32.020202, respectively). Monosaccharides **11**[1] and **30**[2] were synthesized following previously described procedures.

---

[1] *Chemistry Letters* **2008,** *37*, 942-943.
[2] *Carbohydrate Research* **2003,** *338*, 1441-1454.

Dibutyl 2-*O*-benzoyl- 3,4,6-tri-*O*-benzyl-β-D-galactopyranoside (14)

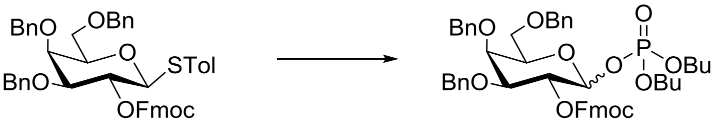

S0 **14**

Dibutyl hydrogen phosphate (1.77 mL, 8.93 mmol, 2.0 equiv.) was added to a round-bottom flask containing activated 4 Å molecular sieves sieve (5.00 g) anhydrous DCM (50.0 mL) and left stirring for 1.5 h. The molecular sieves were allowed to settle and the supernatant was added to a solution of commercially-available thioglycoside **S0** (3.47 g, 4.46 mmol) in anhydrous DCM (30.0 mL). The mixture was cooled down to 0 °C and NIS (1.30 g, 5.80 mmol, 1.3 equiv.) and triflic acid (118 µL, 1.34 mmol, 0.3 equiv.) were added. The reaction was stirred for 1h and then quenched with aq. sat $NaHCO_3$ (30.0 mL). The organic layer was washed with $Na_2S_2O_3$ (30.0 mL) and water (30.0 mL), dried with Na_2SO_4, filtered and the solvent was removed *in vacuo*. Purification by silica gel chromatography (hexanes/EtOAc 75:25) afforded **14** (3.32 g, 3.84 mmol, 86% yield, α/β mixture) as an oil.β only: ^{1}H NMR (400 MHz, $CDCl_3$) δ 7.84 – 7.74 (m, 2H), 7.65 (ddt, *J* = 7.7, 1.9, 1.0 Hz, 2H), 7.48 – 7.21 (m, 19H), 5.40 – 5.23 (m, 2H, **H-1** + H-2), 4.99 (d, *J* = 11.5 Hz, 1H), 4.73 (d, *J* = 12.2 Hz, 1H), 4.63 (dd, *J* = 11.8, 5.1 Hz, 2H), 4.53 – 4.40 (m, 3H), 4.32 (dd, *J* = 10.2, 7.7 Hz, 1H), 4.28 – 4.18 (m, 1H), 4.13 – 3.87 (m, 5H, 2xCH_2 + H-4), 3.76 (ddd, *J* = 7.6, 5.5, 1.1 Hz, 1H, H-5), 3.72 – 3.53 (m, 3H, H-3 + H-6), 1.66 – 1.55 (m, 2H, CH_2), 1.55 – 1.43 (m, 2H, CH_2), 1.42 – 1.31 (m, 2H, CH_2), 1.27 (dtd, J = 15.8, 7.2, 1.0 Hz, 2H, CH_2), 0.94 – 0.85 (m, 3H, CH_3), 0.81 (t, J = 7.4 Hz, 3H, CH_3). ^{13}C NMR (101 MHz, $CDCl_3$) δ 154.4 (C=O), 143.4, 143.2, 141.23, 141.19, 138.2, 137.6, 137.4, 128.5, 128.4, 128.3, 128.18, 128.15, 127.88, 127.86, 127.8, 127. 7, 127.5, 127.4, 127.22, 127.19, 125.2, 125.1, 120.00, 119.98, 96.70 (C1), 96.65 (C1), 79.8 (C3), 75.7 (C2), 75.6 (C2), 74.7 (BnOCH_2), 74.2 (C5), 73.5 (OCH_2CH), 72.5 (C4), 72.4 (BnOCH_2), 70.3 (BnOCH_2), 67.9 (C6), 67.83 (POCH_2), 67.81 (POCH_2), 67.78 (POCH_2), 67.75 (POCH_2), 46.7 (OCH_2CH), 32.04 (CH_2), 31.98 (CH_2), 31.97 (CH_2), 31.91 (CH_2), 18.6(CH_2CH_3), 18.5 (CH_2CH_3), 13.6 (CH_2CH_3), 13.5 (CH_2CH_3). ^{31}P NMR (162 MHz, $CDCl_3$) δ -2.64. $[\alpha]_D$ +6.54 (c 1, $CHCl_3$). IR (film): 3063 cm-1 (w, C-H Ar), 2870 cm-1 (w, C-H Ar), 1726 cm-1 (s, C=O), 1268 cm-1 (s, C-O ester), 1098 cm-1, 1071 (s, C-O ether), 735 cm-1 (w), 710 cm-1 (s). HRMS (ESI) calc. for $C_{50}H_{57}O_{11}PNa$, 887.3531; found: $[M+Na]^+$, 887.3537.

Synthesis of Protected Oligosaccharides

General Procedure for Automated Glycan Assembly

All solutions were freshly-prepared and kept under argon during the automation run. Oven-heated, argon-flushed flasks were used to prepare all moisture-sensitive solutions.

Solution A (acidic wash / phosphate activation): TMSOTf (450 µL, 2.49 mmol) were dissolved in anhydrous DCM (40 mL).

Solution B (thioglycoside activation): NIS (1.35 g, 6.00 mmol) was dissolved in anhydrous DCM/dioxane (40 mL, v/v, 2:1), and TfOH (55.0 µL, 0.60 mmol) was added. The solution was kept at 0 °C by using an ice bath.

Solution C (Fmoc deprotection): A solution of 20% piperidine in DMF (v/v) was prepared.

Solution D (Lev deprotection): Hydrazine acetate (550 mg, 5.97 mmol) was dissolved in pyridine/AcOH/H_2O (40 mL, v/v, 32:8:2), unless stated otherwise.

Solutions E*j* (building blocks): 5, 6.5 or 8 equiv. of building block ***j*** were coevaporeted with toluene twice, kept under vacuum overnight and dissolved in 1.00 mL of anhydrous DCM.

<u>Automated Synthesis</u>

Photocleavable linker-functionalized resin **16** (37.8 mg, 12.5 µmol) was added into the reaction vessel, and swollen in 2 mL DCM for 20 min. All reagent lines needed for the synthesis were washed and primed. The resin was washed with DMF, THF, and DCM (3 x 2 mL, 25 s each).

To couple each building block ***j***, a cycle consistent in the sequential use of **Modules 1-3** was used:

1. **Module 1 – acidic wash:** DCM (2 mL) was added to the reaction vessel and the mixture was cooled to -20 °C. **Solution A** (1.00 mL) was delivered dropwise into the reaction vessel, and kept under Ar bubbling for 3 min. The reaction vessel was emptied and the resin was washed with DCM (2 mL, 25 s).
2. **Module 2 – glycosylation with building block *j*: Module 2.a** (for activation of thioglycoside donors) <u>or</u> **module 2.b** (for activation of phosphate donors) was used.
    a. **Module 2.a – thioglycoside donors: Solution E*j*** (1.00 mL, *n* equiv.) was delivered into the reaction vessel and the mixture was cooled to an incubation temperature (T_1). Then **solution B** (1.00 mL) was added into the reaction vessel dropwise. After the incubation time (t_1) was completed, the temperature was increased to a glycosylation temperature (T_2) and kept for a glycosylation

time (t_2) (*see* **Tables 1, S1**). The solution was drained and the resin was washed with dioxane (2 mL, 20 s) for 20 s and DCM (2 x 2 mL, 25 s). The temperature of the reaction vessel was increased to 25 °C.

b. **Module 2.b – phosphate donors: Solution E$_j$** (1.00 mL, *n* equiv.) was delivered into the reaction vessel and the mixture was cooled to an incubation temperature (T_1). Then **solution A** (1.00 mL) was added into the reaction vessel dropwise. After the incubation time (t_1) was completed, the temperature was increased to a glycosylation temperature (T_2) and kept for a glycosylation time (t_2) (*see* **Tables 1, S1**). The solution was drained and the resin was washed with DCM (6 x 2 mL, 15 s).

3. **Module 3 – deprotection: Module 3.a** (for Fmoc deprotection) <u>or</u> **module 3.b** (for Lev deprotection) was used.
    a. **Module 3.a:** After washing the resin with DCM (3 x 2 mL, 25 s), **solution C** (2 mL) was delivered into the reaction vessel, and kept under Ar bubbling for 5 min at 25 ºC. The solution was drained and the resin was washed with DMF (3 x 3 mL, 25 s) and DCM (5 x 2 mL, 25 s).
    b. **Module 3.b:** After washing the resin with DCM (6 x 2 mL, 25 s), DCM (1.3 mL) was delivered to the reaction vessel. **Solution D** (0.8 mL) was added and kept under Ar bubbling (pulsed bubbling, 2x 30 min) at 25 ºC. The solution was drained and the resin was washed with DMF (3 x 3 mL, 25 s) and DCM (5 x 2 mL, 25 s).

Note on the AGA of oligosaccharides that require the use of five building blocks. The automated oligosaccharide synthesizer has four lines for building block solutions. For the AGA of compounds **18**, **21-26**, **28** and **29** five different building blocks were used, and the following procedure was implemented:

I. Preparation of building block **solutions E$_j$** (*j* = **11**-**14**) and attachment of each solution to building block lines 1 to 4 (respectively).
II. One coupling cycle (**Modules 1-3**) was performed for building block **11**.
III. **Solution E$_{11}$** was removed from building block line 1 and the line was washed. **Solution E$_{15}$** was prepared and connected to building block line 1.
IV. AGA continued with the subsequent coupling cycles of building blocks **12**-**15** as above described.

Photocleavage and Purification

After the automated synthesis was completed, the resin was removed from the reaction vessel, suspended in DCM (20 mL), and photocleaved in a flow reactor. A Vapourtec E-Series easy-MedChem, equipped with a UV-150 Photochemical reactor was used. A UV-150 Medium-Pressure Mercury Lamp (arc length 27.9 cm, 450 W) surrounded by an UV filter (Pyrex, 50% transmittance at 305 nm). A Pump 11 Elite Series (Harvard Apparatus) syringe pump at a flow rate of 0.8 mL/min was used to pump the mixture through a FEP tubing (i.d. 3.0 inch, volume: 12 mL), at 20 °C. The reactor was washed with 20 mL DCM at a flow rate of 2.0 mL/min. The output solution was filtered to remove the resin, and the solvent was evaporated *in vacuo*. The crude material was analyzed by MALDI and analytical NP-HPLC. Purification was performed by NP-HPLC. HPLCs were performed on Agilent 1200 Series systems. A YMC-Diol-300-NP column (150 mm x 4.60 mm I.D.) was used for analytical NP-HPLC, with a flow rate of 1.00 mL/min and hexanes/EtOAc as eluent. A YMC-Pack Diol-300-NP column (150 mm x 20.0 mm I.D.) was used for preparative NP-HPLC, with a flow rate of 15.0 mL/min and hexanes/EtOAc as eluent. Unless stated otherwise, the gradient program detailed hereafter was used:

1. Isocratic 20% EtOAc in hexanes (5 min).
2. Linear gradient 20 to 55% EtOAc in hexanes (35 min).
3. Linear gradient to 100% EtOAc (10 min).

β-Stereoselectivity for the Nonreducing End Gal-GlcNAc Linkage

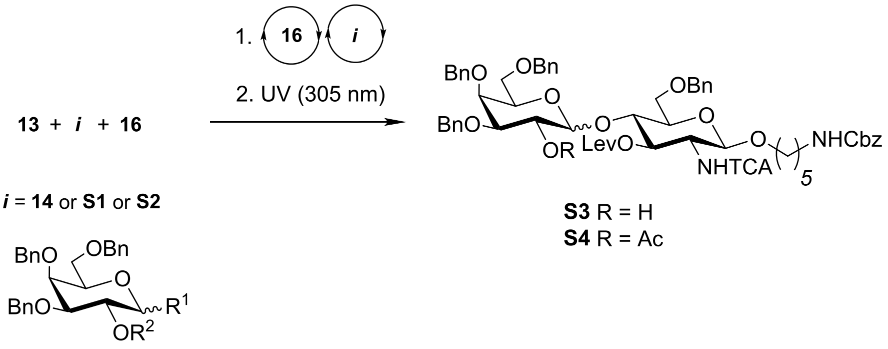

S3 R = H
S4 R = Ac

14 R^1 = $OP(O)(OBu)_2$, R^2=Fmoc
S1 R^1 = SEt, R^2=Fmoc
S2 R^1 = SEt, R^2=Ac

Scheme S1. Glycosyl donors tested for the synthesis of the nonreducing end Gal-GlcNAc linkage.

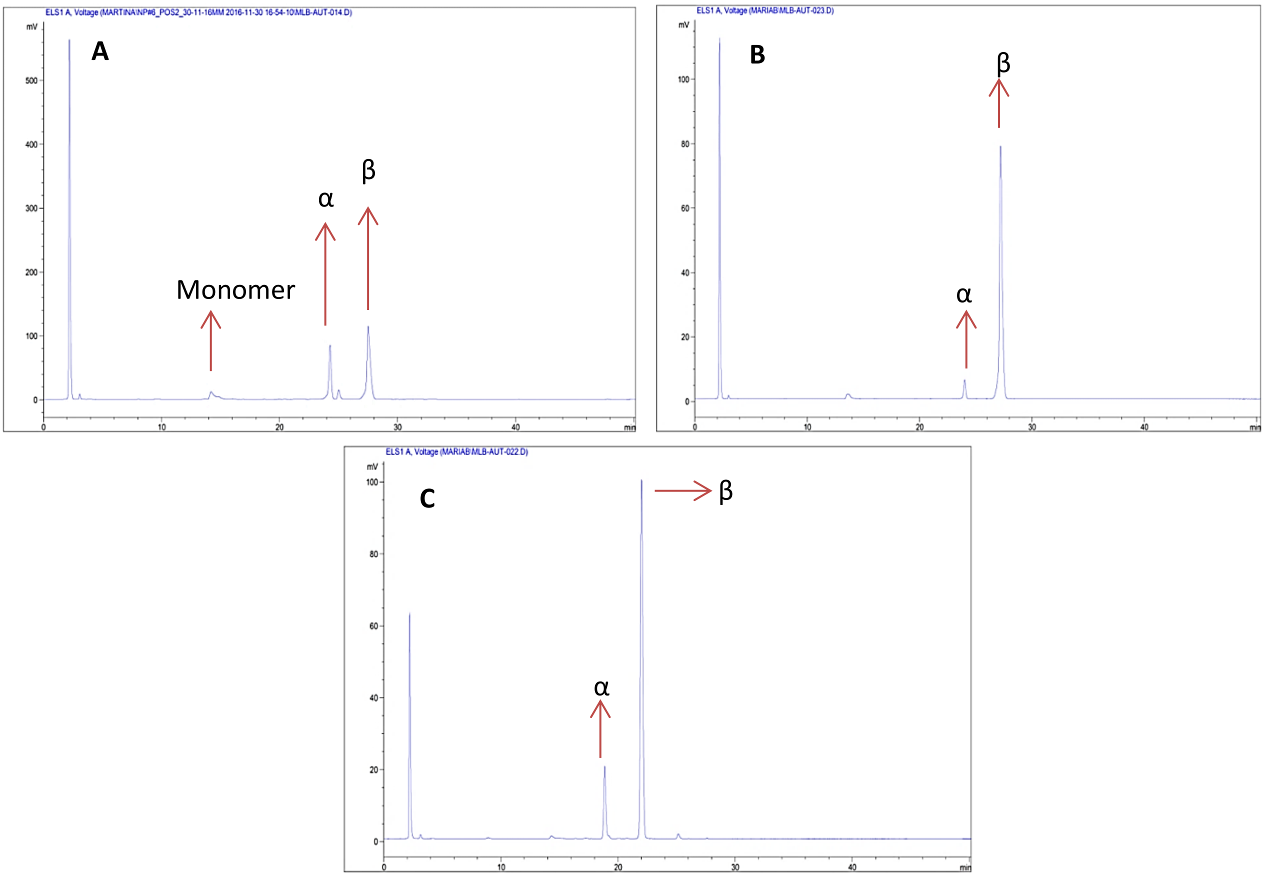

Figure S1. Analytical NP-HPLC chromatograms from the AGA of **S3** and **S4**. (reaction crude after photocleavage).a) Using **S1** as glycosyl donor, b) using **14** as glycosyl donor and c) using **S2** as glycosyl donor. Detection: ELSD.

Table S1. Reaction conditions and observed stereoselectivity for the AGA of the nonreducing end Gal-β(1-4)-GlcNAc fragment.

Entry	BB	# Equiv.	T_1 (°C)	t_1 (min)	T_2 (°C)	t_2 (min)	α/β ratio
1	**14**	5	-35	5	-15	30	1:5
2	**S1**	8	-20	5	0	20	1:1.5
3	**S2**	8	-20	5	0	20	1:3

T_1= incubation temperature; t_1= incubation time;

T_2= glycosylation temperature; t_2= glycosylation time.

Table S2. Anomeric coupling constants for the nonreducing end Gal-β(1-4)-GlcNAc fragment.

Product	H-1A			H-1B			m/z
	Δ (ppm)	$^3J_{H-H}$ (Hz)	$^1J_{C-H}$ (Hz)	δ (ppm)	$^3J_{H-H}$ (Hz)	$^1J_{C-H}$ (Hz)	
**S3-α**	5.20	4.10	173	4.62	8.30	166	1185.3662[1]
**S3-β**	4.53	-	162	4.21	7.60	165	1185.3650
**S4-α**	5.38	4.30	177	4.75	8.30	166	1227.3749[2]
**S4-β**	4.45	-	165	4.33	7.90	164	1227.3766

[1]Calc. for $C_{60}H_{69}Cl_3N_2O_{15}Na$, 1185.3656. [2]Calc. for $C_{62}H_{71}Cl_3N_2O_{16}Na$, 1127.3761.

N-Benzyloxycarbonyl-5-amino-pentyl 3,4,6-tri-*O*-benzyl-β-D-galactopyranosyl-(1→4)-6-*O*-benzyl-2-deoxy-2-*N*-trichloroacetyl-3-*O*-levunoyl-β-D-glucopyranoside (S3-β)

^{1}H NMR (600 MHz, $CDCl_3$) δ 7.37 – 7.22 (m, 25H), 7.13 (d, *J* = 8.5 Hz, 1H, NHTCA), 5.28 (t, *J* = 10.0 Hz, 1H), 5.08 (s, 2H), 4.82 (d, *J* = 11.5 Hz, 1H), 4.71 – 4.59 (m, 4H), 4.55 – 4.42 (m, 4H, **H-1**, 3 x CHH‘), 4.21 (d, *J* = 7.6 Hz, 1H, **H-1**), 3.94 (q, *J* = 9.1 Hz, 1H), 3.89 – 3.80 (m, 5H), 3.75 – 3.67 (m, 2H), 3.57 (t, *J* = 8.4 Hz, 1H), 3.55 – 3.51 (m, 1H), 3.45 – 3.37 (m, 2H), 3.28 (dd, *J* = 9.7, 2.8 Hz, 1H), 3.11 (m, 2H, CH_2-NHCbz), 2.55 – 2.35 (m, 4H, CH_2 Lev), 1.95 (s, 3H, CH_3 Lev), 1.52 – 1.27 (m, 6H, CH_2 pentane). HRMS (ESI) calc. for $C_{60}H_{69}Cl_3N_2O_{15}Na$, 1185.3656; found: 1185.3662.

Experimental Data for Protected Oligosaccharides 17-29

_N_-Benzyloxycarbonyl-5-amino-pentyl 3,4,6-tri-_O_-benzyl-β-D-galactopyranosyl-(1→4)-6-_O_-benzyl-2-deoxy-2-_N_-trichloroacetyl-3-_O_-levunoyl-β-D-glucopyranosyl-(1→3)-2-_O_-benzoyl-4,6-di-_O_-benzyl-β-D-galactopyranosyl-(1→4)-6-_O_-benzyl-2-deoxy-2-_N_-

trichloroacetyl-3-*O*-levunoyl-β-D-glucopyranosyl-(1→3)-2-*O*-benzoyl-4,6-di-*O*-benzyl-β-D-galactopyranosyl-(1→4)-2,3-di-*O*-benzoyl-6-*O*-benzyl-β-D-glucopyranoside (17)

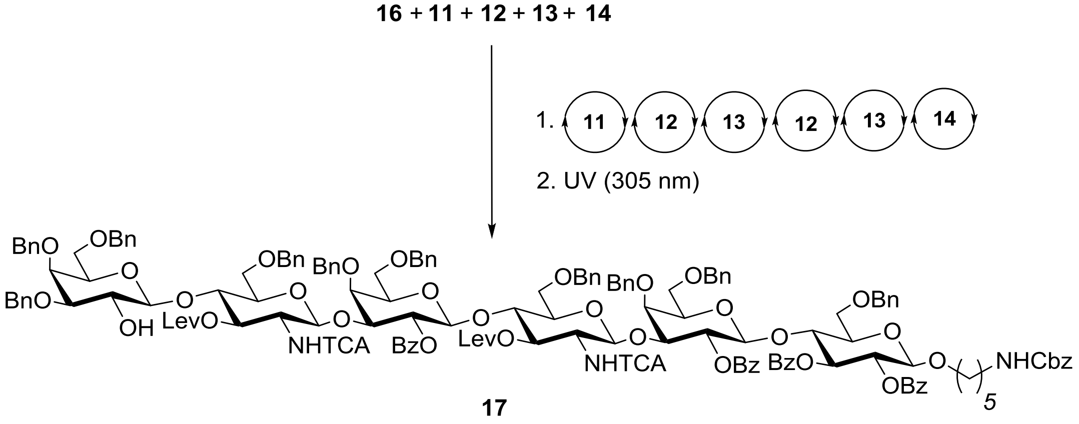

17

Yield: 55%, 16.1 mg

^{1}H NMR (600 MHz, $CDCl_3$) δ 7.91 – 7.80 (m, 8H), 7.60 – 7.52 (m, 2H), 7.48 – 7.07 (m, 65H), 6.36 (d, *J* = 9.2 Hz, 1H, NHTCA), 6.30 (d, *J* = 9.1 Hz, 1H, NHTCA), 5.52 (t, *J* = 9.3 Hz, 1H), 5.34 – 5.25 (m, 3H), 5.05 (s, 2H), 4.98 – 4.91 (m, 2H), 4.87 (d, *J* = 11.7 Hz, 1H), 4.82 (d, *J* = 11.3 Hz, 1H), 4.76 (dd, *J* = 10.4, 8.7 Hz, 1H), 4.70 (d, *J* = 11.9 Hz, 1H), 4.61 – 4.35 (m, 18H, including 5 x **H-1**), 4.31 (d, *J* = 11.7 Hz, 1H), 4.26 – 4.20 (m, 2H, including **H-1**), 4.13 (d, *J* = 12.0 Hz, 1H), 4.03 – 3.97 (m, 4H), 3.93 – 3.77 (m, 10H), 3.73 (dd, *J* = 9.8, 7.6 Hz, 1H), 3.68 (dd, *J* = 10.2, 3.0 Hz, 1H), 3.59 – 3.38 (m, 11H), 3.35 (dd, *J* = 11.0, 2.3 Hz, 2H), 3.19 – 3.13 (m, 1H), 2.93 – 2.82 (m, 3H, CH_2-NHCbz, H-6), 2.78 (t, *J* = 8.8 Hz, 1H, H-6'), 2.47 – 2.34 (m, 6H, CH_2 Lev), 2.32 – 2.24 (m, 2H, CH_2 Lev), 1.91 (s, 3H, CH_3 Lev), 1.89 (s, 3H, CH_3 Lev), 1.47 – 1.14 (m, 6H, CH_2 pentane). ^{13}C NMR (151 MHz, $CDCl_3$) δ 206.6 (*C*(O)CH_3), 206.4 (*C*(O)CH_3), 172.7 (O*C*(O)CH_2), 172.5 (O*C*(O)CH_2), 165.34 (C=O Bz), 165.28 (C=O Bz), 164.5 (C=O Bz), 164.4 (C=O Bz), 162.07, 162.03 (C=O Cbz, C=O TCA), 156.35 (C=O TCA), 139.1, 138.8, 138.7, 138.3, 138.12, 138.04, 137.94, 137.83, 137.79, 136.81, 133.76, 133.6, 133.2, 132.5, 130.6, 130.0, 129.8, 129.7, 129.5, 129.4, 128.91, 128.85, 128.67, 128.62, 128.61, 128.59, 128.58, 128.58, 128.4, 128.34, 128.31, 128.25, 128.22, 128.17, 128.10, 128.08, 128.04, 128.01, 127.94, 127.90, 127.74, 127.71, 127.69, 127.61, 127.2, 102.9 (**C-1**, $J_{C\text{-}H}$ = 163 Hz), 101.13 (**C-1**, $J_{C\text{-}H}$ = 165 Hz), 101.09 (**C-1**, $J_{C\text{-}H}$ = 163 Hz), 101.04 (**C-1**, $J_{C\text{-}H}$ = 161 Hz), 100.9 (**C-1**, $J_{C\text{-}H}$ = 163 Hz), 100.6 (**C-1**, $J_{C\text{-}H}$ = 163 Hz), 92.03 (*C*Cl_3), 91.95 (*C*Cl_3), 82.0, 79.0, 78.7, 76.0, 75.8, 75.27, 75.20, 75.1, 74.86, 74.84, 74.73, 74.65, 73.9, 73.65, 73.63, 73.59, 73.53, 73.49, 73.38, 73.1, 73.0, 72.8, 72.63, 72.61, 72.59, 72.4, 72.3, 72.2, 72.1, 71.5, 69.76, 68.3, 68.11, 68.05, 67.7, 67.5, 67.3, 66.6, 56.1, 55.8, 40.9 (CH_2-NHCbz), 37.8 (CH_2 Lev), 37.7 (CH_2 Lev), 29.78 (CH_3 Lev), 29.76 (CH_3 Lev), 29.5 (CH_2 pentane), 29.0 (CH_2 pentane), 27.99 (CH_2 Lev), 27.95 (CH_2 Lev), 23.2 (CH_2 pentane). HRMS (ESI) calc. for $C_{161}H_{167}Cl_{16}N_3O_{41}Na_2$, 1526.9495; found: 1526.9541.

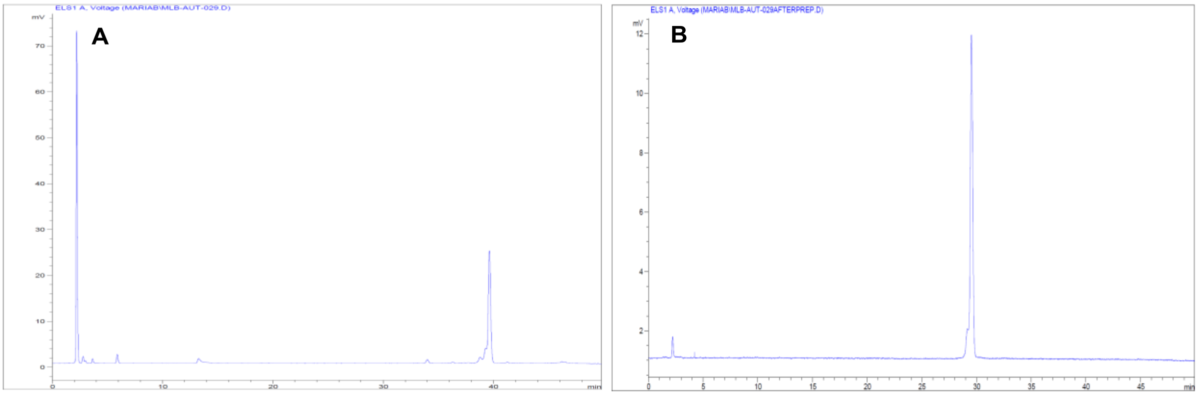

*Analytical NP-HPLC of (**A**) reaction crude after photocleavage and (**B**) the purified product (detection: ELSD)*

_N_-Benzyloxycarbonyl-5-amino-pentyl 2,3,4-tri-_O_-benzyl-α-L-fucopyranosyl-(1→2)-3,4,6-tri-_O_-benzyl-β-D-galactopyranosyl-(1→4)-6-_O_-benzyl-3-_O_-(2,3,4-tri-_O_-benzyl-α-L-fucopyranosyl)-2-deoxy-2-_N_-trichloroacetyl-β-D-glucopyranosyl-(1→3)-2-_O_-benzoyl-4,6-di-_O_-benzyl-β-D-galactopyranoside (18)

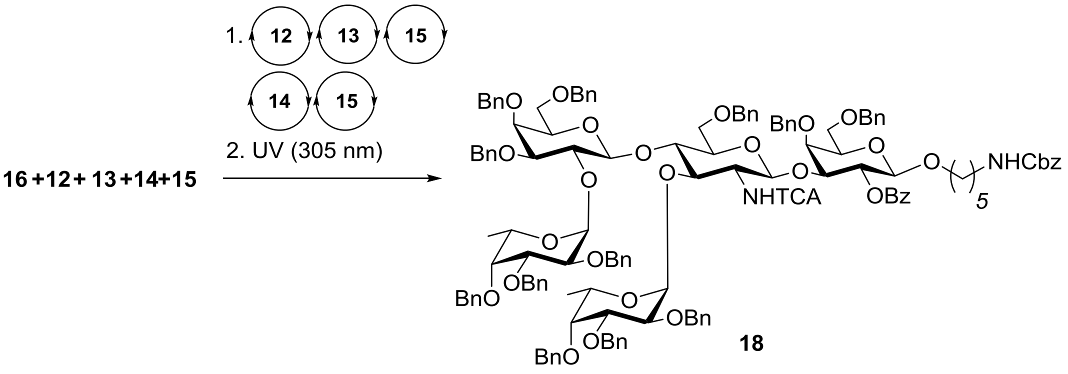

Yield: 51%, 14.9 mg

^{1}H NMR (600 MHz, $CDCl_3$) δ 7.99 – 7.93 (m, 2H), 7.37 – 7.13 (m, 66H), 7.09 – 7.03 (m, 2H), 6.69 (d, J = 7.4 Hz, 1H, N-H TCA), 5.68 (d, J = 3.8 Hz, 1H, **H-1α**), 5.51 (dd, J = 10.1, 7.8 Hz, 1H), 5.10 – 5.05 (m, 3H, including **H-1**), 5.03 – 4.94 (m, 3H, including **H-1**), 4.78 – 4.68 (m, 5H), 4.65 – 4.31 (m, 19H, including 2 x **H-1**), 4.21 – 4.12 (m, 3H), 4.09 – 3.99 (m, 4H), 3.95 (d, J = 3.2 Hz, 1H), 3.91 – 3.87 (m, 2H), 3.84 – 3.80 (m, 3H), 3.78 (dd, J = 10.2, 2.7 Hz, 1H), 3.70 – 3.51 (m, 9H), 3.35 – 3.27 (m, 3H), 3.18 (s, 1H), 2.83 (q, J = 6.9 Hz, 2H, NH-CH_2 linker), 1.47 – 0.99 (m, 6H, CH_2 pentane), 1.17 (d, J = 6.5 Hz, 3H, CH_3 Fuc), 1.06 (d, J = 6.4 Hz, 3H, CH_3 Fuc). ^{13}C NMR (151 MHz, $CDCl_3$) δ 165.5 (C=O Bz), 161.5 (C=O Cbz), 156.4 (C=O TCA), 139.3, 139.02, 138.97, 138.92, 138.70, 138.66, 138.13, 138.11, 138.05, 137.8, 136.9, 133.3, 130.4, 130.1, 128.8, 128.68, 128.66, 128.64, 128.58, 128.55,

128.52, 128.48, 128.44, 128.41, 128.33, 128.31, 128.25, 128.23, 128.19, 128.15, 128.12, 128.10, 128.07, 128.05, 128.04, 127.94, 127.93, 127.85, 127.8, 127.62, 127.59, 127.55, 127.50, 127.48, 127.37, 127.34, 127.24, 127.21, 127.19, 127.17, 126.29, 102.0 (**C-1**, $J_{C\text{-}H}$= 161 Hz), 100.5 (**C-1**, $J_{C\text{-}H}$= 165 Hz), 99.7 (**C-1**, $J_{C\text{-}H}$= 171 Hz), 98.1 (**C-1**, $J_{C\text{-}H}$= 172 Hz), 98.0 (**C-1**, $J_{C\text{-}H}$= 176 Hz), 91.9 (CCl_3 TCA), 84.2, 80.0, 79.46, 79.41, 78.56, 78.50, 76.5, 76.0, 75.7, 75.6, 75.19, 75.11, 75.05, 75.04, 73.9, 73.77, 73.71, 73.69, 73.57, 73.4, 73.1, 72.76, 72.71, 72.69, 72.5, 72.2, 71.2, 69.5, 68.9, 68.4, 67.9, 67.0, 66.7, 66.6, 61.1, 40.9 (CH_2-NHCbz), 29.5 (CH_2 pentane), 29.1 (CH_2 pentane), 23.2 (CH_2 pentane), 16.6 (CH_3 Fuc), 16.4 (CH_3 Fuc). HRMS (ESI) calc. for $C_{136}H_{145}Cl_3N_2O_{27}Na$, 2365.8993; found: 2365.9055.

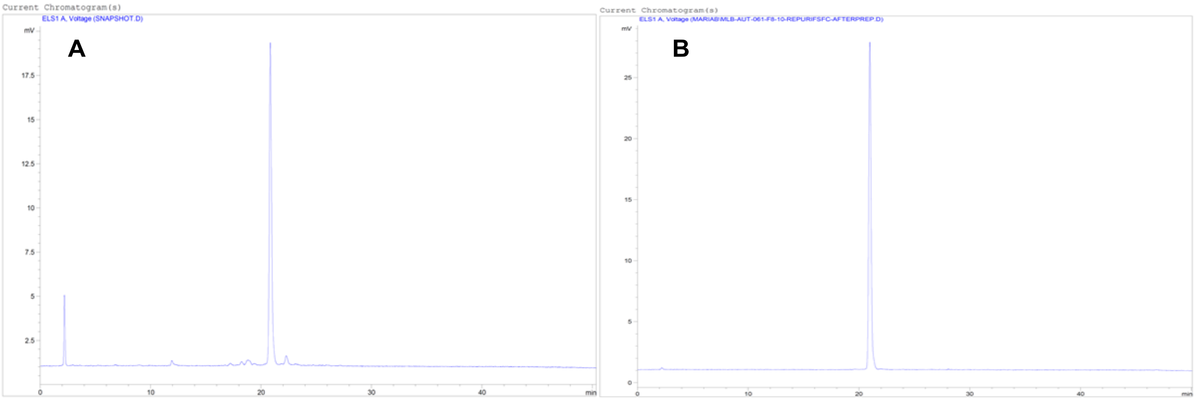

*Analytical NP-HPLC of (**A**) reaction crude after photocleavage and (**B**) the purified product (detection: ELSD).*

***N*-Benzyloxycarbonyl-5-amino-pentyl 2,3,4-tri-O-benzyl-α-L-fucopyranosyl-(1→2)-3,4,6-tri-*O*-benzyl-β-D-galactopyranosyl-(1→4)-6-*O*-benzyl-2-deoxy-2-*N*-trichloroacetyl-3-*O*-levunoyl-β-D-glucopyranoside (19)**

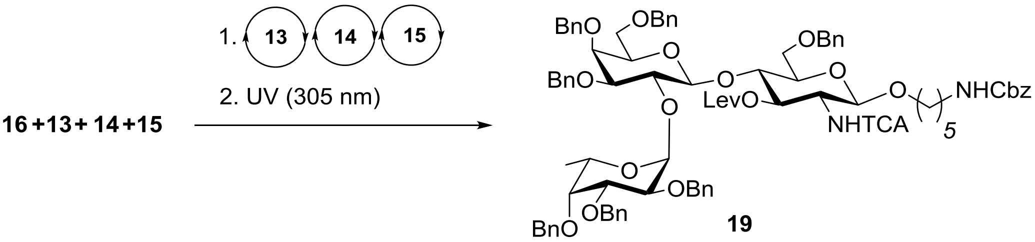

Yield: 57%, 11.3 mg

^{1}H NMR (600 MHz, $CDCl_3$) δ 7.41 – 7.11 (m, 38H), 7.07 (d, J = 7.0 Hz, 2H), 6.78 (d, J = 8.7 Hz, 1H, N-H TCA), 5.66 (d, J = 3.8 Hz, 1H, **H-1α**), 5.11 – 5.05 (m, 3H), 4.92 (d, J = 11.6 Hz, 1H), 4.77 – 4.68 (m, 3H), 4.66 – 4.61 (m, 3H), 4.55 – 4.46 (m, 7H, **H-1 β**, C*H₂*-Ph), 4.39 – 4.32 (m, 2H, **H-1β**, C*H*H'-Ph), 4.17 (q, J = 6.5 Hz, 1H, H-5 Fuc), 4.03 – 3.98 (m, 2H), 3.93 – 3.83 (m, 4H), 3.80 (dd, J = 10.2, 2.6 Hz, 1H), 3.71 (d, J = 3.0 Hz, 2H), 3.66 (s, 1H), 3.63 – 3.52 (m, 3H), 3.48 – 3.43 (m, 1H), 3.43 – 3.38 (m, 1H), 3.34 (dt, J = 9.2, 3.1 Hz, 1H), 3.16 (q,

J = 6.4 Hz, 2H, CH_2-NHCbz), 2.47 – 2.33 (m, 3H, 3H, CH_2 Lev), 2.16 – 2.08 (m, 1H, CH_2 Lev), 1.88 (s, 3H, CH_3 Lev), 1.68 – 1.24 (m, 6H, CH_2 pentane), 1.19 (d, J = 6.5 Hz, 3H, CH_3 Fuc). ^{13}C NMR (151 MHz, $CDCl_3$) δ 206.7 (C(O)CH_3), 172.8 (OC(O)CH_2), 162.1 (Cbz), 156.6 (TCA), 139.0, 138.9, 138.7, 138.6, 138.13, 138.06, 137.9, 136.8, 130.0, 128.68, 128.64, 128.60, 128.53, 128.49, 128.47, 128.43, 128.40, 128.38, 128.36, 128.30, 128.27, 128.24, 128.22, 128.19, 128.15, 128.12, 128.11, 128.08, 128.01, 128.00, 127.87, 127.85, 127.79, 127.76, 127.72, 127.68, 127.64, 127.62, 127.59, 127.55, 127.50, 127.47, 127.45, 127.34, 127.30, 127.0, 126.4, 122.2 (Ar), 101.06 (**C-1**, J_{C-H} = 176 Hz), 100.94 (**C-1**, J_{C-H} = 160 Hz), 97.3 (**C-1**, J_{C-H} = 166 Hz), 92.5 ($C$$Cl_3$ TCA), 84.2, 79.4, 78.0, 76.0, 75.9, 74.85, 74.83, 73.7, 73.6, 73.4, 73.2, 72.8, 72.7, 72.53, 72.47, 71.4, 71.3, 69.7, 68.2, 66.7, 66.6 (C-5 Fuc), 55.6, 41.0 ($C$$H_2$-NHCbz), 37.6 ($C$$H_2$ Lev), 29.8 ($C$$H_2$ pentane), 29.7 ($C$$H_3$ Lev), 29.1($C$$H_2$ pentane), 28.0 ($C$$H_2$ Lev), 23.4 ($C$$H_2$ pentane), 16.7 ($C$$H_3$ Fuc). HRMS (ESI) calc. for $C_{87}H_{97}Cl_3N_2O_{19}Na$, 1601.5643; found: 1601.5697.

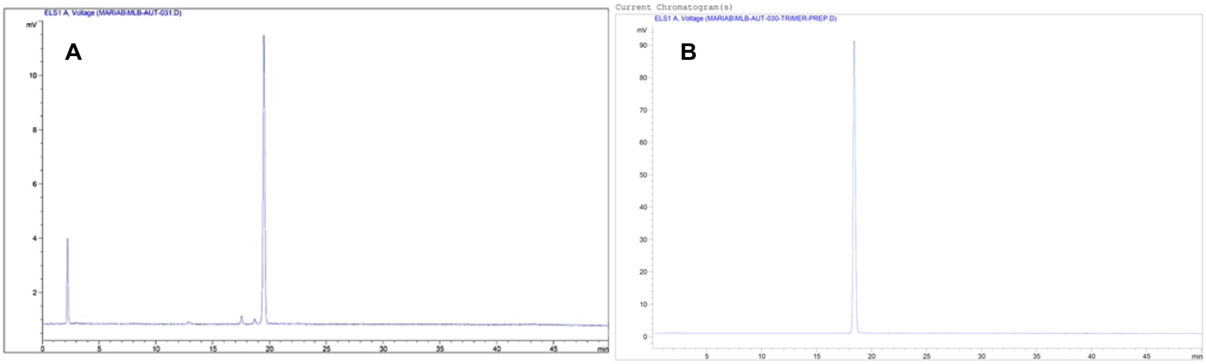

*Analytical NP-HPLC of (**A**) reaction crude after photocleavage and (**B**) the purified product (detection: ELSD)*

_N_-Benzyloxycarbonyl-5-amino-pentyl 3,4,6-tri-_O_-benzyl-β-D-galactopyranosyl- (1→4)-6-_O_-benzyl-3-_O_-(2,3,4-tri-O-benzyl-α-L-fucopyranosyl)-2-deoxy-2-_N_-trichloroacetyl-3-_O_-levunoyl-β-D-glucopyranoside (20)

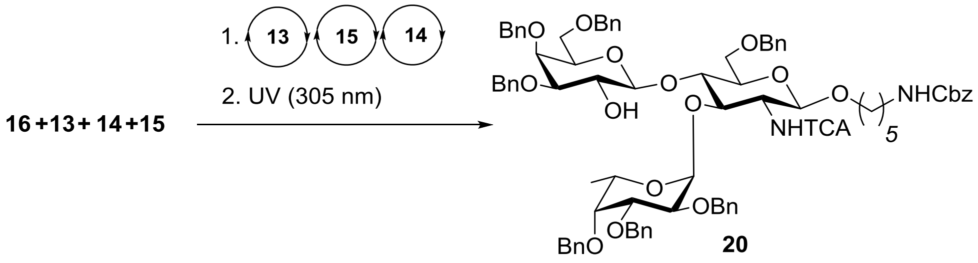

Yield: 48%, 8.90 mg

^{1}H NMR (600 MHz, $CDCl_3$) δ 7.43 – 7.17 (m, 40H), 6.92 (d, *J* = 7.7 Hz, 1H, N*H*TCA), 5.14 (d, *J* = 3.7 Hz, 1H, **H-1α**), 5.08 (s, 2H), 4.86 (d, *J* = 10.8 Hz, 1H), 4.81 (d, *J* = 7.8 Hz, 1H, **H-1**), 4.76 (dd, *J* = 11.9, 7.0 Hz, 3H), 4.70 – 4.59 (m, 6H), 4.50 – 4.46 (m, 3H), 4.44 (d, *J* = 7.7 Hz, 1H, **H-1**), 4.38 – 4.32 (m, 2H), 4.28 (t, *J* = 8.9 Hz, 1H), 4.11 (d, *J* = 11.3 Hz, 1H), 4.06 (t, *J* = 8.8 Hz, 1H), 3.99 – 3.92 (m, 3H), 3.89 (dd, *J* = 10.2, 2.7 Hz, 1H), 3.84 – 3.77 (m, 3H), 3.68 (t, *J* = 8.6 Hz, 1H), 3.64 – 3.59 (m, 2H), 3.57 (dt, *J* = 9.3, 3.0 Hz, 1H), 3.43 – 3.38 (m, 1H), 3.35 (dd, *J* = 8.7, 5.1 Hz, 1H), 3.33 (s, 1H), 3.28 (dd, *J* = 9.7, 2.9 Hz, 1H), 3.13 (q, *J* = 6.8 Hz, 2H, CH_2-NHCbz), 1.56 – 1.50 (m, 2H, CH_2 pentane), 1.49 – 1.42 (m, 2H, CH_2 pentane), 1.34 – 1.28 (m, 2H, CH_2 pentane), 1.02 (d, *J* = 6.5 Hz, 3H, CH_3 Fuc). ^{13}C NMR (151 MHz, $CDCl_3$) δ 161.6 (C=O Cbz), 156.5 (C=O TCA), 139.2, 139.0, 138.86, 138.84, 138.3, 138.2, 138.0, 136.8, 128.73, 128.70, 128.63, 128.63, 128.51, 128.49, 128.47, 128.40, 128.22, 128.18, 128.09, 128.05, 128.03, 127.99, 127.8, 127.77, 127.74, 127.72, 127.53, 127.51, 127.45, 127.3, 101.8 (**C-1**, J_{C-H} = 164 Hz), 99.5 (**C-1**, J_{C-H} = 165 Hz), 97.4 (**C-1**, J_{C-H} = 171 Hz), 92.5 (*C*Cl_3 TCA), 82.4, 79.7, 78.5, 76.3, 75.4, 75.2, 75.0, 74.2, 73.8, 73.48, 73.46, 73.3, 73.1, 72.6, 72.5, 71.6, 69.7, 68.6, 68.1, 66.74, 66.69, 59.3, 41.1 (*C*H_2-NHCbz), 29.8 (*C*H_2 pentane), 29.2 (*C*H_2 pentane), 23.4 (*C*H_2 pentane), 16.7 (*C*H_3 Fuc). HRMS (ESI) calc. for $C_{82}H_{91}Cl_3N_2O_{17}Na$, 1503.5276; found: 1503.5294.

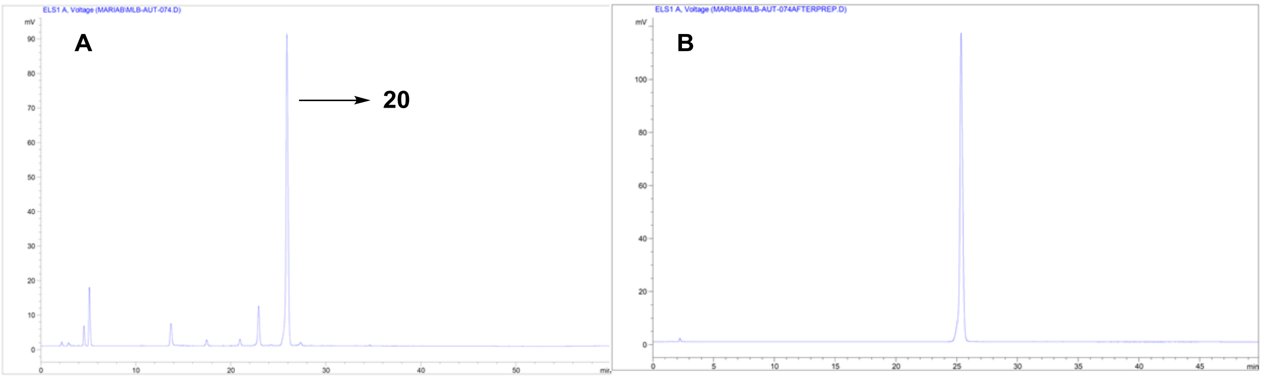

*Analytical NP-HPLC of (**A**) reaction crude after photocleavage and (**B**) the purified product (detection: ELSD)*

***N*-Benzyloxycarbonyl-5-amino-pentyl 2,3,4-tri-*O*-benzyl-α-L-fucopyranosyl-(1→2)-3,4,6-tri-*O*-benzyl-β-D-galactopyranosyl-(1→4)-6-*O*-benzyl-2-deoxy-2-*N*-trichloroacetyl-β-D-glucopyranosyl-(1→3)-2-*O*-benzoyl-4,6-di-*O*-benzyl-β-D-galactopyranosyl-(1→4)-2,3-di-*O*-benzoyl-6-*O*-benzyl-β-D-glucopyranoside (21)**

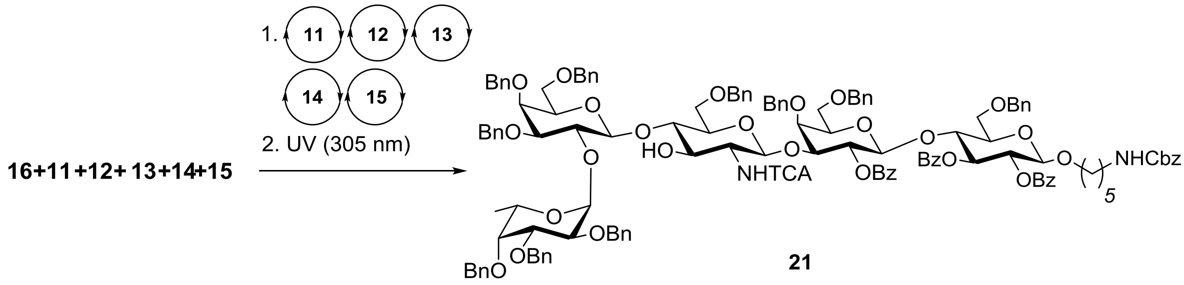

21

Yield: 44%, 13.0 mg

^{1}H NMR (600 MHz, $CDCl_3$) δ 7.94 (d, J = 7.8 Hz, 2H), 7.90 (d, J = 8.0 Hz, 2H), 7.82 (d, J = 7.8 Hz, 2H), 7.54 (t, J = 7.4 Hz, 1H), 7.48 – 7.06 (m, 61H), 7.01 (d, J = 7.5 Hz, 2H), 6.41 (d, J = 8.1 Hz, 1H, N*H*TCA), 5.69 (d, J = 3.9 Hz, 1H, **H-1**), 5.56 (t, J = 9.3 Hz, 1H), 5.42 (dd, J = 10.1, 7.8 Hz, 1H), 5.32 – 5.26 (m, 1H), 5.05 (s, 2H), 4.91 (d, J = 11.8 Hz, 1H), 4.84 (d, J = 11.6 Hz, 1H), 4.78 – 4.66 (m, 4H, including **H-1**), 4.56 – 4.47 (m, 8H, , including 2 x **H-1**), 4.43 (s, 2H), 4.40 (dd, J = 12.0, 5.0 Hz, 2H), 4.35 – 4.32 (m, 2H, including **H-1**), 4.27 (d, J = 12.3 Hz, 1H), 4.19 – 4.15 (m, 1H), 4.12 (q, J = 6.9, 6.3 Hz, 1H), 4.07 – 3.99 (m, 5H), 3.93 (s, 1H), 3.89 – 3.78 (m, 5H), 3.69 – 3.64 (m, 2H), 3.62 – 3.58 (m, 2H), 3.55 – 3.41 (m, 9H), 3.36 (m, 1H), 3.24 (dd, J = 8.7, 5.0 Hz, 1H), 2.94 – 2.79 (m, 4H), 1.50 – 1.15 (m, 6H, CH_2 pentane), 1.13 (d, J = 6.4 Hz, 3H, CH_3 Fuc). ^{13}C NMR (151 MHz, $CDCl_3$) δ 165.36 (C=O Bz), 165.30 (C=O Bz), 164.7 (C=O Bz), 161.9 (C=O Cbz), 156.4 (C=O TCA), 139.1, 138.89, 138.84, 138.4, 138.10, 138.08, 137.98, 137.8, 137.5, 136.8, 133.5, 133.2, 132.6, 130.5, 129.98, 129.92, 129.84, 129.78, 129.69, 128.74, 128.63, 128.60, 128.56, 128.50, 128.45, 128.45, 128.43, 128.39, 128.30, 128.28, 128.25, 128.22, 128.20, 128.17, 128.14, 128.11, 128.11, 127.99, 127.94, 127.87, 127.81, 127.80, 127.72, 127.64, 127.61, 127.55, 127.50, 127.39, 127.32, 127.30, 126.4, 102.0 (**C-1**, $J_{C\text{-}H}$ = 167 Hz), 101.1 (**C-1**, $J_{C\text{-}H}$ = 163 Hz), 100.9 (**C-1**, $J_{C\text{-}H}$ = 163 Hz), 100.2 (**C-1**, $J_{C\text{-}H}$ = 165 Hz), 97.8 (**C-1**, $J_{C\text{-}H}$ = 176 Hz), 92.4 (CCl_3), 84.0, 80.2, 79.6, 79.2, 77.9, 75.81, 75.77, 75.67, 75.3, 75.0, 74.82, 74.80, 74.78, 73.95, 73.91, 73.86, 73.6, 73.4, 73.2, 73.1, 73.0, 72.8, 72.7, 72.5, 72.2, 72.1, 71.5, 71.0, 69.8, 69.5, 68.49, 67.8, 67.2, 66.6, 66.5, 58.5, 40.9 (*C*H_2-NHCbz), 29.8 (*C*H_2 pentane), 29.0 (*C*H_2 pentane), 23.2 (*C*H_2 pentane), 16.8 (CH_3 Fuc). HRMS (ESI) calc. for $C_{136}H_{141}Cl_3N_2O_{30}Na$, 2409.8527; found: 2409.8503.

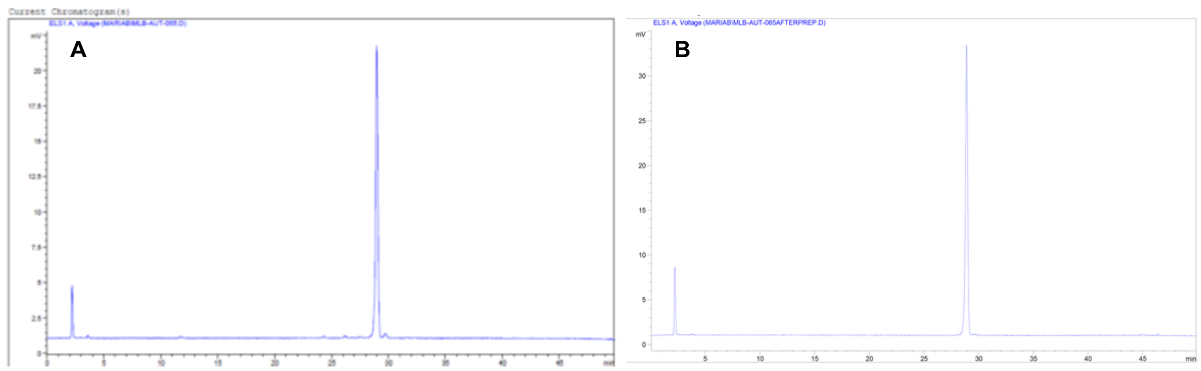

*Analytical NP-HPLC of (**A**) reaction crude after photocleavage and (**B**) the purified product (detection: ELSD)*

N-Benzyloxycarbonyl-5-amino-pentyl 3,4,6-tri-O-benzyl-β-D-galactopyranosyl- (1→4)-6-O-benzyl-3-O-(2,3,4-tri-O-benzyl-α-L-fucopyranosyl)-2-deoxy-2-N-trichloroacetyl-β-D-glucopyranosyl-(1→3)-2-O-benzoyl-4,6-di-O-benzyl-β-D-galactopyranosyl-(1→4)-2,3-di-O-benzoyl-6-O-benzyl-β-D-glucopyranoside (22)

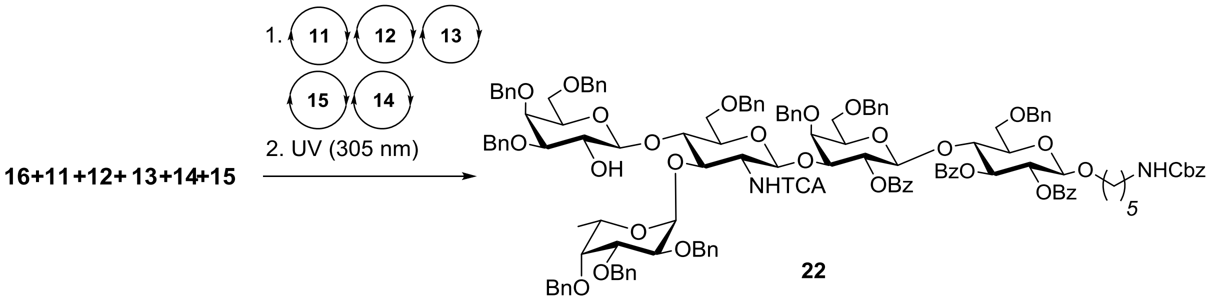

Yield: 48%, 14.5 mg

^{1}H NMR (600 MHz, $CDCl_3$) δ 7.91 – 7.85 (m, 4H), 7.80 (d, J = 8.2 Hz, 2H), 7.49 (t, J = 7.4 Hz, 1H), 7.44 (t, J = 7.4 Hz, 1H), 7.37 – 7.15 (m, 58H), 7.13 – 7.08 (m, 4H), 6.57 (d, J = 7.9 Hz, 1H, N-H TCA), 5.53 (t, J = 9.4 Hz, 1H), 5.36 (dd, J = 10.2, 7.8 Hz, 1H), 5.28 (dd, J = 9.7, 7.9 Hz, 1H), 5.08 – 5.01 (m, 3H, including **H-1**), 4.91 – 4.87 (m, 2H, including **H-1**), 4.80 (d, J = 10.9 Hz, 1H), 4.72 (d, J = 11.7 Hz, 1H), 4.65 – 4.61 (m, 3H), 4.59 – 4.55 (m, 2H), 4.53 – 4.41 (m, 7H, including 2 x **H-1**), 4.39 – 4.31 (m, 4H, including **H-1**), 4.28 – 4.22 (m, 3H), 4.12 – 3.97 (m, 6H), 3.94 (d, J = 2.9 Hz, 1H), 3.92 – 3.83 (m, 4H), 3.80 – 3.68 (m, 4H), 3.64 – 3.58 (m, 2H), 3.54 – 3.49 (m, 2H), 3.46 (dd, J = 11.1, 4.0 Hz, 1H), 3.41 – 3.33 (m, 3H), 3.32 – 3.28 (m, 1H), 3.26 – 3.22 (m, 2H), 2.92 – 2.84 (m, 3H, CH_2-NH linker, H-6), 2.77 (t, J = 8.7 Hz, 1H, H-6‘), 1.49 – 1.08 (m, 6H, CH_2 pentane), 0.94 (d, J = 6.5 Hz, 3H, CH_3 Fuc). ^{13}C NMR (151 MHz, $CDCl_3$) δ 165.39 (C=O Bz), 165.36 (C=O Bz), 164.8 (C=O Bz), 161.29 (C=O Cbz), 156.4 (C=O TCA), 139.3, 139.17, 139.12, 138.8, 138.7, 138.3, 138.22, 138.15, 138.09, 137.90, 136.9, 133.3, 133.2, 132.5, 130.6, 130.1, 129.95, 129.93, 129.84, 129.72, 128.71,

128.67, 128.64, 128.63, 128.59, 128.53, 128.49, 128.46, 128.45, 128.42, 128.39, 128.29, 128.23, 128.18, 128.10, 128.08, 128.05, 128.02, 127.98, 127.96, 127.87, 127.82, 127.74, 127.68, 127.62, 127.45, 127.40, 127.24, 127.20, 101.6 (**C-1**, $J_{C\text{-}H}$ = 163 Hz), 101.06 (**C-1**, $J_{C\text{-}H}$ = 162 Hz), 100.94 (**C-1**, $J_{C\text{-}H}$ = 165 Hz), 99.8 (**C-1**, $J_{C\text{-}H}$ = 167 Hz), 97.3 (**C-1**, $J_{C\text{-}H}$ = 171 Hz), 92.0 (*C*Cl_3), 82.4, 79.58, 78.53, 78.4, 76.17, 76.03, 75.4, 75.1, 75.01, 74.96, 74.92, 74.8, 74.0, 73.6, 73.45, 73.43, 73.41, 73.2, 73.14, 73.07, 72.9, 72.60, 72.56, 72.2, 71.4, 69.8, 68.6, 67.9, 67.8, 67.5, 66.62, 66.58, 60.0, 40.9 (*C*H_2-NHCbz), 29.5 (*C*H_2 pentane), 29.0 (*C*H_2 pentane), 23.2 (*C*H_2 pentane), 16.6 (CH_3 Fuc). HRMS (ESI) calc. for $C_{136}H_{141}Cl_3N_2O_{30}Na$, 2409.8527; found: 2409.8574.

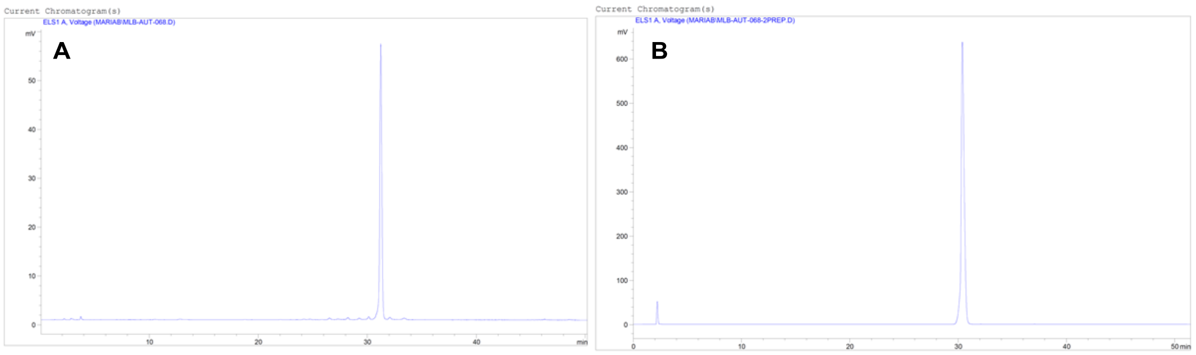

*Analytical NP-HPLC of (**A**) reaction crude after photocleavage and (**B**) the purified product (detection: ELSD).*

***N*-Benzyloxycarbonyl-5-amino-pentyl 2,3,4-tri-*O*-benzyl-α-L-fucopyranosyl-(1→2)-3,4,6-tri-*O*-benzyl-β-D-galactopyranosyl-(1→4)-6-*O*-benzyl-3-*O*-(2,3,4-tri-*O*-benzyl-α-L-fucopyranosyl)-2-deoxy-2-*N*-trichloroacetyl-β-D-glucopyranosyl-(1→3)-2-*O*-benzoyl-4,6-di-*O*-benzyl-β-D-galactopyranosyl-(1→4)-2,3-di-*O*-benzoyl-6-*O*-benzyl-β-D-glucopryanoside (23)**

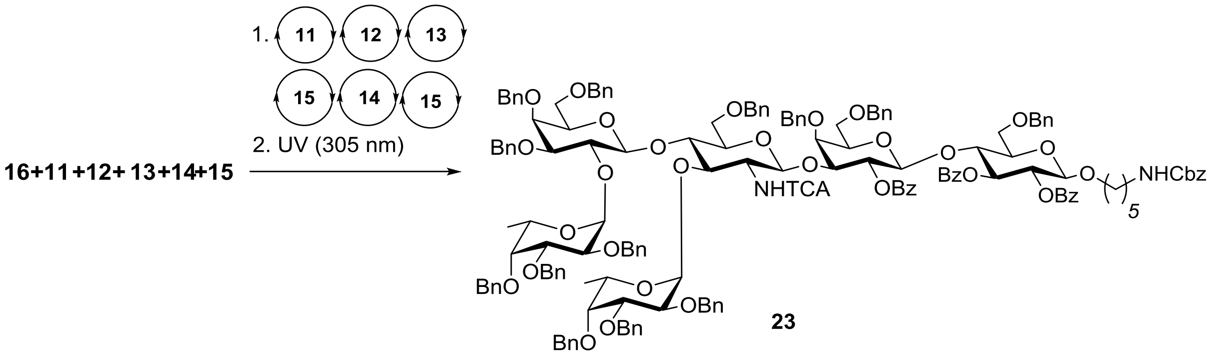

Yield: 65%, 23.1 mg

^{1}H NMR (600 MHz, $CDCl_3$) δ 7.91 (td, *J* = 8.4, 1.4 Hz, 4H), 7.82 – 7.79 (m, 2H), 7.48 – 7.44 (m, 2H), 7.36 – 7.08 (m, 75H), 7.06 – 7.03 (m, 2H), 6.66 (d, *J* = 7.2 Hz, 1H, N*H*TCA), 5.65 (d, *J* = 3.9 Hz, 1H, **H-1**), 5.55 (t, *J* = 9.4 Hz, 1H), 5.40 (dd, *J* = 10.1, 7.8 Hz, 1H), 5.28 (dd, *J* = 9.8, 7.9 Hz, 1H), 5.04 (m, 3H, including **H-1**), 4.98 – 4.90 (m, 3H, including **H-1**), 4.73 (m, 3H, including **H-1**), 4.70 – 4.60 (m, 3H), 4.57 – 4.45 (m, 13H, including 2 x **H-1**), 4.42 (d, *J* = 11.3 Hz, 1H), 4.36 – 4.26 (m, 6H), 4.18 (q, *J* = 6.3 Hz, 1H), 4.10 (t, *J* = 8.9 Hz, 1H), 4.07 – 3.98 (m, 6H), 3.92 (d, *J* = 2.9 Hz, 1H), 3.89 – 3.82 (m, 3H), 3.77 (m, 4H), 3.67 – 3.63 (m, 2H), 3.54 (td, *J* = 7.4, 6.4, 3.3 Hz, 2H), 3.50 – 3.46 (m, 2H), 3.44 (s, 1H), 3.42 – 3.23 (m, 6H), 3.16 (s, 1H), 2.93 – 2.86 (m, 3H, CH_2-NHCbz, H-6), 2.80 (t, *J* = 8.7 Hz, 1H, H-6‘), 1.49 – 1.39 (m, 4H, CH_2 pentane), 1.17 (d, *J* = 6.5 Hz, 3H, CH_3 Fuc), 1.23 – 1.06 (m, 2H, CH_2 pentane), 1.04 (d, *J* = 6.5 Hz, 3H CH_3 Fuc). ^{13}C NMR (151 MHz, $CDCl_3$) δ 165.36 (C=O Bz), 165.34 (C=O Bz), 164.9 (C=O Bz), 161.3 (C=O Cbz), 156.4 (C=O TCA), 139.35, 139.33, 139.02, 138.93, 138.91, 138.71, 138.68, 138.4, 138.13, 138.08, 137.8, 136.9, 133.3, 133.2, 132.5, 130.5, 130.2, 129.98, 129.93, 129.8, 129.7, 128.8, 128.63, 128.61, 128.57, 128.53, 128.50, 128.46, 128.44, 128.43, 128.3, 128.22, 128.20, 128.18, 128.16, 128.14, 128.11, 128.09, 128.03, 127.86, 127.84, 127.83, 127.78, 127.76, 127.72, 127.63, 127.60, 127.58, 127.57, 127.47, 127.35, 127.32, 127.30, 127.23, 127.20, 126.3, 101.0 (**C-1**, $J_{C\text{-}H}$ = 167 Hz), 100.9 (**C-1**, $J_{C\text{-}H}$ = 160 Hz), 100.4 (**C-1**, $J_{C\text{-}H}$ = 168 Hz), 99.4 (**C-1**, $J_{C\text{-}H}$ = 169 Hz), 98.02 (**C-1**, $J_{C\text{-}H}$ = 171 Hz), 97.96 (**C-1**, $J_{C\text{-}H}$ = 175 Hz), 91.8 (CCl_3 TCA), 84.1, 80.1, 79.3, 79.2, 78.6, 78.5, 76.3, 76.0, 75.8, 75.6, 75.05, 75.02, 74.96, 74.8, 73.8, 73.7, 73.59, 73.55, 73.52, 73.37, 73.32, 73.26, 73.06, 72.89, 72.77, 72.72, 72.6, 72.5, 72.2, 71.2, 69.8, 68.3, 67.90, 67.88, 67.4, 67.0, 66.68, 66.62, 61.2, 49.7, 40.9 (CH_2-NHCbz), 29.5 (CH_2 pentane), 29.0 (CH_2 pentane), 23.2 (CH_2 pentane), 16.6 (CH_3 Fuc), 16.3 (CH_3 Fuc). HRMS (ESI) calc. for $C_{163}H_{169}Cl_3N_2O_{34}Na$, 2826.0515; found: 2826.0513.

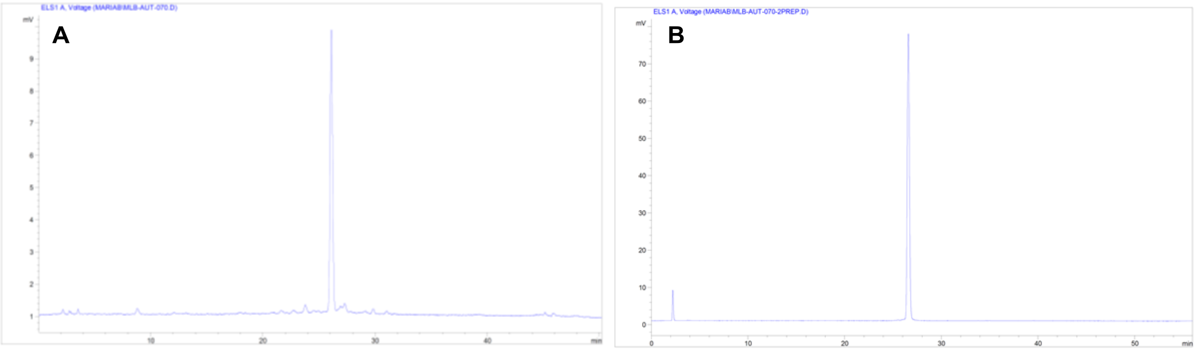

*Analytical NP-HPLC of (**A**) reaction crude after photocleavage and (**B**) the purified product (detection: ELSD).*

_N_-Benzyloxycarbonyl-5-amino-pentyl 2-_O_-benzoyl-4,6-di-_O_-benzyl-β-D-galactopyranosyl-(1→4)-6-_O_-benzyl-2-deoxy-2-_N_-trichloroacetyl-3-_O_-levunoyl-β-D-glucopyranosyl-(1→3)-2-_O_-benzoyl-4,6-di-_O_-benzyl-β-D-galactopyranosyl-(1→4)-2,3-di-_O_-benzoyl-6-_O_-benzyl-β-D-glucopyranoside (24)

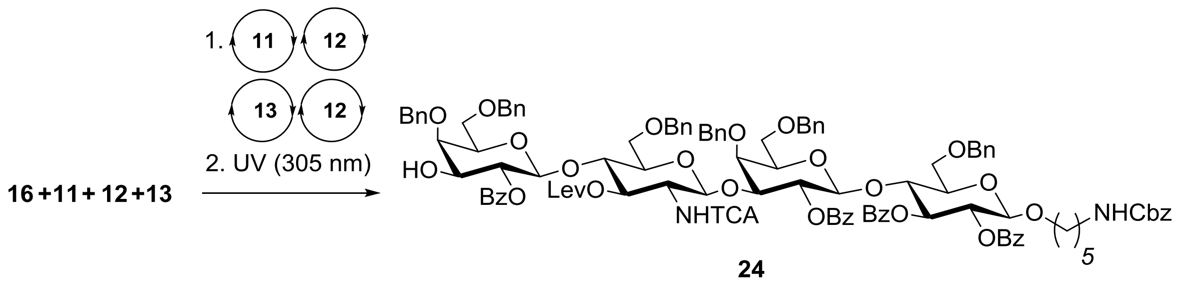

Yield: 50%, 13.0 mg

^{1}H NMR (600 MHz, $CDCl_3$) δ 7.94 – 7.88 (m, 4H), 7.85 – 7.81 (m, 4H), 7.58 – 7.52 (m, 2H), 7.47 – 7.10 (m, 45H), 6.36 (d, J = 9.2 Hz, 1H, NHTCA), 5.53 (t, J = 9.4 Hz, 1H), 5.34 (dd, J = 10.2, 7.8 Hz, 1H), 5.29 (dd, J = 9.8, 7.9 Hz, 1H), 5.09 – 5.02 (m, 3H), 4.89 (d, J = 11.7 Hz, 1H), 4.83 (dd, J = 10.7, 8.8 Hz, 1H), 4.65 (s, 2H), 4.56 – 4.45 (m, 6H), 4.42 (dd, J = 12.5, 8.0 Hz, 2H), 4.34 (d, J = 11.7 Hz, 1H), 4.23 (dd, J = 12.1, 2.0 Hz, 2H), 4.06 – 3.98 (m, 3H), 3.91 – 3.83 (m, 4H), 3.80 – 3.73 (m, 2H), 3.66 (t, J = 8.2 Hz, 1H), 3.63 – 3.58 (m, 4H), 3.54 – 3.46 (m, 2H), 3.42 – 3.38 (m, 2H), 3.37 – 3.32 (m, 1H), 3.29 – 3.25 (m, 2H), 2.92 – 2.84 (m, 3H, CH_2-NHCbz, H-6), 2.79 (t, J = 8.8 Hz, 1H, H-6'), 2.59 – 2.50 (m, 1H, CH*H'* Lev), 2.47 – 2.40 (m, 3H, CH_2 Lev), 1.98 (s, 3H, CH_3 Lev), 1.51 – 1.38 (m, 2H), 1.30 – 1.26 (m, 2H), 1.19 – 1.10 (m, 2H). ^{13}C NMR (151 MHz, $CDCl_3$) δ 206.3 (*C*(O)CH_3), 172.5 (OC(O)CH_2), 166.3 (C=O Bz), 165.35 (C=O Bz), 165.32 (C=O Bz), 164.5 (C=O Bz), 162.1 (C=O TCA), 156.4 (C=O Cbz), 139.1, 138.3, 138.12, 138.09, 137.8, 137.6, 136.8, 133.7, 133.5, 133.2, 132.5, 130.5, 130.0, 129.86, 129.82, 129.68, 129.64, 129.5, 128.9, 128.73, 128.68, 128.68, 128.65, 128.62, 128.59, 128.47, 128.45, 128.24, 128.22, 128.18, 128.12, 128.02, 127.95, 127.88, 127.79, 127.74, 127.3, 101.1 (**C-1**, $J_{C\text{-}H}$ = 161 Hz) 100.88 (**C-1**, $J_{C\text{-}H}$ = 164 Hz), 100.85 (**C-1**, $J_{C\text{-}H}$ = 164 Hz), 100.4 (**C-1**, $J_{C\text{-}H}$ = 161 Hz), 92.0 (*C*Cl_3 TCA), 78.8, 76.4, 75.8, 75.6, 75.2, 75.0, 74.9, 74.7, 74.39, 74.37, 73.7, 73.6, 73.5, 73.4, 73.3, 73.2, 73.05, 73.01, 72.8, 72.3, 72.1, 69.8, 67.67, 67.66, 67.2, 66.6, 40.9 (*C*H_2-NHCbz), 37.9 (*C*H_2 Lev), 29.8 (*C*H_3 Lev), 29.5 (*C*H_2 pentane), 29.0 (*C*H_2 pentane), 28.0 (*C*H_2 Lev), 23.2 (*C*H_2 pentane). HRMS (ESI) calc. for $C_{114}H_{117}Cl_3N_2O_{29}Na_2$, 1064.3296; found: 1064.3237.

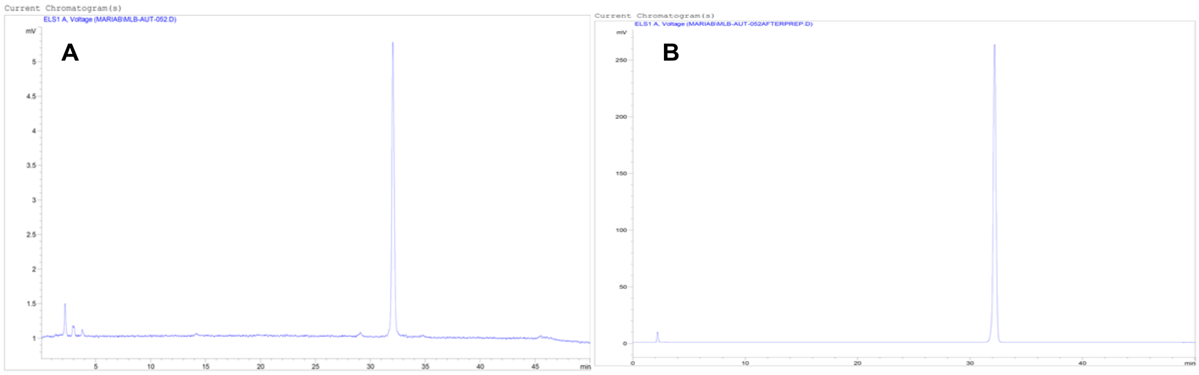

*Analytical NP-HPLC of (**A**) reaction crude after photocleavage and (**B**) the purified product (detection: ELSD).*

N-Benzyloxycarbonyl-5-amino-pentyl 3,4,6-tri-O-benzyl-β-D-galactopyranosyl- (1→4)-6-O-benzyl-3-O-(2,3,4-tri-O-benzyl-α-L-fucopyranosyl)-2-deoxy-2-N-trichloroacetyl-β-D-glucopyranosyl-(1→3)-2-O-benzoyl-4,6-di-O-benzyl-β-D-galactopyranosyl-(1→4)-6-O-benzyl-3-O-(2,3,4-tri-O-benzyl-α-L-fucopyranosyl)-2-deoxy-2-N-trichloroacetyl-β-D-glucopyranosyl-(1→3)-2-O-benzoyl-4,6-di-O-benzyl-β-D-galactopyranosyl-(1→4)-2,3-di-O-benzoyl-6-O-benzyl-β-D-glucopyranoside (25)

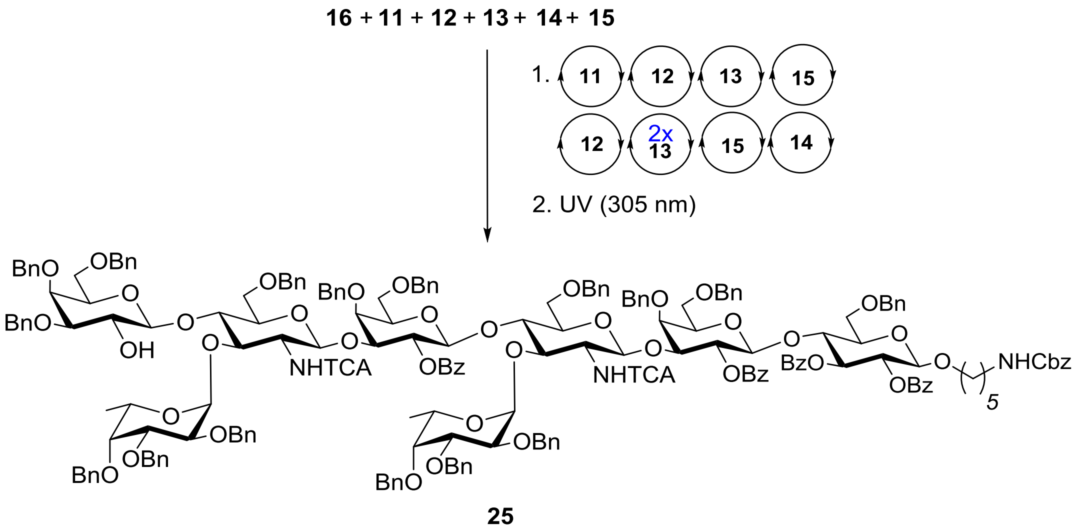

25

Yield: 28%, 12.9 mg

^{1}H NMR (600 MHz, $CDCl_3$) δ 7.90 (d, *J* = 8.0 Hz, 4H), 7.85 (d, *J* = 7.4 Hz, 2H), 7.83 – 7.80 (m, 2H), 7.53 – 7.06 (m, 97H), 6.59 (d, *J* = 8.1 Hz, 1H, NHTCA), 6.44 (d, *J* = 7.9 Hz, 1H, NHTCA), 5.54 (t, *J* = 9.3 Hz, 1H), 5.39 – 5.26 (m, 3H), 5.09 – 5.03 (m, 4H, including 2 x **H-1**), 4.98 (d, *J* = 10.9 Hz, 1H), 4.94 (d, *J* = 7.4 Hz, 1H, **H-1**), 4.82 (d, *J* = 10.8 Hz, 1H), 4.78 – 4.72 (m, 2H), 4.65 – 4.56 (m, 7H), 4.54 – 4.43 (m, 12H, including 3 x **H-1**), 4.39 (m, 4H, including 2 x **H-1**), 4.35 – 4.31 (m, 1H), 4.29 – 4.20 (m, 7H), 4.17 – 4.15 (m, 1H), 4.13 – 4.09 (m, 2H),

4.07 – 3.99 (m, 4H), 3.97 – 3.88 (m, 6H), 3.86 (dd, *J* = 10.1, 3.7 Hz, 1H), 3.82 – 3.75 (m, 6H), 3.75 – 3.68 (m, 3H), 3.67 – 3.55 (m, 6H), 3.53 – 3.45 (m, 3H), 3.42 – 3.34 (m, 4H), 3.32 (dd, *J* = 8.9, 5.0 Hz, 1H), 3.28 – 3.23 (m, 2H), 3.20 (s, 1H), 3.17 (dd, *J* = 8.3, 5.1 Hz, 1H), 3.07 – 3.02 (m, 1H), 2.93 – 2.85 (m, 2H, CH_2-NHCbz), 2.83 – 2.79 (m, 1H), 2.72 (t, *J* = 8.7 Hz, 1H), 1.50 – 1.37 (m, 2H, CH_2 pentane), 1.33 – 1.23 (m, 2H, CH_2 pentane), 1.15 (m, 2H, CH_2 pentane), 1.09 (d, *J* = 6.5 Hz, 3H, CH_3 Fuc), 0.96 (d, *J* = 6.4 Hz, 3H, CH_3 Fuc). ^{13}C NMR (151 MHz, $CDCl_3$) δ 165.37 (C=O Bz), 165.32 (C=O Bz), 164.7 (C=O Bz), 164.5 (C=O Bz), 161.2 (C=O Cbz), 156.4 (2x C=O TCA), 139.37, 139.37, 139.34, 139.14, 139.11, 139.05, 138.8, 138.7, 138.6, 138.4, 138.3, 138.1, 138.02, 137.95, 137.94, 137.87, 136.86, 133.5, 133.4, 133.2, 132.4, 130.6, 130.0, 129.9, 129.8, 129.7, 129.1, 128.8, 128.7, 128.63, 128.60, 128.55, 128.52, 128.49, 128.48, 128.44, 128.41, 128.38, 128.33, 128.32, 128.26, 128.20, 128.17, 128.15, 128.12, 128.11, 128.09, 128.02, 128.01, 127.99, 127.90, 127.88, 127.86, 127.84, 127.71, 127.68, 127.65, 127.61, 127.48, 127.47, 127.44, 127.3, 127.18, 127.17, 101.7 (**C-1**, $J_{C\text{-}H}$ = 162 Hz), 101.06 (**C-1**, $J_{C\text{-}H}$ = 162 Hz), 101.04 (**C-1**, $J_{C\text{-}H}$ = 162 Hz), 100.4 (**C-1**, $J_{C\text{-}H}$ = 166 Hz), 100.24 (**C-1**, $J_{C\text{-}H}$ = 162 Hz), 100.17 (**C-1**, $J_{C\text{-}H}$ = 167 Hz), 97.4 (**C-1**, $J_{C\text{-}H}$ = 171 Hz), 97.1 (**C-1**, $J_{C\text{-}H}$ = 171 Hz), 92.08 (CCl_3), 92.06 (CCl_3), 82.5, 79.6, 79.3, 78.9, 78.8, 78.48, 78.45, 76.1, 75.83, 75.80, 75.4, 75.17, 75.14, 75.10, 74.8, 74.7, 74.2, 74.0, 73.7, 73.6, 73.5, 73.44, 73.42, 73.41, 73.3, 73.13, 73.10, 73.08, 73.06, 73.02, 73.02, 72.97, 72.68, 72.64, 72.56, 72.48, 72.2, 71.4, 69.7, 68.6, 67.89, 67.87, 67.86, 67.78, 67.5, 66.8, 66.6, 59.9, 59.0, 40.9 (CH_2-NHCbz), 29.5 (CH_2 pentane), 29.0 (CH_2 pentane), 23.2 (CH_2 pentane), 16.6 (CH_3 Fuc), 16.4 (CH_3 Fuc). HRMS (ESI) calc. for $C_{205}H_{211}Cl_6N_3O_{45}Na_2$, 1845.1115; found: 1845.1084.

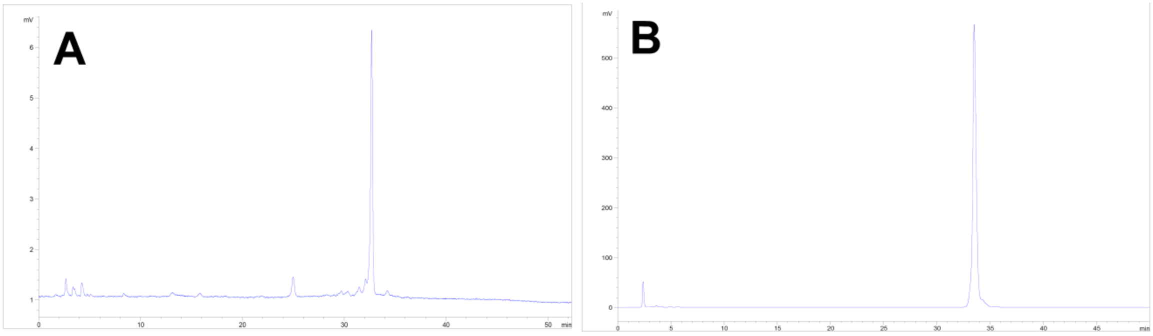

*Analytical NP-HPLC of (**A**) reaction crude after photocleavage and (**B**) the purified product (detection: ELSD).*

***N*-Benzyloxycarbonyl-5-amino-pentyl 2,3,4-tri-*O*-benzyl-α-L-fucopyranosyl-(1→2)-3,4,6-tri-*O*-benzyl-β-D-galactopyranosyl-(1→4)-6-*O*-benzyl-3-*O*-(2,3,4-tri-*O*-benzyl-α-L-fucopyranosyl)-2-deoxy-2-*N*-trichloroacetyl-β-D-glucopyranosyl-(1→3)-2-*O*-benzoyl-4,6-di-*O*-benzyl-β-D-galactopyranosyl-(1→4)-6-*O*-benzyl-3-*O*-(2,3,4-tri-*O*-benzyl-α-L-fucopyranosyl)-2-deoxy-2-*N*-trichloroacetyl-β-D-glucopyranosyl-(1→3)-2-*O*-benzoyl-**

4,6-di-*O*-benzyl-β-D-galactopyranosyl-(1→4)-2,3-di-*O*-benzoyl-6-*O*-benzyl-β-D-glucopyranoside (26)

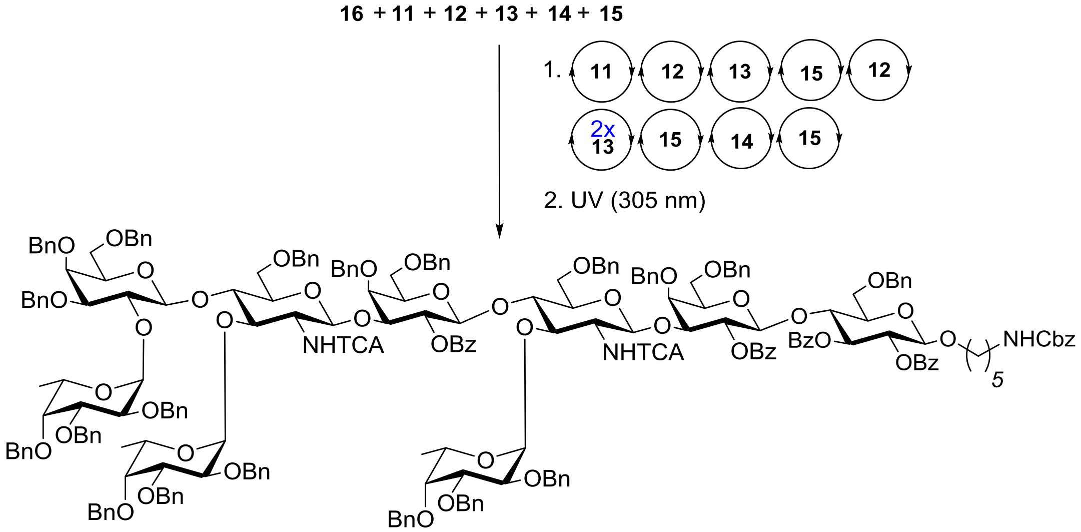

26

Yield: 28%, 14.6 mg

^{1}H NMR (600 MHz, $CDCl_3$) δ 7.95 – 7.78 (m, 8H), 7.49 (tt, *J* = 7.3, 1.3 Hz, 1H), 7.45 – 7.41 (m, 2H), 7.39 – 7.03 (m, 109H), 6.66 (d, *J* = 7.3 Hz, 1H, NHTCA), 6.44 (d, *J* = 8.2 Hz, 1H, NHTCA), 5.66 (d, *J* = 3.9 Hz, 1H, **H-1**), 5.52 (t, *J* = 9.3 Hz, 1H), 5.40 (dd, *J* = 10.2, 8.0 Hz, 1H), 5.33 – 5.25 (m, 2H), 5.11 (d, *J* = 7.5 Hz, 1H, **H-1**), 5.06 (d, *J* = 3.8 Hz, 1H, **H-1**), 5.04 (s, 2H), 4.99 (d, *J* = 10.9 Hz, 1H), 4.97 – 4.93 (m, 2H, including **H-1**), 4.78 – 4.70 (m, 4H), 4.69 – 4.62 (m, 3H), 4.58 – 4.20 (m, 31H, including 5 x **H-1**), 4.14 – 4.09 (m, 1H), 4.09 – 4.05 (m, 2H), 4.04 – 3.99 (m, 4H), 3.92 (d, *J* = 4.6 Hz, 2H), 3.89 – 3.75 (m, 12H), 3.70 – 3.61 (m, 6H), 3.60 – 3.56 (m, 3H), 3.54 – 3.44 (m, 5H), 3.43 – 3.36 (m, 3H), 3.35 – 3.31 (m, 2H), 3.28 (dd, *J* = 8.9, 5.0 Hz, 1H), 3.19 – 3.14 (m, 3H), 3.05 (d, *J* = 7.7 Hz, 1H), 2.91 – 2.85 (m, 2H, CH_2-NHCbz), 2.79 (dd, *J* = 8.9, 4.7 Hz, 1H), 2.69 (t, *J* = 8.7 Hz, 1H), 1.49 – 1.11 (m, 6H, CH_2 pentane), 1.21 (d, *J* = 6.5 Hz, 3H, CH_3 Fuc), 1.09 (d, *J* = 6.4 Hz, 3H, CH_3 Fuc), 1.04 (d, *J* = 6.4 Hz, 3H, CH_3 Fuc). ^{13}C NMR (151 MHz, $CDCl_3$) δ 165.37 (C=O Bz), 165.34 (C=O Bz), 164.68 (C=O Bz), 164.67 (C=O Bz), 161.27, 161.22, 156.4 (C=O Cbz, 2x C=O TCA), 139.36, 139.33, 139.31, 139.14, 139.03, 138.95, 138.93, 138.87, 138.69, 138.65, 138.5, 138.3, 138.2, 138.1, 138.0, 137.83, 137.81, 136.9, 133.5, 133.4, 133.2, 132.4, 130.6, 130.1, 129.95, 129.92, 129.8, 129.7, 129.0, 128.77, 128.71, 128.68, 128.65, 128.63, 128.58, 128.55, 128.52, 128.45, 128.43, 128.41, 128.3, 128.27, 128.24, 128.22, 128.18, 128.16, 128.12, 128.07, 128.03, 128.00, 127.98, 127.93, 127.83, 127.81, 127.76, 127.71, 127.66, 127.64, 127.57, 127.50, 127.4, 127.3, 127.2, 126.3, 101.07 (**C-1**, J_{C-H} = 165 Hz), 101.01 (**C-1**, J_{C-H} = 163 Hz), 100.5 (**C-1**, J_{C-H} = 167 Hz), 100.4 (**C-1**, J_{C-H} = 165 Hz), 100.3 (**C-1**, J_{C-H} = 163 Hz), 99.7 (**C-1**, J_{C-H} = 169 Hz), 98.0 (**2 x C-1**, J_{C-H} = 176 Hz, J_{C-H} = 171 Hz), 97.2 (**C-1**, J_{C-H} = 171

Hz), 92.1 (CCl_3), 91.8 (CCl_3), 84.0, 80.1, 79.43, 79.41, 79.3, 79.0, 78.9, 78.46, 78.43, 76.7, 76.04, 76.02, 75.86, 75.82, 75.6, 75.2, 75.08, 75.05, 75.02, 74.9, 74.7, 73.86, 73.81, 73.7, 73.58, 73.55, 73.49, 73.43, 73.3, 73.18, 73.11, 73.07, 73.05, 72.8, 72.70, 72.66, 72.61, 72.2, 72.1, 71.3, 69.8, 68.4, 68.2, 67.86, 67.82, 67.75, 67.5, 67.0, 66.78, 66.7, 66.6, 61.2, 59.0, 40.9 (CH_2-NHCbz), 29.5 (CH_2 pentane), 29.0 (CH_2 pentane), 23.2 (CH_2 pentane), 16.7 (CH_3 Fuc), 16.44 (CH_3 Fuc), 16.37 (CH_3 Fuc). HRMS (ESI) calc. for $C_{232}H_{239}Cl_6N_3O_{49}Na_2$, 2053.2109; found: 2053.2148.

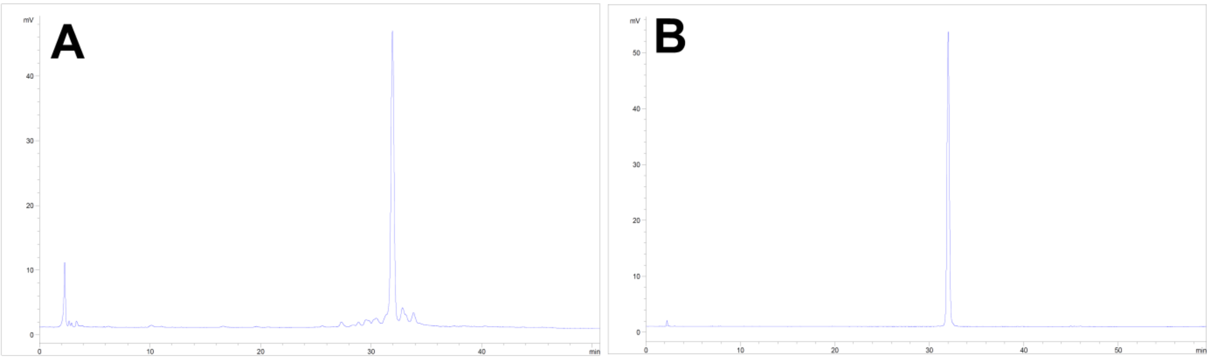

*Analytical NP-HPLC of (**A**) reaction crude after photocleavage and (**B**) the purified product (detection: ELSD).*

***N*-Benzyloxycarbonyl-5-amino-pentyl 2,4,6-tri-*O*-benzyl-β-D-galactopyranosyl-(1→3)-6-*O*-benzyl-2-deoxy-2-*N*-trichloroacetyl-β-D-glucopyranosyl-(1→3)-2-*O*-benzoyl-4,6-di-*O*-benzyl-β-D-galactopyranosyl-(1→4)-2,3-di-*O*-benzoyl-6-*O*-benzyl-β-D-glucopyranoside (27)**

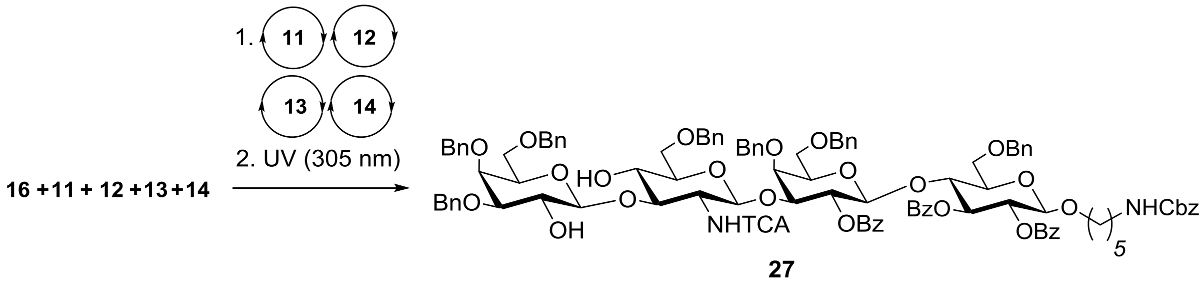

Yield: 47%, 11.5 mg

^{1}H NMR (600 MHz, $CDCl_3$) δ 7.93 – 7.87 (m, 4H), 7.84 (d, *J* = 8.1 Hz, 2H), 7.55 (t, *J* = 7.4 Hz, 1H), 7.45 (t, *J* = 7.4 Hz, 1H), 7.41 (t, *J* = 7.6 Hz, 2H), 7.37 – 7.23 (m, 39H), 7.20 – 7.12 (m, 6H), 6.60 (d, *J* = 7.6 Hz, 1H, NHTCA), 5.54 (t, *J* = 9.4 Hz, 1H), 5.39 – 5.35 (m, 1H), 5.33 – 5.28 (m, 1H), 5.06 (s, 2H), 4.86 (d, *J* = 11.7 Hz, 1H), 4.81 (d, *J* = 11.9 Hz, 1H), 4.70 – 4.63 (m, 3H, including **H-1**), 4.55 – 4.48 (m, 5H, including **H-1**), 4.43 – 4.35 (m, 5H, including **H-1**),

4.24 (d, J = 12.3 Hz, 1H), 4.11 – 4.03 (m, 4H, including **H-1**), 3.93 – 3.83 (m, 4H), 3.79 (dt, J = 10.6, 5.8 Hz, 1H), 3.73 (d, J = 2.5 Hz, 1H), 3.62 – 3.57 (m, 2H), 3.56 – 3.32 (m, 11H), 3.28 (dd, J = 9.7, 2.5 Hz, 1H), 2.94 – 2.87 (m, 3H), 2.84 (t, J = 8.7 Hz, 1H), 1.45 (m, 2H, CH_2 pentane), 1.30 – 1.28 (m, 2H, CH_2 pentane), 1.20 – 1.13 (m, 2H, CH_2 pentane). ^{13}C NMR (151 MHz, $CDCl_3$) δ 165.3 (2x C=O Bz), 164.9 (C=O Bz), 162.7 (C=O Cbz), 156.4 (C=O TCA), 139.2, 138.3, 138.24, 138.23, 138.15, 138.13, 137.56, 133.52, 133.2, 132.6, 130.6, 129.99, 129.97, 129.84, 129.75, 129.73, 128.8, 128.63, 128.59, 128.55, 128.51, 128.49, 128.46, 128.42, 128.20, 128.18, 128.16, 128.13, 128.11, 128.04, 128.01, 127.94, 127.92, 127.91, 127.84, 127.79, 127.77, 127.72, 127.70, 127.3, 104.3 (**C-1**, $J_{C\text{-}H}$ = 165 Hz), 101.1 (**C-1**, $J_{C\text{-}H}$ = 163 Hz), 100.8 (**C-1**, $J_{C\text{-}H}$ = 162 Hz), 99.4 (**C-1**, $J_{C\text{-}H}$ = 165 Hz), 92.3 ($\mathit{C}Cl_3$), 84.9, 81.2, 78.0, 76.1, 75.7, 75.06, 74.95, 74.75, 74.72, 74.3, 73.8, 73.7, 73.57, 73.52, 73.2, 73.1, 72.8, 72.2, 71.3, 69.9, 69.80, 69.75, 68.9, 67.6, 67.3, 66.6, 57.7, 40.9 ($\mathit{C}H_2$-NHCbz), 29.5 ($\mathit{C}H_2$ pentane), 29.0 ($\mathit{C}H_2$ pentane), 23.2 ($\mathit{C}H_2$ pentane). HRMS (ESI) calc. for $C_{109}H_{113}Cl_3N_2O_{26}Na$, 1993.6539; found: 1993.6573.

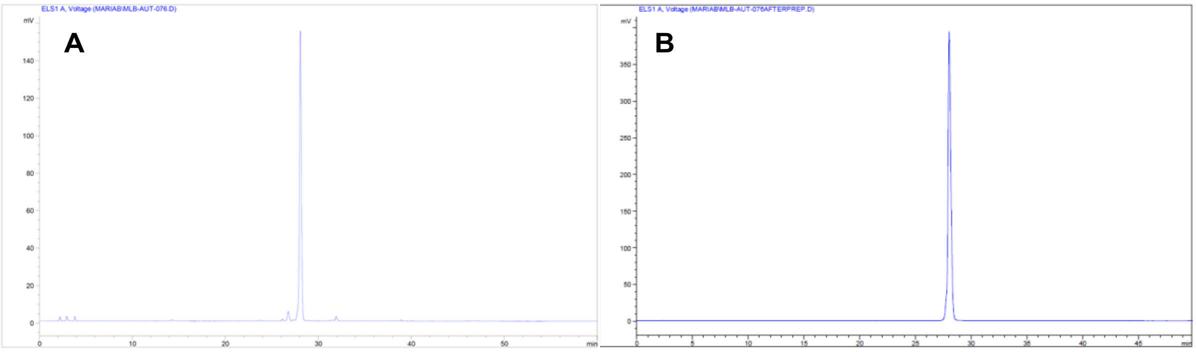

*Analytical NP-HPLC of (**A**) reaction crude after photocleavage and (**B**) the purified product (detection: ELSD).*

_N_-Benzyloxycarbonyl-5-amino-pentyl 2,3,4-tri-_O_-benzyl-α-L-fucopyranosyl-(1→2)-3,4,6-tri-_O_-benzyl-β-D-galactopyranosyl-(1→3)-6-_O_-benzyl-4-_O_-(2,3,4-tri-_O_-benzyl-α-L-fucopyranosyl)-2-deoxy-2-_N_-trichloroacetyl-β-D-glucopyranosyl-(1→3)-2-_O_-benzoyl-4,6-di-_O_-benzyl-β-D-galactopyranosyl-(1→4)-2,3-di-_O_-benzoyl-6-_O_-benzyl-β-D-glucopryanoside (28)

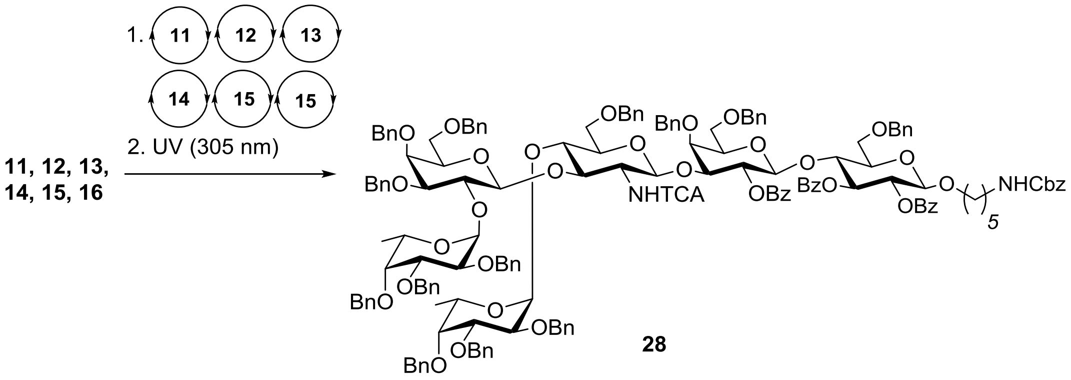

28

Yield: 34%, 12.1 mg

^{1}H NMR (600 MHz, $CDCl_3$) δ 7.92 – 7.86 (m, 4H), 7.80 (d, *J* = 8.2 Hz, 2H), 7.51 (t, *J* = 7.4 Hz, 1H), 7.45 (t, *J* = 7.4 Hz, 1H), 7.40 – 7.07 (m, 75H), 7.01 (dd, *J* = 6.4, 2.8 Hz, 2H), 6.53 (d, *J* = 9.4 Hz, 1H, NHTCA), 5.54 – 5.49 (m, 2H, including **H-1**), 5.35 (dd, *J* = 10.1, 7.8 Hz, 1H), 5.30 (dd, *J* = 9.8, 7.9 Hz, 1H), 5.06 (s, 2H), 4.99 – 4.94 (m, 2H, including **H-1**), 4.82 – 4.74 (m, 4H), 4.72 (d, *J* = 10.9 Hz, 1H), 4.65 (d, *J* = 11.9 Hz, 1H), 4.58 – 4.52 (m, 4H, including **H-1**), 4.50 – 4.46 (m, 4H, including **H-1**), 4.45 – 4.29 (m, 13H, including 2 x **H-1**), 4.24 (d, *J* = 12.3 Hz, 2H), 4.07 – 4.02 (m, 3H), 4.01 – 3.89 (m, 6H), 3.87 – 3.83 (m, 3H), 3.81 – 3.76 (m, 4H), 3.69 – 3.59 (m, 3H), 3.54 (dd, *J* = 8.7, 5.5 Hz, 1H), 3.49 (dd, *J* = 11.2, 3.9 Hz, 1H), 3.41 – 3.32 (m, 6H), 3.15 (s, 1H), 2.93 – 2.88 (m, 3H), 2.83 (t, *J* = 8.6 Hz, 1H), 1.50 – 1.40 (m, 2H, CH_2 pentane), 1.33 – 1.29 (m, 2H, CH_2 pentane), 1.20 – 1.13 (m, 8H, CH_2 pentane, 2x CH_3 Fuc). ^{13}C NMR (151 MHz, $CDCl_3$) δ 165.4 (C=O Bz), 165.3 (C=O Bz), 164.3 (C=O Bz), 161.2 (C=O Cbz), 156.4 (C=O TCA), 139.6, 139.5, 139.4, 139.3, 139.2, 138.87, 138.82, 138.6, 138.33, 138.27, 138.15, 138.12, 137.9, 136.9, 133.3, 133.2, 132.5, 130.6, 129.97, 129.96, 129.93, 129.8, 129.7, 128.84, 128.75, 128.73, 128.6, 128.54, 128.51, 128.48, 128.46, 128.45, 128.31, 128.29, 128.24, 128.18, 128.14, 128.12, 128.10, 128.07, 128.04, 127.91, 127.87, 127.83, 127.75, 127.72, 127.70, 127.66, 127.60, 127.54, 127.51, 127.32, 127.28, 127.24, 127.23, 127.12, 127.06, 101.3 (**C-1**, $J_{C\text{-}H}$ = 162 Hz), 101.1 (**C-1**, $J_{C\text{-}H}$ = 161 Hz), 100.8 (**C-1**, $J_{C\text{-}H}$ = 163 Hz), 100.5 (**C-1**, $J_{C\text{-}H}$ = 164 Hz), 99.0 (**C-1**, $J_{C\text{-}H}$ = 175 Hz), 98.0 (**C-1**, $J_{C\text{-}H}$ = 172 Hz), 92.5 (CCl_3), 83.4, 80.4, 79.6, 78.4, 78.2, 78.0, 76.1, 76.0, 75.67, 75.65, 75.57, 75.00, 74.98, 74.94, 74.86, 74.79, 74.6, 74.5, 74.0, 73.6, 73.47, 73.44, 73.24, 73.22, 73.16, 73.0, 72.8, 72.4, 72.1, 71.9, 71.7, 69.8, 68.7, 67.7, 67.43, 67.40, 67.2, 66.7, 66.6, 58.9, 40.9 (CH_2-NHCbz), 29.5 (CH_2 pentane), 29.0 (CH_2 pentane), 23.2 (CH_2 pentane), 16.4 (2 x CH_3 Fuc). HRMS (ESI) calc. for $C_{163}H_{169}Cl_3N_2O_{34}Na_2$, 1424.5203; found: 1424.5269.

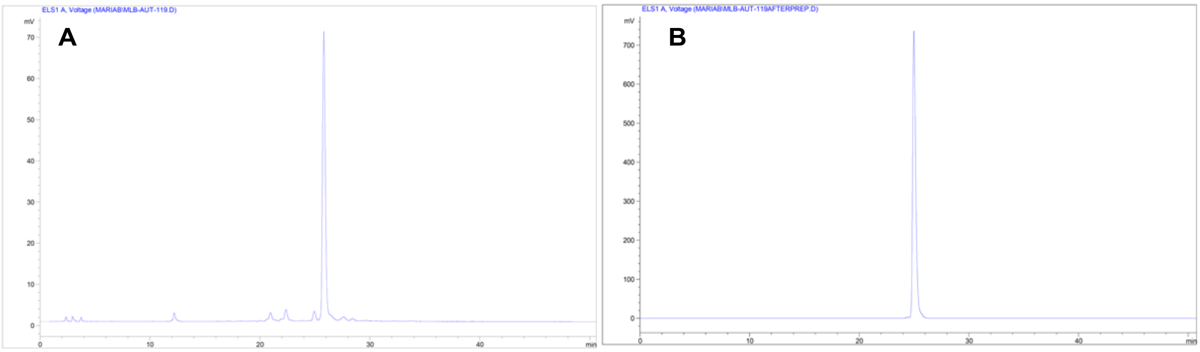

*Analytical NP-HPLC of (**A**) reaction crude after photocleavage and (**B**) the purified product (detection: ELSD).*

_N_-Benzyloxycarbonyl-5-amino-pentyl 2-_O_-benzoyl-3,4,6-tri-_O_-benzyl-β-D-galactopyranosyl-(1→3)-4-_O_-(2,3,4-tri-_O_-benzyl-α-L-fucopyranosyl) -6-_O_-benzyl -_N_-trichloroacetyl-2-deoxy-β-D-glucopyranosyl- (1→3)-2-_O_-benzoyl-4,6-di-_O_-benzyl-β-D-galactopyranosyl-(1→4)-2,3-di-_O_-benzyl- β-D-glucopyranoside (29)

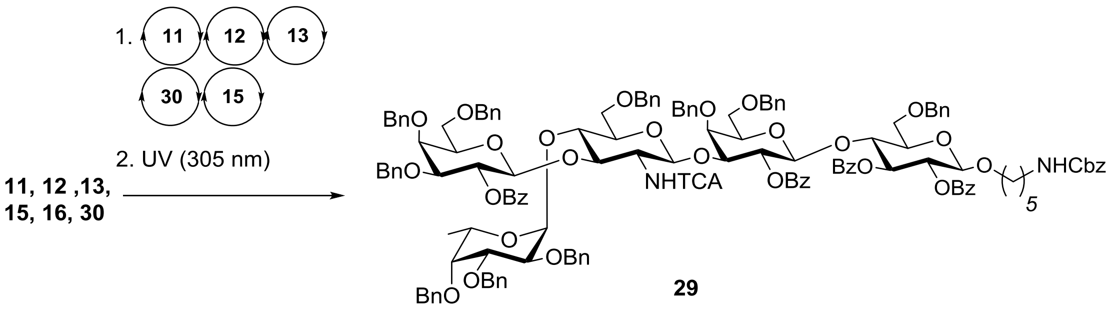

Yield: 47%, 14.5 mg

^{1}H NMR (600 MHz, $CDCl_3$) δ 7.99 (d, *J* = 7.2 Hz, 2H), 7.92 – 7.88 (m, 2H), 7.85 – 7.79 (m, 4H), 7.55 – 7.50 (m, 2H), 7.45 (t, *J* = 7.4 Hz, 1H), 7.41 – 7.07 (m, 59H), 7.05 – 6.99 (m, 5H), 6.31 (d, *J* = 6.8 Hz, 1H), 5.54 – 5.48 (m, 2H), 5.29 (dd, *J* = 9.8, 8.0 Hz, 1H), 5.22 (dd, *J* = 10.2, 7.9 Hz, 1H), 5.06 (s, 2H), 4.96 (d, *J* = 10.6 Hz, 1H), 4.88 (d, *J* = 3.8 Hz, 1H, **H-1**), 4.79 – 4.73 (m, 2H, including **H-1**), 4.65 – 4.50 (m, 7H), 4.49 – 4.45 (m, 3H, including 2 x **H-1**), 4.44 – 4.38 (m, 4H), 4.26 – 4.14 (m, 5H, including **H-1**), 4.08 – 3.96 (m, 6H), 3.83 (d, *J* = 2.8 Hz, 1H), 3.82 – 3.75 (m, 3H), 3.70 – 3.56 (m, 5H), 3.44 – 3.24 (m, 9H), 2.94 – 2.87 (m, 3H), 2.72 (t, *J* = 8.7 Hz, 1H), 2.49 – 2.40 (m, 1H), 1.51 – 1.39 (m, 4H, 2x CH_2 pentane), 1.30 (d, *J* = 6.4 Hz, 3H, CH_3 Fuc), 1.19 – 1.10 (m, 2H, CH_2 pentane). ^{13}C NMR (151 MHz, $CDCl_3$) δ 165.4 (C=O Bz), 165.3 (C=O Bz), 165.2 (C=O Bz), 164.6 (C=O Bz), 161.0 (C=O Cbz), 156.4 (C=O TCA), 139.5, 139.3, 138.91, 138.87, 138.5, 138.16, 138.07, 138.01, 137.8, 137.3, 136.9, 133.5, 133.16, 133.13, 132.4, 130.7, 130.2, 130.03, 129.96, 129.91, 129.8, 129.7, 129.1, 128.9, 128.64, 128.62, 128.54, 128.50, 128.45, 128.44, 128.43, 128.27, 128.22,

128.17, 128.16, 128.12, 128.0, 127.89, 127.87, 127.84, 127.76, 127.73, 127.69, 127.65, 127.4, 127.31, 127.26, 101.1 (**C-1**, $J_{C\text{-}H}$ = 165 Hz), 100.9 (**C-1**, $J_{C\text{-}H}$ = 162 Hz), 100.5 (**C-1**, $J_{C\text{-}H}$ = 164 Hz), 98.2 (**C-1**, $J_{C\text{-}H}$ = 169 Hz), 97.7 (**C-1**, $J_{C\text{-}H}$ = 173 Hz), 92.2 (*C*Cl_3), 80.4, 79.2, 78.4, 77.5, 75.7, 75.4, 75.2, 74.9, 74.8, 74.73, 74.67, 74.59, 73.8, 73.48, 73.43, 73.26, 73.20, 73.1, 72.9, 72.3, 72.09, 72.08, 71.9, 71.6, 71.5, 69.7, 67.72, 67.68, 67.4, 67.3, 66.9, 66.6, 61.0, 40.9 (*C*H_2-NHCbz), 29.0 (2x *C*H_2 pentane), 23.2 (*C*H_2 pentane), 16.6 (*C*H_3 Fuc). HRMS (ESI) calc. for $C_{143}H_{145}Cl_3N_2O_{31}Na$, 2513.8789; found: 2513.8787.

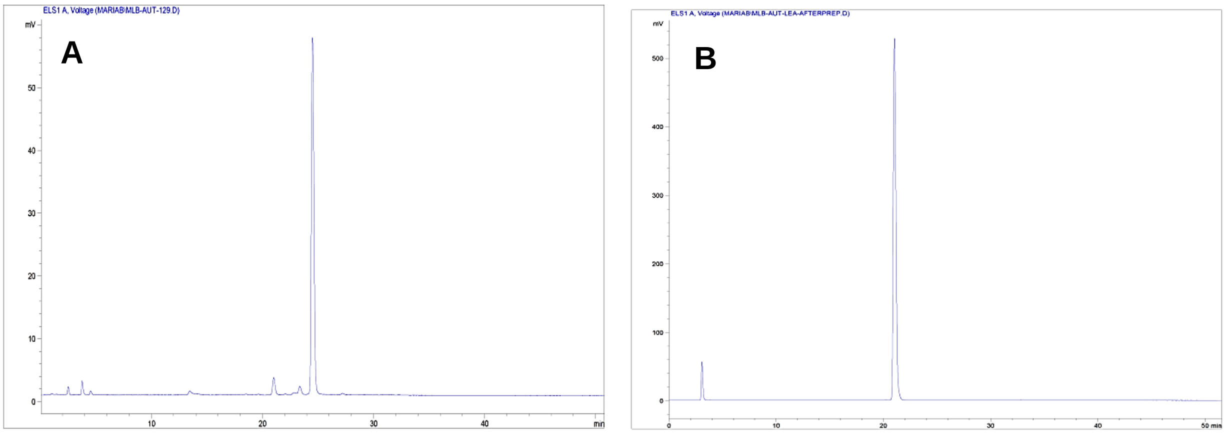

*Analytical NP-HPLC of (**A**) reaction crude after photocleavage and (**B**) the purified product (detection: ELSD).*

Deprotection of Oligosaccharides 31-40

Global Deprotection: Methanolysis - Hydrogenolysis

AGA-synthesized, photocleaved, NP-HPLC purified product was sequentially subjected to methanolysis (when containing ester protecting groups) and hydrogenolysis. Hydrogenolysis product was purified by RP-HPLC to afford the final deprotected compound.

1) Methanolysis

The protected oligosaccharide was coevaporated twice with toluene and kept under vacuum. Subsequently, it was dissolved in DCM (0.50 mL) under N_2 atmosphere and MeOH (0.50 mL) was added. NaOMe in MeOH 0.5 M (0.735 mL) was added and the solution was stirred until reaction completion (~48h, monitored by MALDI). Amberlite resin was added and when the

pH of the solution was neutral it was filtered. Solvents were removed *in vacuo* to obtain the partially deprotected oligosaccharide.

2) Hydrogenolysis

The methanolysis reaction crude was dissolved in AcOEt/t-BuOH/H_2O 2:1:1 (2 mL) and Pd/C was added. The mixture was degassed using N_2, purged with H_2 and kept under H_2 atmosphere until all TCA, Cbz and Bn protecting groups were removed (monitored by ^1H NMR, MALDI and/or LC-MS). TEA (5.00 μL) was added, the reaction mixture was filtrated through a PTFE syringe filter, and washed with MeOH (0.50 mL) and H_2O (0.5 mL). Solvents were removed *in vacuo*. The concentrated was lyophilized.

3) Purification

The lyophilized reaction crude was dissolved in water and purified by RP-HPLC. A Thermo-Scientific Hypercarb column (150 mm x 4.60 mm I.D.) was used for analytical RP-HPLC with a flow rate of 0.70 mL/min and 0.1 % HCO_2H in H_2O/ACN as eluents. A Thermo-Scientific Hypercarb column (150 mm x 10.0 mm I.D.) was used for preparative RP-HPLC, with a flow rate of 3.50 mL/min and 0.1 % HCO_2H in H_2O/ACN as eluents. Unless stated otherwise, the gradient program detailed hereafter was used:

1. Isocratic 0.1 % HCO_2H in H_2O (5 min).
2. Linear gradient 0 to 30% ACN in 0.1 % HCO_2H in H_2O (30 min).
3. Linear gradient to 100% ACN (5 min).

The product was lyophilized and isolated as its formate salt.

Experimental Data for Deprotected Oligosaccharides 31-38

5-Amino-pentyl α-L-fucopyranosyl-(1→2)-β-D-galactopyranosyl-(1→4)-2-*N*-acetyl-2-deoxy-β-D-glucopyranoside (31)

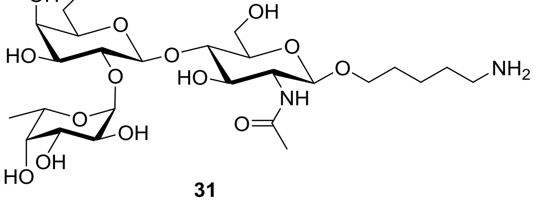

31

Compound **19** (8.33 mg, 5.62 μmol) was subjected to hydrogenolysis (see above *global deprotection* method). After RP-HPLC purification, conjugation-ready compound **34** was isolated as its formate salt (38%, 1.42 mg). ^{1}H NMR (600 MHz, D_2O) δ 8.47 (s, 1H, *H*COO$^-$), 5.32 (d, *J* = 3.2 Hz, 1H, **H-1**), 4.55 (d, *J* = 7.7 Hz, 1H, **H-1**), 4.51 (d, *J* = 8.4 Hz, 1H, **H-1**), 4.24 (q, *J* = 6.6 Hz, 1H, H-5 Fuc), 4.00 (dd, *J* = 12.0, 2.0 Hz, 1H), 3.96 – 3.87 (m, 3H), 3.84 – 3.78 (m, 6H), 3.77 – 3.66 (m, 5H), 3.61 (dt, *J* = 10.2, 6.3 Hz, 1H), 3.49 – 3.45 (m, 1H), 3.03 – 2.98 (m, 2H, CH_2-NH_3^+), 2.05 (s, 3H, CH_3 NHAc), 1.69 (p, *J* = 7.7 Hz, 2H, CH_2 pentane), 1.62 (p, *J* = 6.5 Hz, 2H, CH_2 pentane), 1.46 – 1.38 (m, 2H, CH_2 pentane), 1.25 (d, *J* = 6.6 Hz, 3H, CH_3 Fuc). ^{13}C NMR (151 MHz, D_2O) δ 174.4 (H*C*OO$^-$), 171.0 (C=O NHAc), 101.1 (**C-1**, $J_{C\text{-}H}$ = 166 Hz), 100.2 (**C-1**, $J_{C\text{-}H}$ = 165 Hz), 99.4 (**C-1**, $J_{C\text{-}H}$ = 175 Hz), 76.4, 76.1, 75.24, 75.19, 73.5, 72.3, 71.6, 70.1, 69.6, 69.0, 68.1, 66.8 (C-5 Fuc), 61.0, 60.1, 55.2, 39.3 (*C*H_2-NH_3^+), 28.0 (*C*H_2 pentane), 26.3 (*C*H_2 pentane), 22.10 (*C*H_3 NHAc), 22.06 (*C*H_2 pentane), 15.2. HRMS (ESI) calc. for $C_{25}H_{47}N_2O_{15}$, 615.2971; found: 615.2976.

5-Amino-pentyl β-D-galactopyranosyl-(1→4)-2-*N*-acetyl-2-deoxy-3-*O*-(α-L-fucopyranosyl)-β-D-glucopyranoside (32)

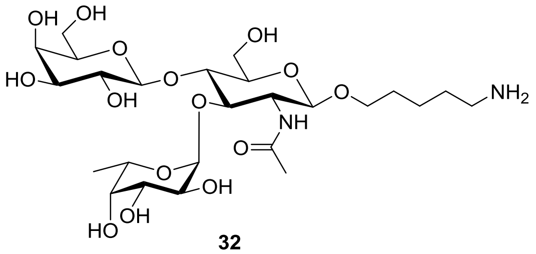

32

Compound **20** (8.89 mg, 6.00 μmol) was subjected to hydrogenolysis (see above *global deprotection* method). After RP-HPLC purification, conjugation-ready compound **35** was isolated as its formate salt (54%, 2.12 mg). ^{1}H NMR (600 MHz, D_2O) δ 8.50 (s, 1H, *H*COO$^-$), 5.12 (d, J = 4.1 Hz, 1H, **H-1** Fuc), 4.87 – 4.82 (m, 1H, H-5 Fuc), 4.54 (d, J = 8.1 Hz, 1H, **H-1**), 4.47 (d, J = 7.8 Hz, 1H, **H-1**), 4.02 c(dd, J = 12.3, 2.3 Hz, 1H), 3.97 – 3.84 (m, 7H), 3.81 (d, J = 3.3 Hz, 1H), 3.78 – 3.65 (m, 4H), 3.63 – 3.58 (m, 3H), 3.51 (dd, J = 9.9, 7.8 Hz, 1H), 3.03 – 2.98 (m, 2H, CH_2-NH_3^+), 2.04 (s, 3H, CH_3 NHAc), 1.69 (p, J = 7.7 Hz, 2H, CH_2 pentane), 1.61 (p, J = 6.6 Hz, 2H, CH_2 pentane), 1.45 – 1.37 (m, 2H, CH_2 pentane), 1.19 (d, J = 6.6 Hz, 3H, CH_3 Fuc). ^{13}C NMR (151 MHz, D_2O) δ 174.1 (H*C*OO$^-$), 170.9 (C=O NHAc), 101.8 (**C-1**, $J_{C\text{-}H}$ = 165 Hz), 100.9 (**C-1**, $J_{C\text{-}H}$ = 166 Hz), 98.6 (**C-1**, $J_{C\text{-}H}$ = 174 Hz), 75.3, 74.9, 73.3, 72.4, 71.8, 71.0, 70.1, 69.1, 68.3, 67.6, 66.7 (C-5 Fuc), 61.4, 59.7, 55.8, 39.3 (*C*H_2-NH_3^+), 28.0 (*C*H_2 pentane), 26.3 (*C*H_2 pentane), 22.15 (*C*H_3 NHAc), 22.06 (*C*H_2 pentane), 15.2 (*C*H_3 Fuc). HRMS (ESI) calc. for $C_{25}H_{47}N_2O_{15}$, 615.2971; found: 615.2974.

5-Amino-pentyl α-L-fucopyranosyl-(1→2)-β-D-galactopyranosyl-(1→4)-2-*N*-acetyl-2-deoxy-3-*O*-(α-L-fucopyranosyl)-β-D-glucopyranosyl-(1→3)-β-D-galactopyranoside (33)

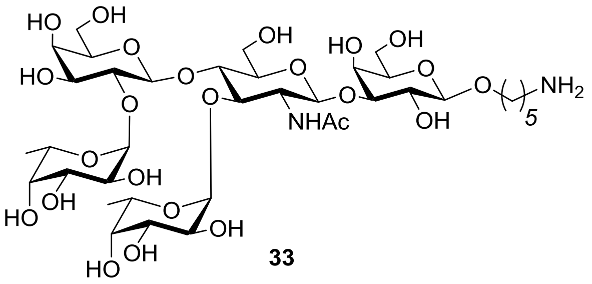

33

Compound **18** (14.4 mg, 6.10 µmol) was subjected to hydrogenolysis (see above *global deprotection* method). After RP-HPLC purification, conjugation-ready compound **33** was isolated as its formate salt (35%, 2.0 mg). ^{1}H NMR (700 MHz, D_2O) δ 8.46 (s, 1H, $HCOO^-$), 5.29 (d, *J* = 3.8 Hz, 1H, **H-1** Fuc), 5.12 (d, *J* = 4.0 Hz, 1H, **H-1** Fuc), 4.88 (q, *J* = 7.1 Hz, 1H, H-5 Fuc), 4.73 (d, *J* = 9.0 Hz, 1H, **H-1**), 4.52 (d, *J* = 7.8 Hz, 1H, **H-1**), 4.39 (d, *J* = 8.0 Hz, 1H, **H-1**), 4.26 (q, *J* = 6.6 Hz, 1H, H-5 Fuc), 4.17 – 4.13 (m, 1H), 4.01 (d, *J* = 10.3 Hz, 1H), 3.98 – 3.49 (m, 31H), 3.46 (dd, *J* = 9.7, 5.0 Hz, 1H), 3.01 (t, *J* = 7.5 Hz, 2H, CH_2-NH_3^+), 2.03 (s, 3H, CH_3 NHAc), 1.74 – 1.64 (m, 4H, 2 x CH_2 pentane), 1.47 (p, *J* = 7.7 Hz, 2H, CH_2 pentane), 1.27 (d, *J* = 6.6 Hz, 3H, CH_3 Fuc), 1.24 (d, *J* = 6.6 Hz, 3H, CH_3 Fuc). ^{13}C NMR (176 MHz, D_2O) δ 174.8 (C=O NHAc), 171.0 ($HCOO^-$), 102.8 (**C-1**, J_{C-H} = 161 Hz), 102.4 (**C-1**, J_{C-H} = 163 Hz), 100.2 (**C-1**, J_{C-H} = 166 Hz), 99.5 (**C-1**, J_{C-H} = 178 Hz), 98.6 (**C-1**, J_{C-H} = 176 Hz), 82.5, 76.4, 75.4, 74.9, 74.7, 74.6, 73.6, 73.1, 71.9, 71.7, 70.0, 69.8, 69.2, 68.8, 68.29, 68.26, 67.7, 66.9, 66.8, 62.5, 61.5, 60.9, 59.8, 56.2, 39.4 (CH_2-NH_3^+), 28.2 (CH_2 pentane), 26.4 (CH_2 pentane), 22.3 (CH_3 NHAc), 22.1 (CH_2 pentane), 15.46 (CH_3 Fuc), 15.45 (CH_3 Fuc). HRMS (ESI) calc. for $C_{37}H_{67}N_2O_{24}$, 923.4078; found: 923.4081.

5-Amino-pentyl α-L-fucopyranosyl-(1→2)-β-D-galactopyranosyl-(1→4)-2-*N*-acetyl-2-deoxy-β-D-glucopyranosyl-(1→3)-β-D-galactopyranosyl-(1→4)-β-D-glucopyranoside (34)

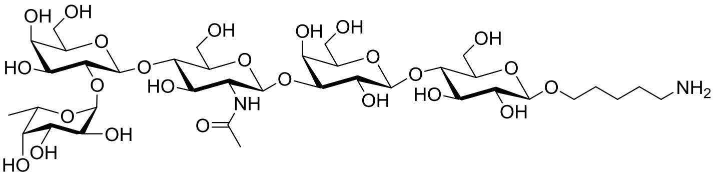

34

Compound **21** (13.0 mg, 5.40 μmol) was subjected to hydrogenolysis (see above *global deprotection* method). After RP-HPLC purification, conjugation-ready compound **34** was isolated as its formate salt (22%, 1.2 mg). ^{1}H NMR (700 MHz, D_2O) δ 8.46 (s, 1H, *H*COO$^-$), 5.33 – 5.31 (d, *J* = 1.4 Hz, 1H, **H-1** Fuc), 4.71 (d, *J* = 8.3 Hz, 1H, **H-1**), 4.56 (d, *J* = 7.7 Hz, 1H, **H-1**), 4.49 (d, *J* = 8.0 Hz, 1H, **H-1**), 4.45 (d, *J* = 7.8 Hz, 1H, **H-1**), 4.23 (q, *J* = 6.5 Hz, 1H), 4.15 (s, 1H), 4.00 – 3.93 (m, 4H), 3.91 – 3.86 (m, 2H), 3.85 – 3.57 (m, 20H), 3.50 – 3.45 (m, 1H), 3.31 (t, *J* = 8.2 Hz, 1H), 3.02 (t, *J* = 7.5 Hz, 2H, CH_2-NH_3^+), 2.05 (s, 3H, CH_3 NHAc), 1.74 – 1.65 (m, 4H, 2 x CH_2 pentane), 1.47 (p, *J* = 7.7 Hz, 2H, CH_2 pentane), 1.24 (d, *J* = 6.5 Hz, 3H, CH_3 Fuc). ^{13}C NMR (176 MHz, D_2O) δ 174.9 (H*C*OO$^-$), 171.0 (C=O NHAc), 102.9 (**C-1**, J_{C-H} = 160 Hz), 102.8 (**C-1**, J_{C-H} = 162 Hz), 102.0 (**C-1**, J_{C-H} = 159 Hz), 100.3 (**C-1**, J_{C-H} = 167 Hz), 99.4 (**C-1**, J_{C-H} = 177 Hz), 82.0, 78.4, 76.5, 75.9, 75.3, 75.1, 74.9, 74.8, 74.5, 73.5, 72.8, 72.1, 71.7, 70.1, 70.0, 69.6, 69.1, 68.3, 68.2, 67.0, 61.1, 60.9, 60.1, 60.0, 55.4, 39.4 (*C*H_2-NH_3^+), 28.2 (*C*H_2 pentane), 26.4 (*C*H_2 pentane), 22.2 (*C*H_3 NHAc), 22.1 (*C*H_2 pentane), 15.3 (*C*H_3 Fuc). HRMS (ESI) calc. for $C_{37}H_{67}N_2O_{25}$, 939.4027; found: 939.4030.

5-Amino-pentyl β-D-galactopyranosyl-(1→4)-2-*N*-acetyl-2-deoxy-3-*O*-(α-L-fucopyranosyl)-β-D-glucopyranosyl-(1→3)-β-D-galactopyranosyl-(1→4)-β-D-glucopyranoside (35)

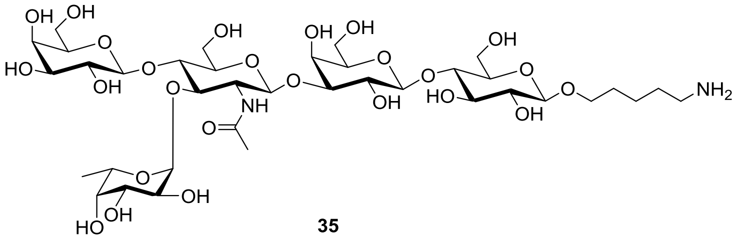

35

Compound **22** (28.3 mg, 11.8 μmol) was subjected to methanolysis followed by hydrogenolysis (see above *global deprotection* method). After RP-HPLC purification, conjugation-ready compound **35** was isolated as its formate salt (35%, 4.21 mg). ^{1}H NMR (600 MHz, D_2O) δ 8.47 (s, 1H, *H*COO$^-$), 5.14 (d, *J* = 4.1 Hz, 1H, **H-1**), 4.85 (q, *J* = 6.8 Hz, 1H, H-5 Fuc), 4.73 (d, *J* = 8.6 Hz, 1H, **H-1**), 4.52 – 4.43 (m, 3H, 3x **H-1**), 4.17 (d, *J* = 3.3 Hz, 1H), 4.01 – 3.86 (m, 9H), 3.82 – 3.69 (m, 10H), 3.69 – 3.58 (m, 7H), 3.51 (dd, *J* = 9.8, 7.9 Hz, 1H), 3.32 (t, *J* = 8.6 Hz, 1H), 3.05 – 3.01 (m, 2H, CH_2-NH_3^+), 2.04 (s, 3H, CH_3 NHAc), 1.75 – 1.66 (m, 4H, 2x CH_2 pentane), 1.47 (p, *J* = 7.7 Hz, 2H, CH_2 pentane), 1.19 (d, *J* = 6.6 Hz, 3H, CH_3 Fuc). ^{13}C NMR (151 MHz, D_2O) δ 174.6 (H*C*OO$^-$), 171.0 (C=O NHAc), 102.9 (**C-1**, J_{C-H} = 167 Hz), 102.5 (**C-1**, J_{C-H} = 165 Hz), 101.9 (**C-1**, J_{C-H} = 164 Hz), 101.7 (**C-1**, J_{C-H} = 164 Hz), 98.5 (**C-1**, J_{C-H} = 173 Hz), 82.0, 78.3, 75.1, 74.9, 74.8, 74.71, 74.67, 74.4, 73.0, 72.7, 72.4, 71.8, 71.0, 70.0, 69.9, 69.1, 68.3, 68.2, 67.6, 66.6 (C-5 Fuc), 61.4, 60.9, 60.0, 59.6, 55.9, 39.3

(CH_2-NH_3^+), 28.1 (CH_2 pentane), 26.3 (CH_2 pentane), 22.2 (CH_3 NHAc), 22.0 (CH_2 pentane), 15.2 (CH_3 Fuc). HRMS (ESI) calc. for $C_{37}H_{67}N_2O_{25}$, 939.4027; found: 939.4024.

5-Amino-pentyl α-L-fucopyranosyl-(1→2)-β-D-galactopyranosyl-(1→4)-2-*N*-acetyl-2-deoxy-3-*O*-(α-L-fucopyranosyl)-β-D-glucopyranosyl-(1→3)-β-D-galactopyranosyl-(1→4)-β-D-glucopyranoside (36)

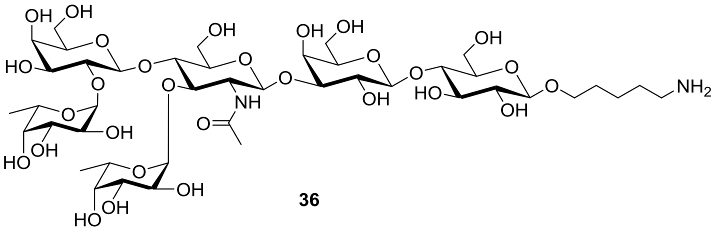

36

Compound **23** (31.7 mg, 11.3 μmol) was subjected to methanolysis followed by hydrogenolysis (see above *global deprotection* method). After RP-HPLC purification, conjugation-ready compound **36** was isolated as its formate salt (17%, 2.2 mg). ^{1}H NMR (600 MHz, D_2O) δ 8.48 (s, 1H, $HCOO^-$), 5.29 (d, J = 3.4 Hz, 1H, **H-1** Fuc), 5.13 (d, J = 4.1 Hz, 1H, **H-1** Fuc), 4.89 (q, J = 6.8 Hz, 1H, H-5 Fuc), 4.73 (d, J = 8.4 Hz, 1H, **H-1**), 4.53 (d, J = 7.8 Hz, 1H, **H-1**), 4.50 (d, J = 8.0 Hz, 1H, **H-1**), 4.45 (d, J = 7.9 Hz, 1H, **H-1**), 4.27 (q, J = 6.7 Hz, 1H, H-5 Fuc), 4.16 (d, J = 3.2 Hz, 1H), 4.03 – 3.57 (m, 29H), 3.47 (dd, J = 9.2, 4.2 Hz, 1H), 3.32 (t, J = 8.5 Hz, 1H), 3.02 (t, J = 7.5 Hz, 2H, CH_2-NH_3^+), 2.04 (s, 3H, CH_3 NHAc), 1.75 – 1.65 (m, 4H, 2 x CH_2 pentane), 1.47 (p, J = 7.7 Hz, 2H, CH_2 pentane), 1.28 (d, J = 6.6 Hz, 3H, CH_3 Fuc), 1.25 (d, J = 6.6 Hz, 3H, CH_3 Fuc). ^{13}C NMR (151 MHz, D_2O) δ 174.6 ($HCOO^-$), 170.9 (C=O NHAc), 102.9 (**C-1**, $J_{C\text{-}H}$ = 166 Hz), 102.4 (**C-1**, $J_{C\text{-}H}$ = 168 Hz), 101.9 (**C-1**, $J_{C\text{-}H}$ = 163 Hz), 100.1 (**C-1**, $J_{C\text{-}H}$ = 166 Hz), 99.4 (**C-1**, $J_{C\text{-}H}$ = 175 Hz), 98.5 (**C-1**, $J_{C\text{-}H}$ = 173 Hz), 82.0, 78.3, 76.3, 75.3, 74.79, 74.71, 74.68, 74.4, 73.5, 73.0, 72.7, 71.9, 71.6, 70.01, 69.91, 69.7, 69.1, 68.7, 68.2, 67.6, 66.9 (C-5 Fuc), 66.7 (C-5 Fuc), 61.4, 60.9, 60.0, 59.7, 56.1, 39.3 (CH_2-NH_3^+), 28.1 (CH_2 pentane), 26.3 (CH_2 pentane), 22.2 (CH_3 NHAc), 22.0 (CH_2 pentane), 15.39 (CH_3 Fuc), 15.37 (CH_3 Fuc). HRMS (ESI) calc. for $C_{43}H_{77}N_2O_{29}$, 1085.4607; found: 1085.4613.

5-Amino-pentyl α-L-fucopyranosyl-(1→2)-β-D-galactopyranosyl-(1→3)-*N*-acetyl-2-deoxy-4-*O*-(α-L-fucopyranosyl)-β-D-glucosaminopyranosyl-(1→3)-β-D-galactopyranosyl-(1→4)-β-D-glucopryanoside (37)

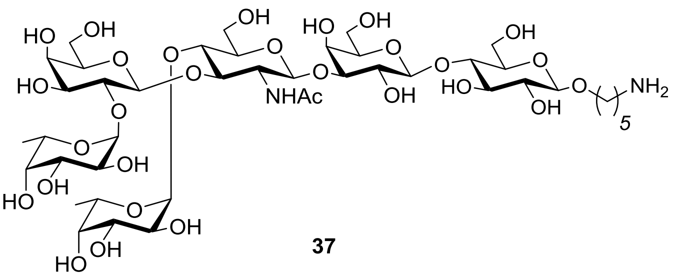

37

Compound **28** (13.7 mg, 4.87 µmol) was subjected to methanolysis followed by hydrogenolysis (see above *global deprotection* method). After RP-HPLC purification, conjugation-ready compound **37** was isolated as its formate salt (28%, 1.5 mg). ^{1}H NMR (600 MHz, D_2O) δ 8.47 (s, 1H, *H*COO^{-}), 5.17 (d, *J* = 4.0 Hz, 1H, **H-1**), 5.04 (d, *J* = 3.9 Hz, 1H, **H-1**), 4.88 (q, *J* = 6.7 Hz, 1H, H-5 Fuc), 4.67 (d, *J* = 7.7 Hz, 1H, **H-1**), 4.62 (d, *J* = 8.4 Hz, 1H, **H-1**), 4.50 (d, *J* = 8.0 Hz, 1H, **H-1**), 4.43 (d, *J* = 7.9 Hz, 1H, **H-1**), 4.36 (q, *J* = 6.7 Hz, 1H, H-5 Fuc), 4.17 – 4.12 (m, 2H), 4.01 – 3.52 (m, 29H), 3.32 (t, *J* = 8.4 Hz, 1H), 3.03 – 2.98 (m, 2H, CH_2-NH_3^+), 2.08 (s, 3H, CH_3 NHAc), 1.75 – 1.65 (m, 4H, 2x CH_2 pentane), 1.47 (p, *J* = 7.7 Hz, 2H, CH_2 pentane), 1.31 – 1.26 (m, 6H, 2x CH_3 Fuc). ^{13}C NMR (151 MHz, D_2O) δ 174.1 (C=O NHAc), 103.2 (**C-1**, $J_{C\text{-}H}$ = 167 Hz), 102.9 (**C-1**, $J_{C\text{-}H}$ = 164 Hz), 101.9 (**C-1**, $J_{C\text{-}H}$ = 161 Hz), 100.6 (**C-1**, $J_{C\text{-}H}$ = 167 Hz), 99.5 (**C-1**, $J_{C\text{-}H}$ = 175 Hz), 97.7 (**C-1**, $J_{C\text{-}H}$ = 172 Hz), 81.5, 78.2, 76.4, 75.1, 74.8, 74.7, 74.41, 74.35, 73.6, 72.7, 71.9, 71.7, 70.1, 70.0, 69.4, 69.0, 68.7, 68.5, 68.2, 67.7, 67.0 (C-5 Fuc), 66.2 (C-5 Fuc), 61.5, 60.9, 60.0, 59.4, 55.7, 39.3 (CH_2-NH_2), 28.1 (CH_2 pentane), 26.4 (CH_2 pentane), 22.1 (CH_3 NHAc), 22.0 (CH_2 pentane), 15.29 (CH_3 Fuc), 15.24 (CH_3 Fuc). HRMS (ESI) calc. for $C_{43}H_{77}N_2O_{29}$, 1085.4607; found: 1085.4615.

5-Amino-pentyl β-D-galactopyranosyl-(1→3)-*N*-acetyl-2-deoxy-4-*O*-(α-L-fucopyranosyl)-β-D-glucosaminopyranosyl-(1→3)-β-D-galactopyranosyl-(1→4)-β-D-glucopryanoside (38)

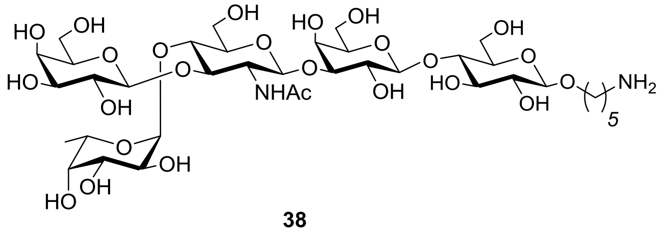

38

Compound **29** (22.7 mg, 9.50 µmol) was subjected to methanolysis followed by hydrogenolysis (see above *global deprotection* method). After RP-HPLC purification, conjugation-ready compound **38** was isolated as its formate salt (23%, 2.2 mg). ^{1}H NMR (600 MHz, D_2O) δ 8.47 (s, 1H, *H*COO^{-}), 5.05 (d, *J* = 3.9 Hz, 1H, **H-1**), 4.90 (q, *J* = 7.4 Hz, 1H, H-5 Fuc), 4.72 (d, *J* = 8.7 Hz, 1H, **H-1**), 4.55 – 4.49 (m, 2H, 2x **H-1**), 4.46 (d, *J* = 7.8 Hz, 1H, **H-1**),

4.18 (d, J = 2.8 Hz, 1H), 4.10 (t, J = 9.7 Hz, 1H), 4.02 – 3.93 (m, 4H), 3.93 – 3.86 (m, 3H), 3.85 – 3.70 (m, 11H), 3.68 – 3.54 (m, 7H), 3.54 – 3.49 (m, 1H), 3.33 (t, J = 8.5 Hz, 1H), 3.03 (t, J = 7.5 Hz, 2H, CH_2-NH_3^+), 2.06 (s, 3H, CH_3 NHAc), 1.78 – 1.64 (m, 4H, 2x CH_2 pentane), 1.48 (p, J = 7.8 Hz, 2H, CH_2 pentane), 1.20 (d, J = 6.6 Hz, 3H, CH_3 Fuc). ^{13}C NMR (151 MHz, D_2O) δ 174.7 (H*C*OO$^-$), 171.0 (C=O NHAc), 102.9 (**C-1**, $J_{C\text{-}H}$ = 163 Hz), 102.8 (**C-1**, $J_{C\text{-}H}$ = 163 Hz), 102.5 (**C-1**, $J_{C\text{-}H}$ = 163 Hz), 101.9 (**C-1**, $J_{C\text{-}H}$ = 165 Hz), 98.0 (**C-1**, $J_{C\text{-}H}$ = 172 Hz), 82.0, 78.4, 75.8, 75.2, 74.82, 74.76, 74.71, 74.4, 72.7, 72.3, 72.1, 71.9, 70.4, 70.0, 69.9, 69.1, 68.28, 68.21, 67.7, 66.8, 61.6, 60.9, 60.0, 59.6, 55.8, 39.3 (*C*H_2-NH_3^+), 28.1 (*C*H_2 pentane), 26.4 (*C*H_2 pentane), 22.2 (*C*H_3 NHAc), 22.0 (*C*H_2 pentane), 15.3 (*C*H_3 Fuc). HRMS (ESI) calc. for $C_{37}H_{67}N_2O_{25}$, 939.4027; found: 939.4044.

Global Deprotection: Birch Reduction - Methanolysis

AGA-synthesized protected oligosaccharide was dissolved in anhydrous THF (0.55 mL) and *t*-BuOH (0.1 mL). The solution was added to a three-necked flask containing a dark-blue solution of sodium (30.0 mg, 1.30 mmol) in liquid ammonia (20.0 mL), under argon at -78 °C. The dark-blue mixture was stirred at -78 °C for 50 min. Then MeOH (4.00 mL) was added dropwise until the solution became colorless. The mixture was concentrated with a stream of argon, allowed to warm to room temperature and stirred overnight. Then AcOH was added dropwise to neutralize the solution. The solvent was evaporated and the reaction crude was purified through a Sephadex G-25 size-exclusion column using water as eluent. Subsequent reverse phase chromatography using a Chromabond® SPE catridge (MeOH in water 1 to 10%) afforded deprotected, conjugation-ready oligosaccharide as is acetate salt.

Experimental Data for Deprotected Oligosaccharides 39-40

5-Amino-pentyl β-D-galactopyranosyl-(1→4)-3-*O*-(α-L-fucopyranosyl)-β-D-glucosaminopyranosyl-(1→3)-β-D-galactopyranosyl-(1→4)-3-*O*-(α-L-fucopyranosyl)-β-D-glucosaminopyranosyl-(1→3)-β-D-galactopyranosyl-(1→4)- β-D-glucopyranoside (39)

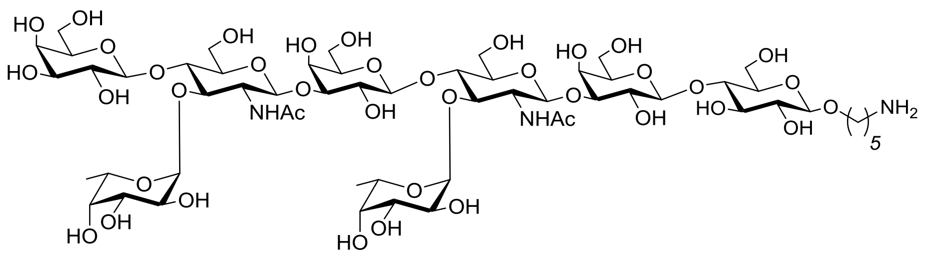

39

Protected Le^x-dimer **25** (35.1 mg, 9.6 µmol) was deprotected following the experimental procedure for *Birch reduction and methanolysis* (see above). Purification using size-exclusion followed by reverse-phase chromatography afforded the acetate salt of Le^x-dimer **39** (1.83 mg, 13%). ^{1}H NMR (700 MHz, D_2O) δ 5.14 (d, *J* = 4.0 Hz, 1H, **H-1** Fuc), 5.12 (d, *J* = 4.0 Hz, 1H, **H-1** Fuc), 4.87 – 4.81 (m, 2H), 4.74 – 4.69 (m, 1H, **H-1**), 4.50 – 4.42 (m, 5H, 5 x **H-1**), 4.16 (d, *J* = 3.1 Hz, 1H), 4.11 (d, *J* = 3.0 Hz, 1H), 4.00 – 3.94 (m, 7H), 3.93 – 3.86 (m, 7H), 3.81 – 3.77 (m, 4H), 3.76 – 3.68 (m, 11H), 3.67 – 3.63 (m, 3H), 3.62 – 3.57 (m, 6H), 3.54 – 3.48 (m, 2H), 3.31 (t, *J* = 8.5 Hz, 1H), 3.02 (d, *J* = 7.7 Hz, 3H, $CH_2NH_3^+$), 2.03 (da, 6H, 2 x CH_3 NHAc), 1.92 (s, 3H, CH_3 AcO^-), 1.73 – 1.65 (m, 4H, 2 x CH_2 pentane), 1.47 (p, *J* = 7.7 Hz, 2H, CH_2 pentane), 1.18 (d, *J* = 6.6 Hz, 3H, CH_3 Fuc), 1.16 (d, *J* = 6.6 Hz, 3H, CH_3 Fuc). ^{13}C NMR (176 MHz, D_2O) δ 181.5 (C=O AcO^-), 174.71 (C=O NHAc), 174.65 (C=O NHAc), 103.0 (2 x **C-1**, $J_{C\text{-}H}$ = 164 Hz), 102.5 (**C-1**, $J_{C\text{-}H}$ = 164 Hz), 102.0 (2 x **C-1**, $J_{C\text{-}H}$ = 163 Hz), 101.8 (**C-1**, $J_{C\text{-}H}$ = 162 Hz), 98.7 (**C-1**, $J_{C\text{-}H}$ = 174 Hz), 98.6 (**C-1**, $J_{C\text{-}H}$ = 174 Hz), 82.1, 81.7, 78.4, 75.13, 75.09, 74.92, 74.88, 74.78, 74.5, 73.1, 72.8, 72.5, 71.92, 71.86, 71.1, 70.5, 70.08, 69.95, 69.20, 69.19, 68.4, 68.28, 68.23, 67.72, 67.66, 66.7, 61.51, 61.46, 61.0, 60.1, 59.7, 56.0, 39.4 (CH_2-NH_3^+), 28.2 (CH_2 pentane), 26.4 (CH_2 pentane), 23.3 (CH_3 AcO^-), 22.3 (CH_3 NHAc), 22.1 (2C, CH_3 NHAc, CH_2 pentane), 15.3 (2 x CH_3 Fuc). HRMS (ESI) calc. for $C_{57}H_{100}N_3O_{39}$, 1450.5928; found: 1450.5925.

5-Amino-pentyl α-L-fucopyranosyl-(1→2)-β-D-galactopyranosyl-(1→4)-3-*O*-(α-L-fucopyranosyl)-β-D-glucosaminopyranosyl-(1→3)-β-D-galactopyranosyl-(1→4)-3-*O*-(α-L-fucopyranosyl)-β-D-glucosaminopyranosyl-(1→3)-β-D-galactopyranosyl-(1→4)- β-D-glucopyranoside (40)

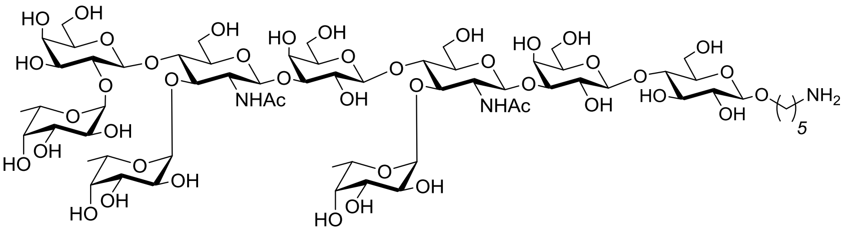

40

Protected KH-1 **26** (56.0 mg, 13.7 µmol) was deprotected following the experimental procedure for *Birch reduction and methanolysis* (see above). Purification using size-exclusion followed by reverse-phase chromatography afforded the acetate salt of KH-1 antigen **40** (4.33 mg, 19%). ^{1}H NMR (700 MHz, D_2O) δ 5.28 (d, *J* = 2.9 Hz, 1H, **H-1α**), 5.15 – 5.11 (m, 2H, 2 x **H-1α**), 4.89 (q, *J* = 7.3, 6.9 Hz, 1H), 4.84 – 4.81 (m, 1H), 4.73 – 4.70 (m, 2H, 2 x **H-1**), 4.54 – 4.51 (d, *J* = 7.6 Hz,1H, **H-1**), 4.49 (d, *J* = 8.0 Hz, 1H, **H-1**), 4.47 – 4.42 (m, 2H, 2 x **H-1**), 4.26 (q, *J* = 6.4 Hz, 1H), 4.16 (d, *J* = 2.9 Hz, 1H), 4.10 (d, *J* = 2.9 Hz, 1H), 4.01 – 3.57 (m, 42H), 3.52 (d, *J* = 8.8 Hz, 1H), 3.47 – 3.44 (m, 1H), 3.31 (t, *J* = 8.3 Hz, 1H), 3.00 (d, *J* = 7.5 Hz, 2H, $CH_2NH_3^+$), 2.03 (s, 6H, 2 x NHC(O)OCH_3), 1.92 (s, 3H, $CH_3C(O)O^-$), 1.73 – 1.65 (m, 4H, 2 x CH_2 pentane), 1.47 (p, *J* = 7.7 Hz, 2H, CH_2 pentane), 1.27 (d, *J* = 6.5 Hz, 3H, CH_3 Fuc), 1.25 (d, *J* = 6.5 Hz, 3H, CH_3 Fuc), 1.16 (d, *J* = 6.5 Hz, 3H, CH_3 Fuc). ^{13}C NMR (176 MHz, D_2O) δ 181.5 (C=O AcO^-), 174.71 (C=O NHAc), 174.66 (C=O NHAc), 103.0 (**C-1**, $J_{C\text{-}H}$ = 164 Hz), 102.5 (**C-1**, $J_{C\text{-}H}$ = 166 Hz), 102.4 (**C-1**, $J_{C\text{-}H}$ = 166 Hz), 102.0 (**C-1**, $J_{C\text{-}H}$ = 166 Hz), 101.7 (**C-1**, $J_{C\text{-}H}$ = 165 Hz), 100.2 (**C-1**, $J_{C\text{-}H}$ = 166 Hz), 99.4 (**C-1** Fuc, $J_{C\text{-}H}$ = 174 Hz), 98.7 (**C-1** Fuc, $J_{C\text{-}H}$ = 173 Hz), 98.6 (**C-1** Fuc, $J_{C\text{-}H}$ = 173 Hz), 82.1, 81.6, 78.4, 76.4, 75.3, 75.1, 74.9, 74.78, 74.74, 74.46, 74.43, 73.6, 73.0, 72.8, 72.0, 71.9, 71.7, 70.6, 70.1, 70.0, 69.7, 69.2, 68.8, 68.28, 68.21, 67.71, 67.65, 67.0, 66.8, 66.7, 61.5, 61.4, 61.0, 60.1, 59.8, 59.7, 56.1, 48.9, 39.4 (CH_2-NH_3^+), 28.2 (CH_2 pentane), 26.7 (CH_2 pentane), 23.3 (CH_3 AcO^-), 22.3 (2 x CH_3 NHAc), 22.1 (CH_2 pentane), 15.5 (CH_3 Fuc), 15.4 (CH_3 Fuc), 15.3 (CH_3 Fuc). HRMS (ESI) calc. for $C_{63}H_{110}N_3O_{43}$, 1596.6508; found: 1596.6504.

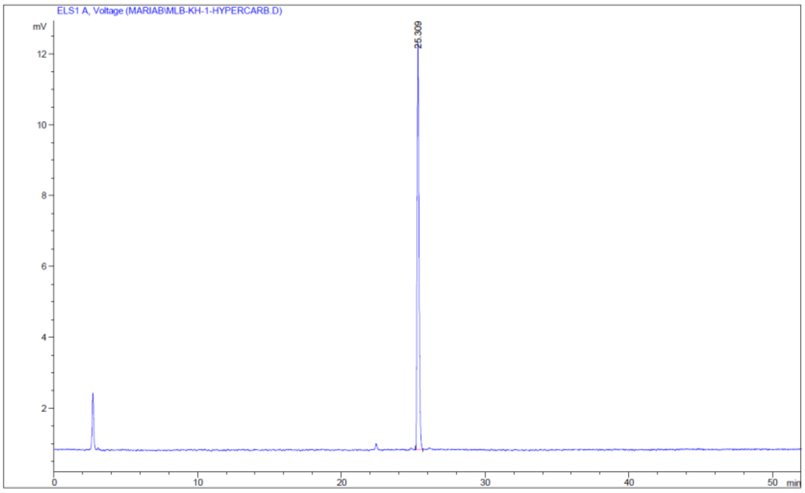

RP-HPLC of final product ***40*** *(detection: ELSD).*

2. NMR spectra of all new compounds

^1H NMR: **14**

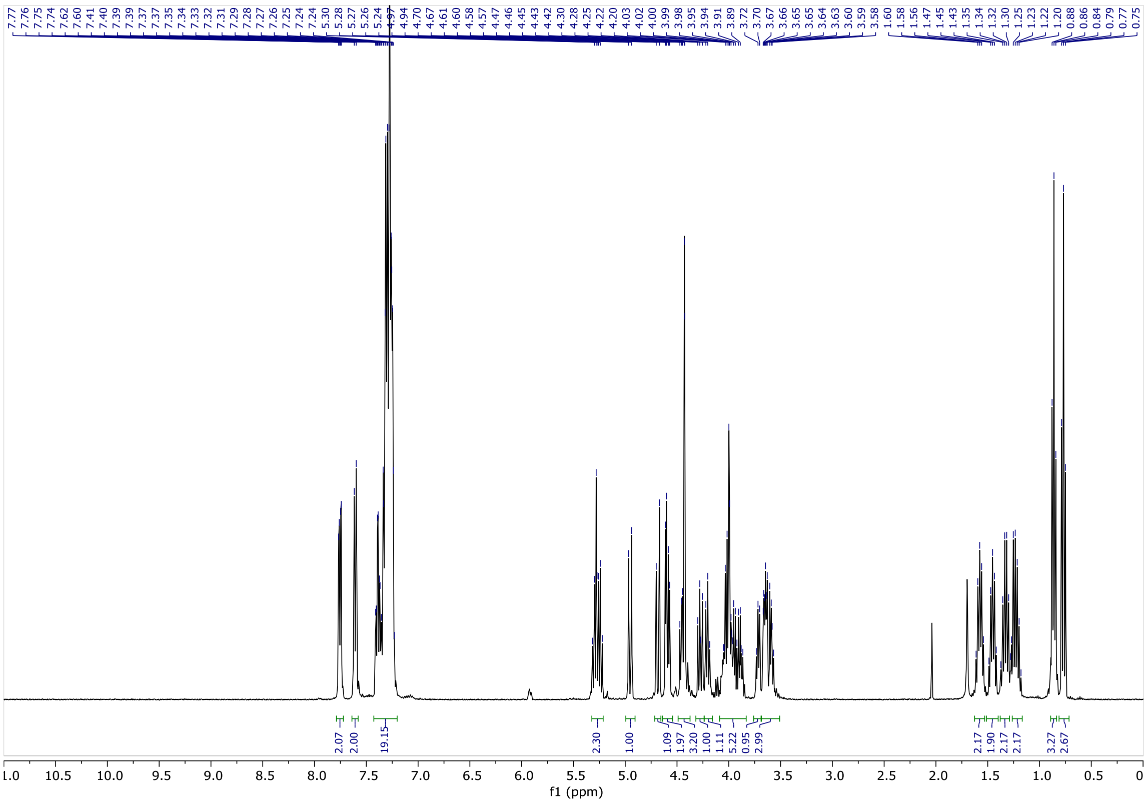

^{13}C NMR: **14**

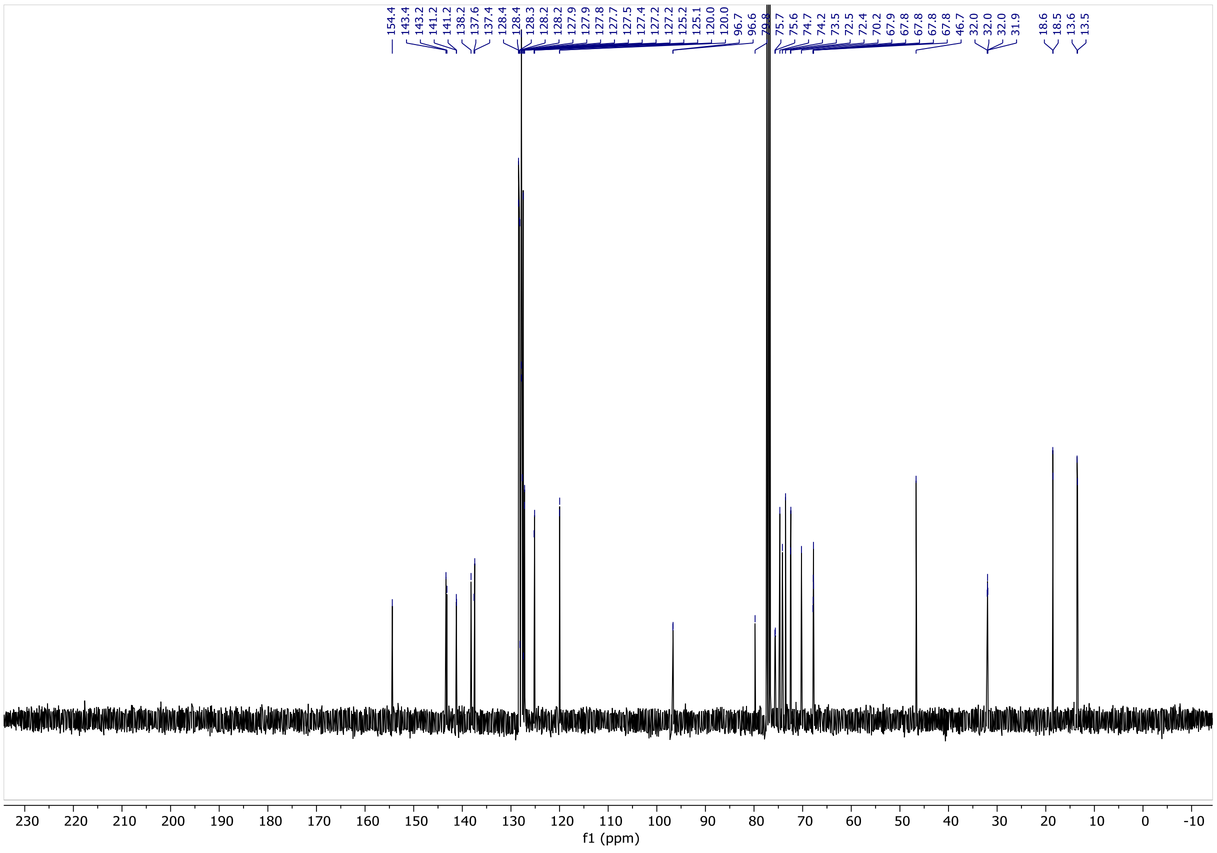

^{31}P NMR: **14**

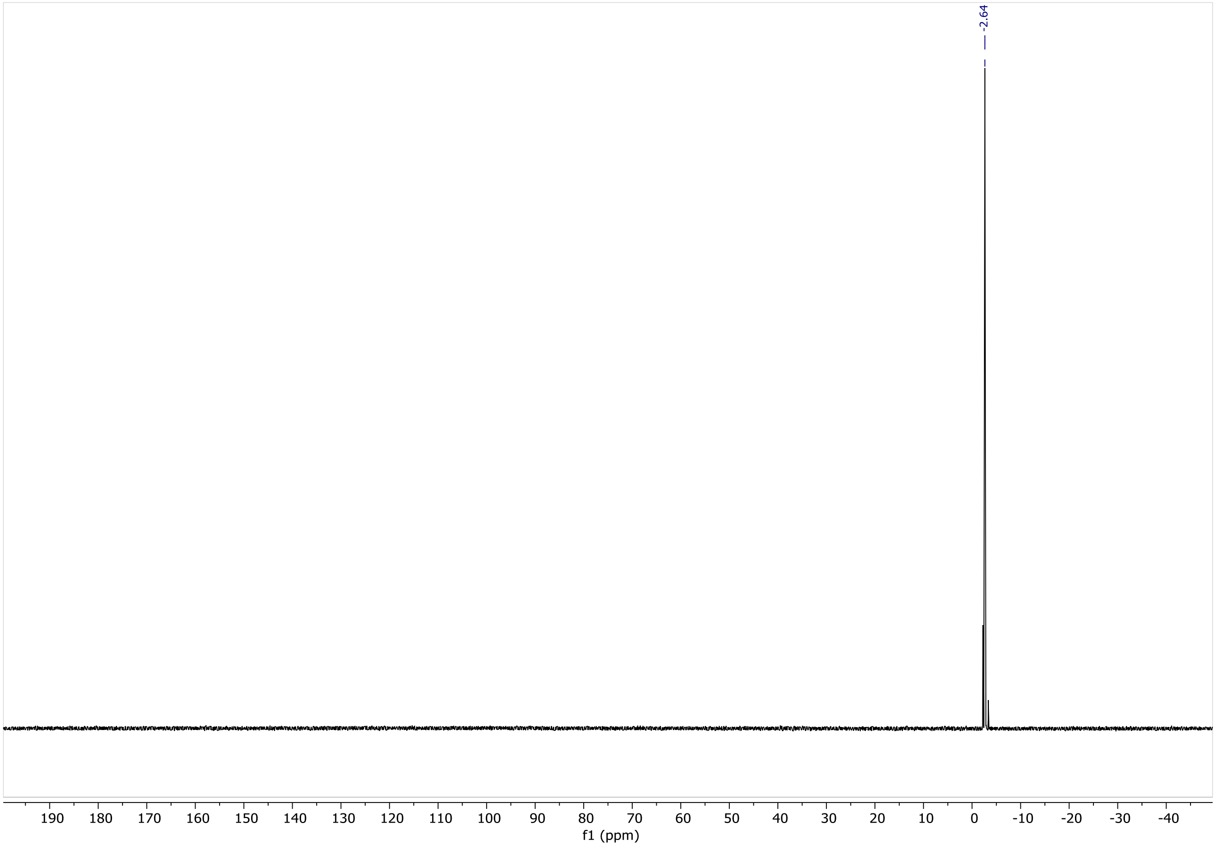

COSY: **14**

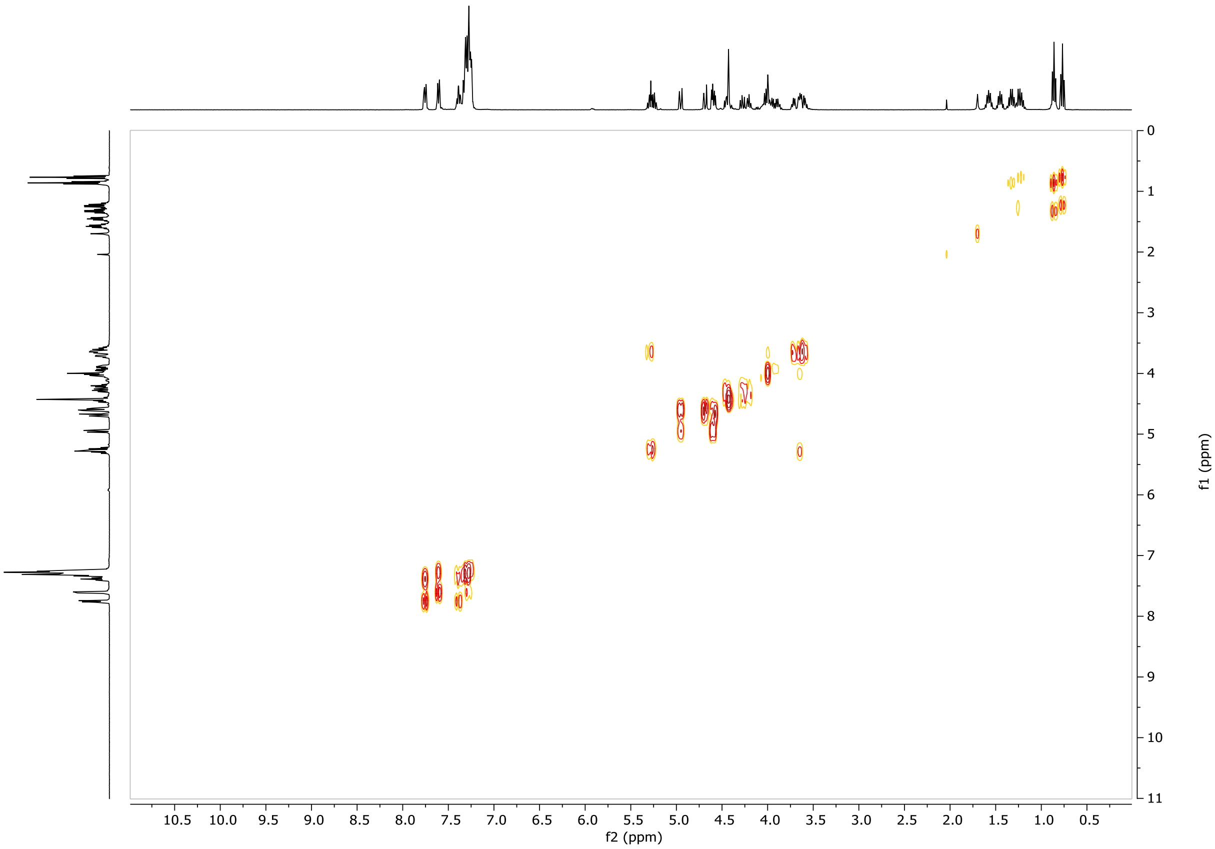

HSQC: **14**

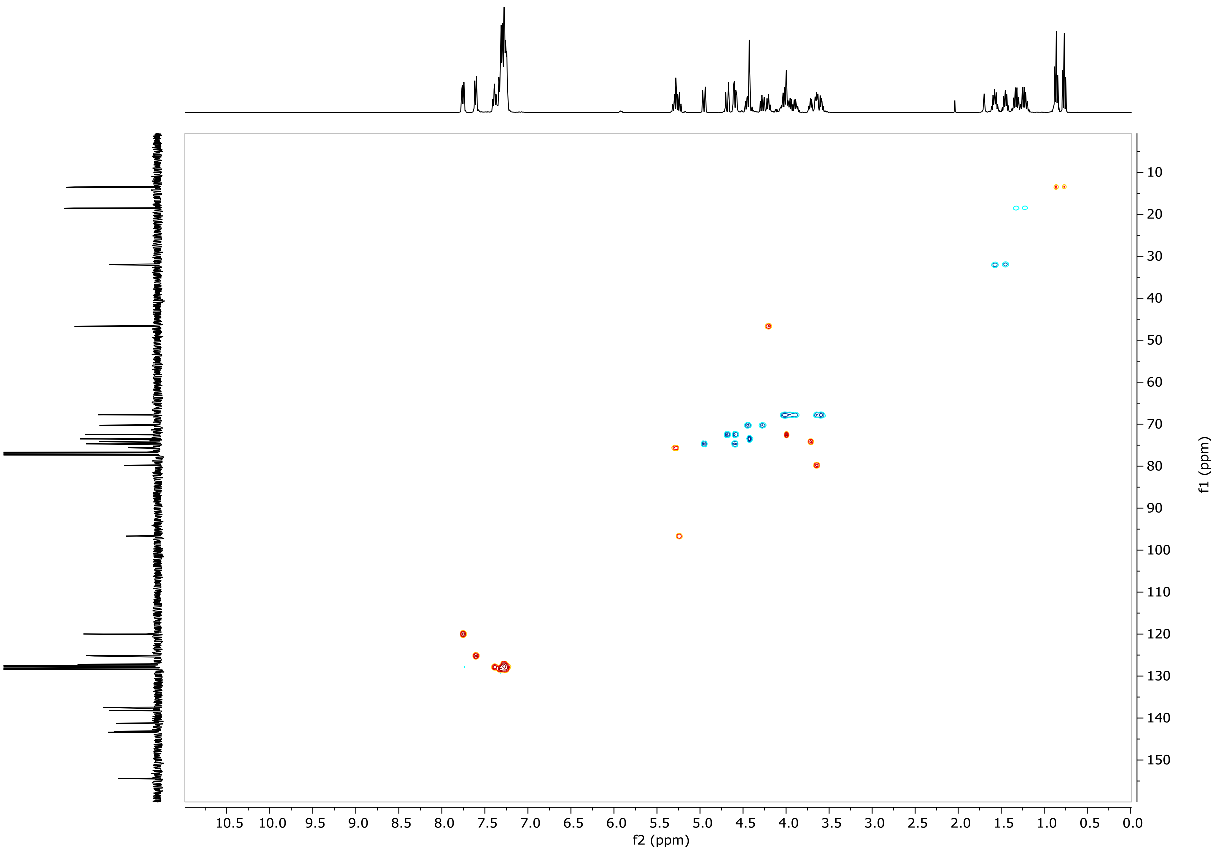

^{1}H NMR: **17**

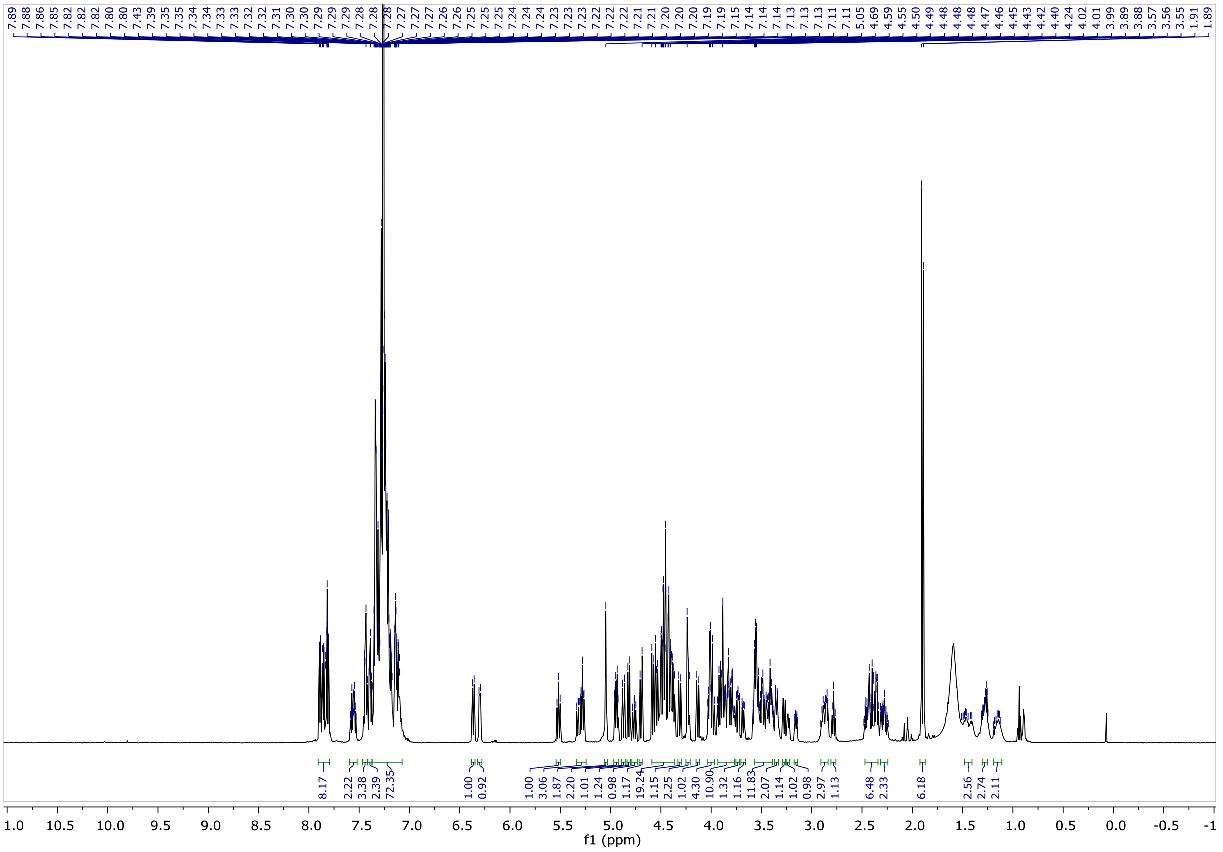

^{13}C NMR: **17**

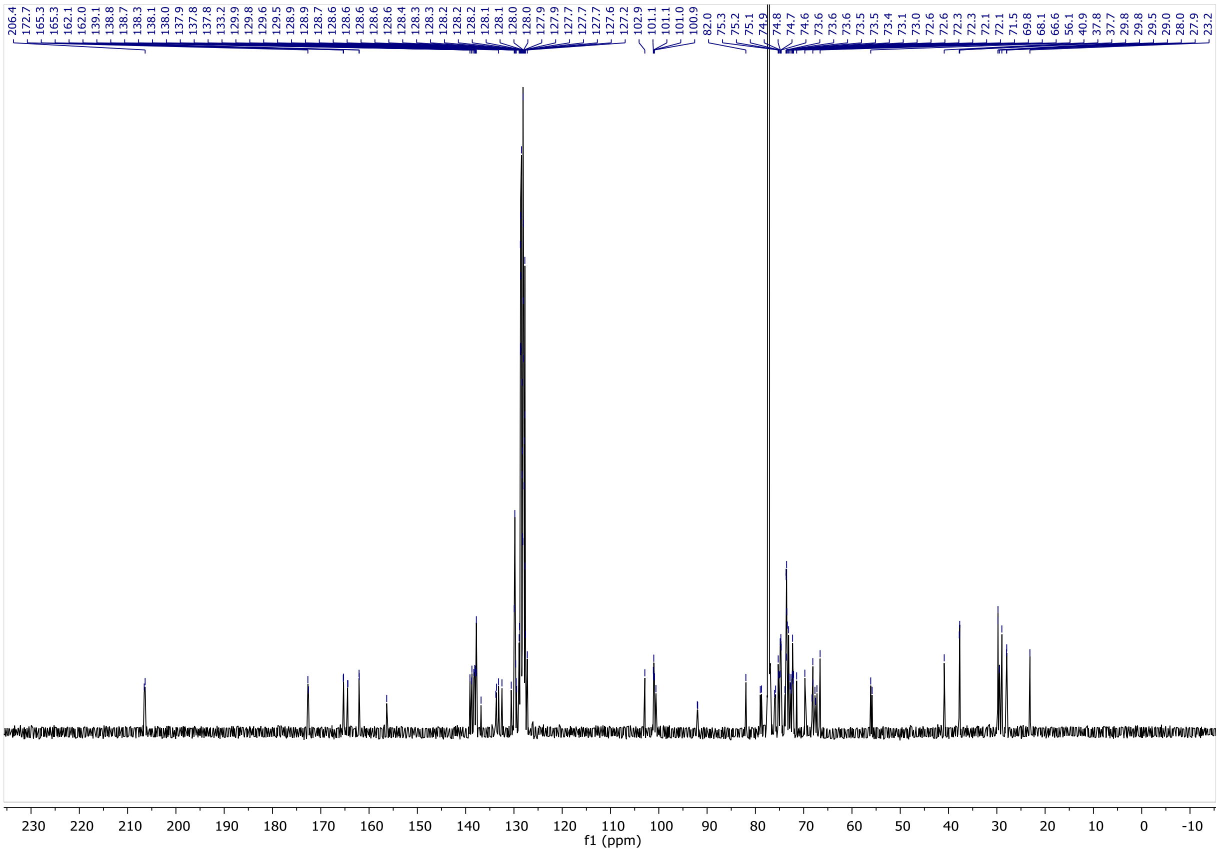

COSY: **17**

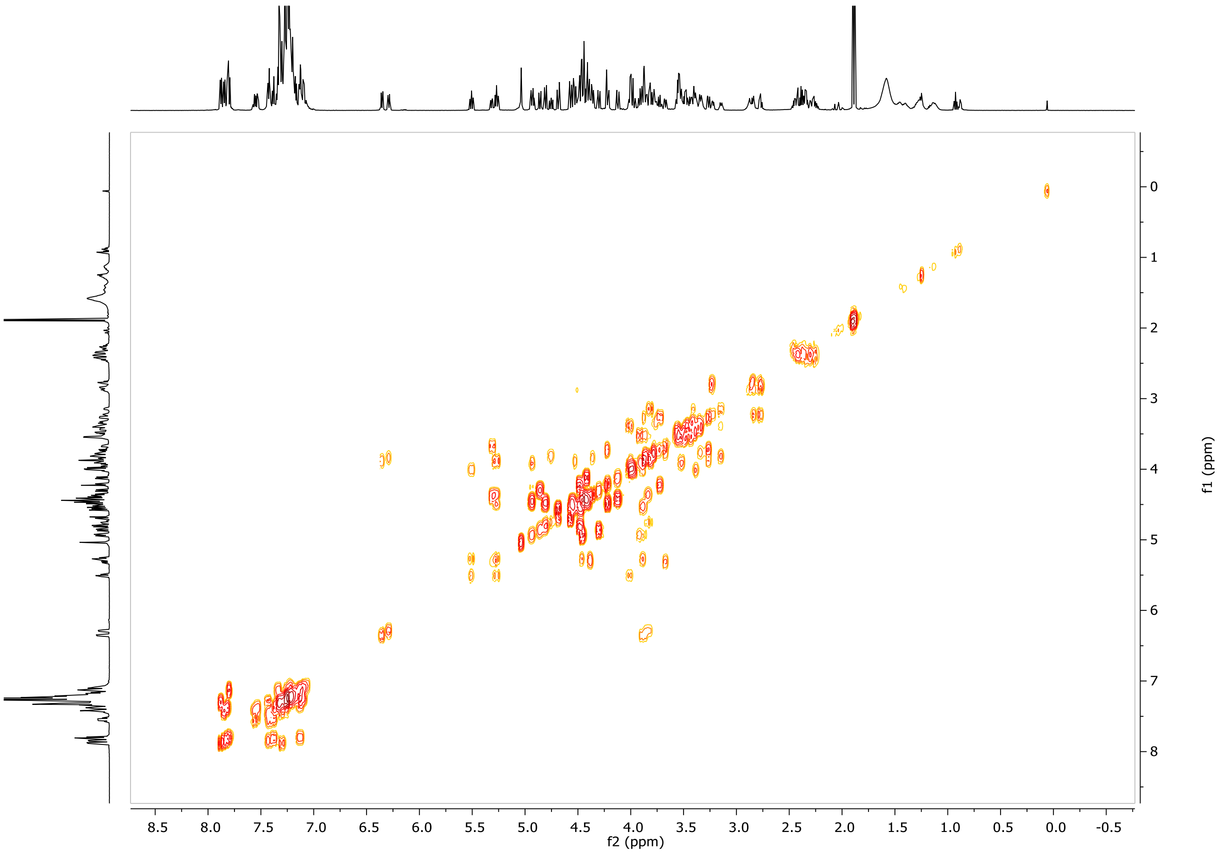

HSQC: **17**

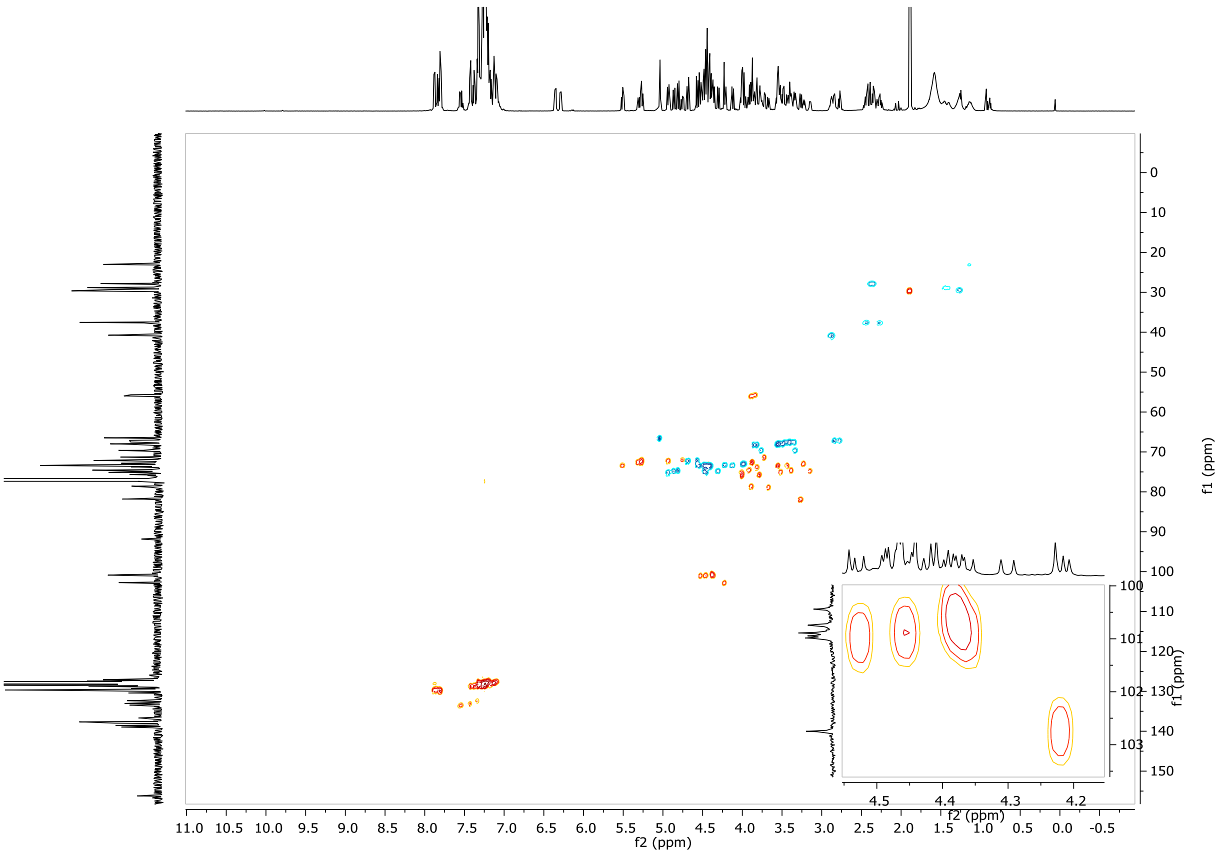

Coupled HSQC: **17**

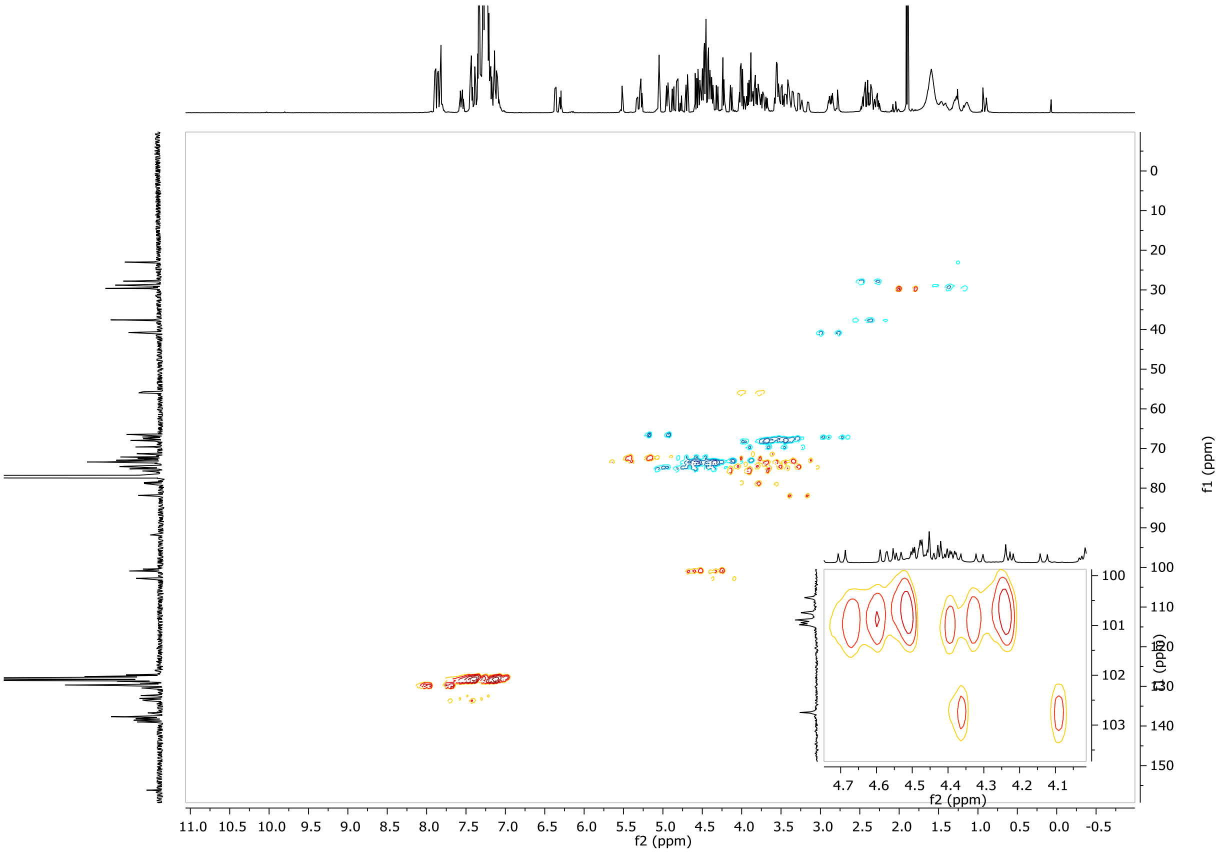

^{1}H NMR: **18**

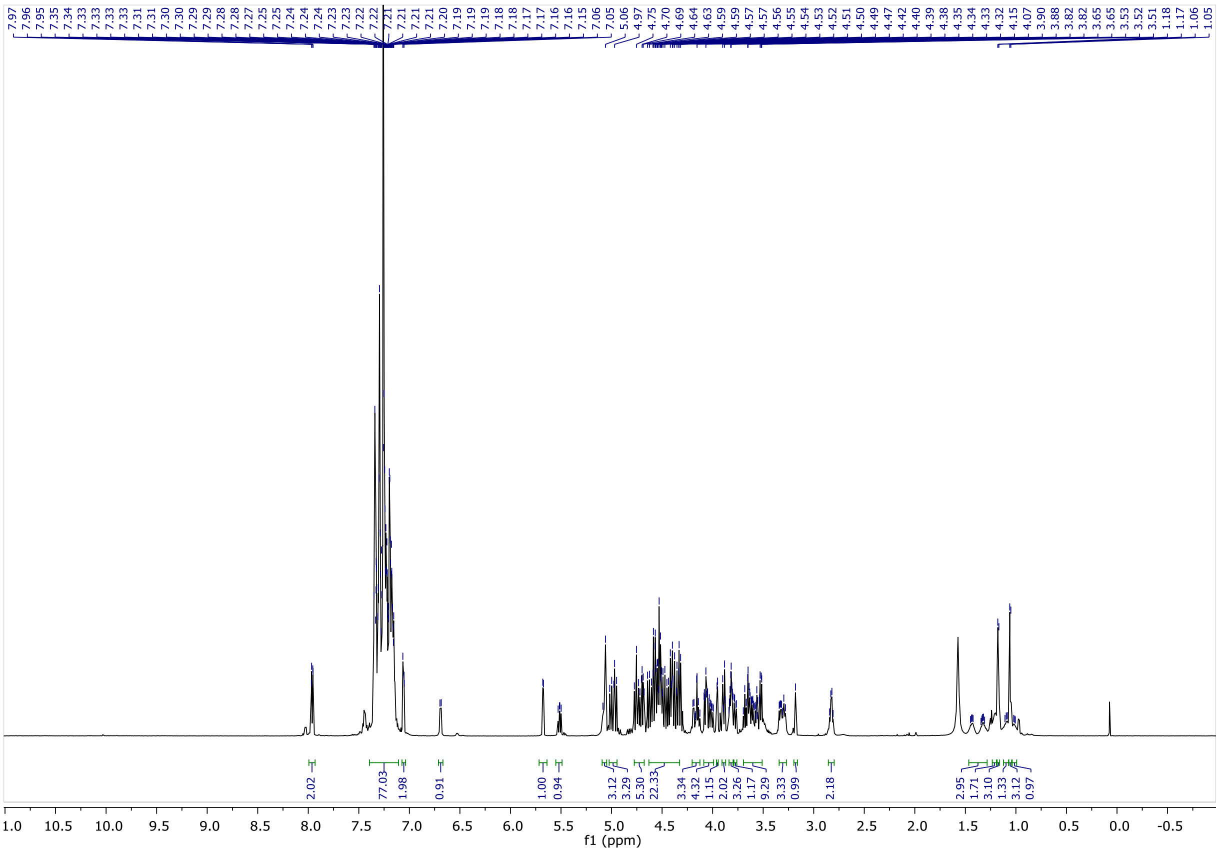

^{13}C NMR: **18**

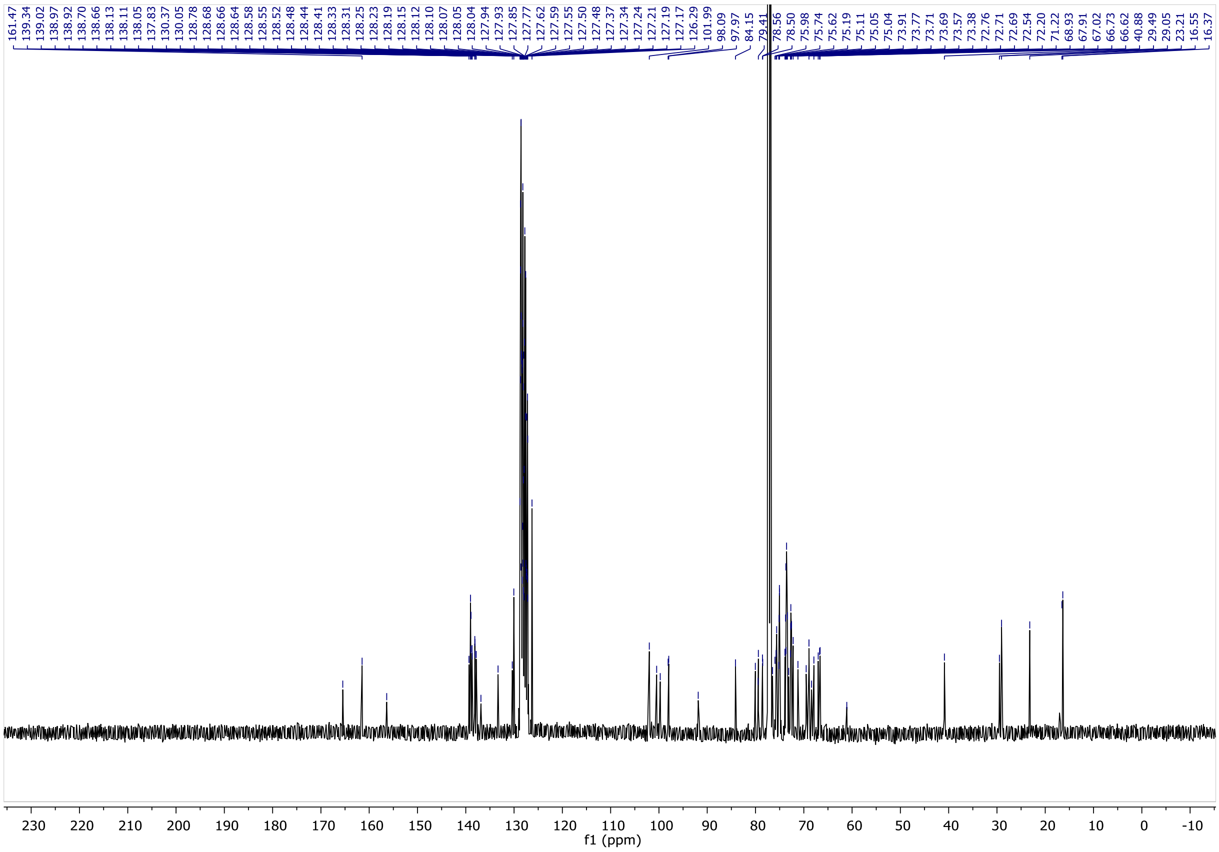

COSY: **18**

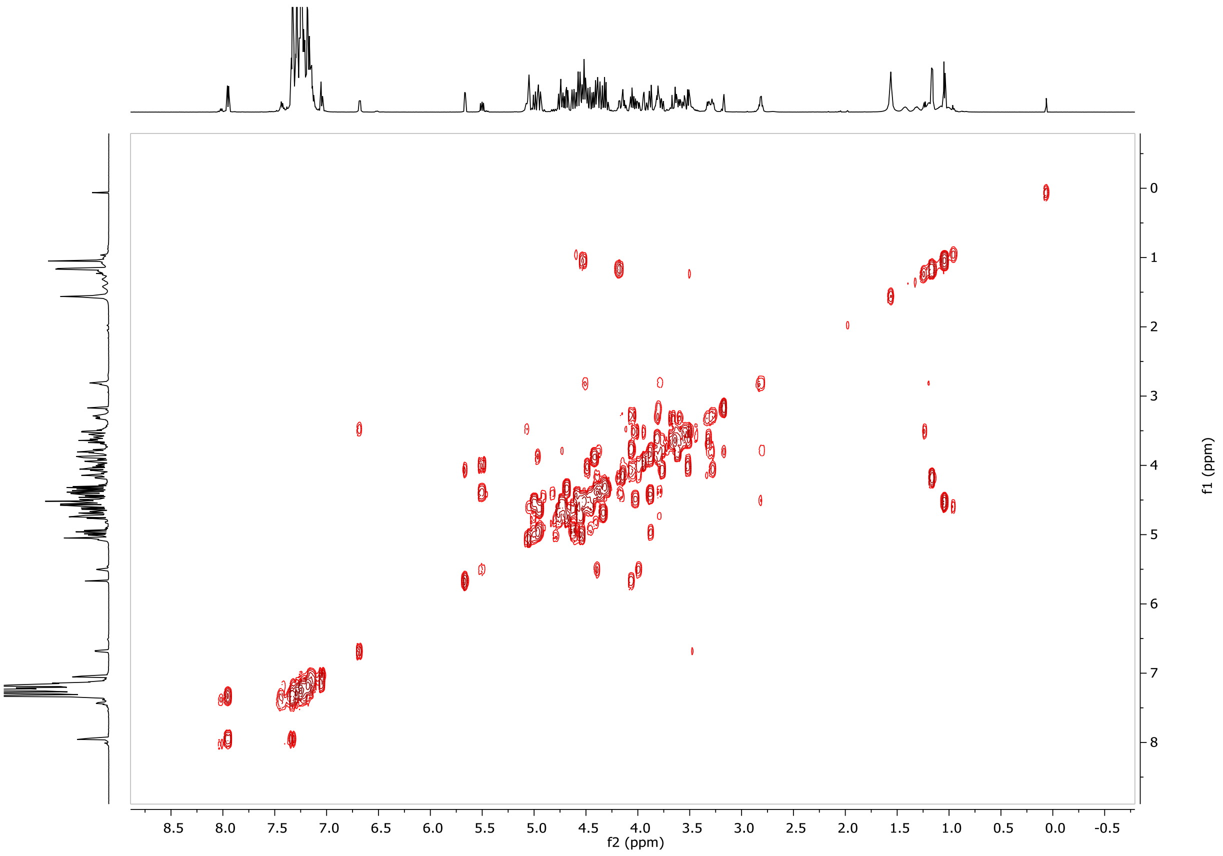

HSQC: **18**

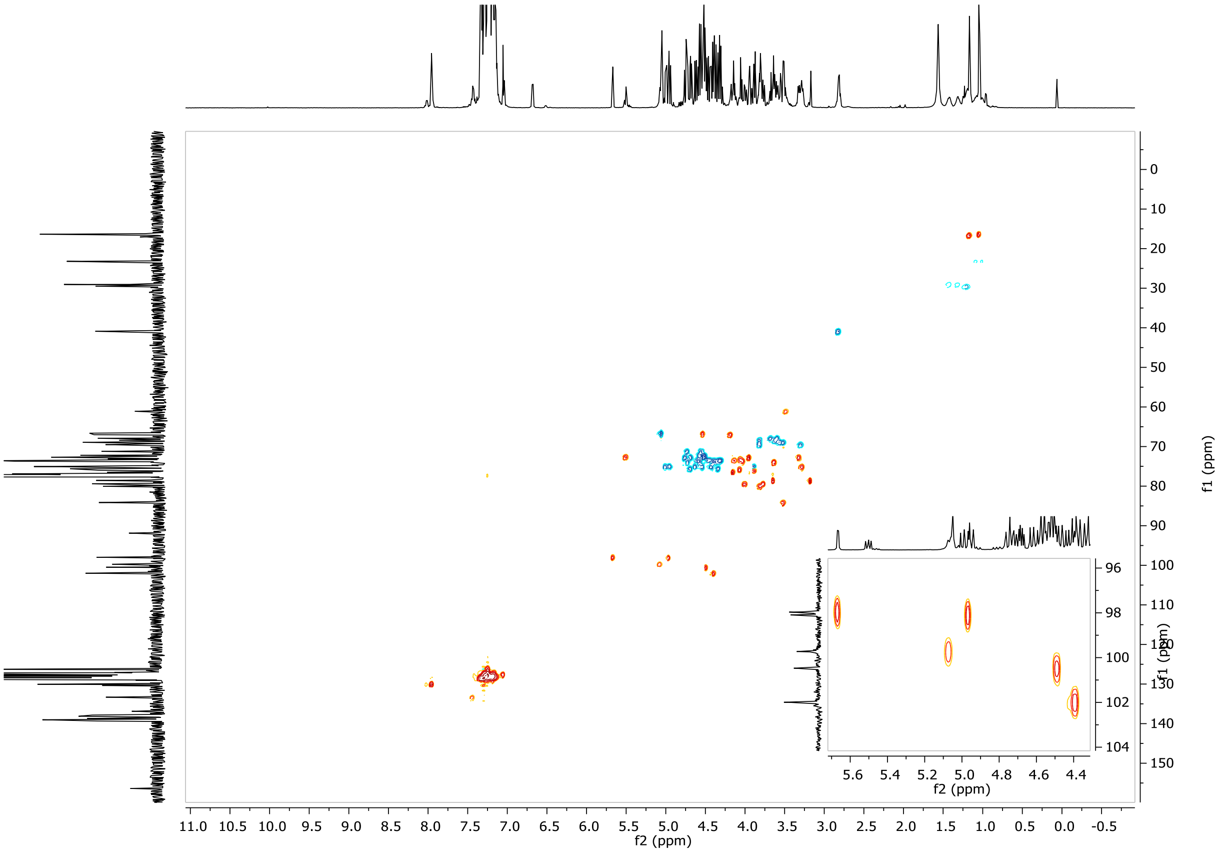

Coupled HSQC: **18**

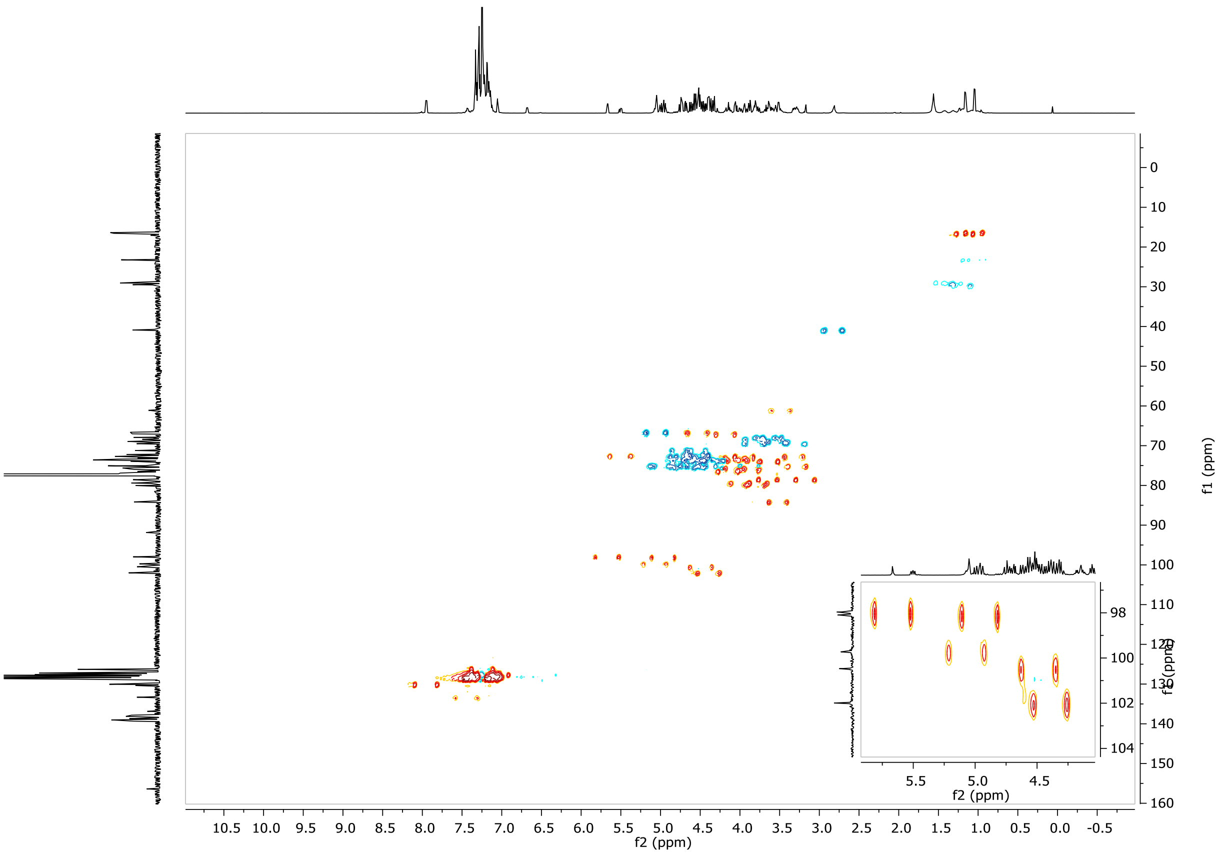

^{1}H NMR: **19**

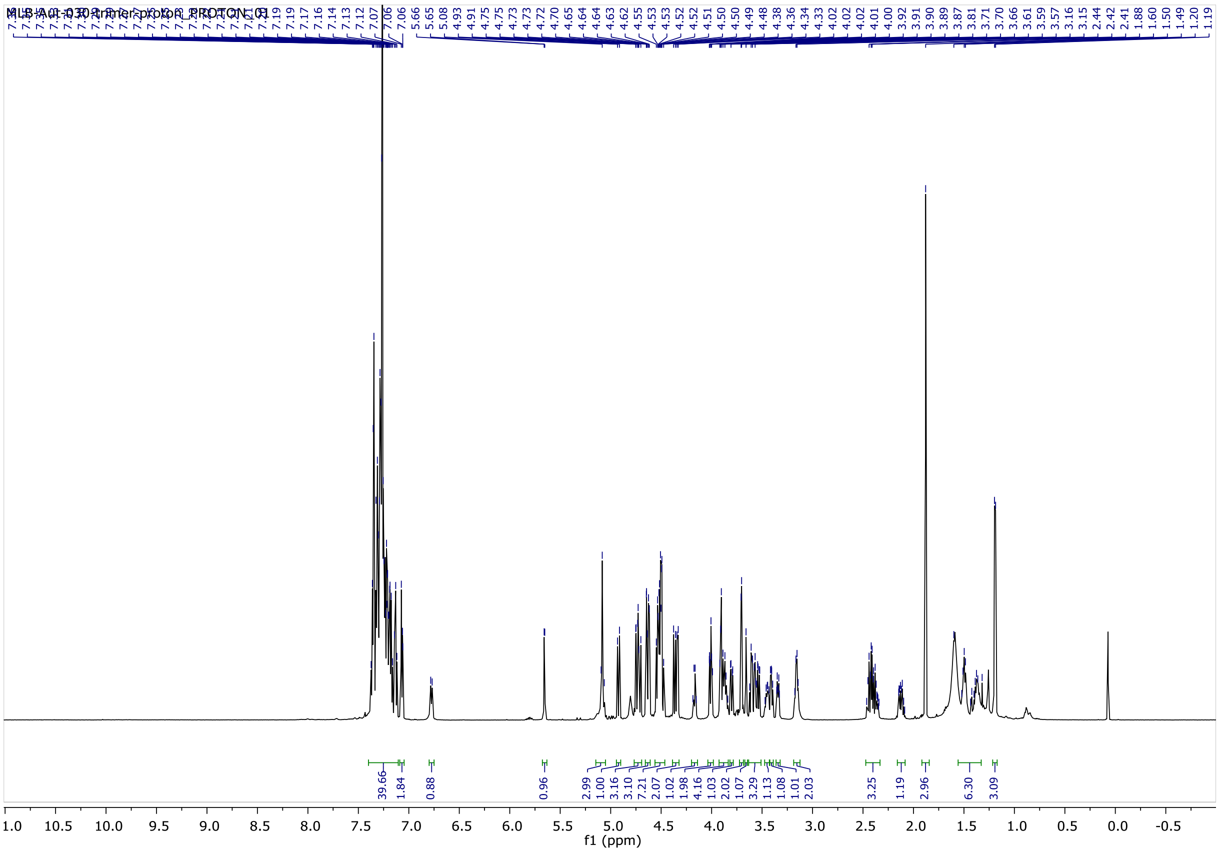

^{13}C NMR: **19**

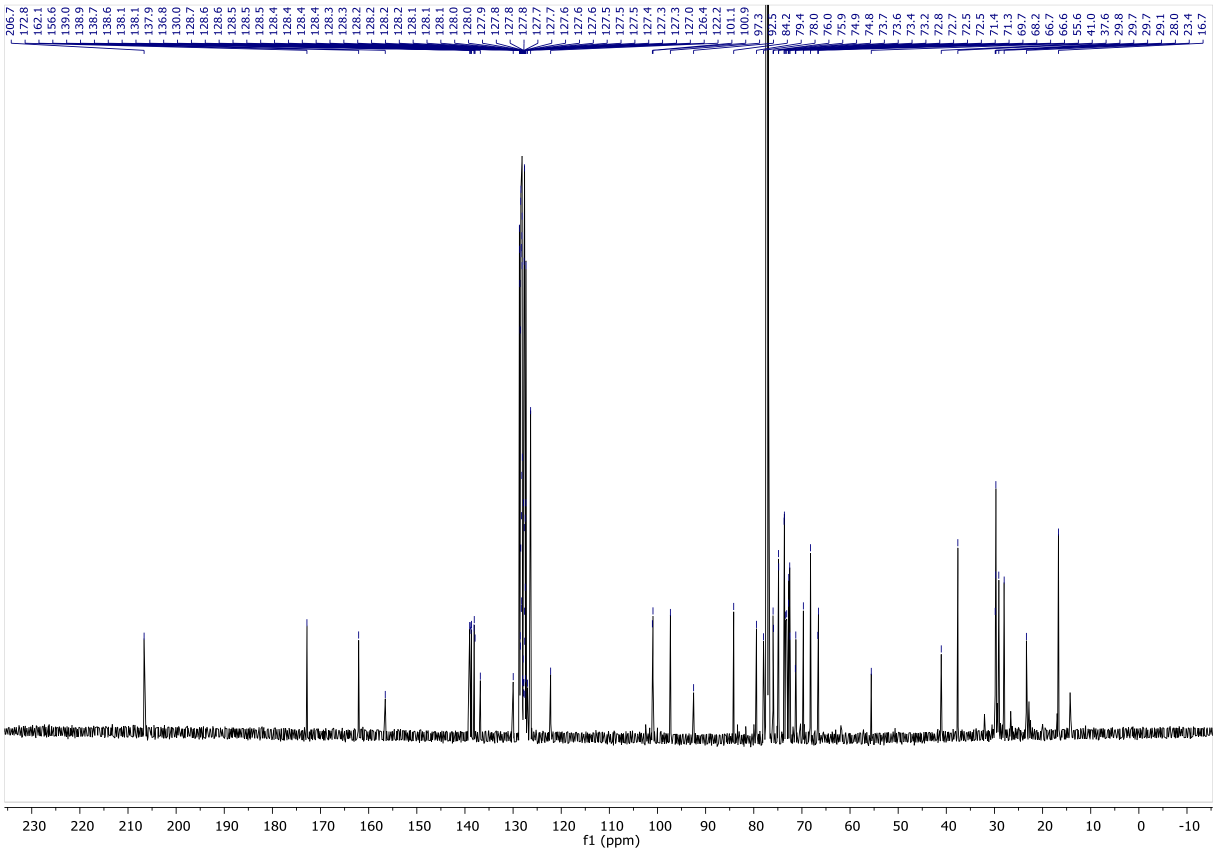

COSY: **19**

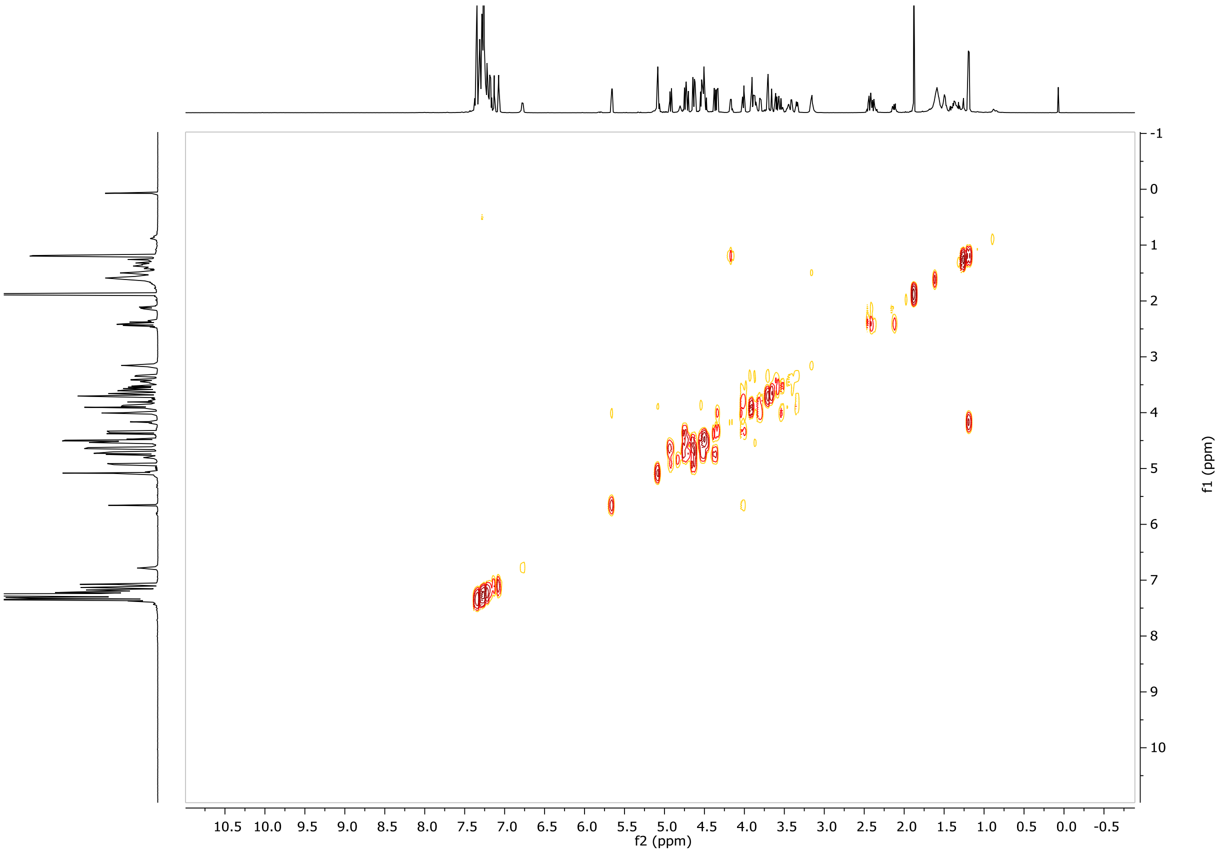

HSQC: **19**

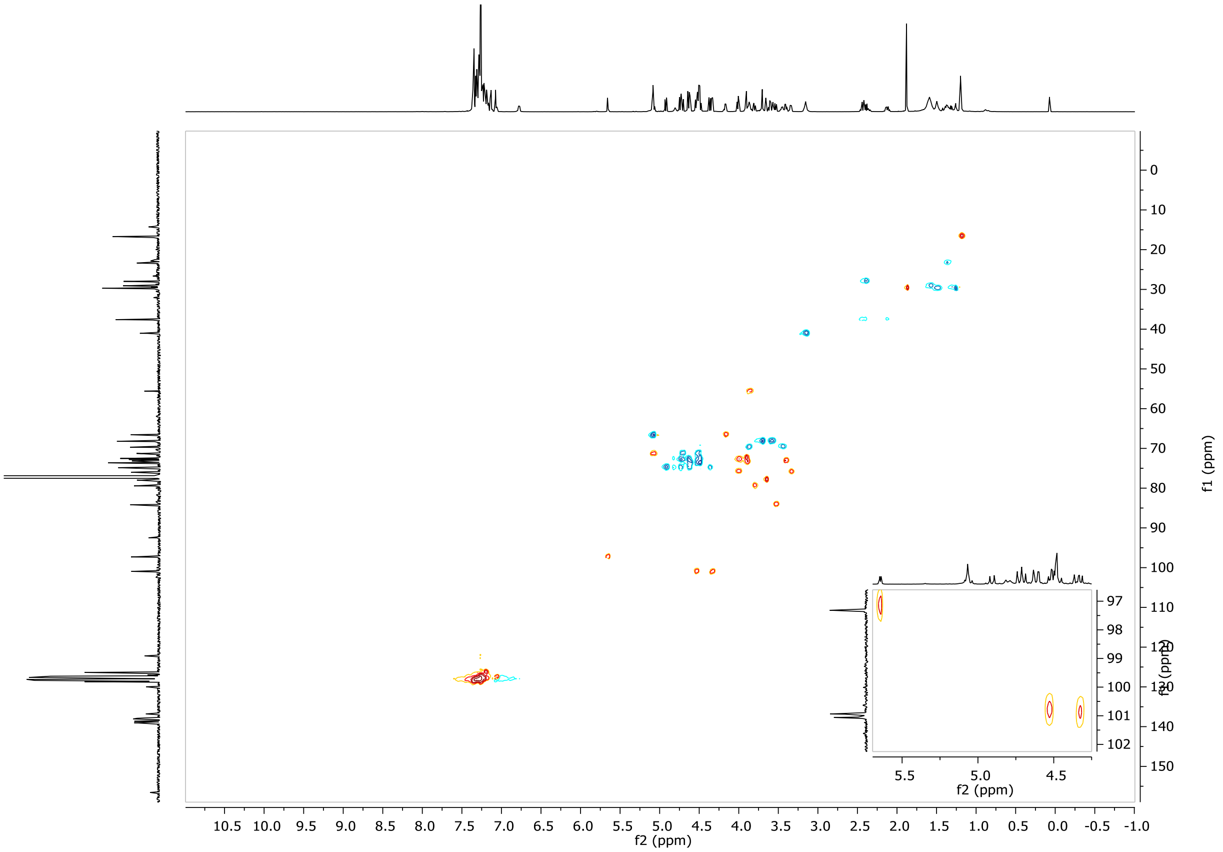

Coupled HSQC: **19**

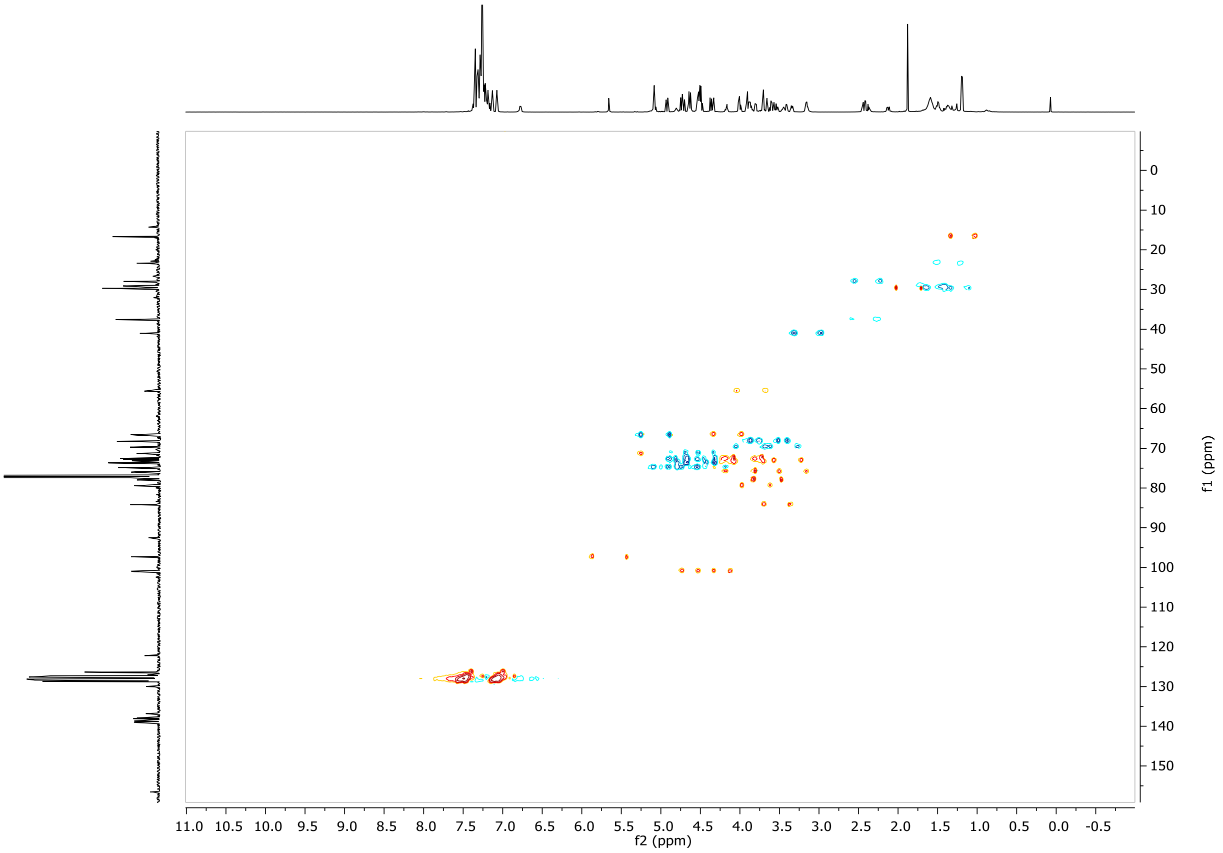

^{1}H NMR: **20**

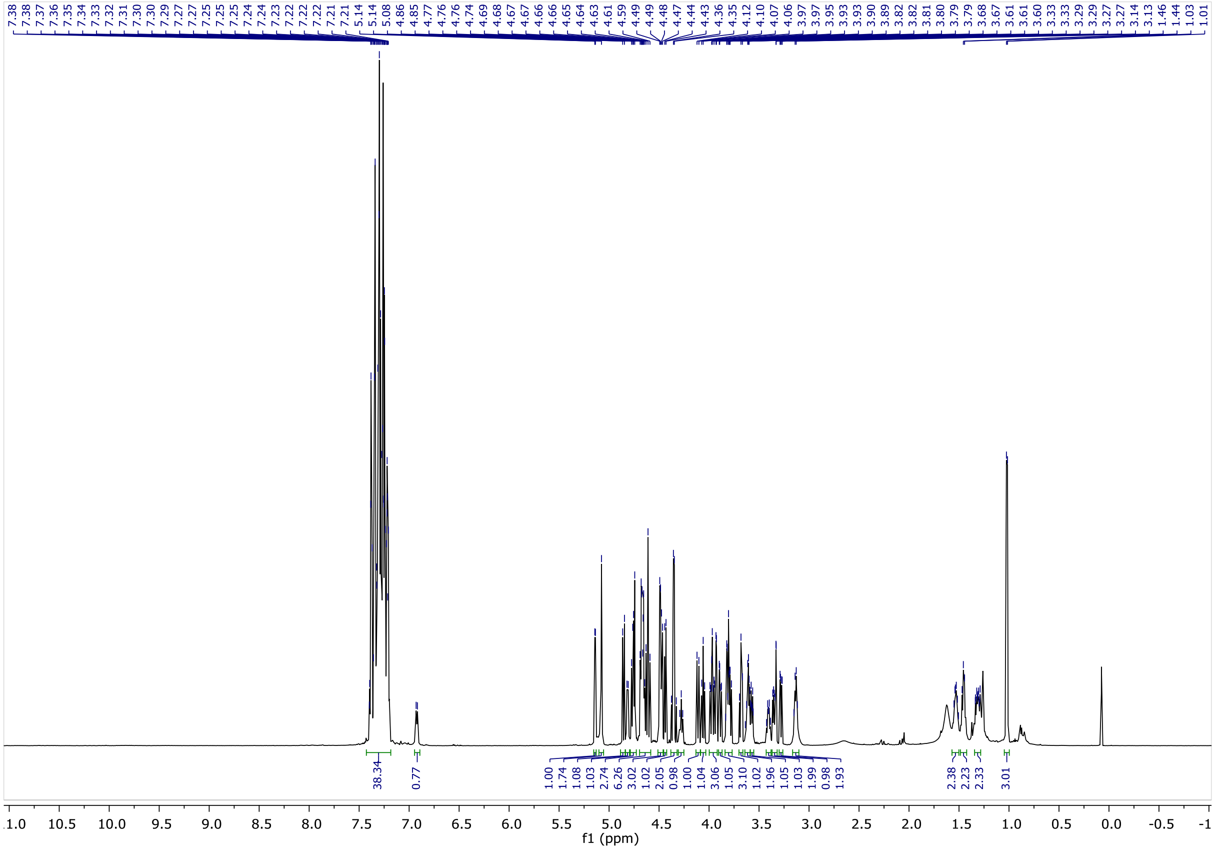

^{13}C NMR: **20**

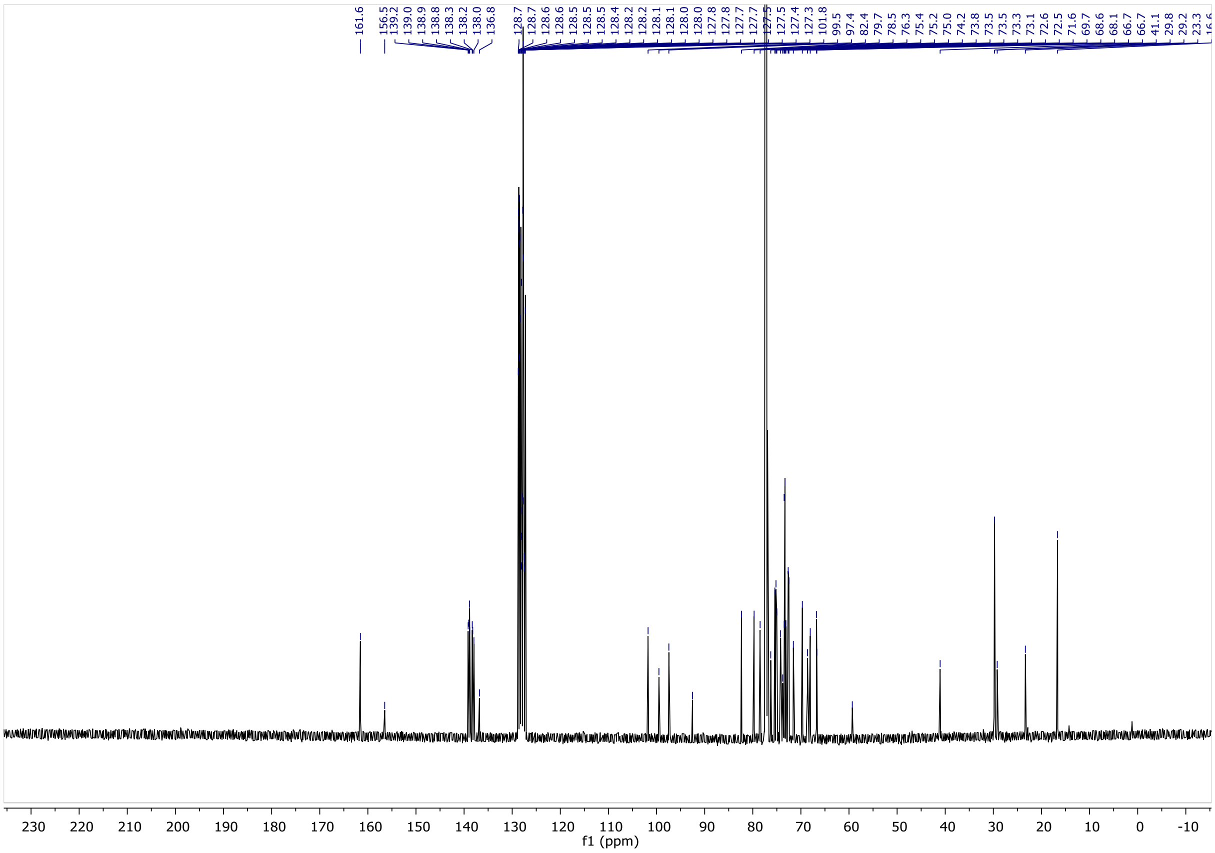

COSY: **20**

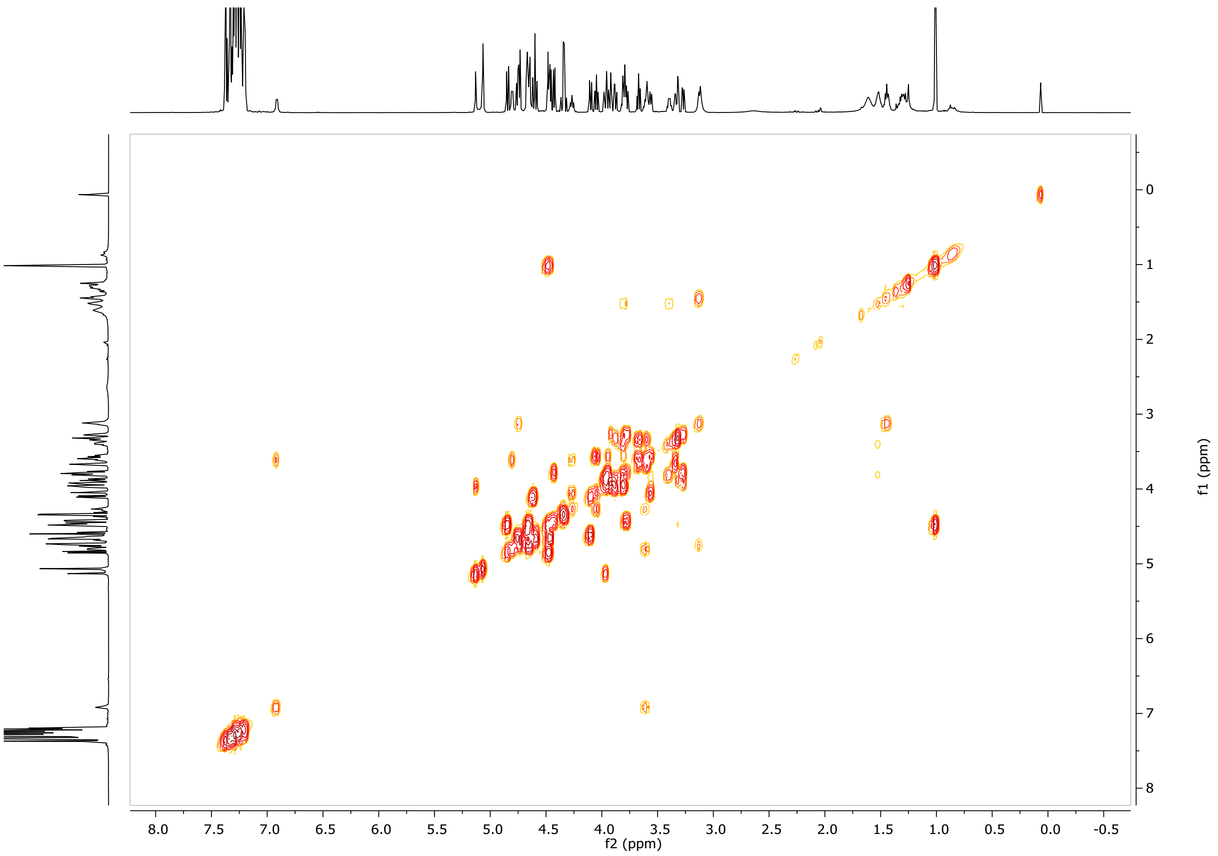

HSQC: **20**

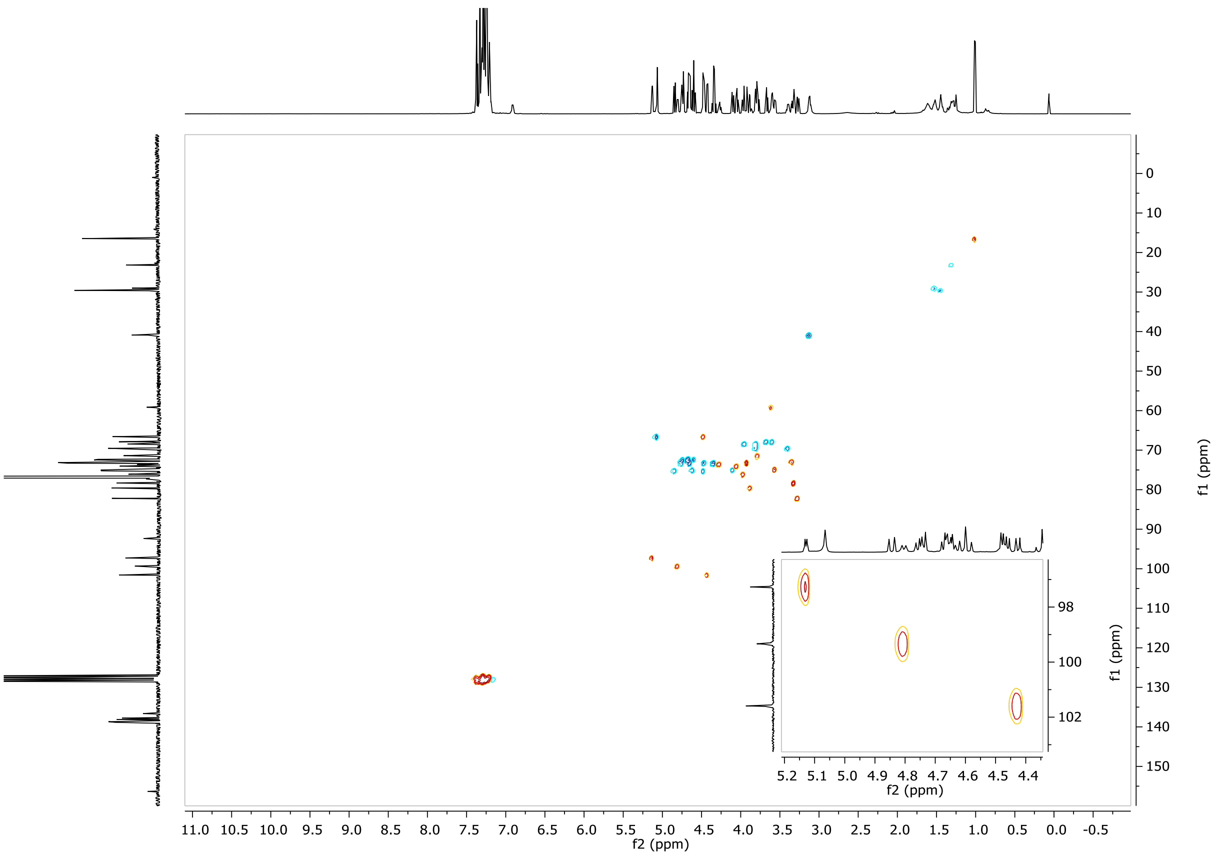

Coupled HSQC: **20**

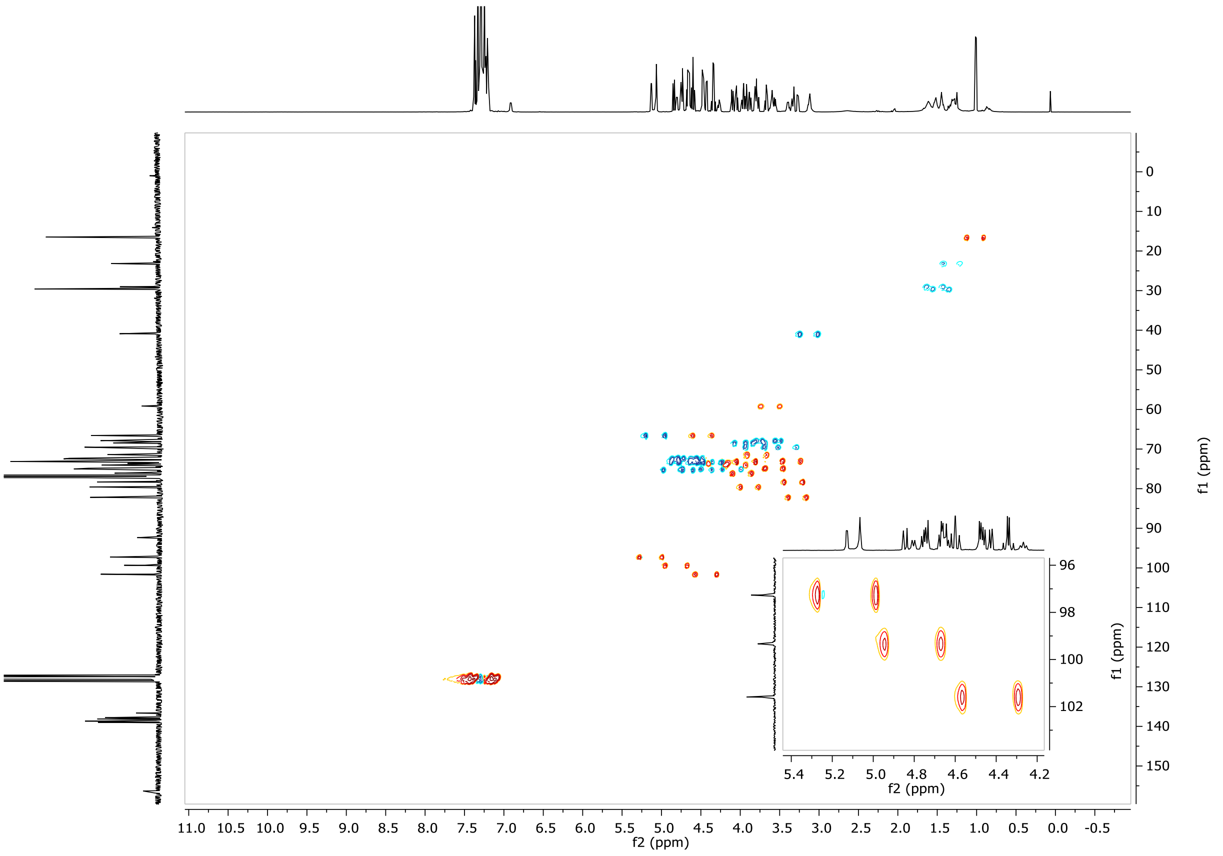

^{1}H NMR: **21**

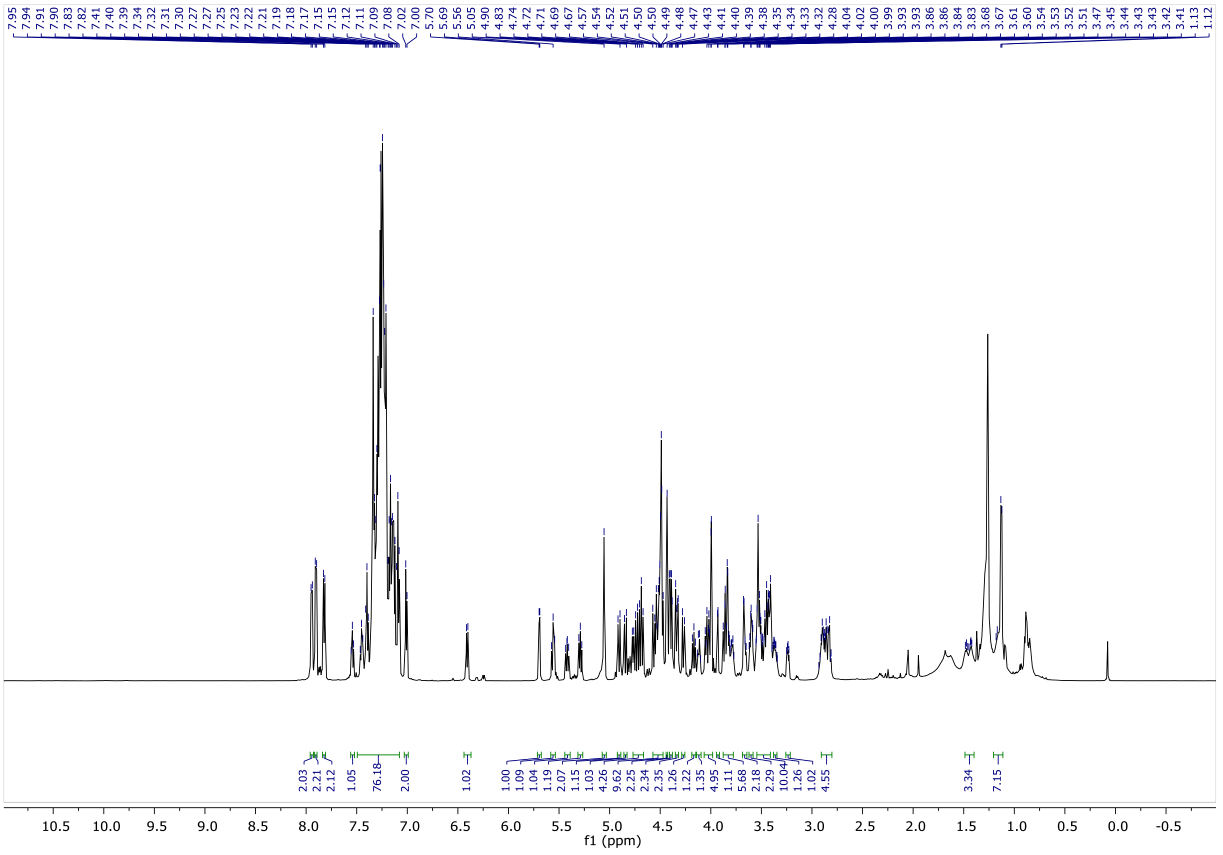

^{13}C NMR: **21**

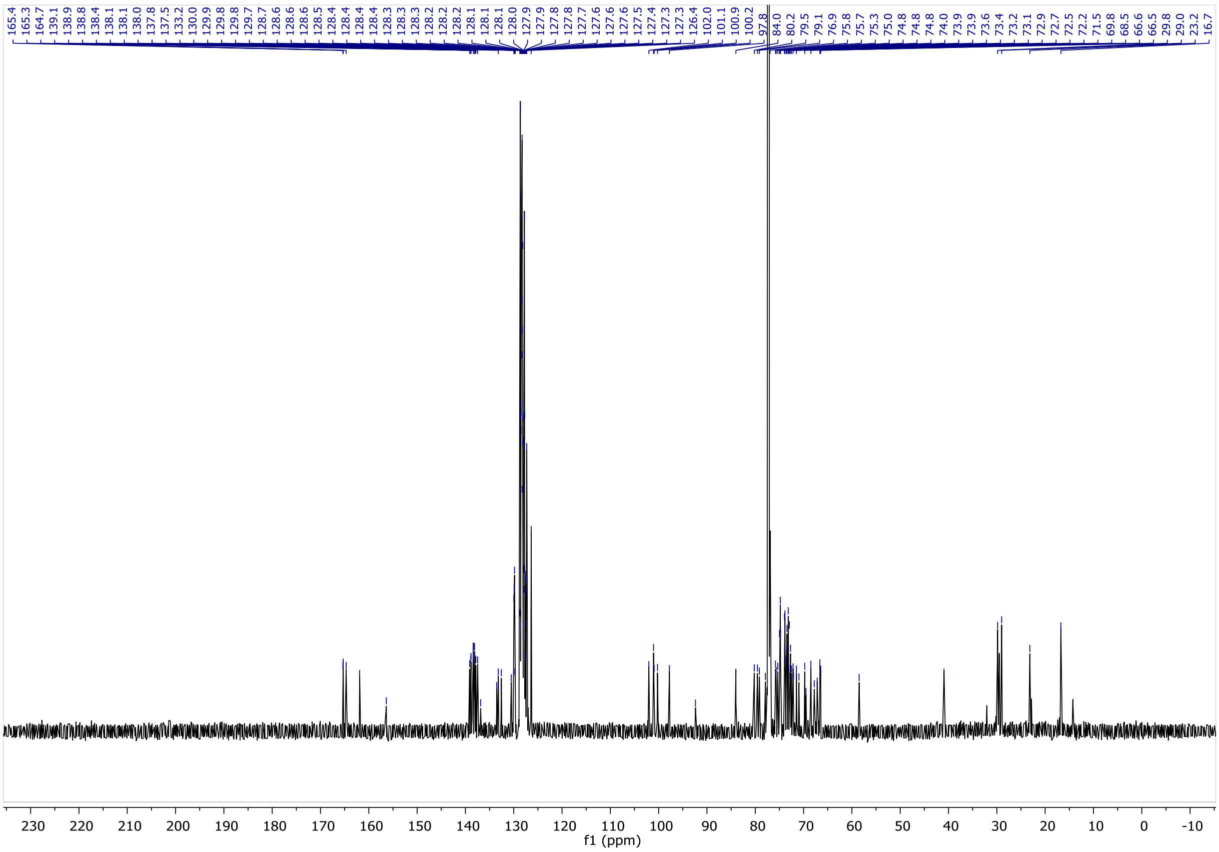

COSY: **21**

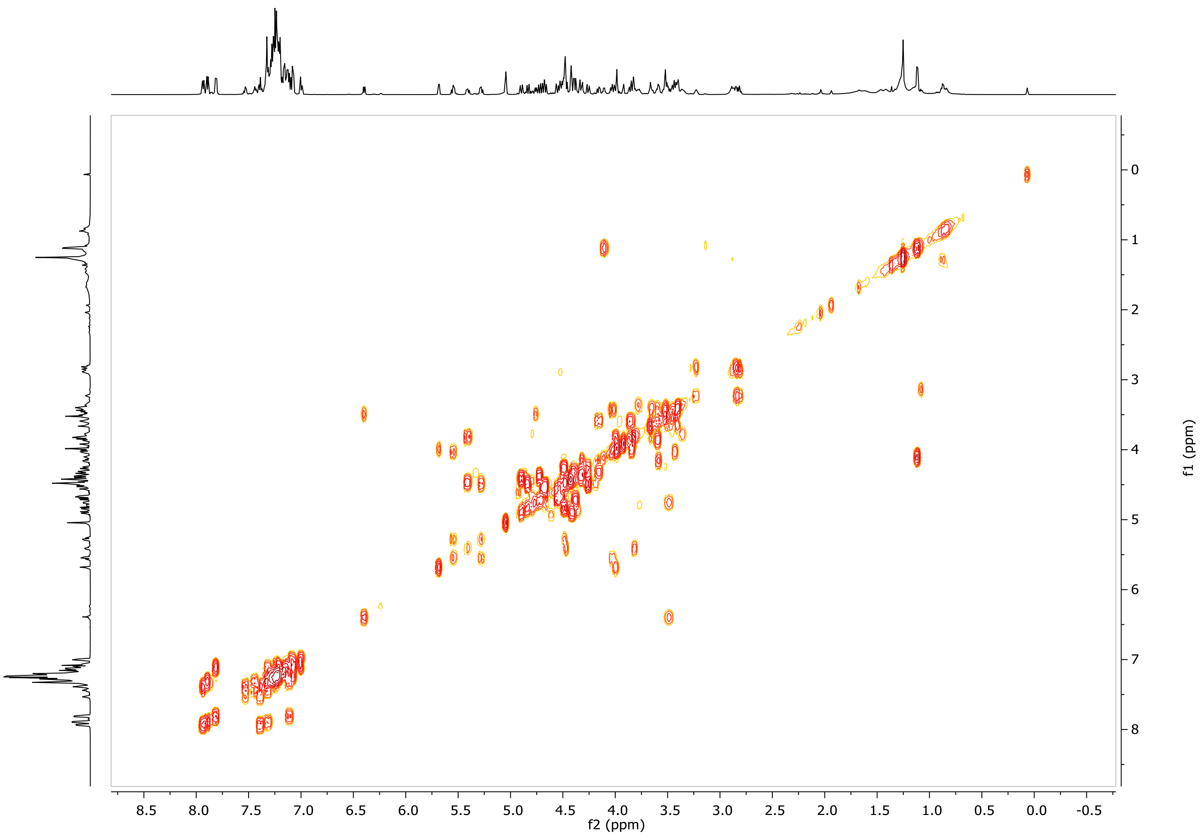

HSQC: **21**

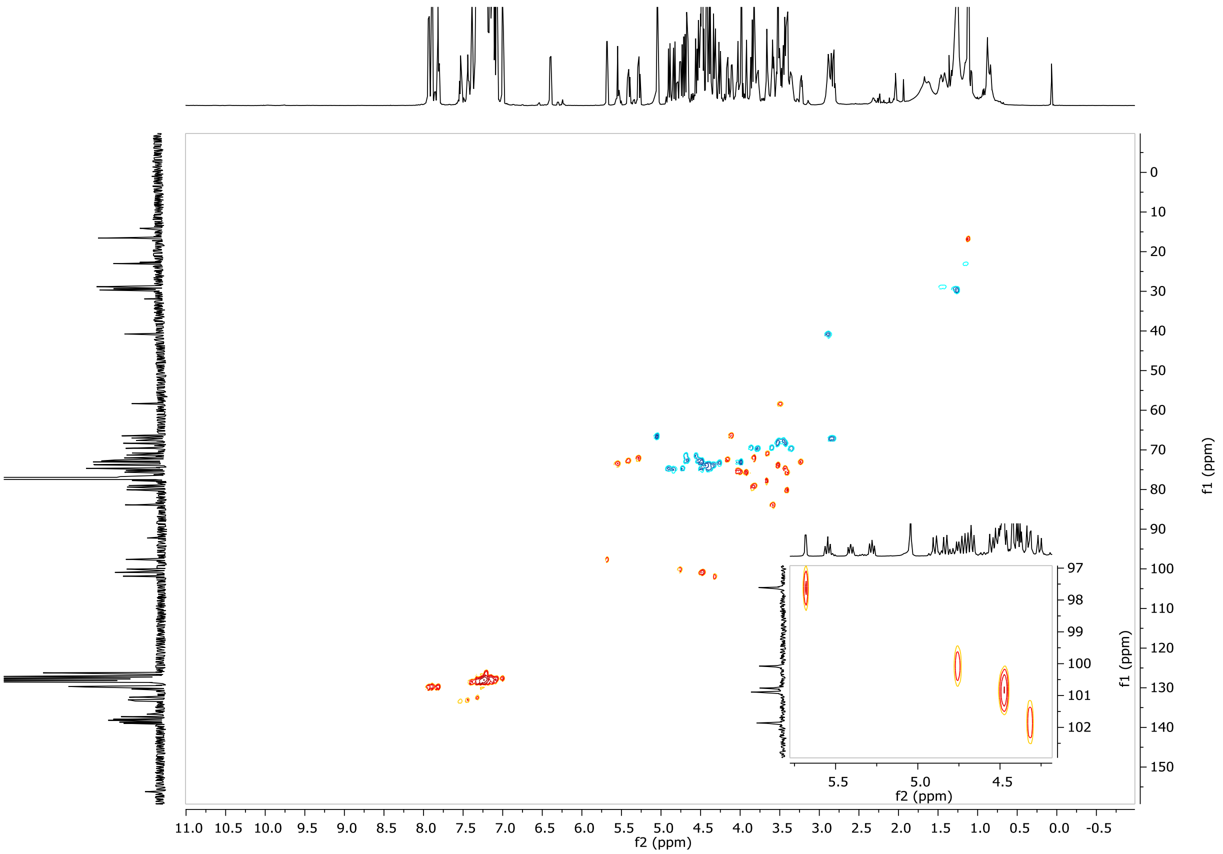

Coupled HSQC: **21**

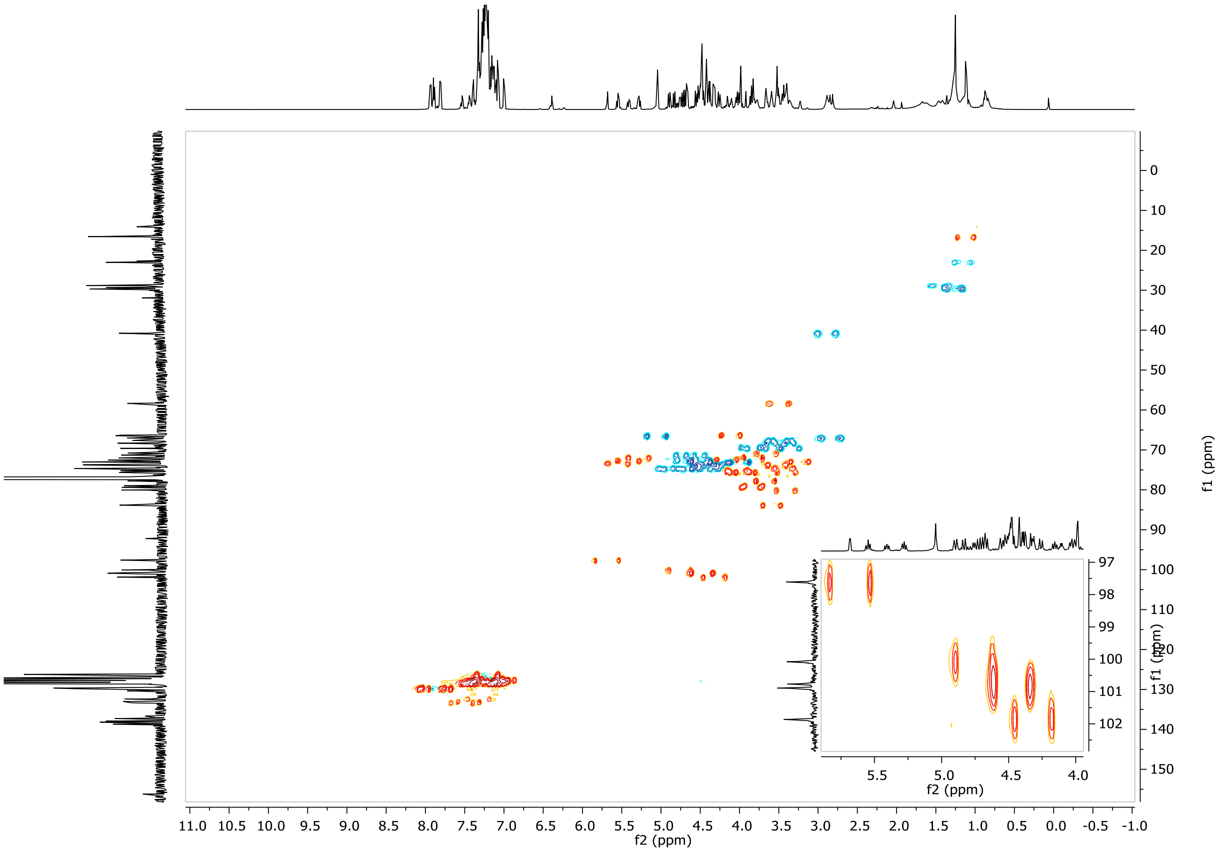

^1H NMR: **22**

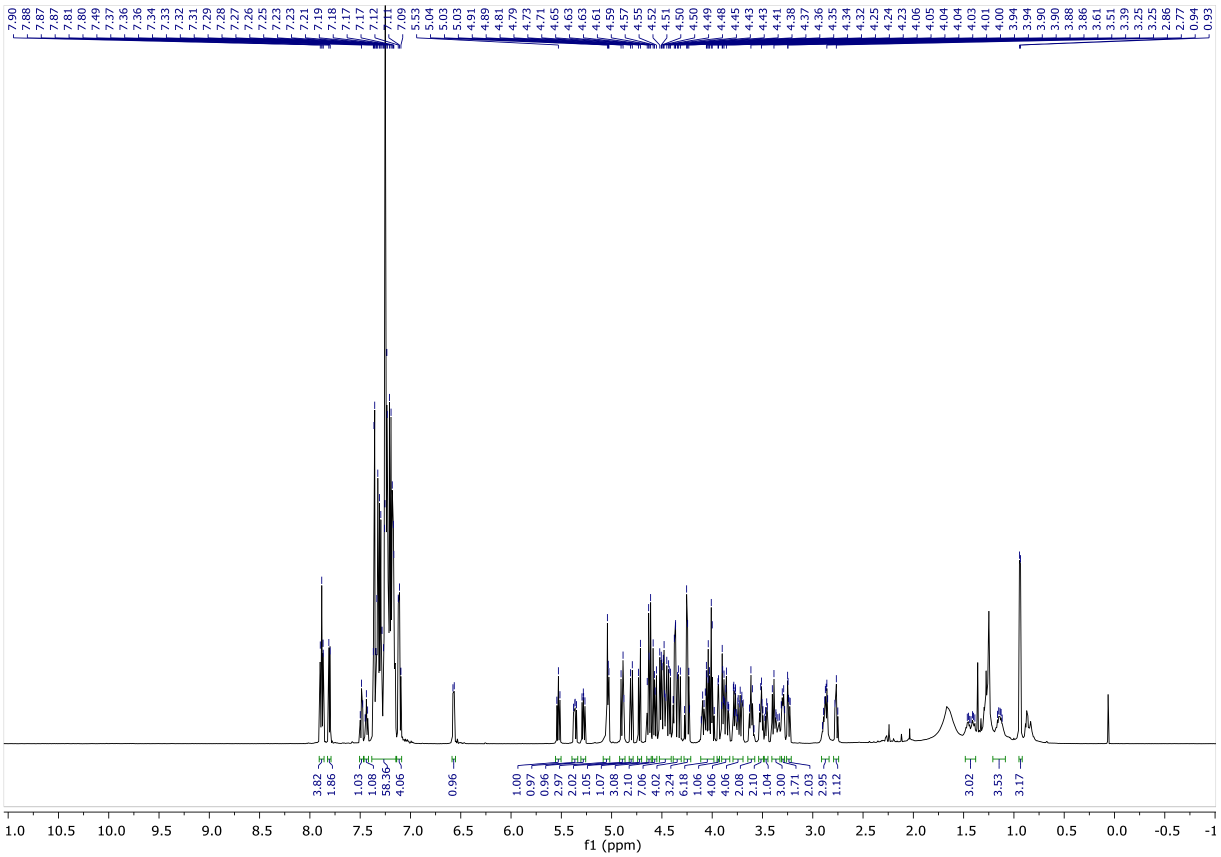

^{13}C NMR: **22**

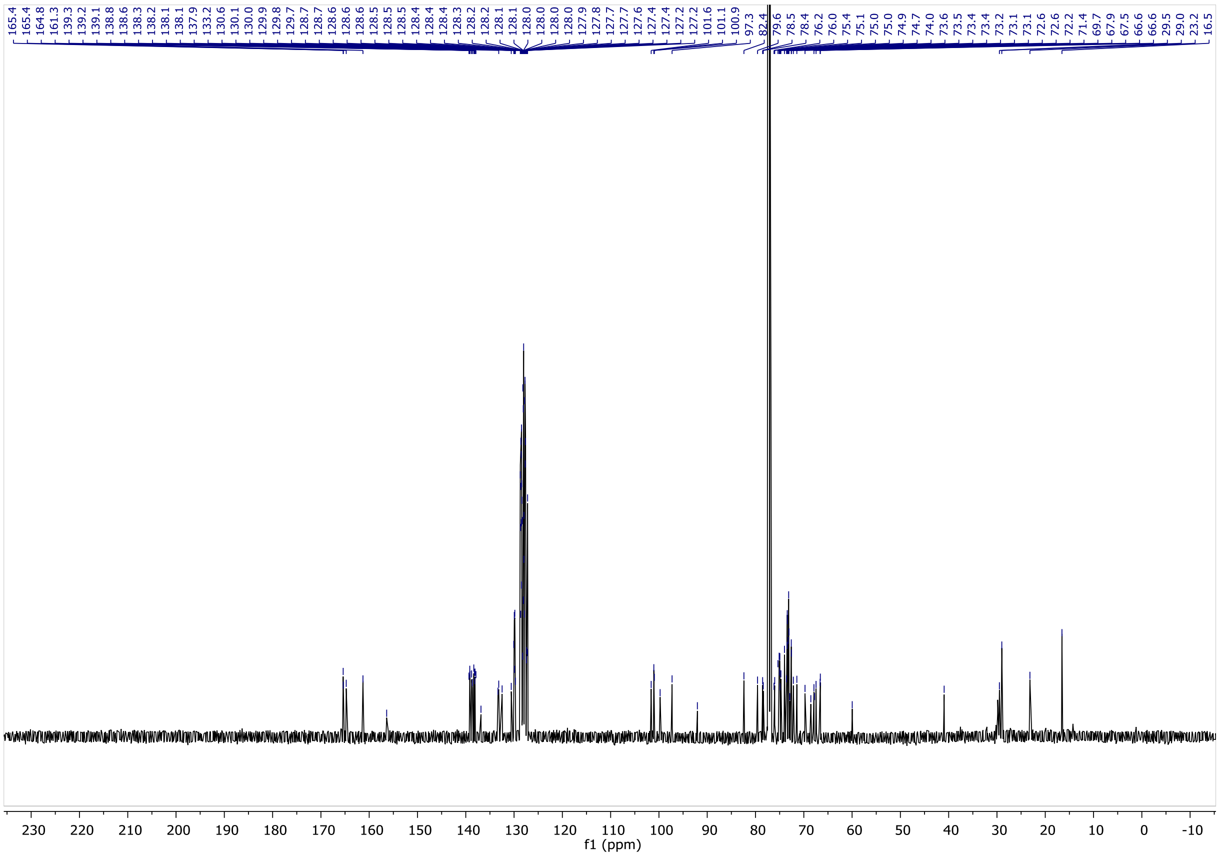

COSY: **22**

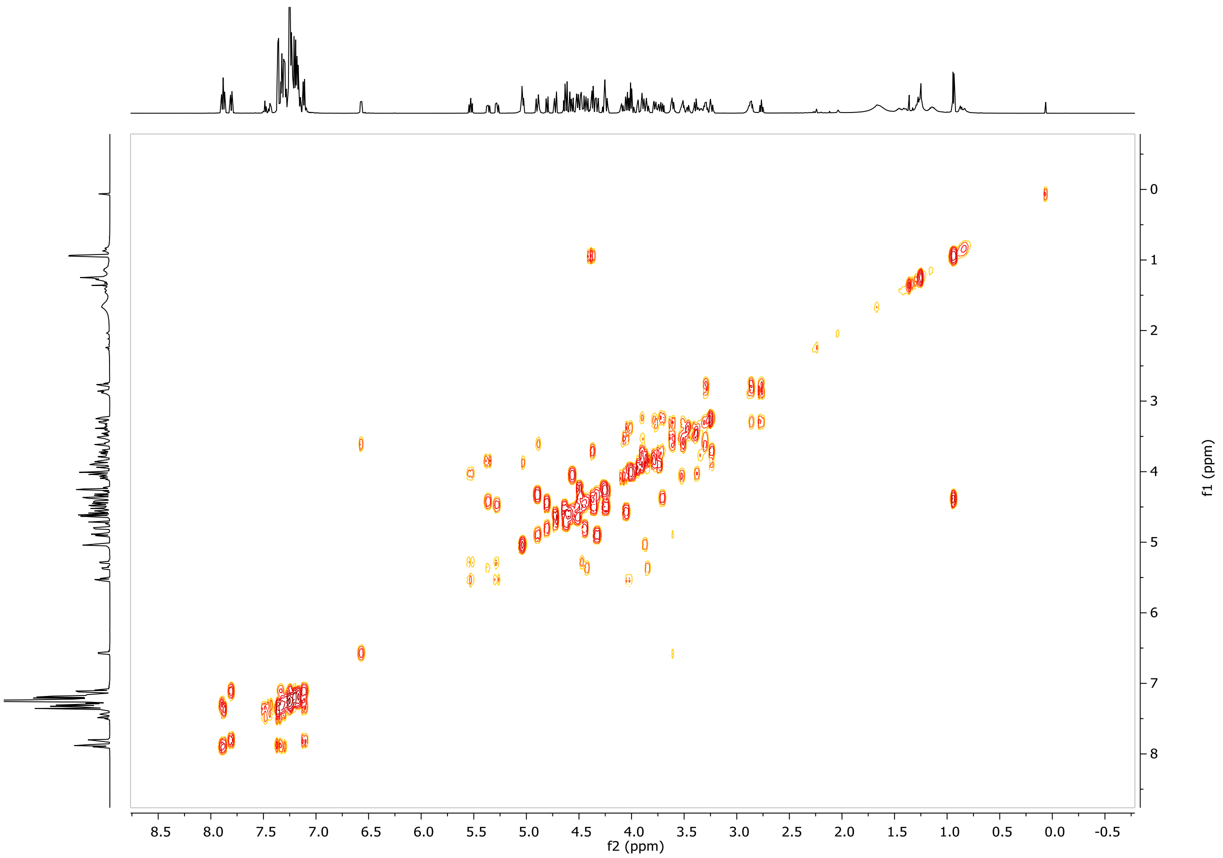

HSQC: **22**

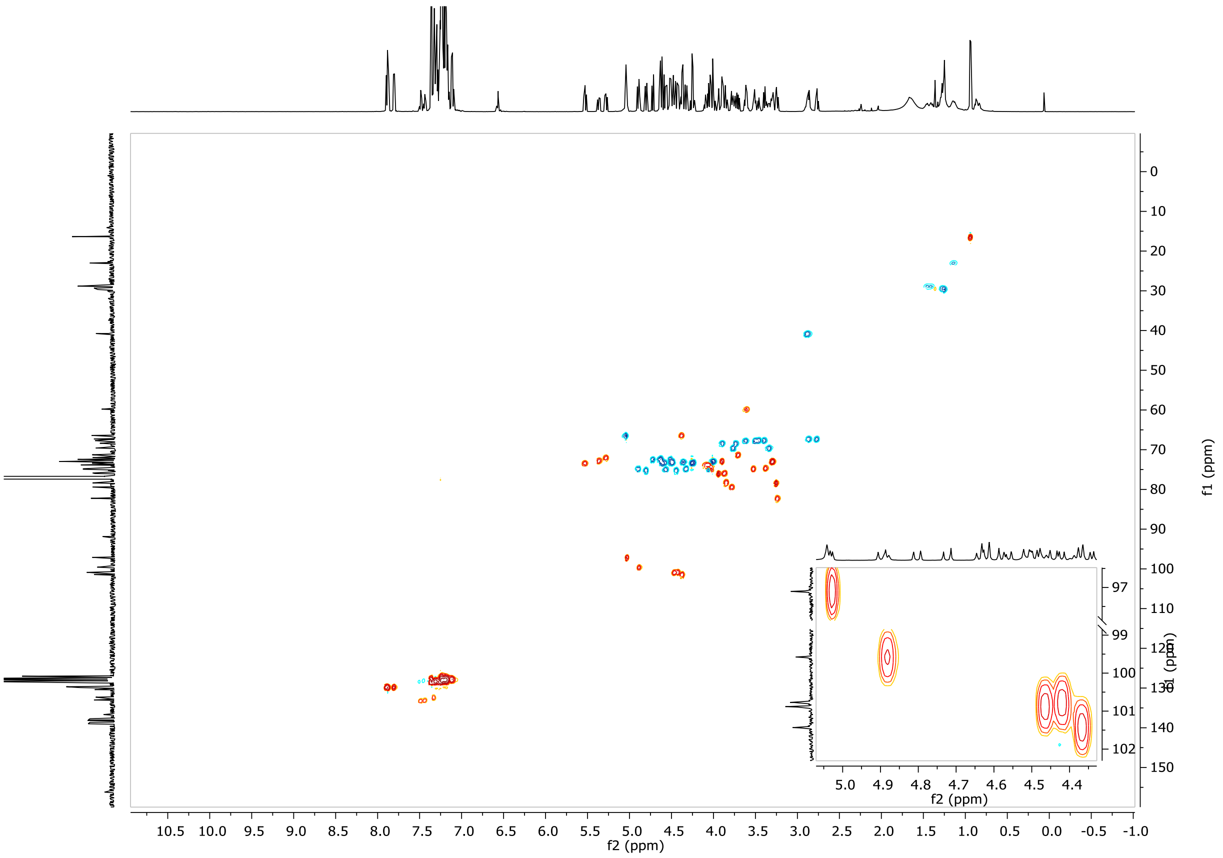

Coupled HSQC: **22**

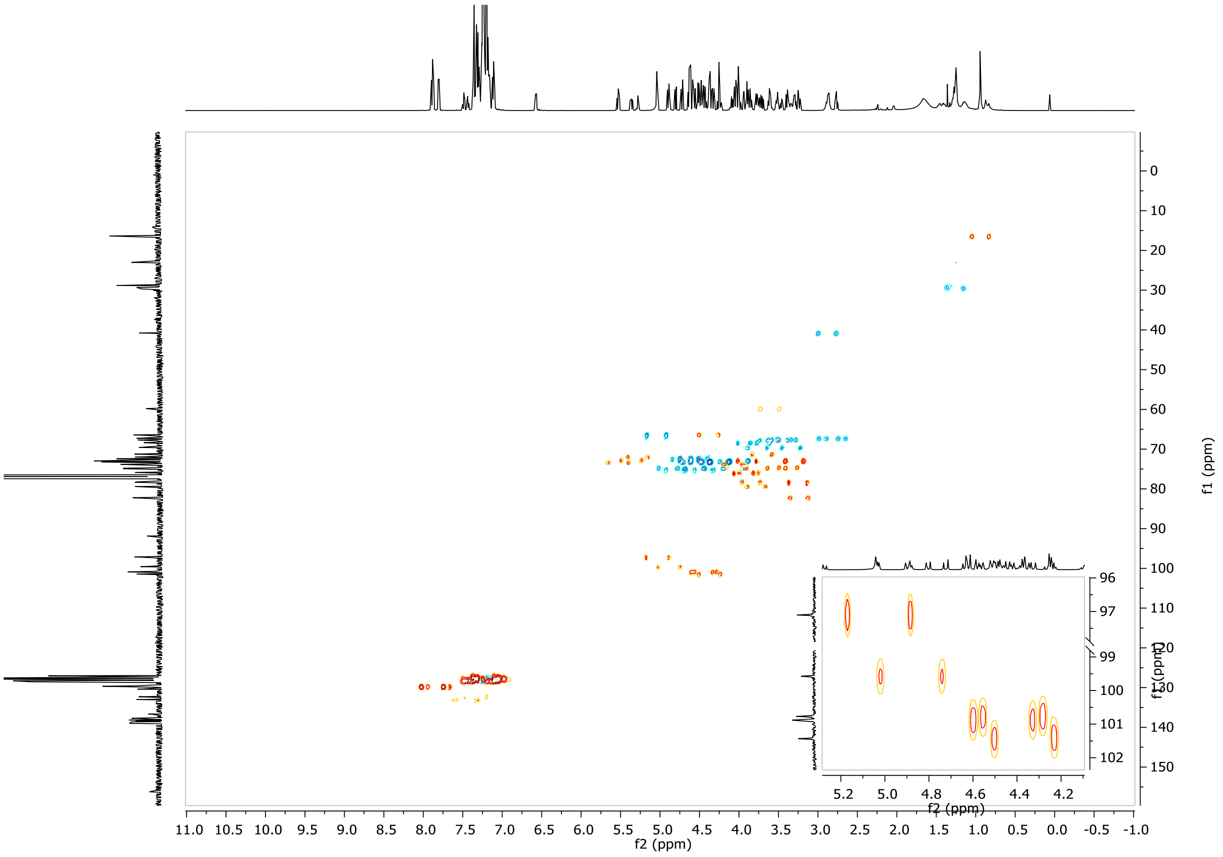

^{1}H NMR: **23**

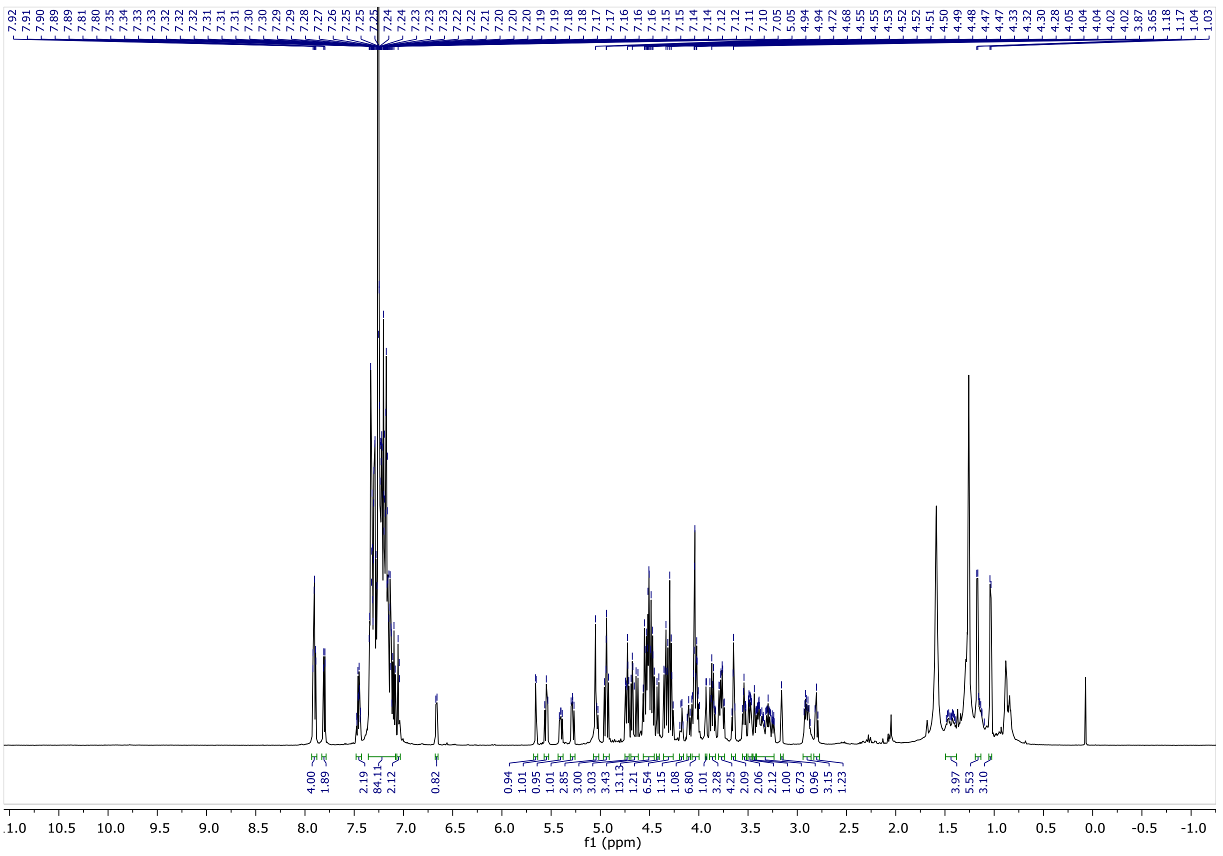

^{13}C NMR: **23**

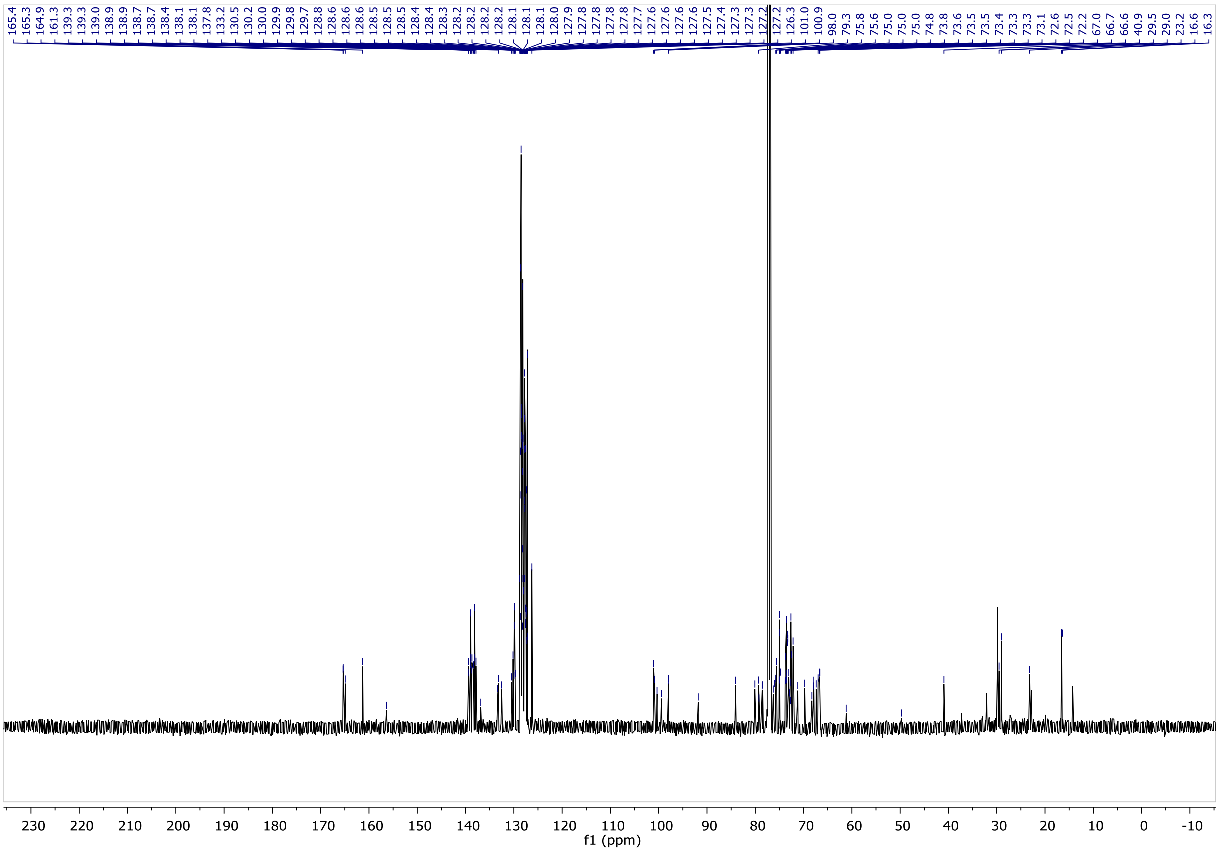

COSY: **23**

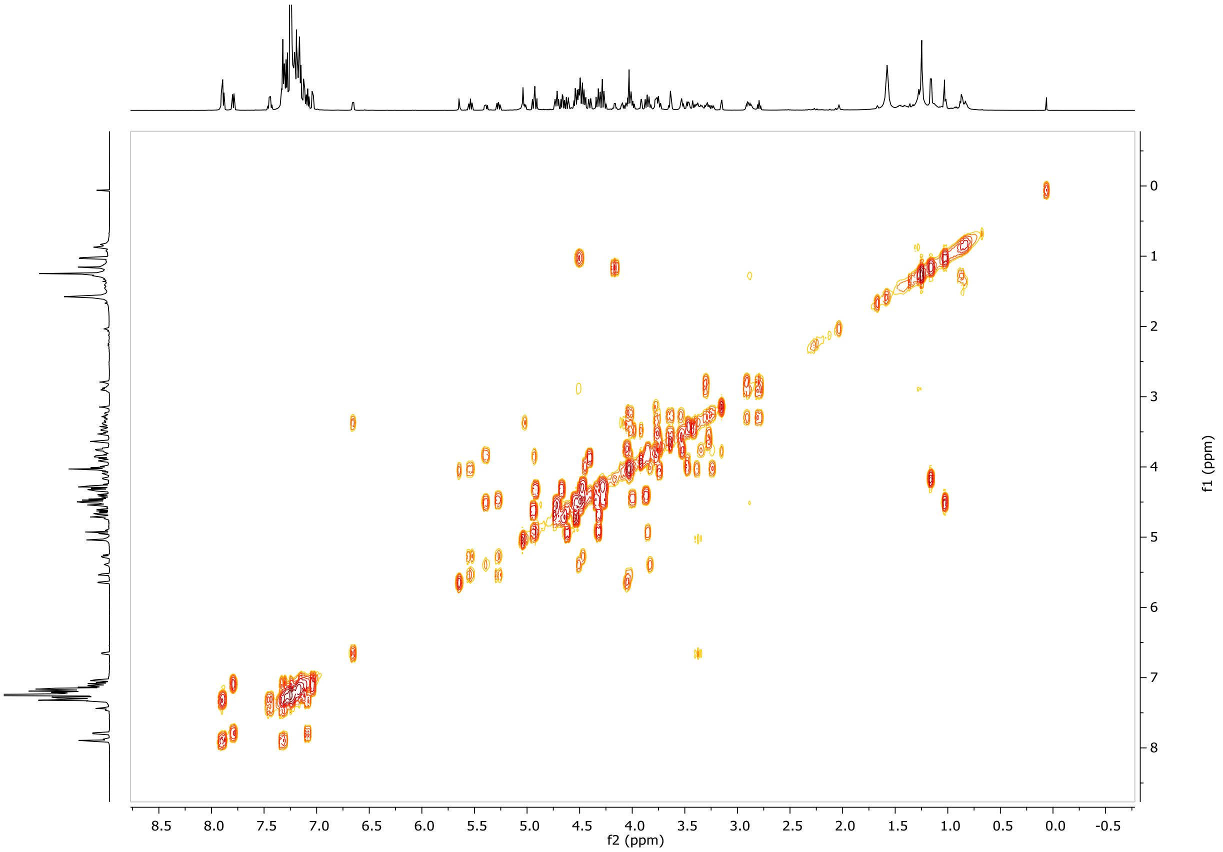

HSQC: **23**

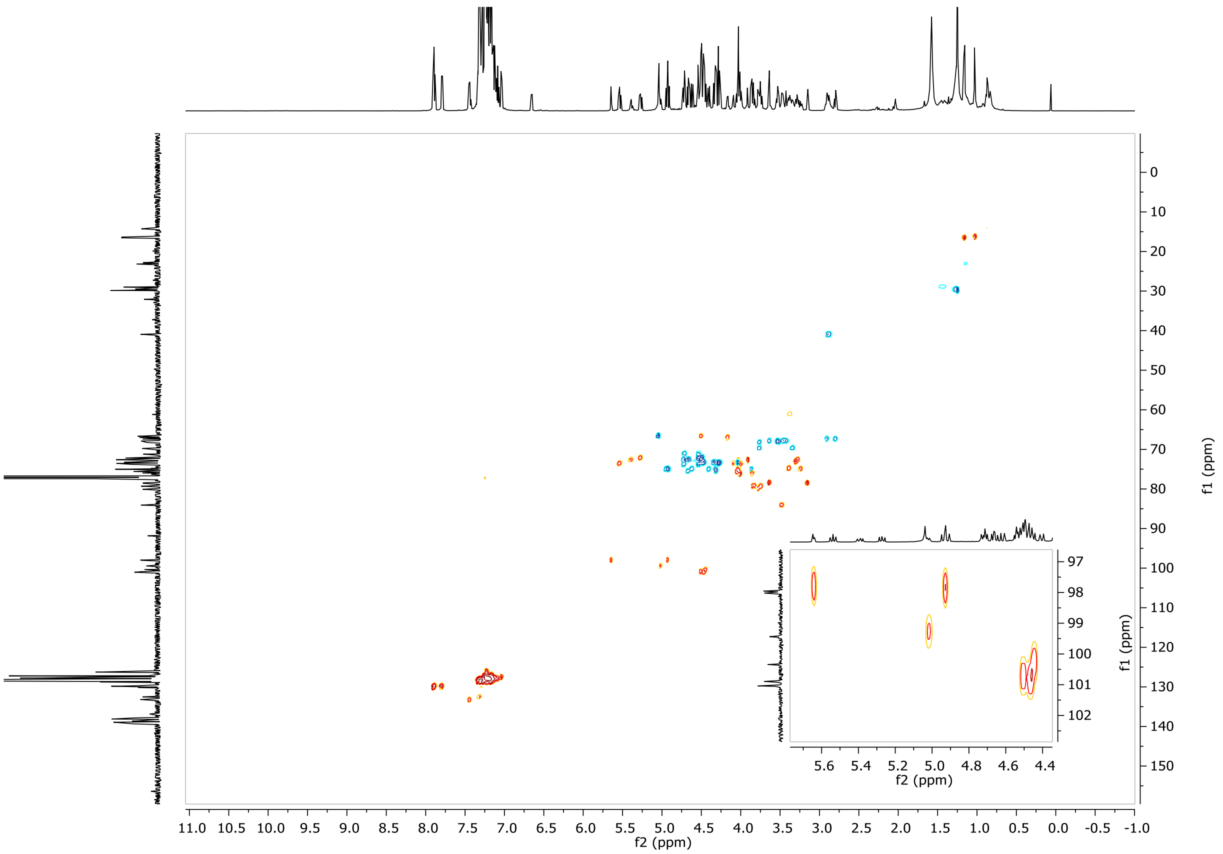

Coupled HSQC: **23**

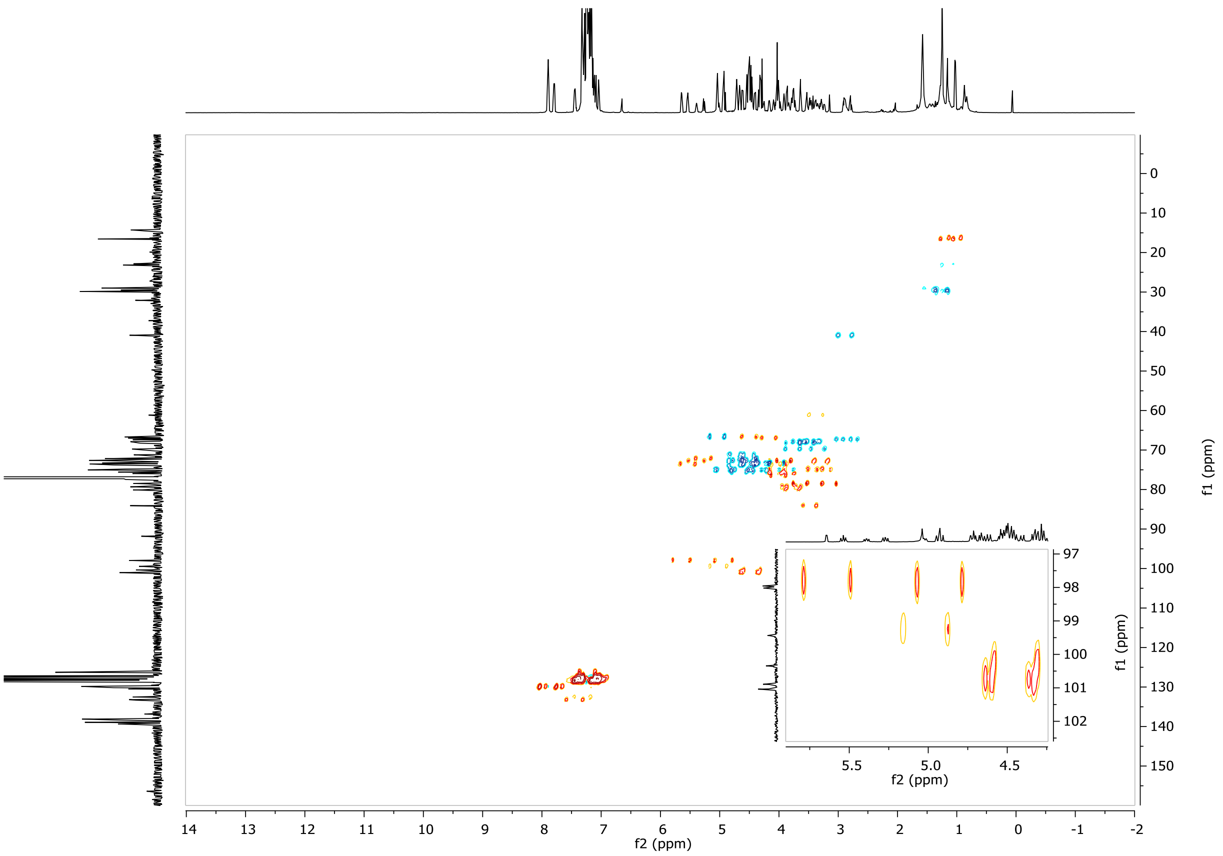

^{1}H NMR: **24**

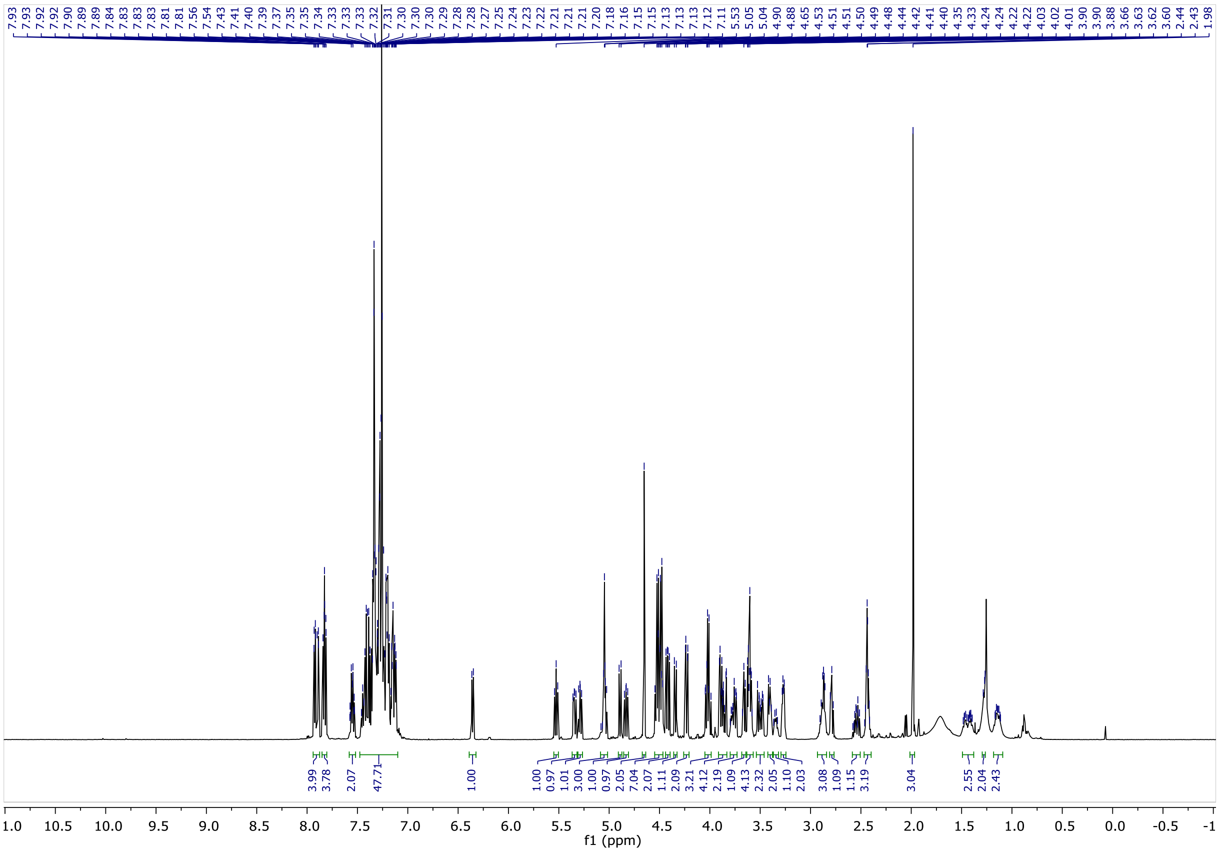

^{13}C NMR: **24**

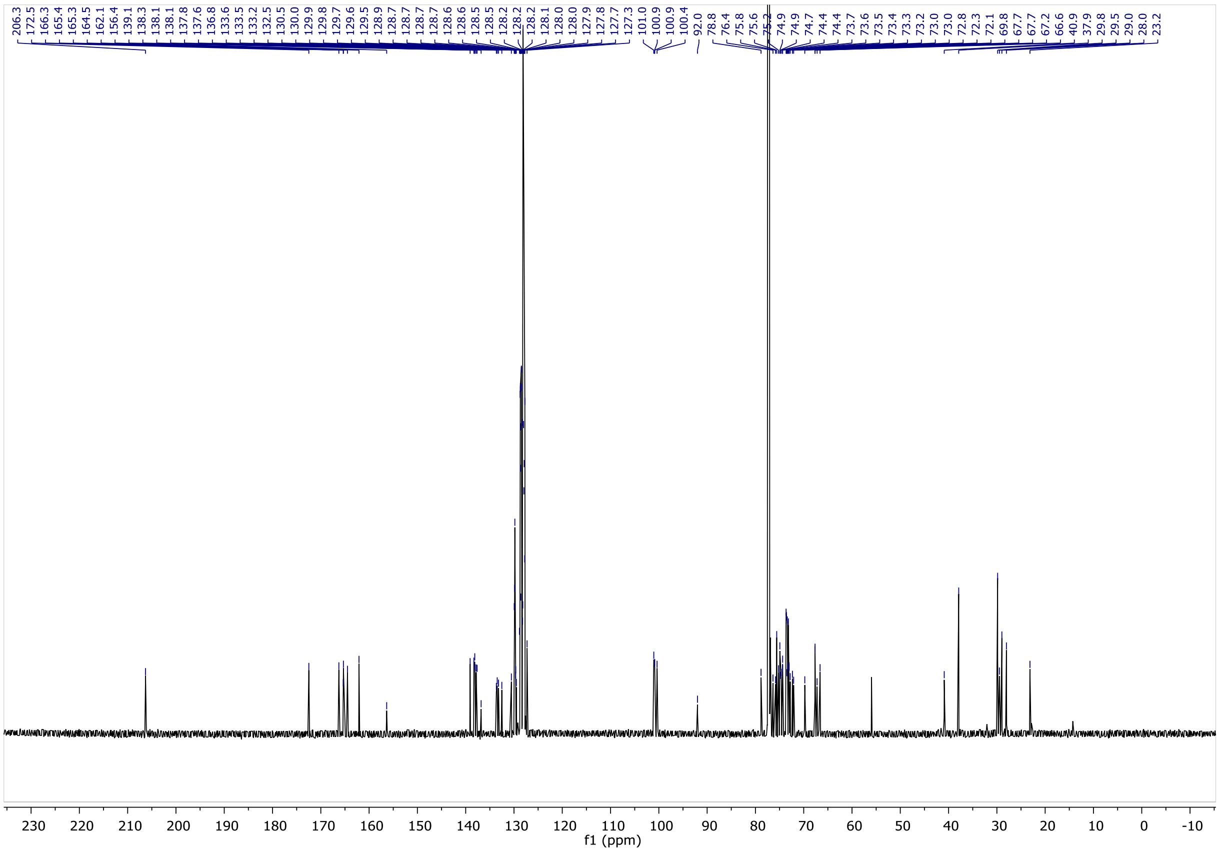

COSY: **24**

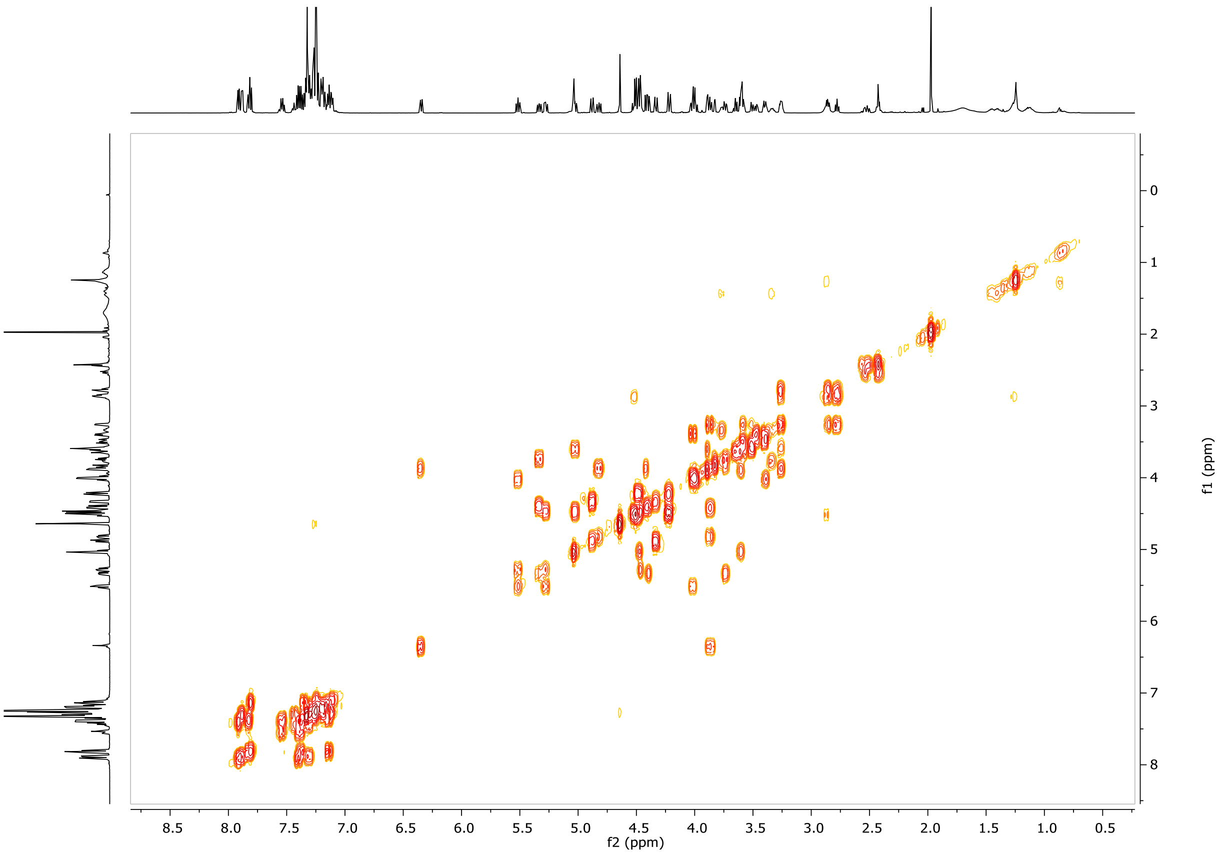

HSQC: **24**

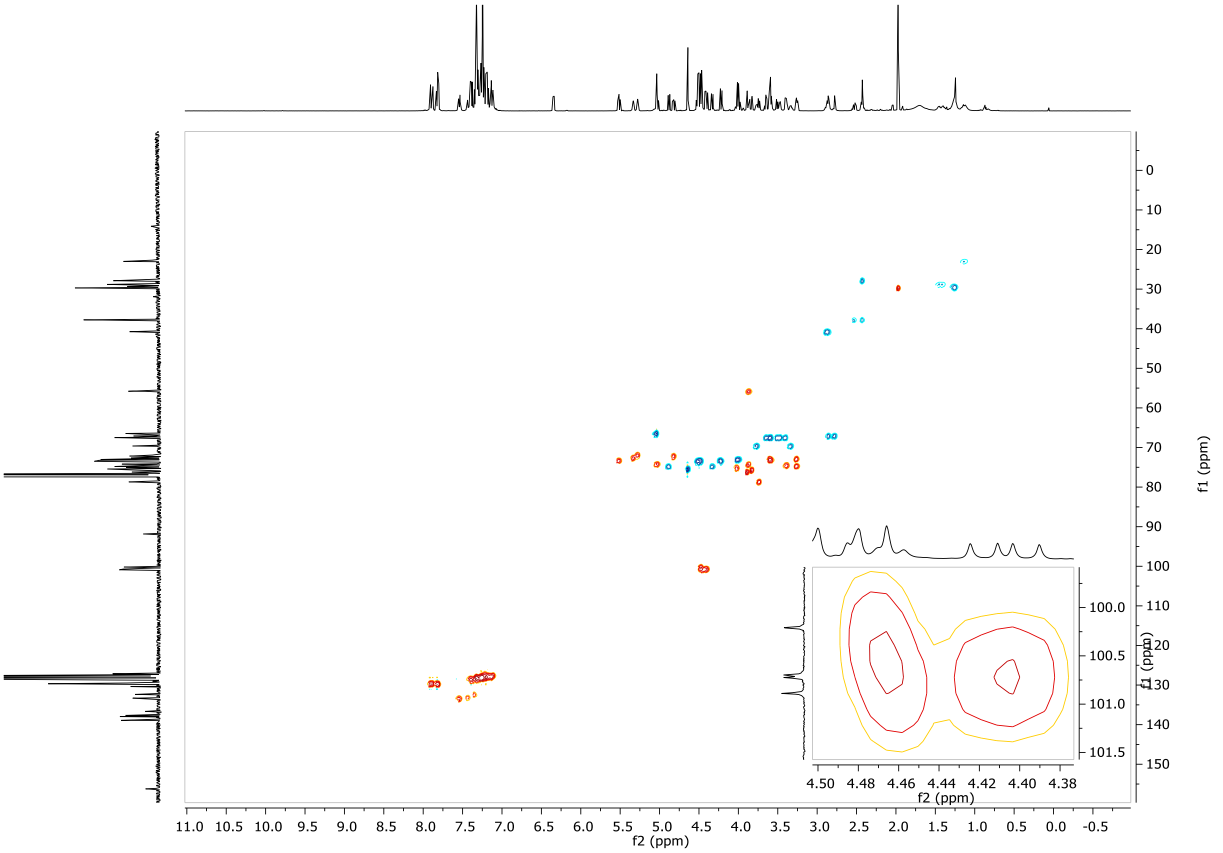

Coupled HSQC: **24**

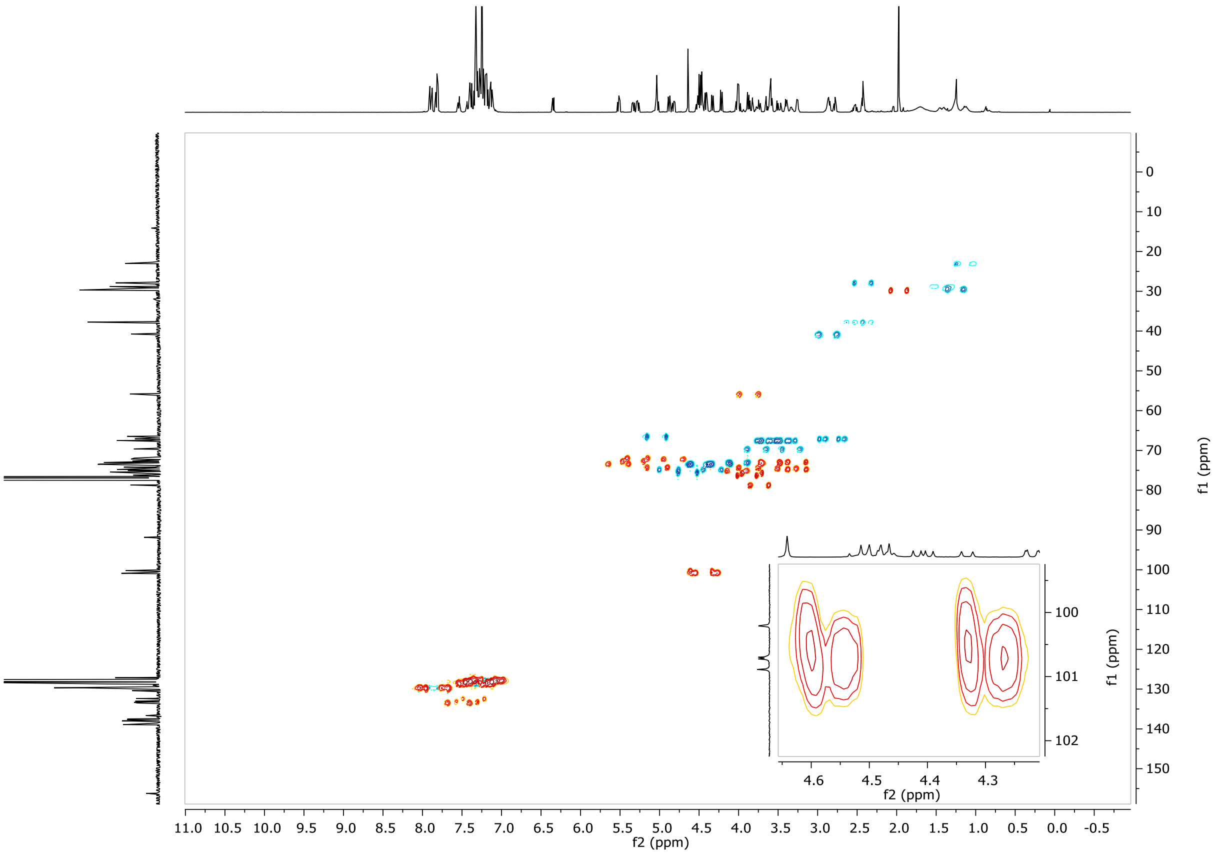

^{1}H NMR: **25**

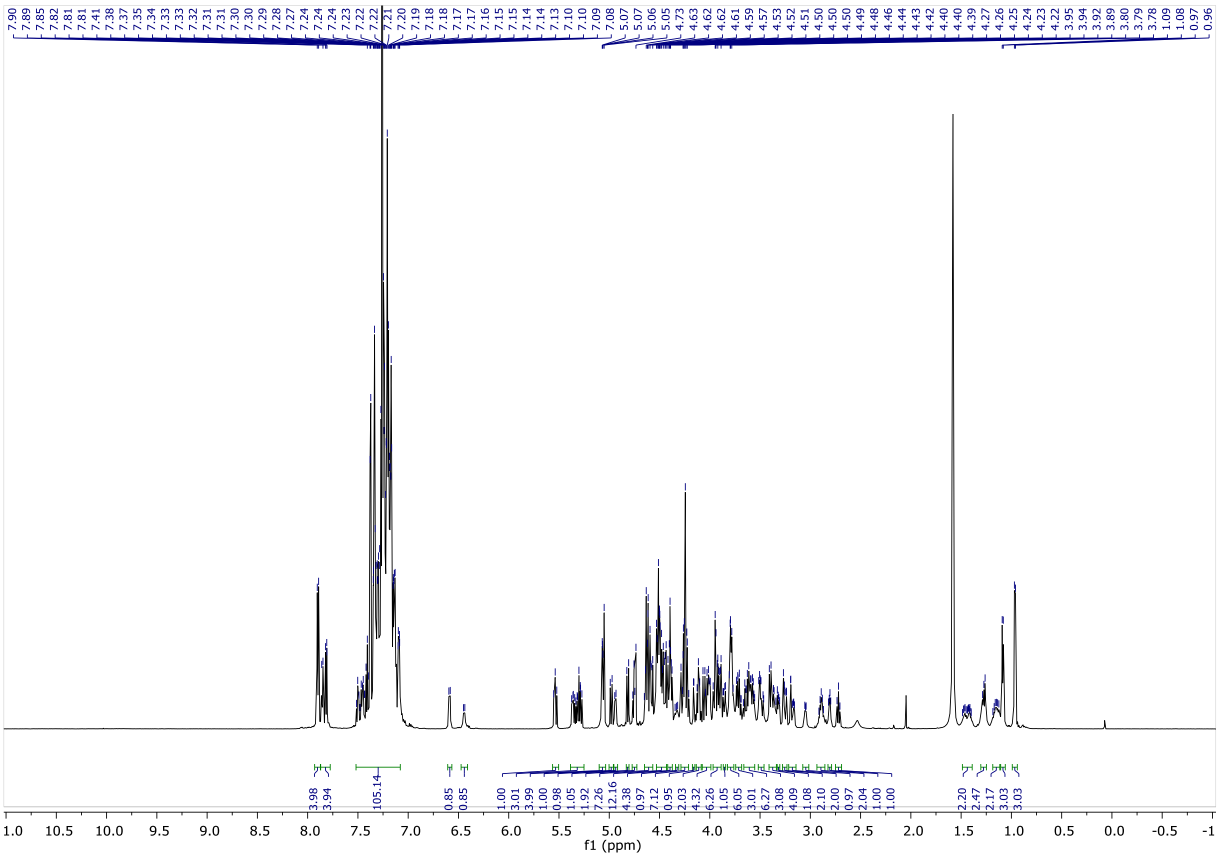

^{13}C NMR: **25**

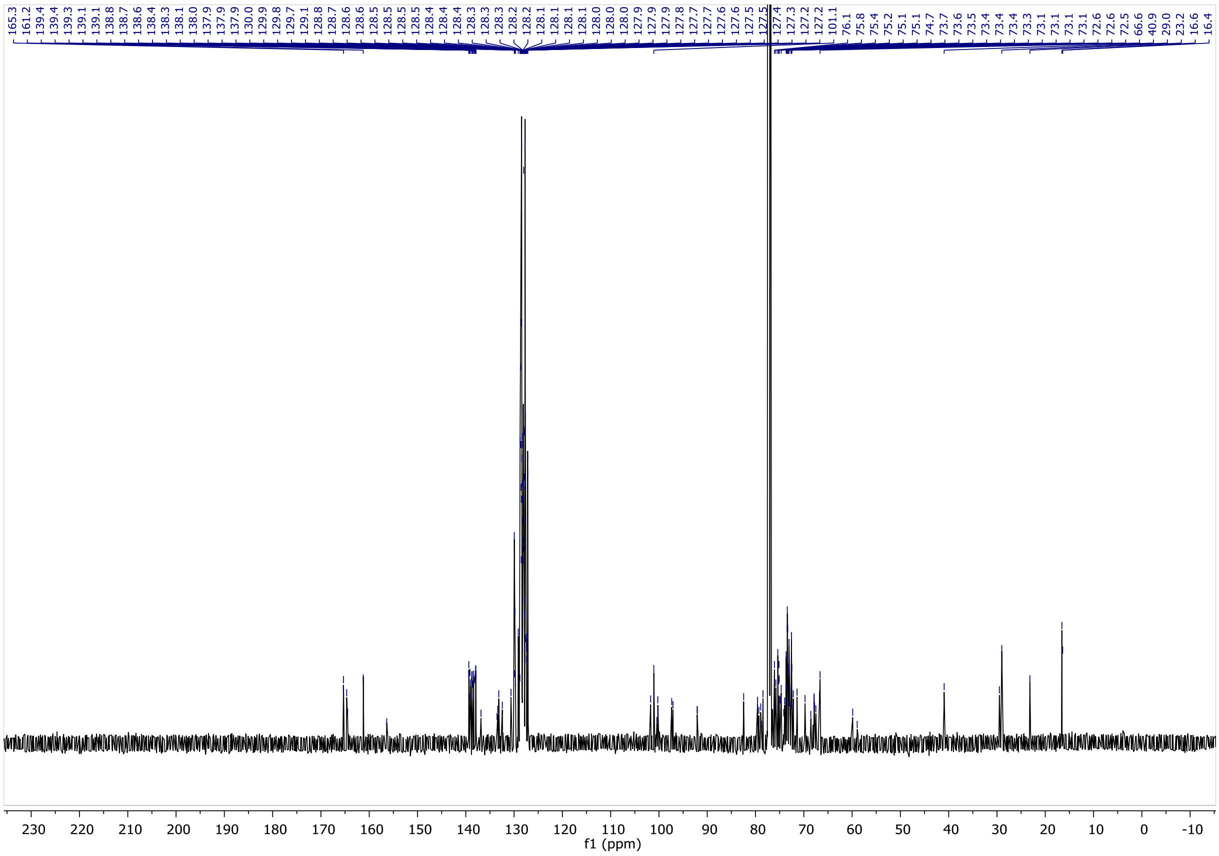

COSY: **25**

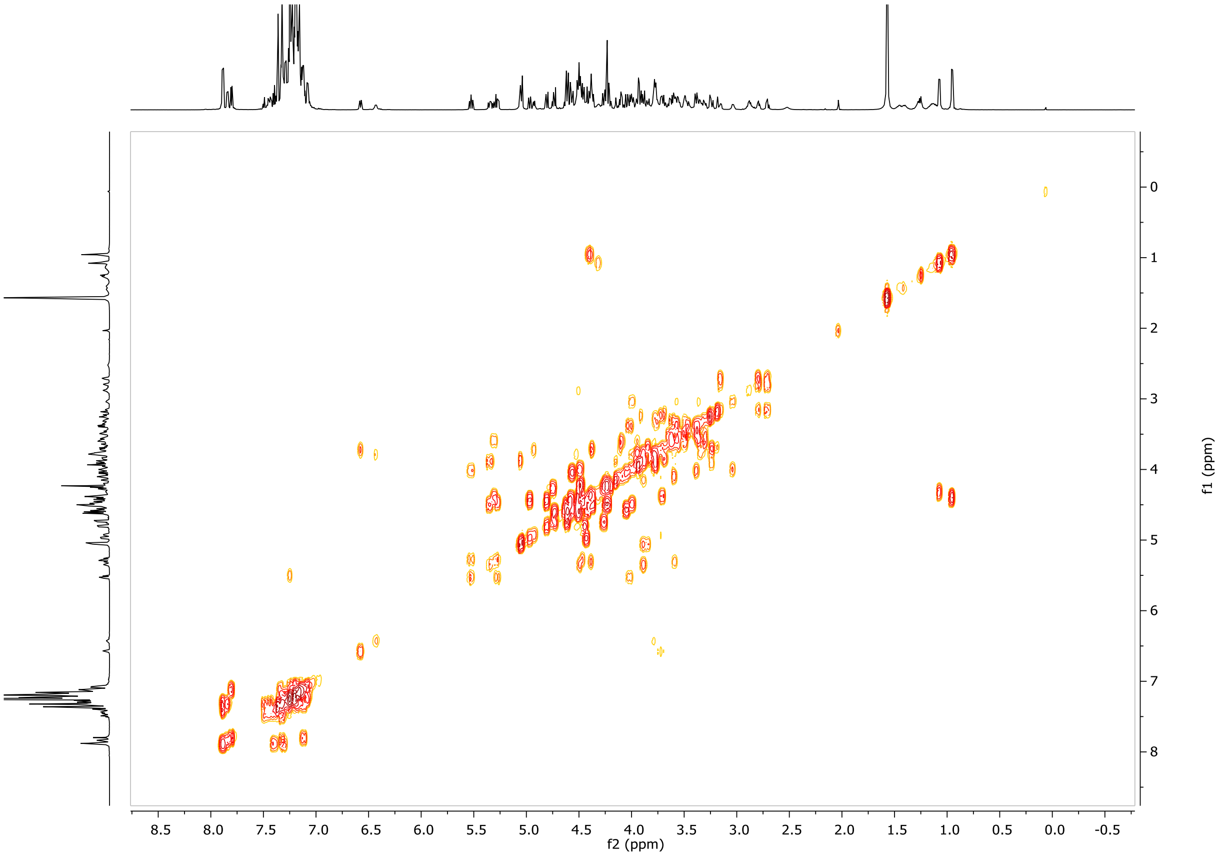

HSQC: **25**

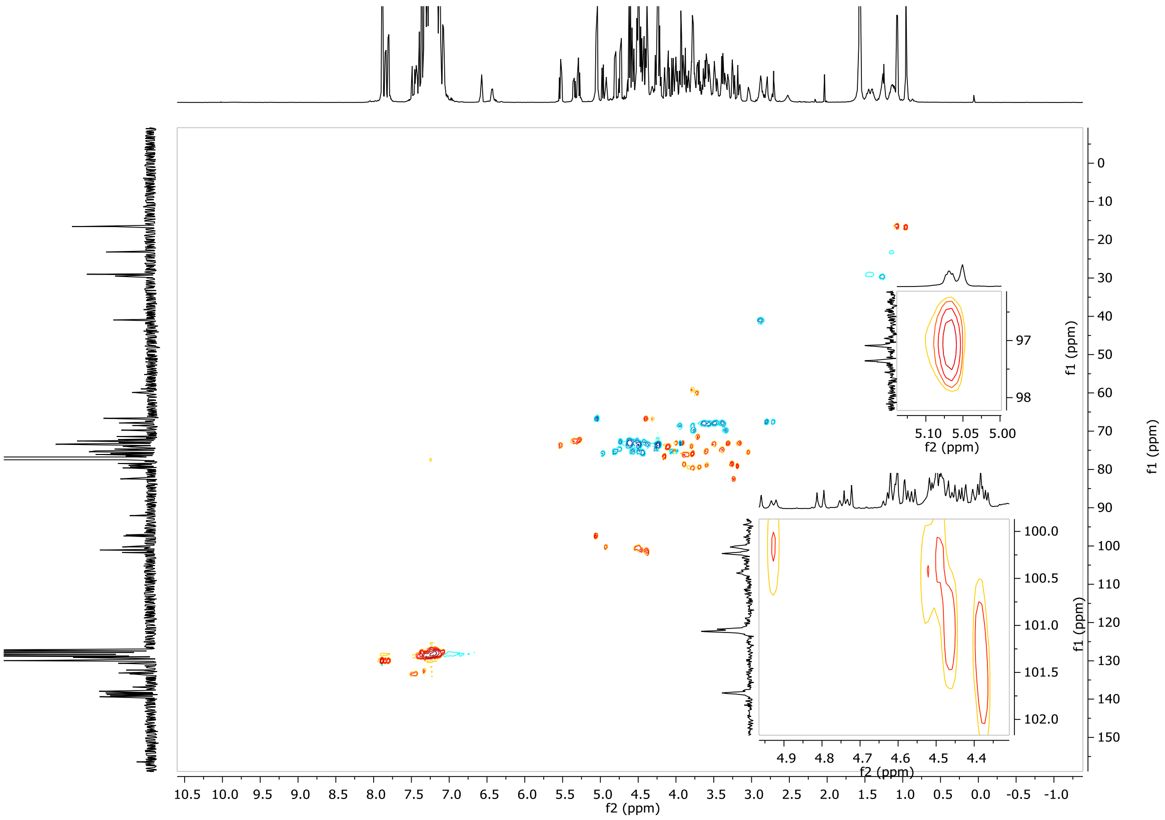

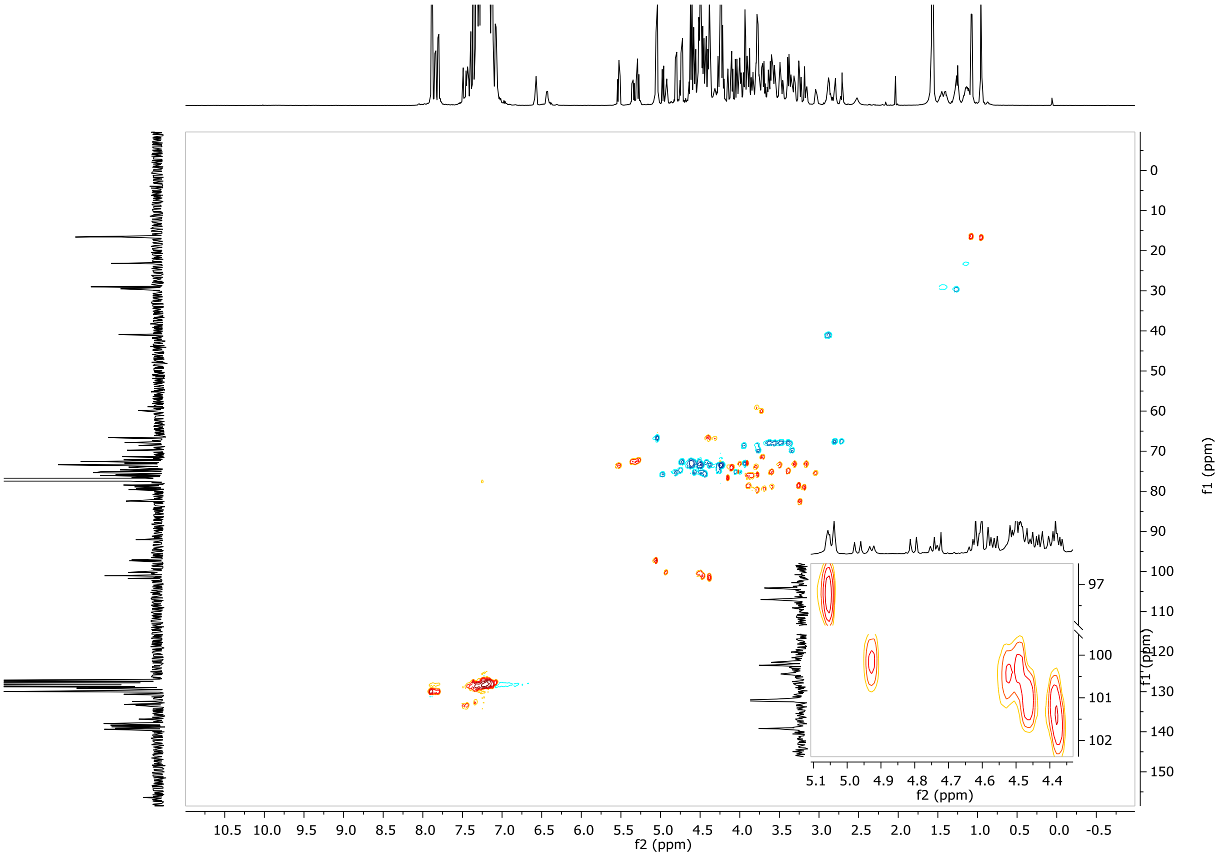

Coupled HSQC: **25**

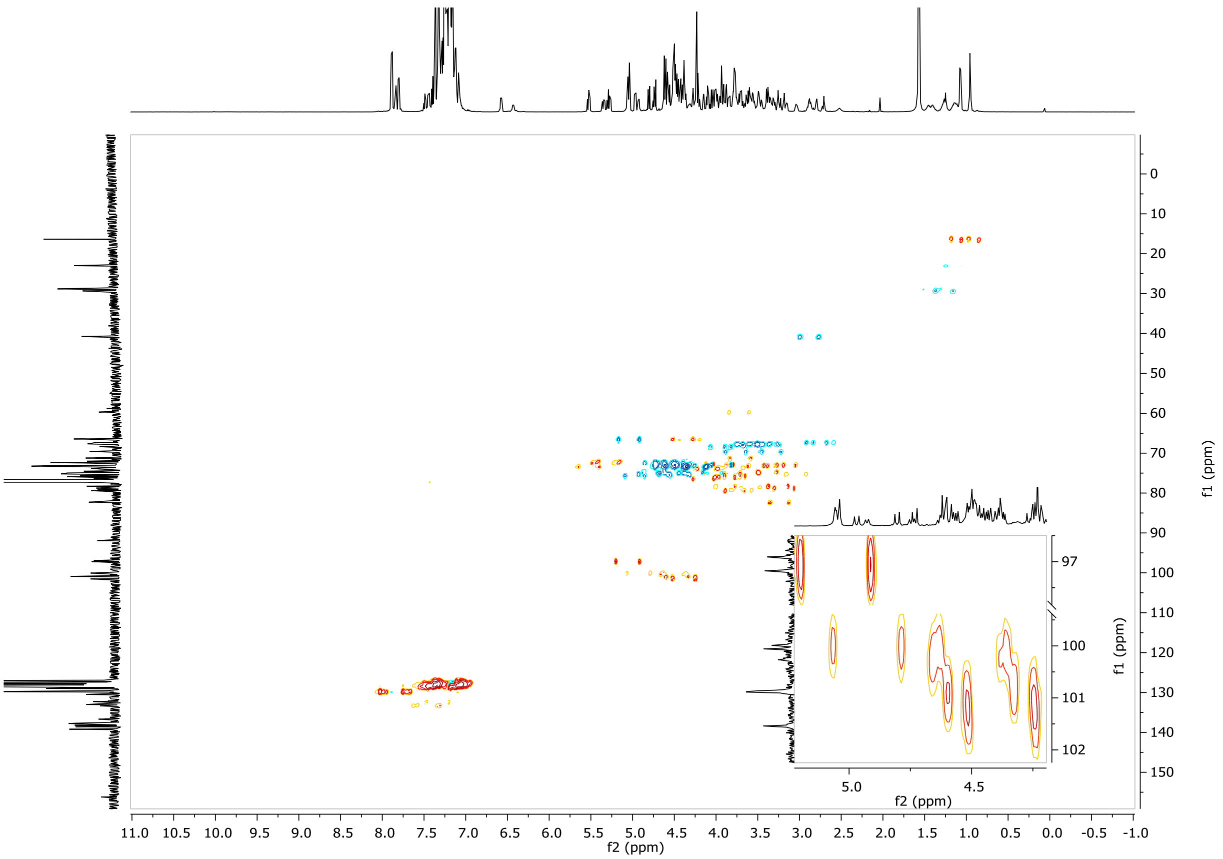

^{1}H NMR: **26**

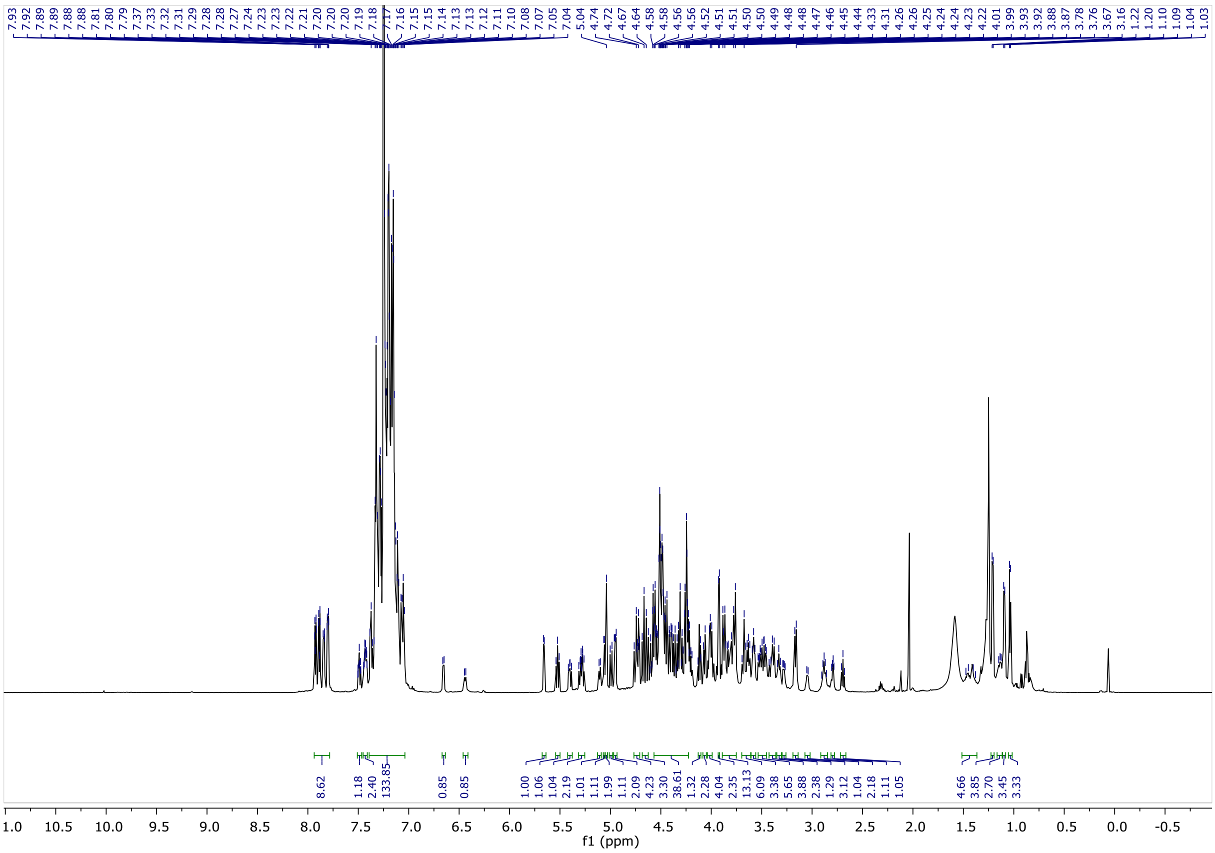

^{13}C NMR: **26**

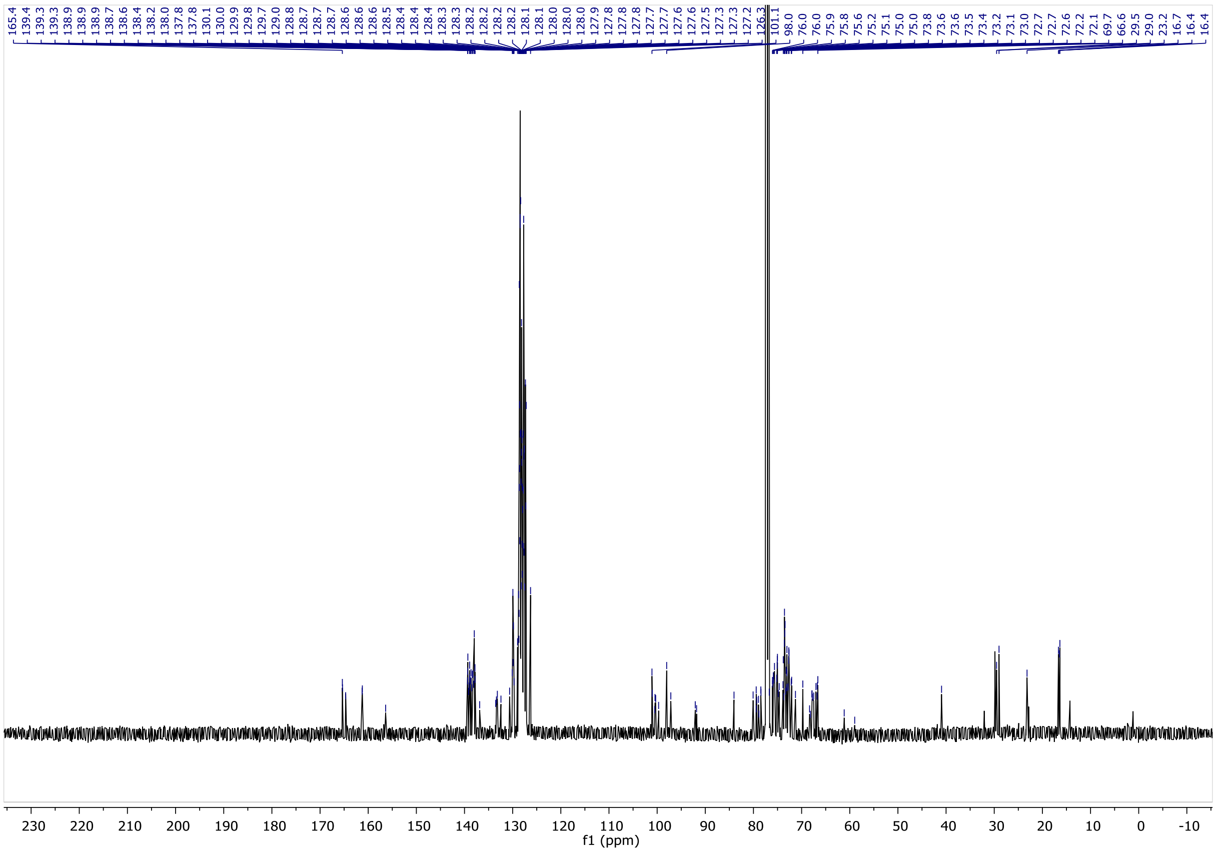

COSY: **26**

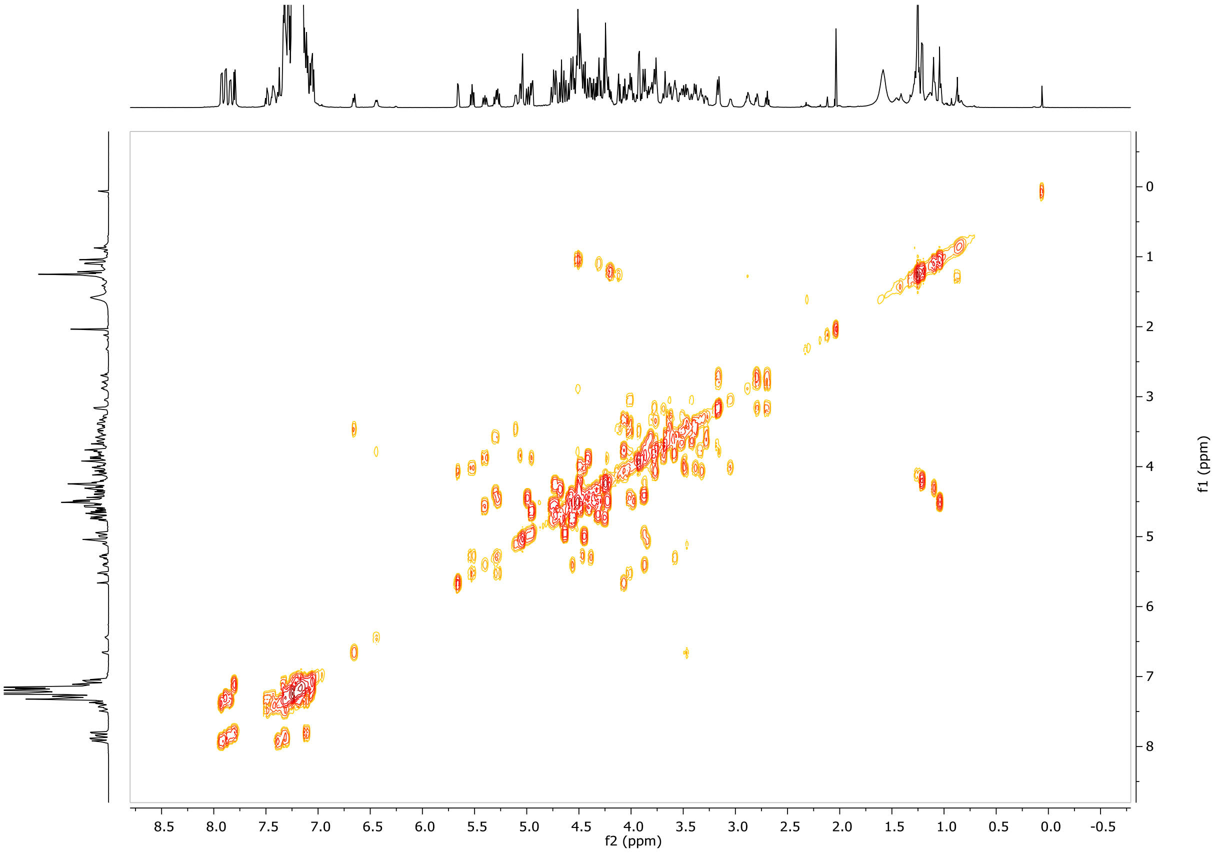

HSQC: **26**

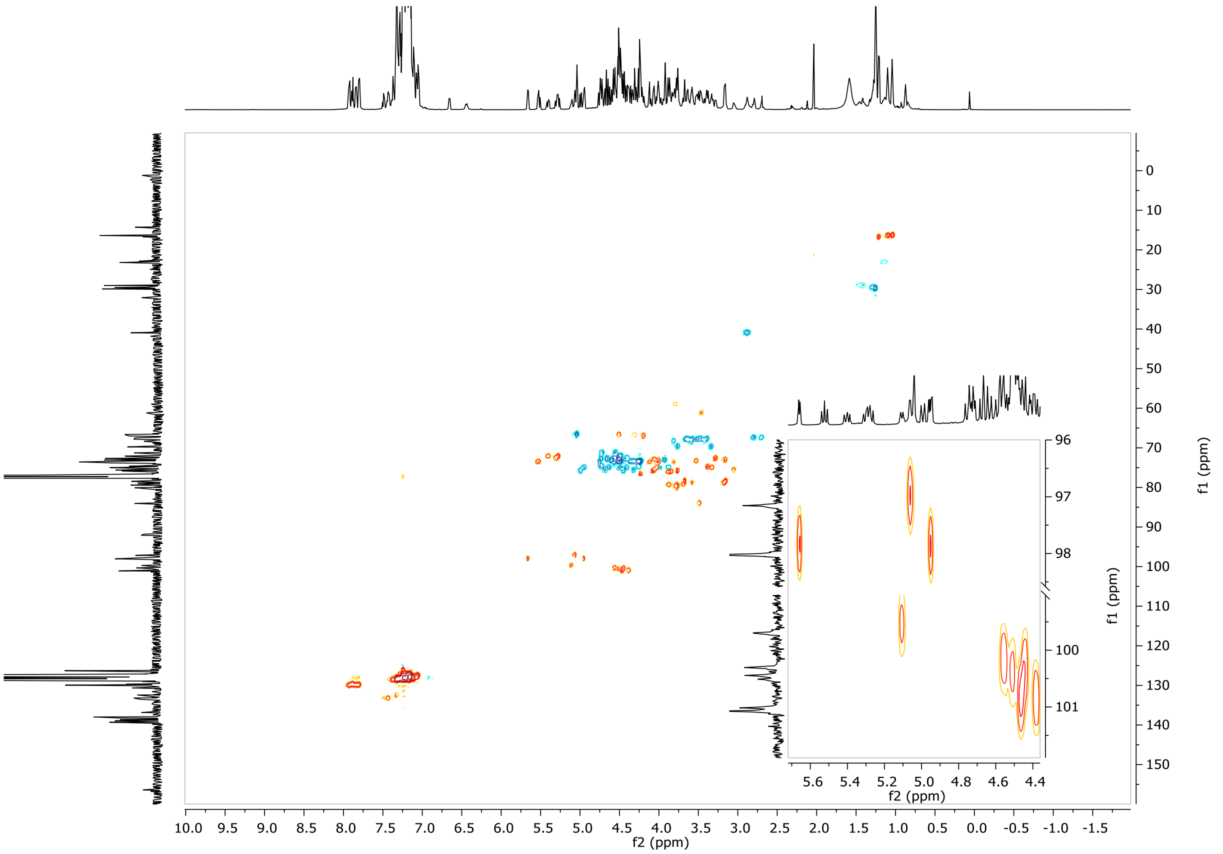

Coupled HSQC: **26**

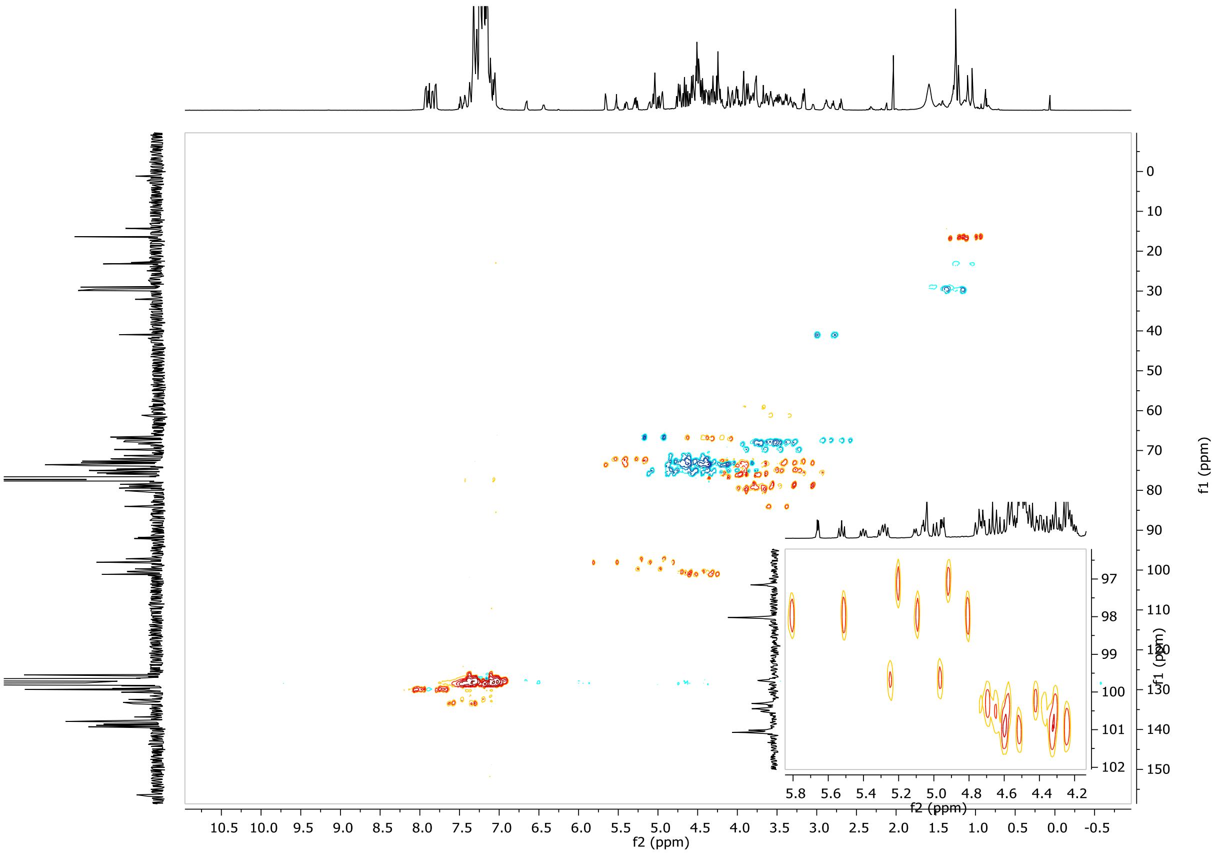

^{1}H NMR: **27**

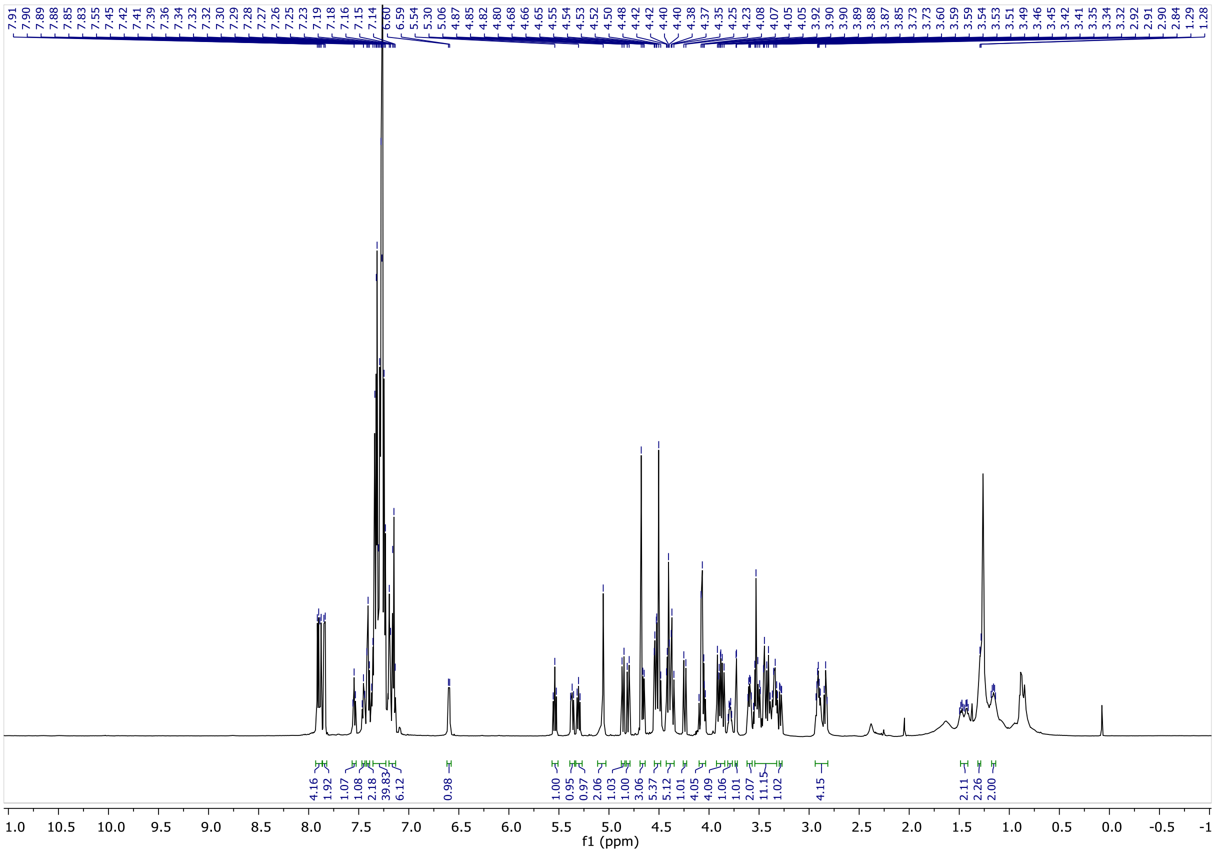

^{13}C NMR: **27**

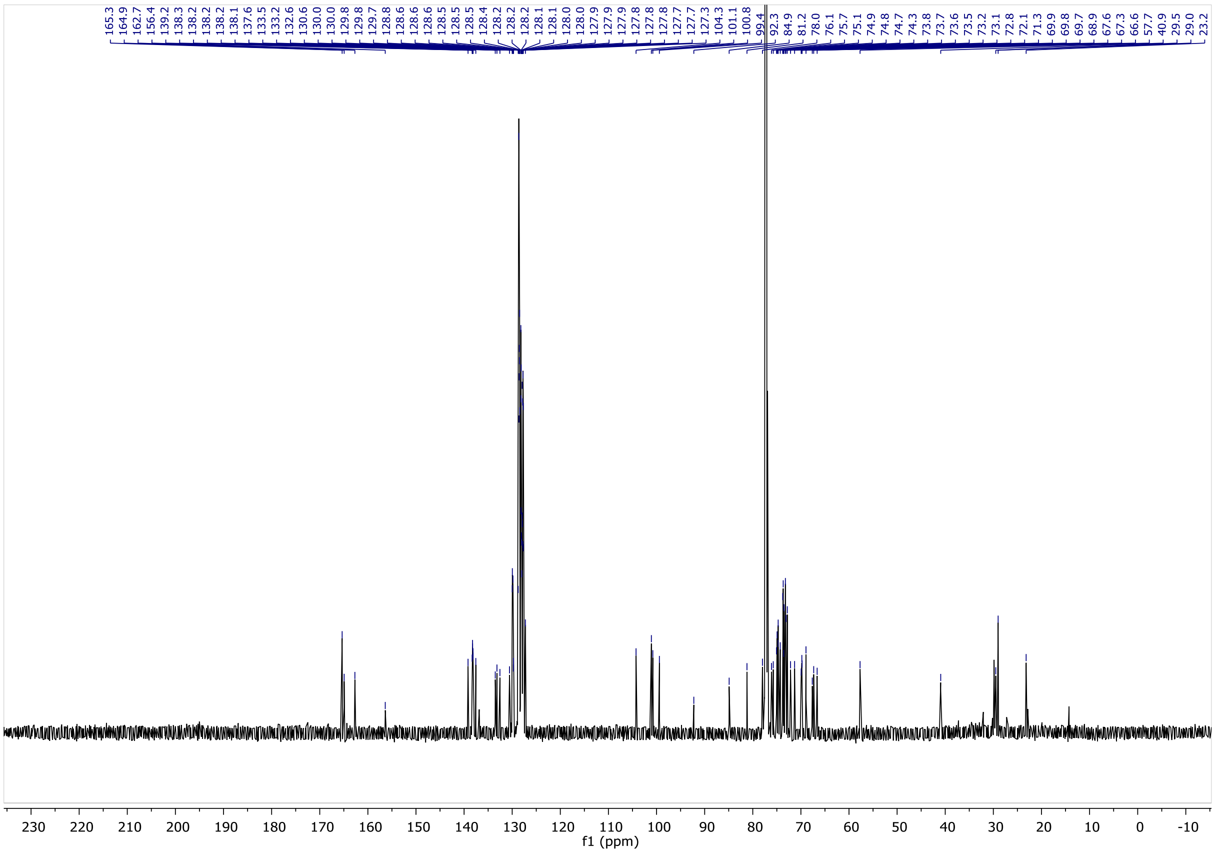

COSY: **27**

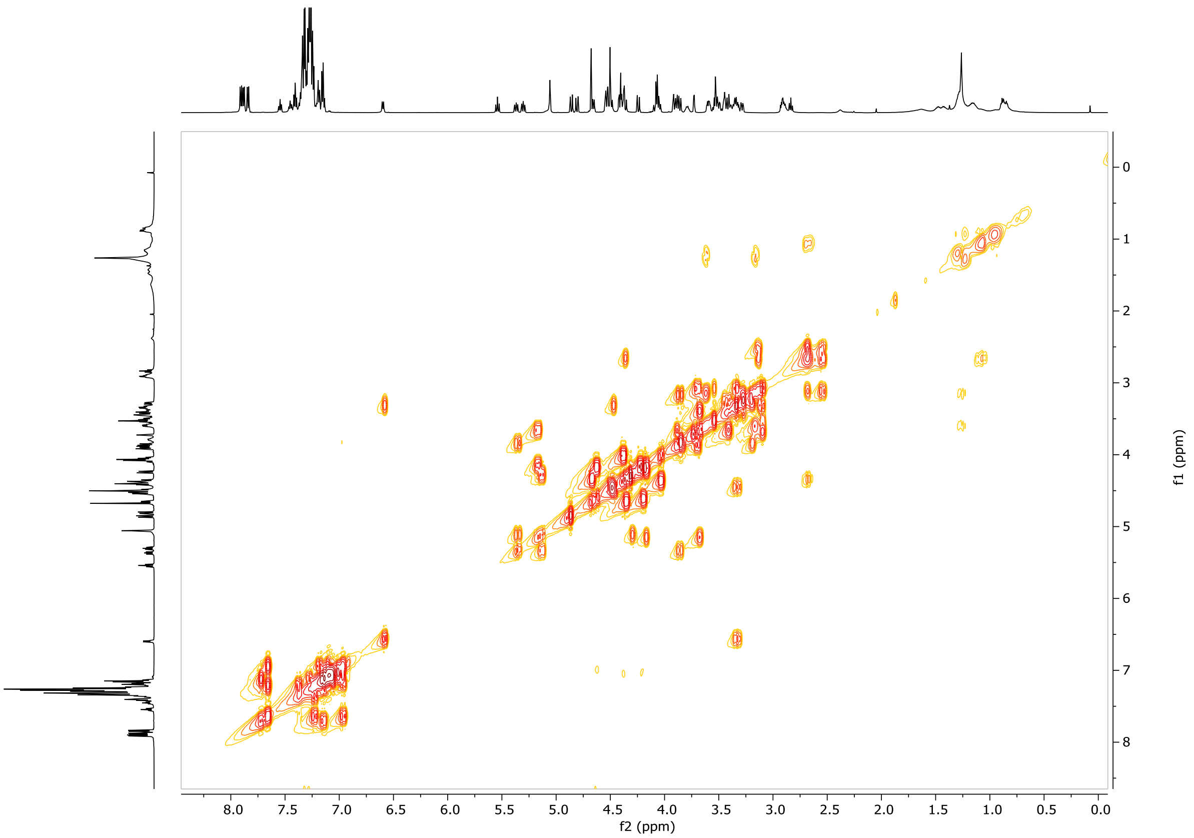

HSQC: **27**

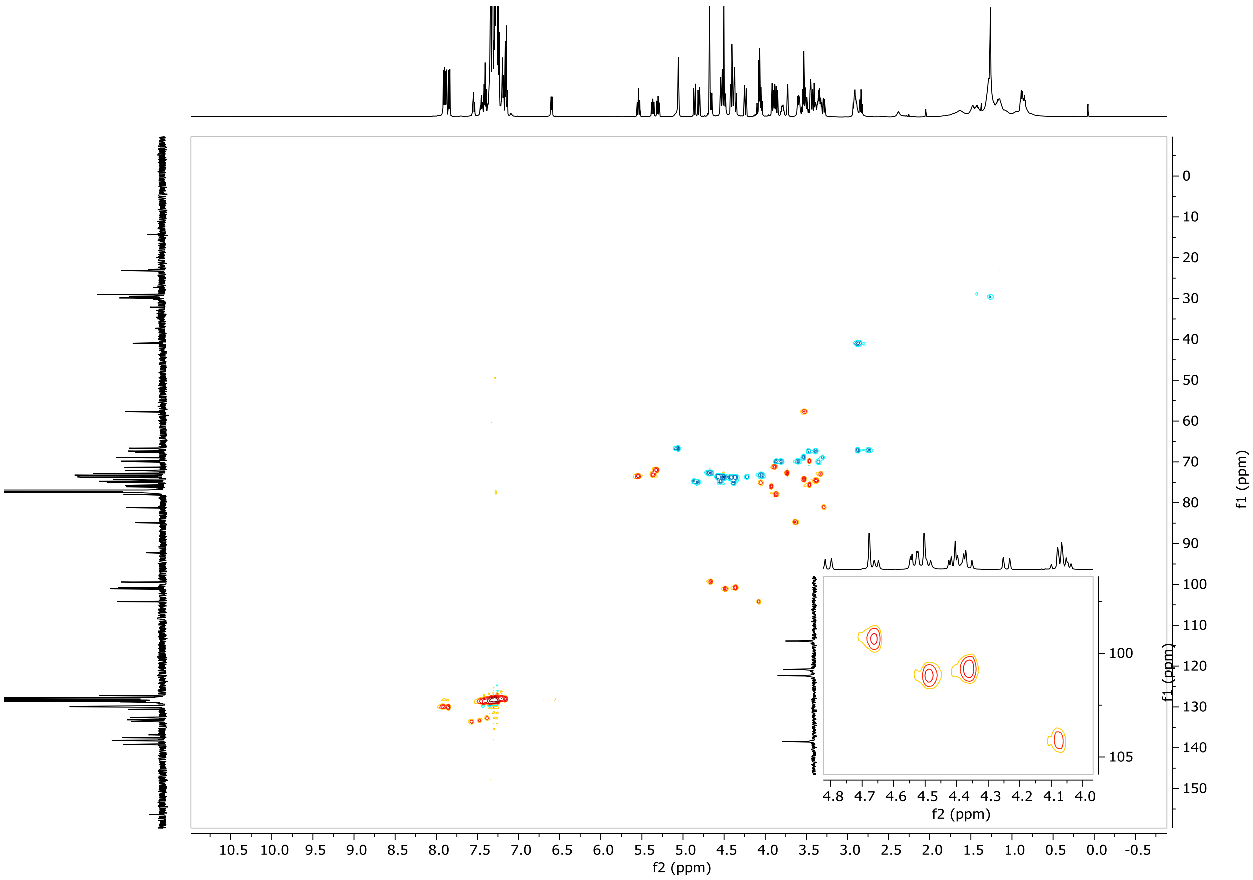

Coupled HSQC: **27**

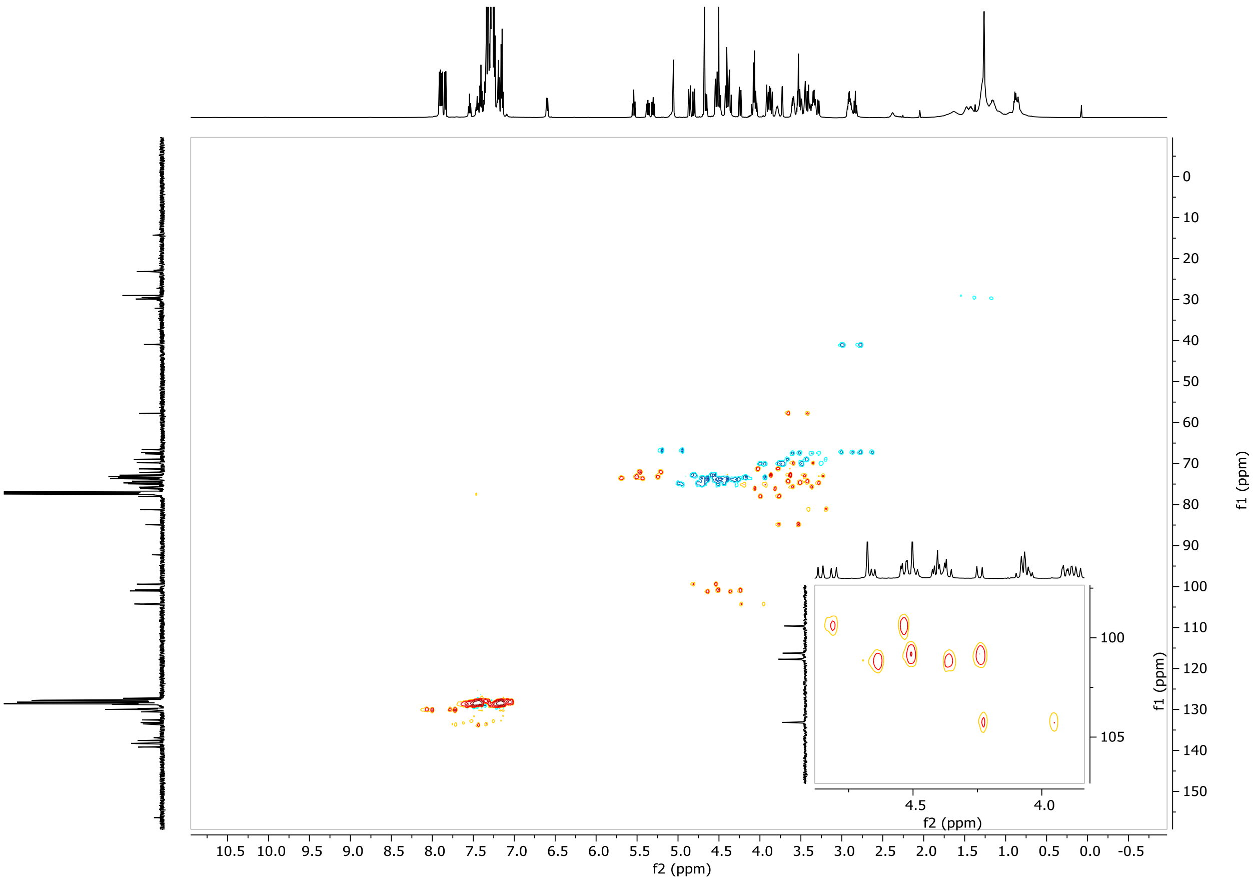

^{1}H NMR: **28**

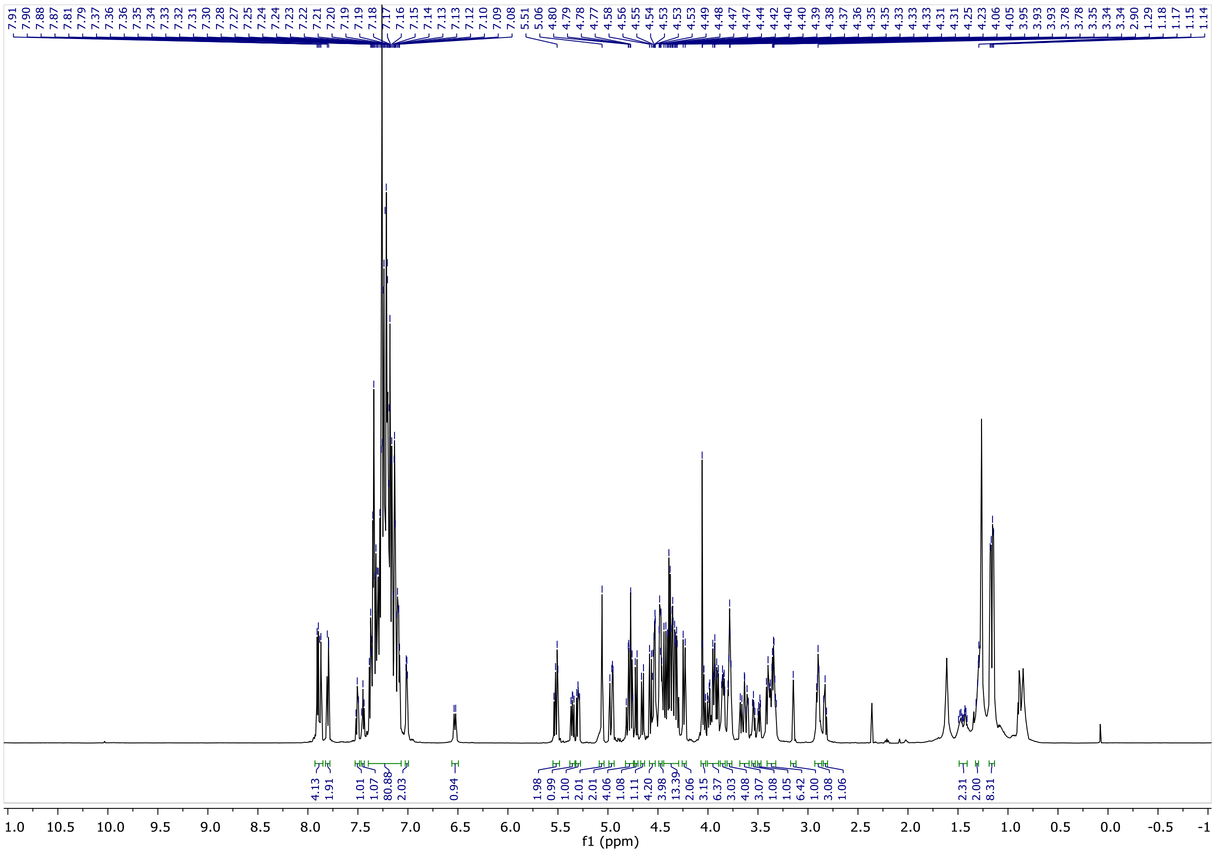

^{13}C NMR: **28**

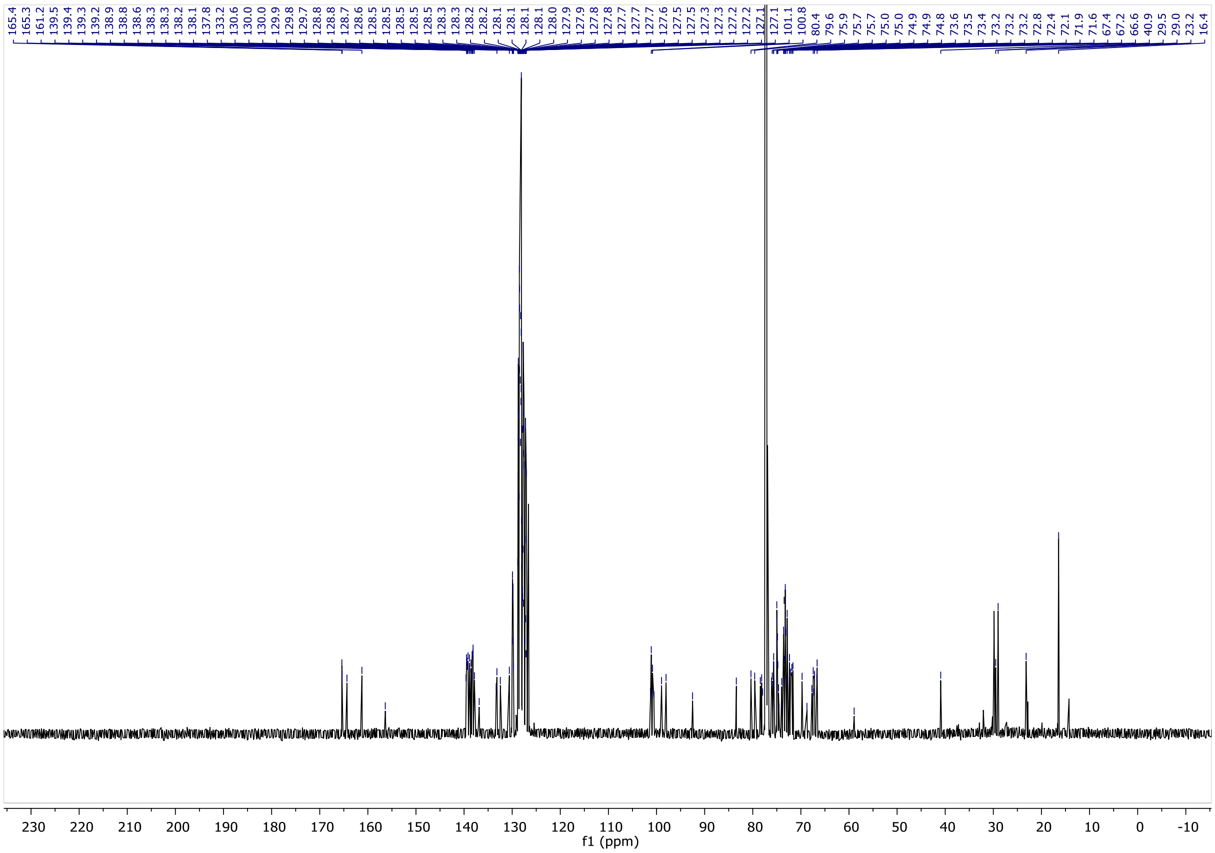

COSY: **28**

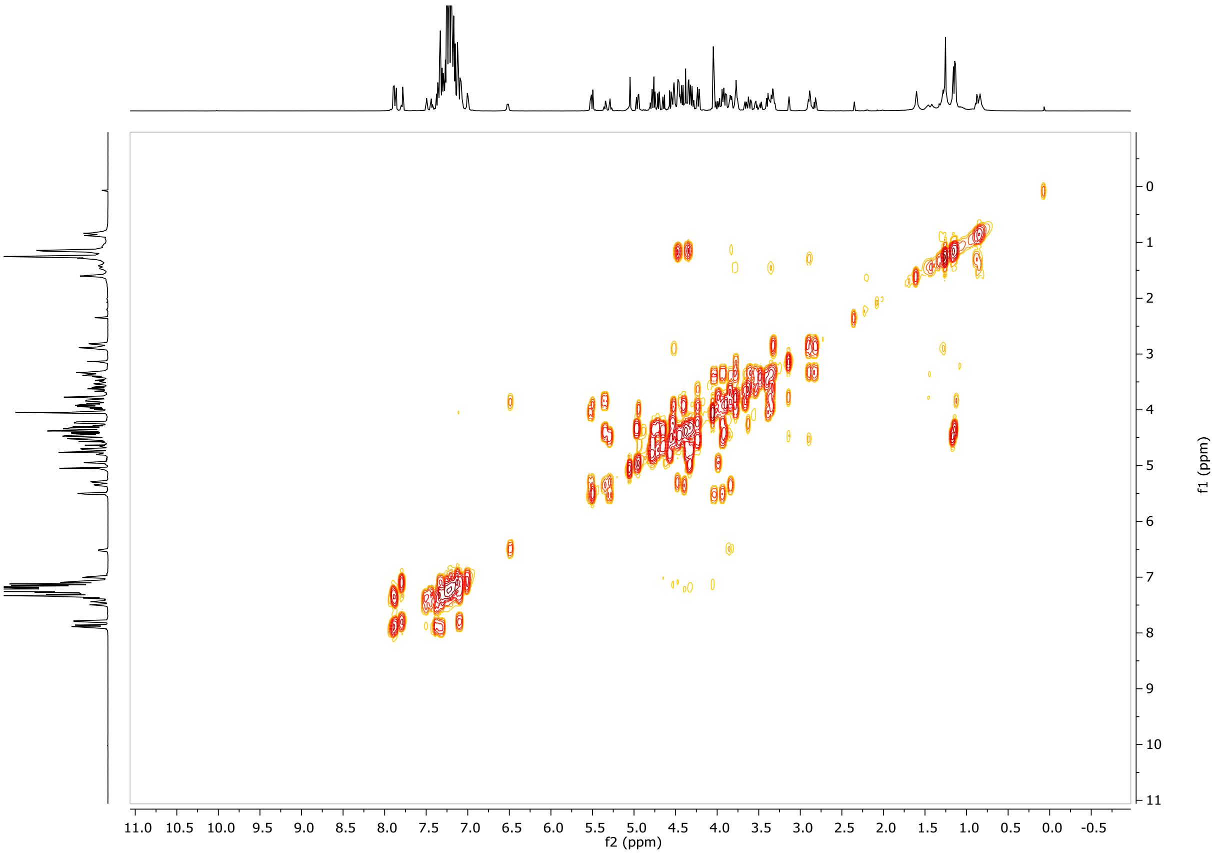

HSQC: **28**

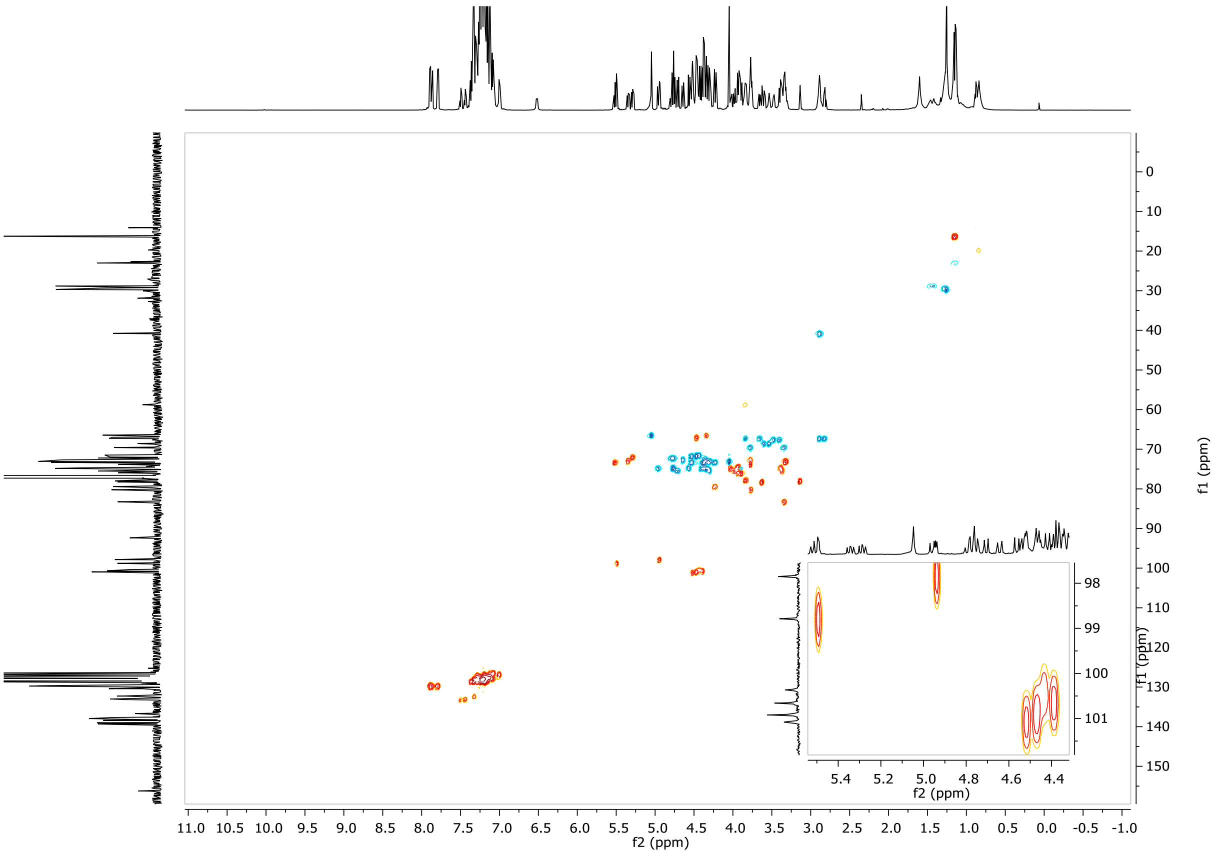

Coupled HSQC: **28**

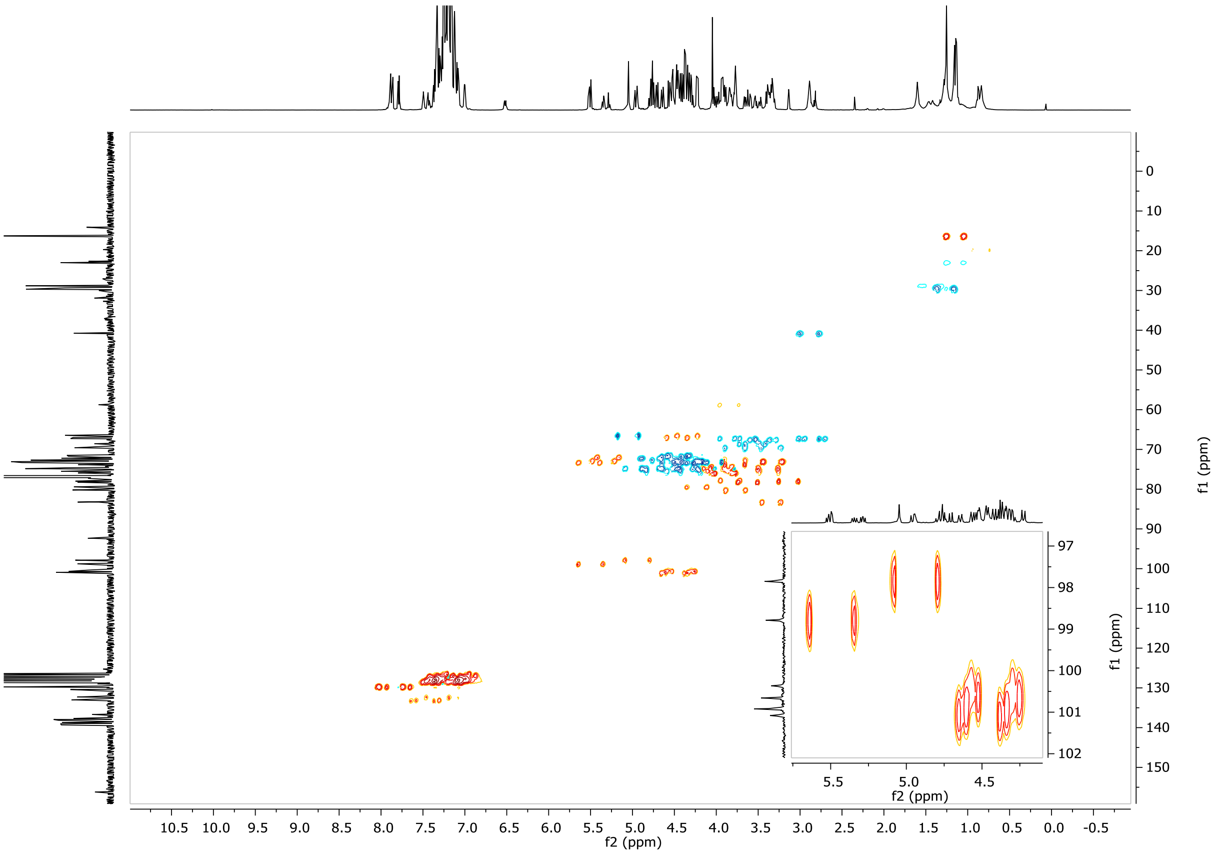

^{1}H NMR: **29**

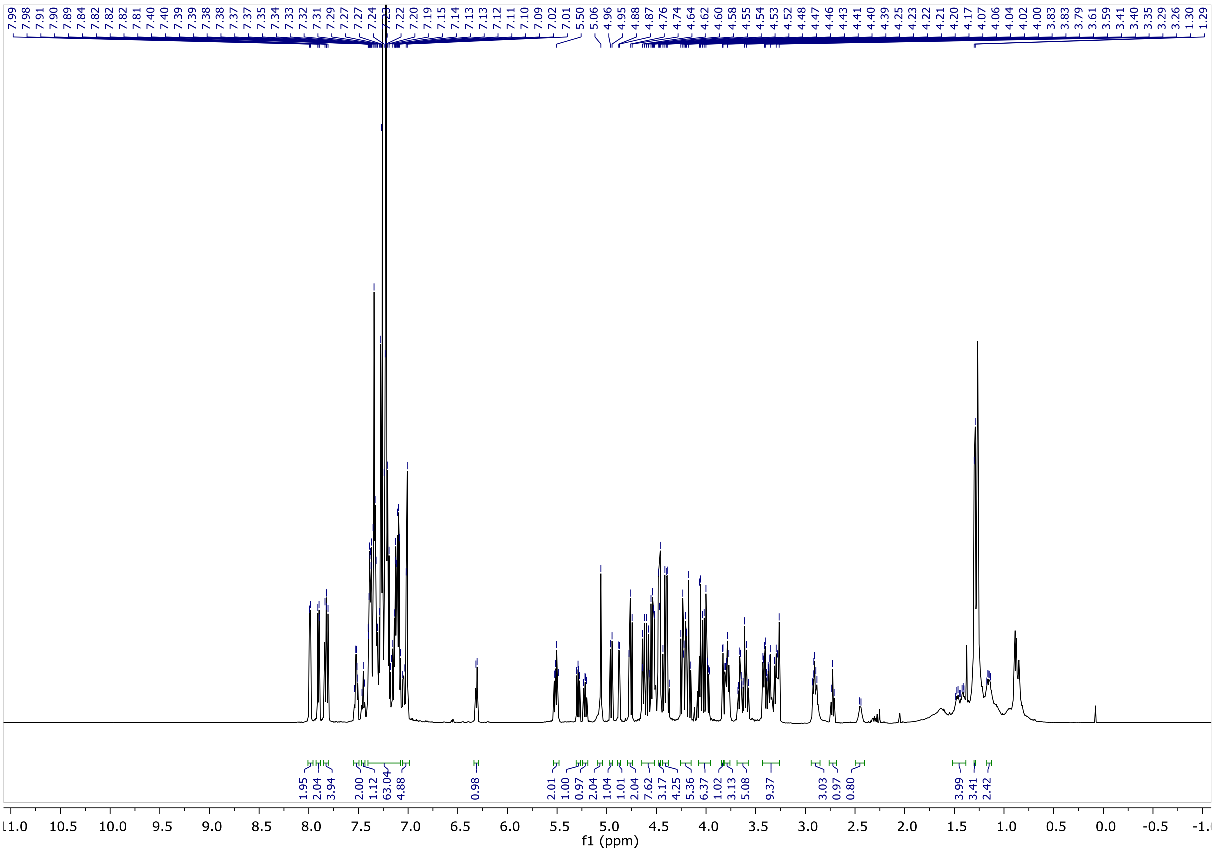

^{13}C NMR: **29**

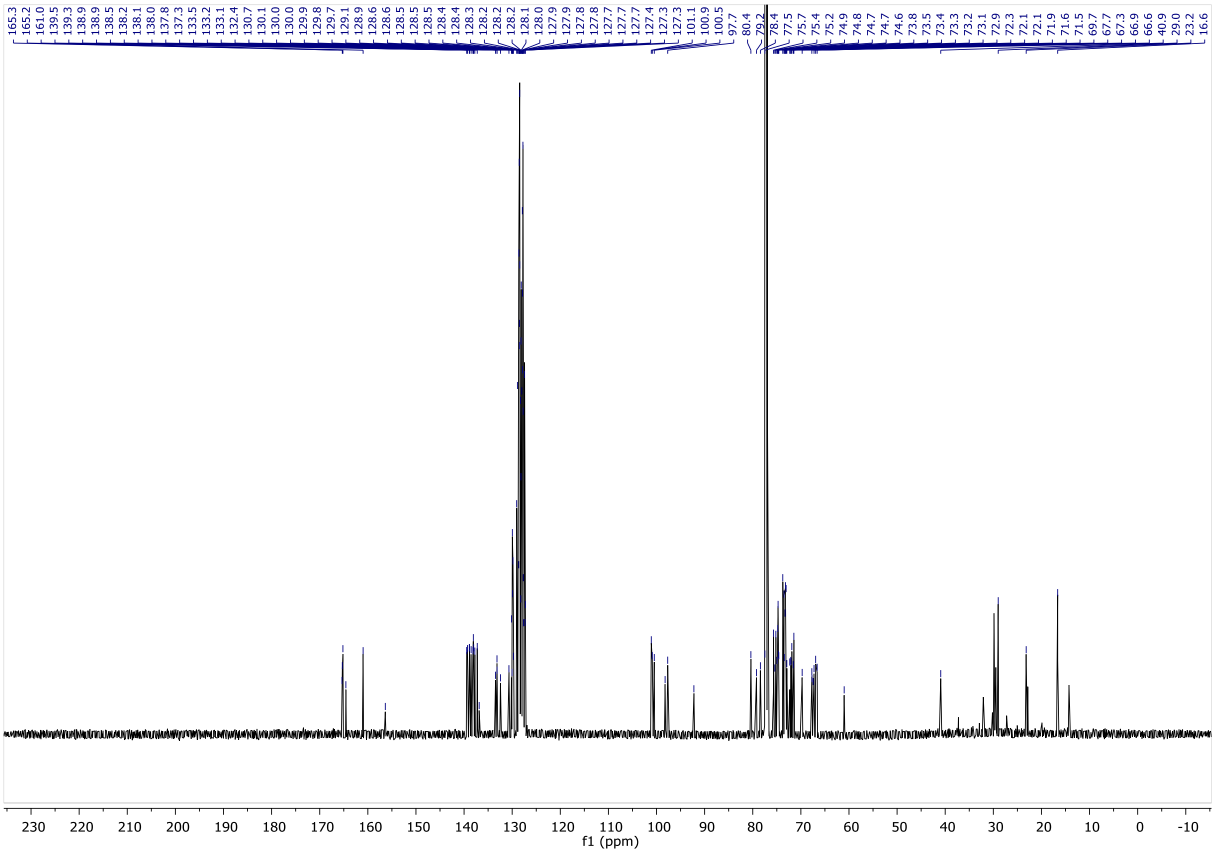

COSY: **29**

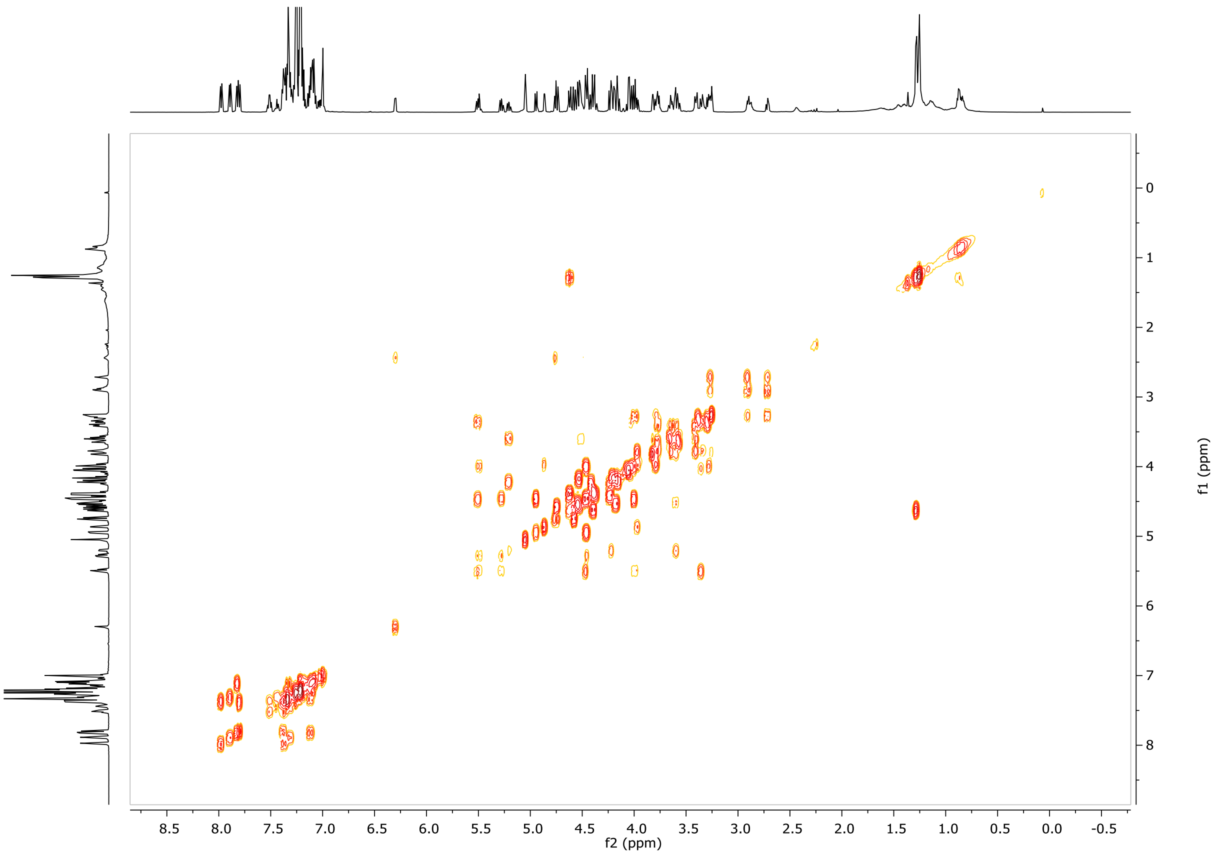

HSQC: **29**

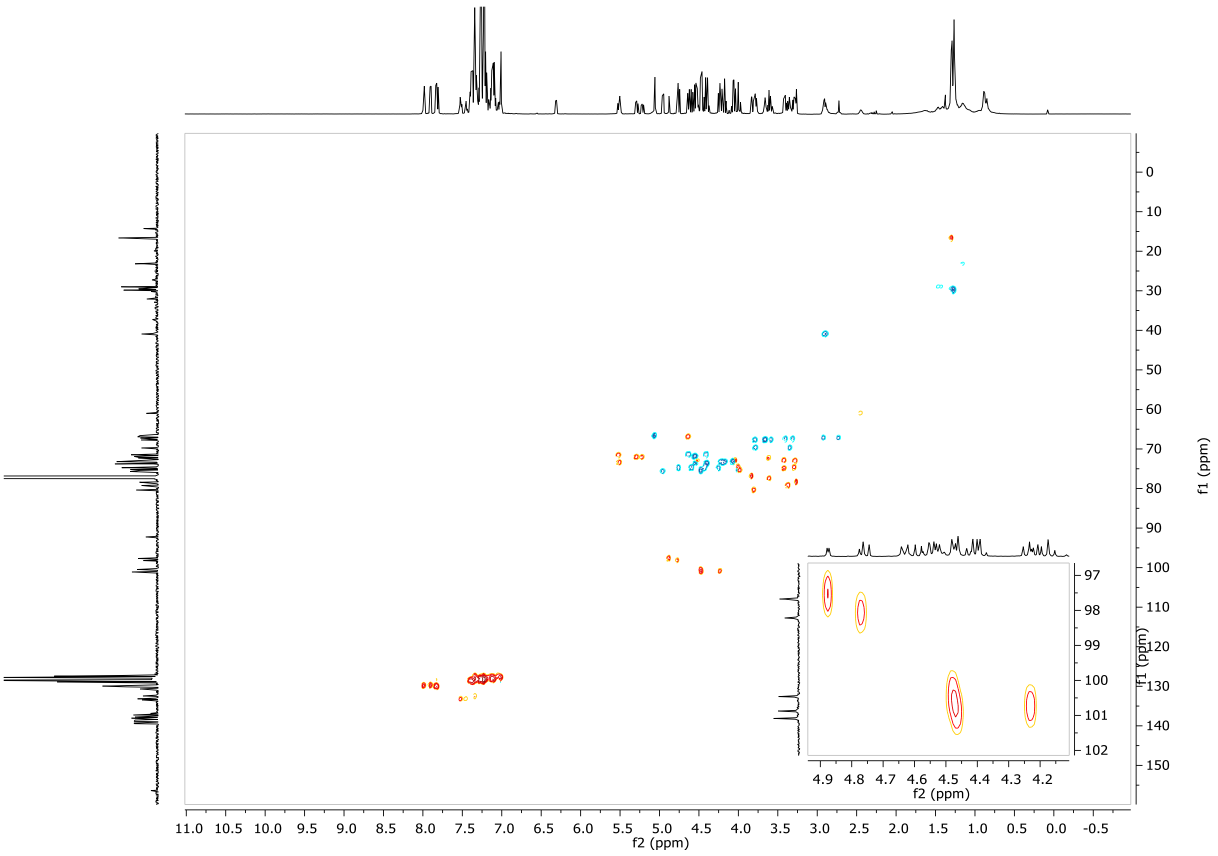

Coupled HSQC: **29**

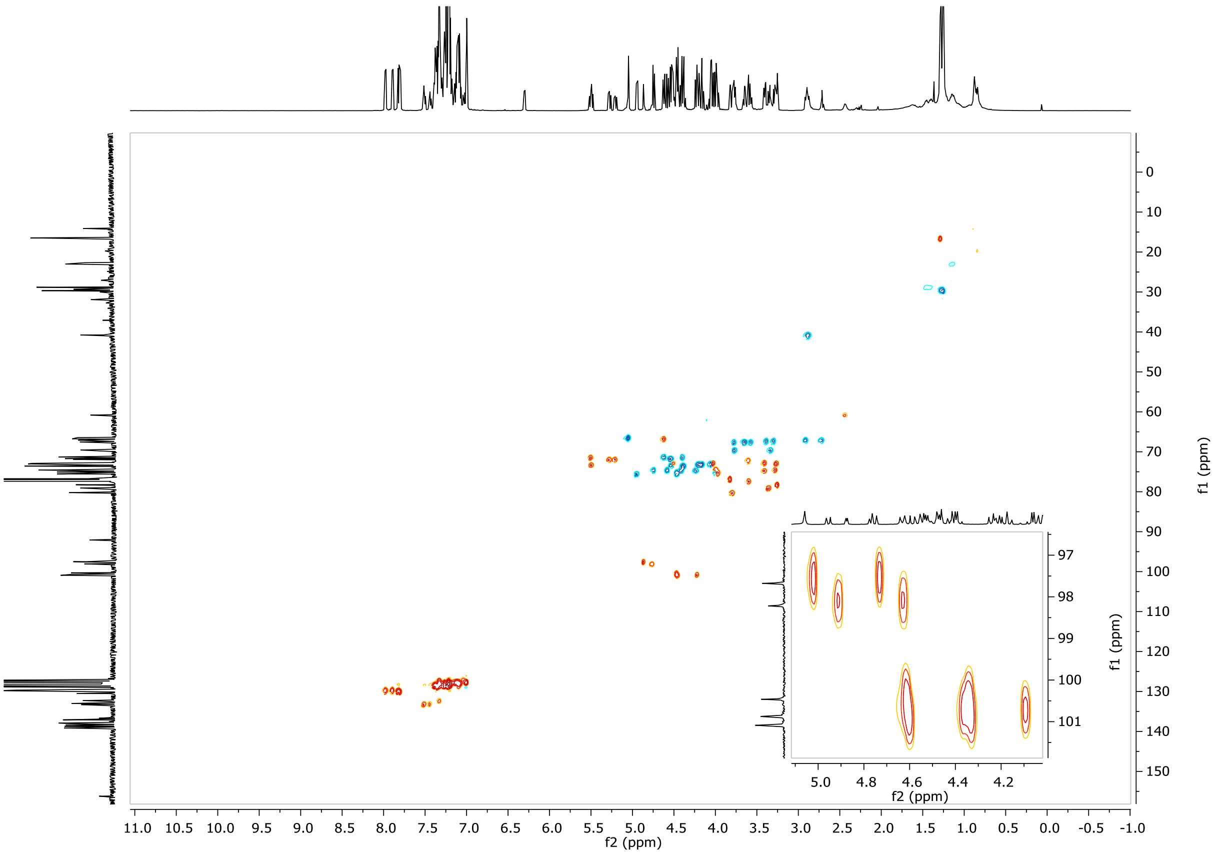

^{1}H NMR: **31**

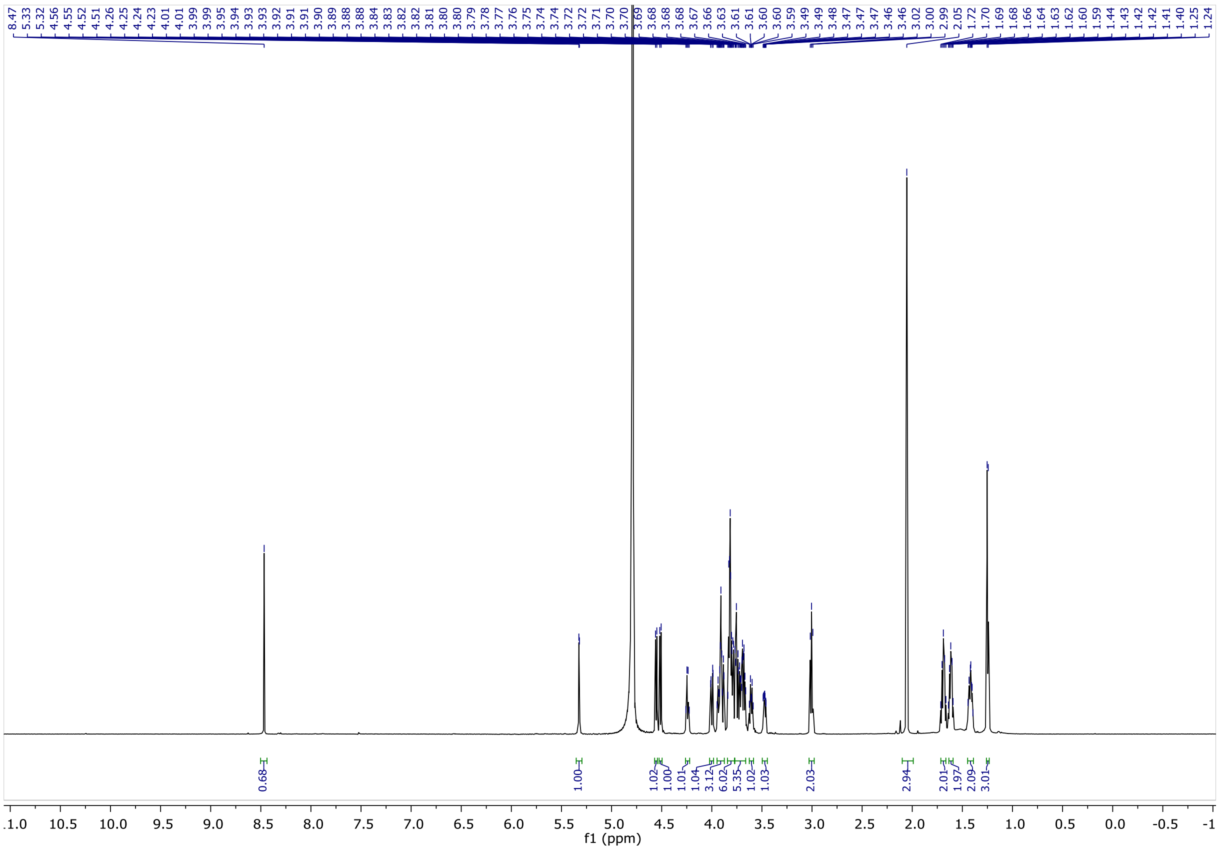

^{13}C NMR: **31**

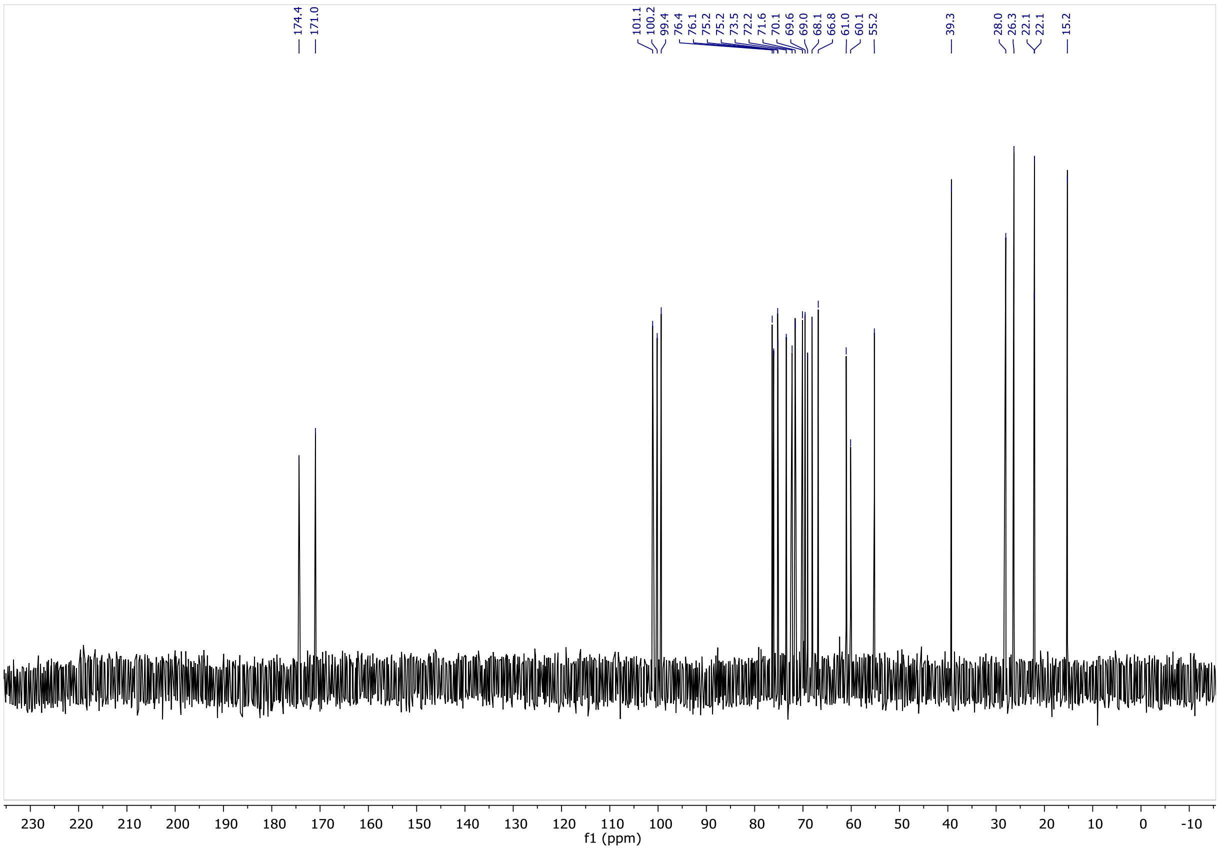

COSY: **31**

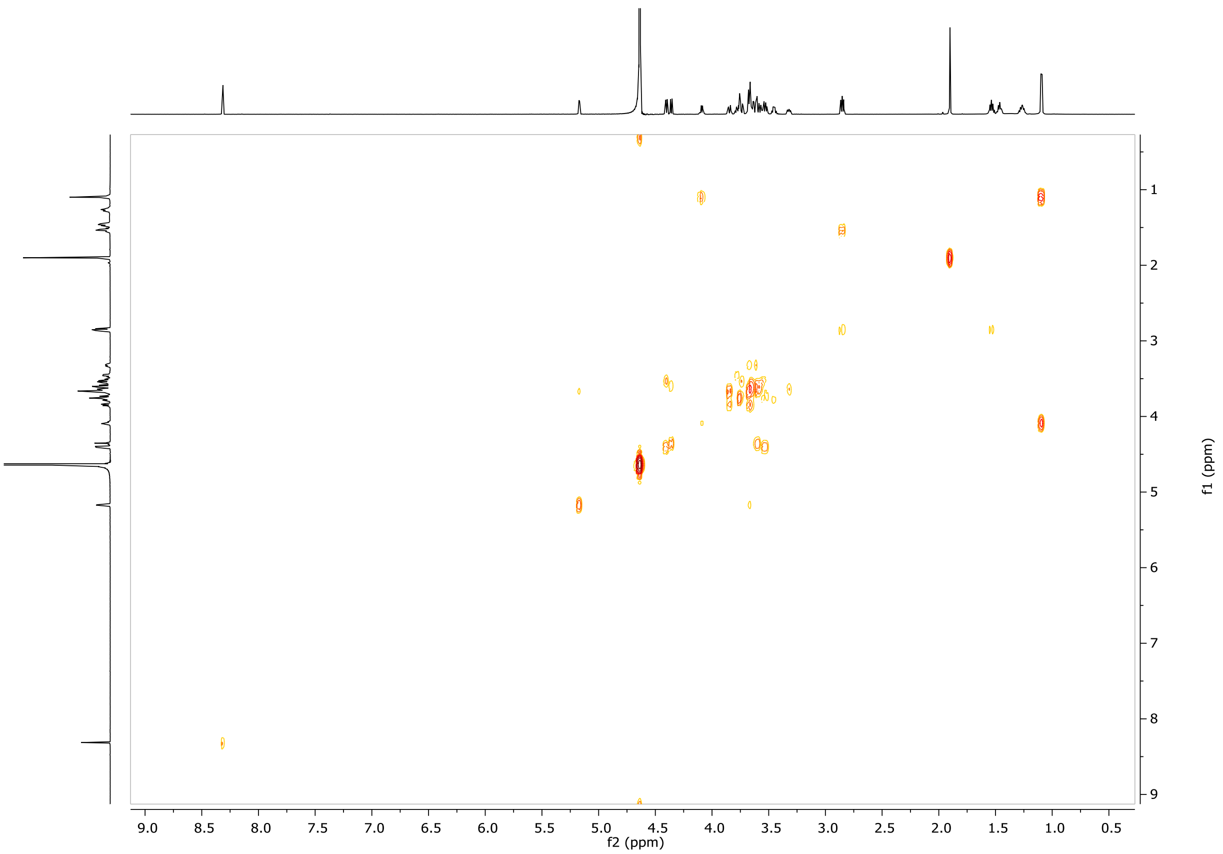

HSQC: **31**

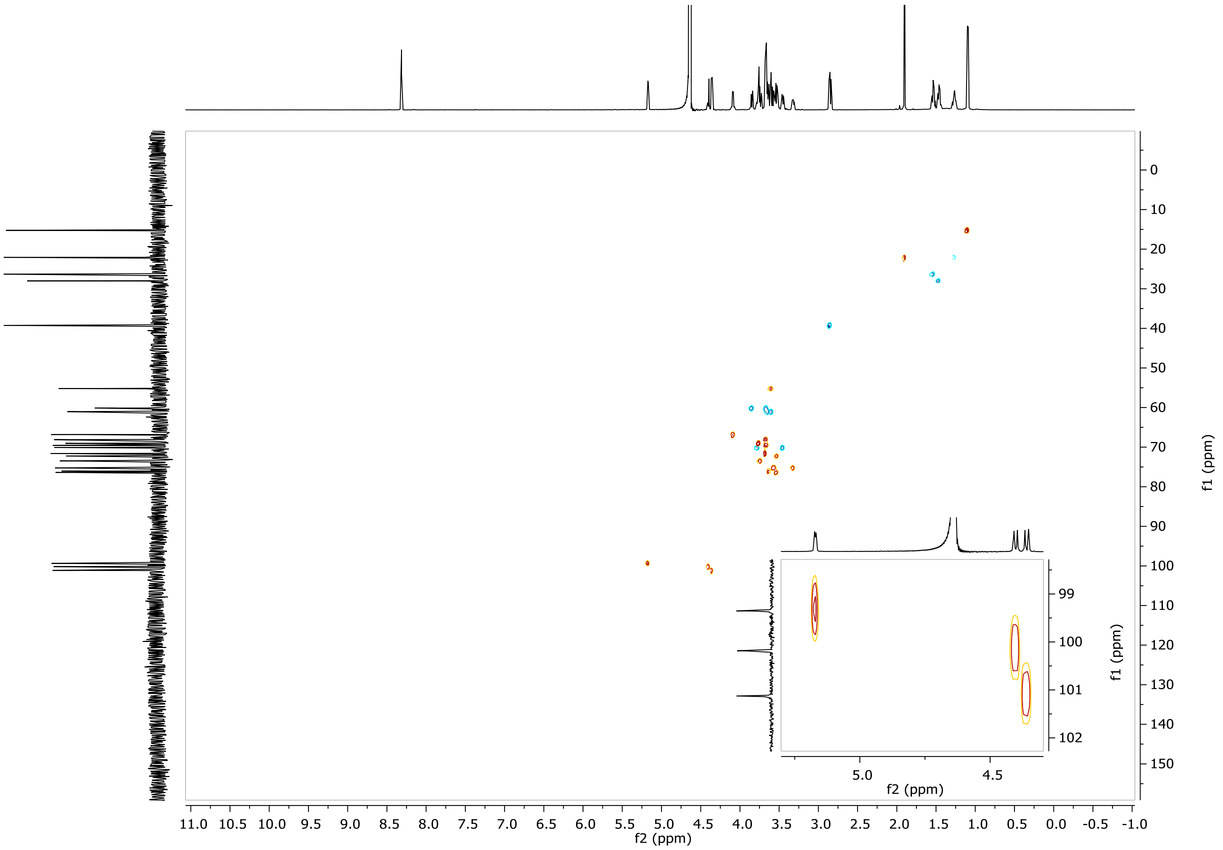

Coupled HSQC: **31**

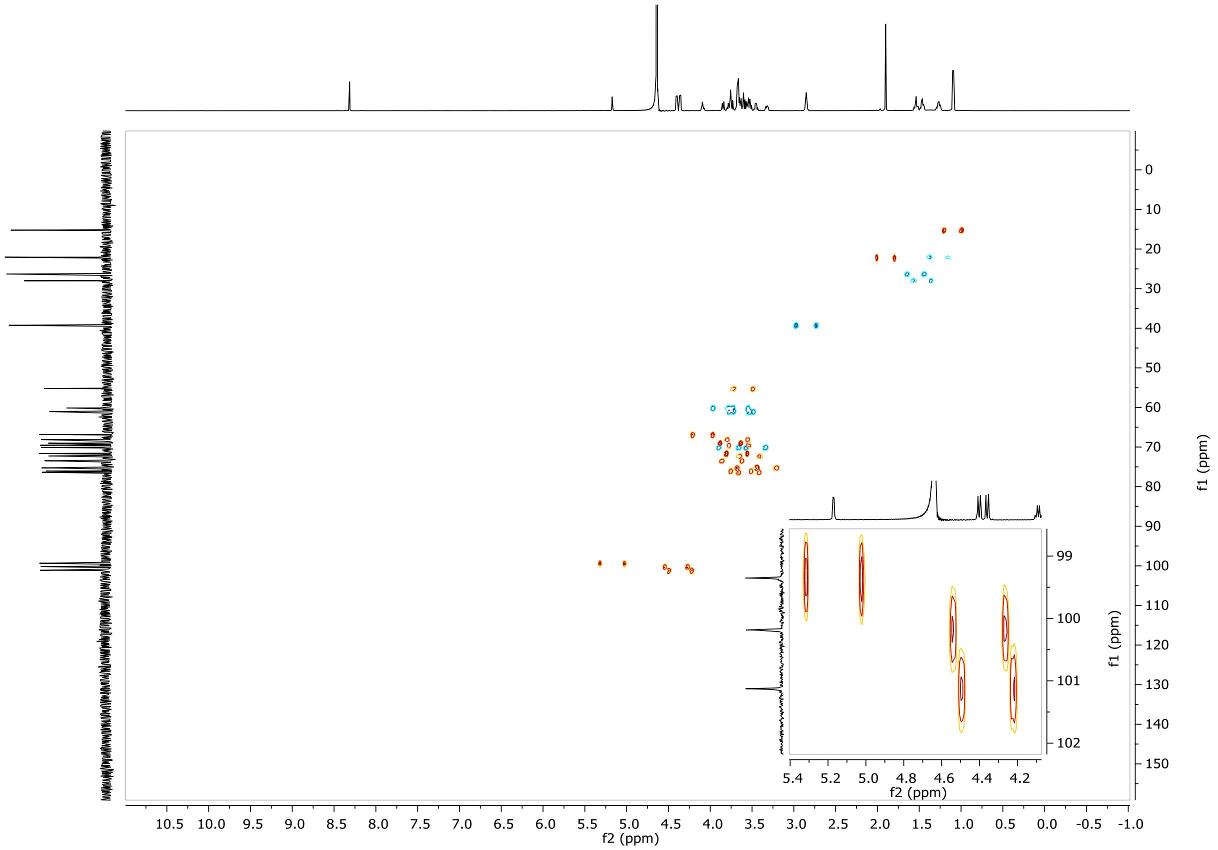

^{1}H NMR: **32**

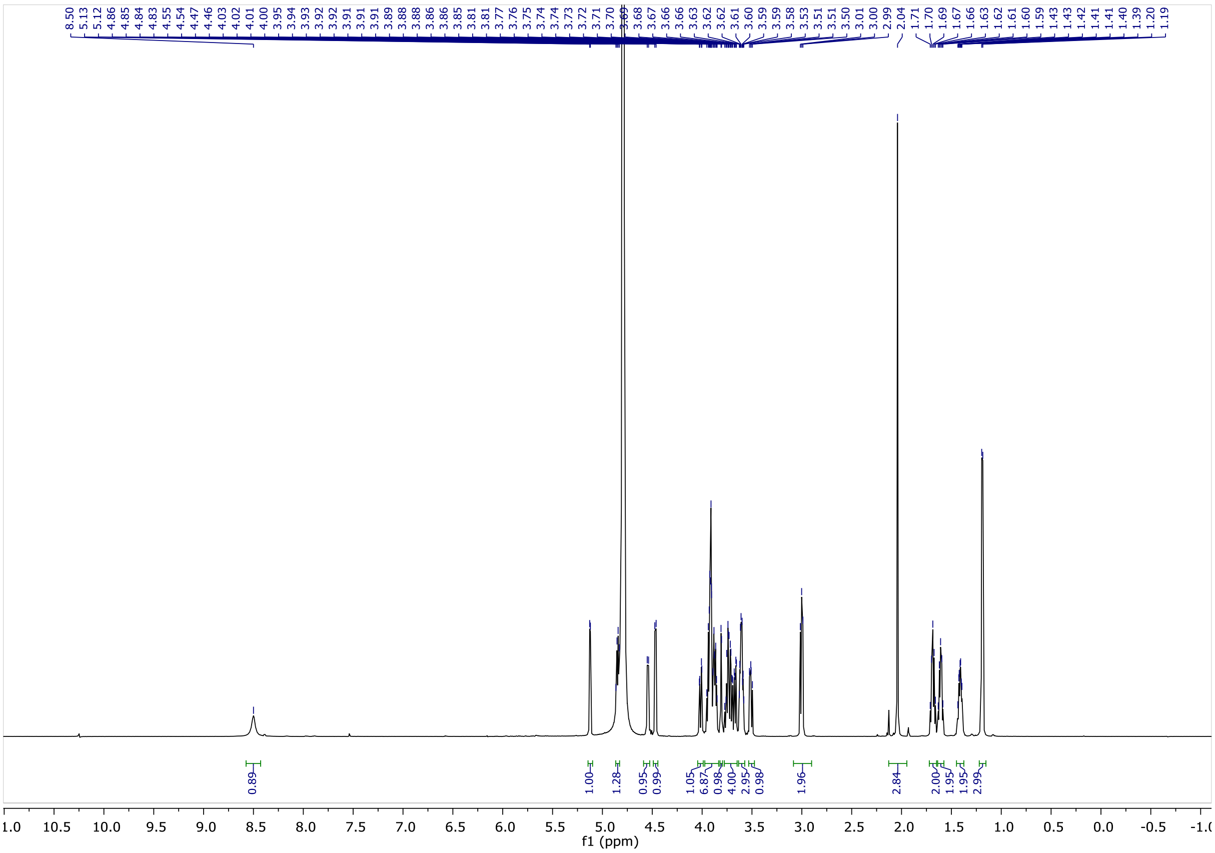

[13] C NMR: **32**

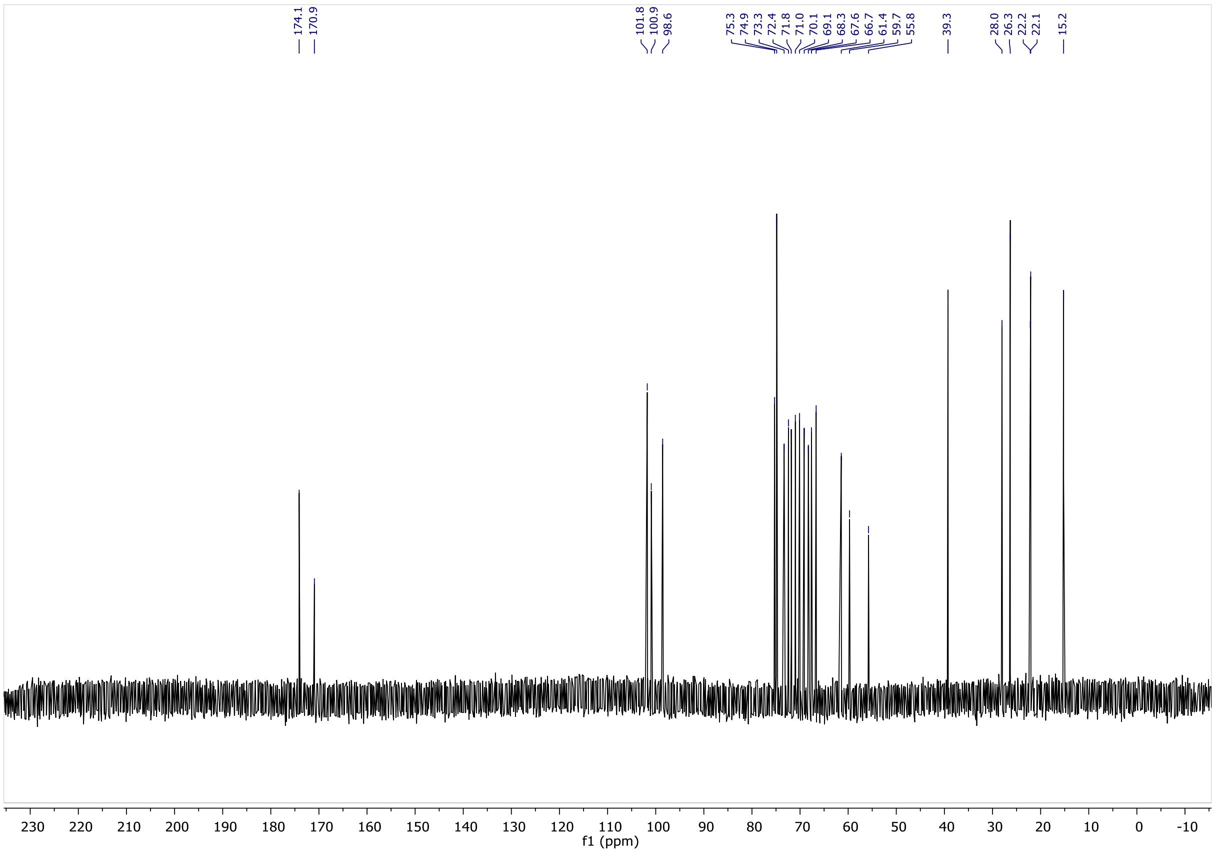

COSY: **32**

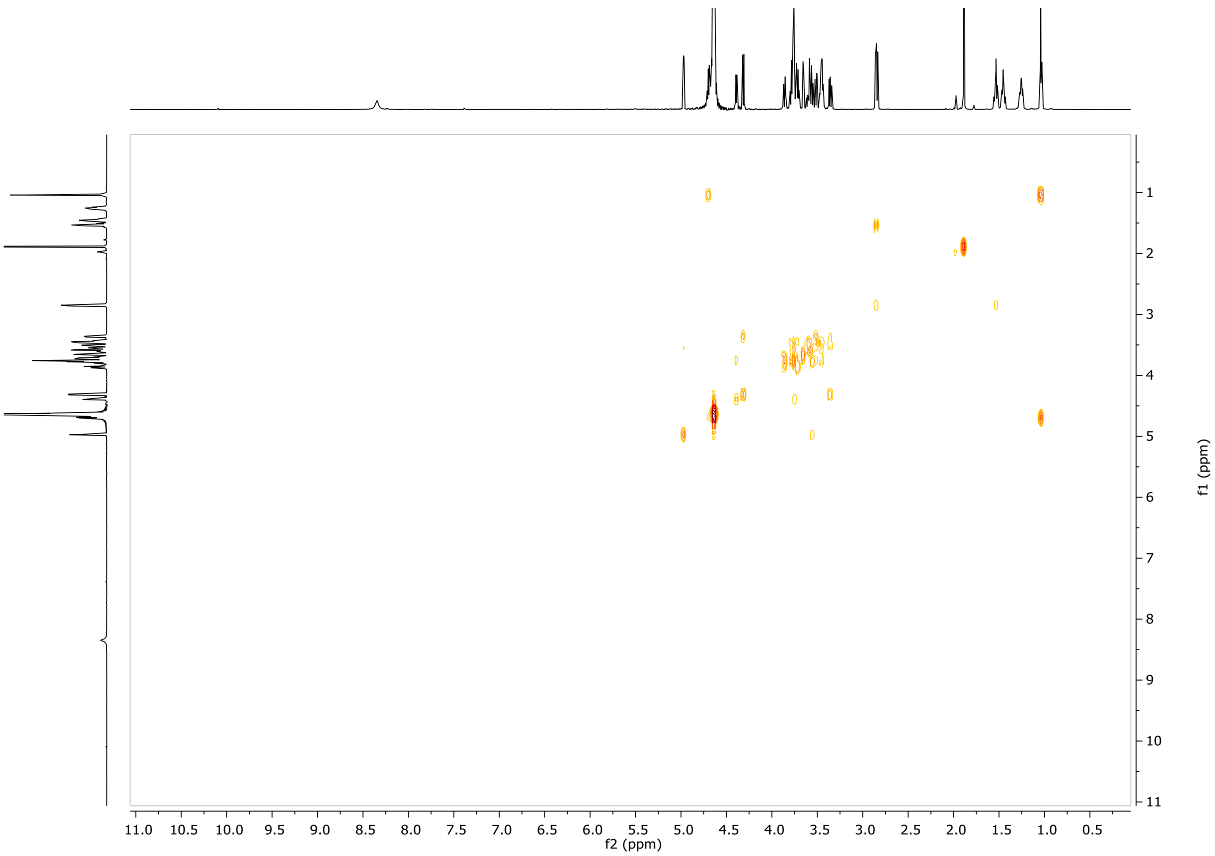

HSQC: **32**

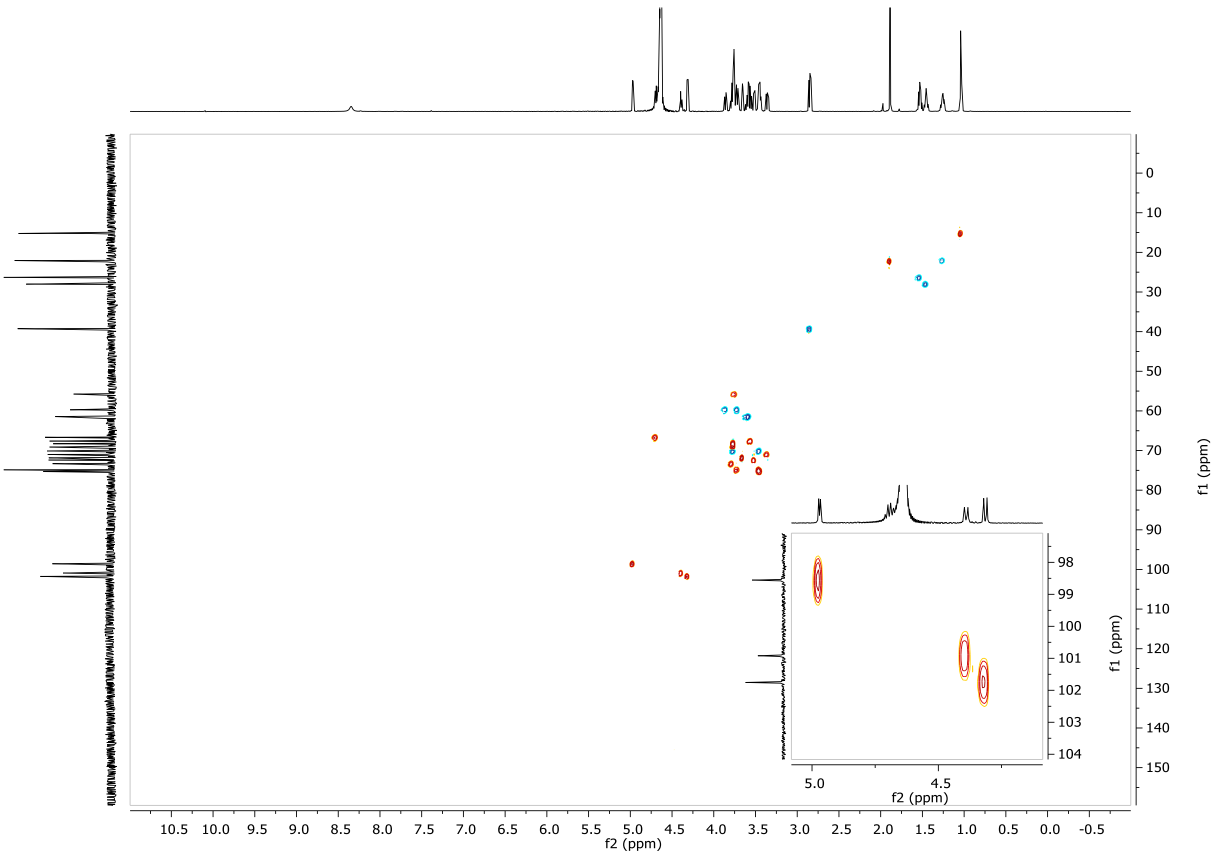

Coupled HSQC: **32**

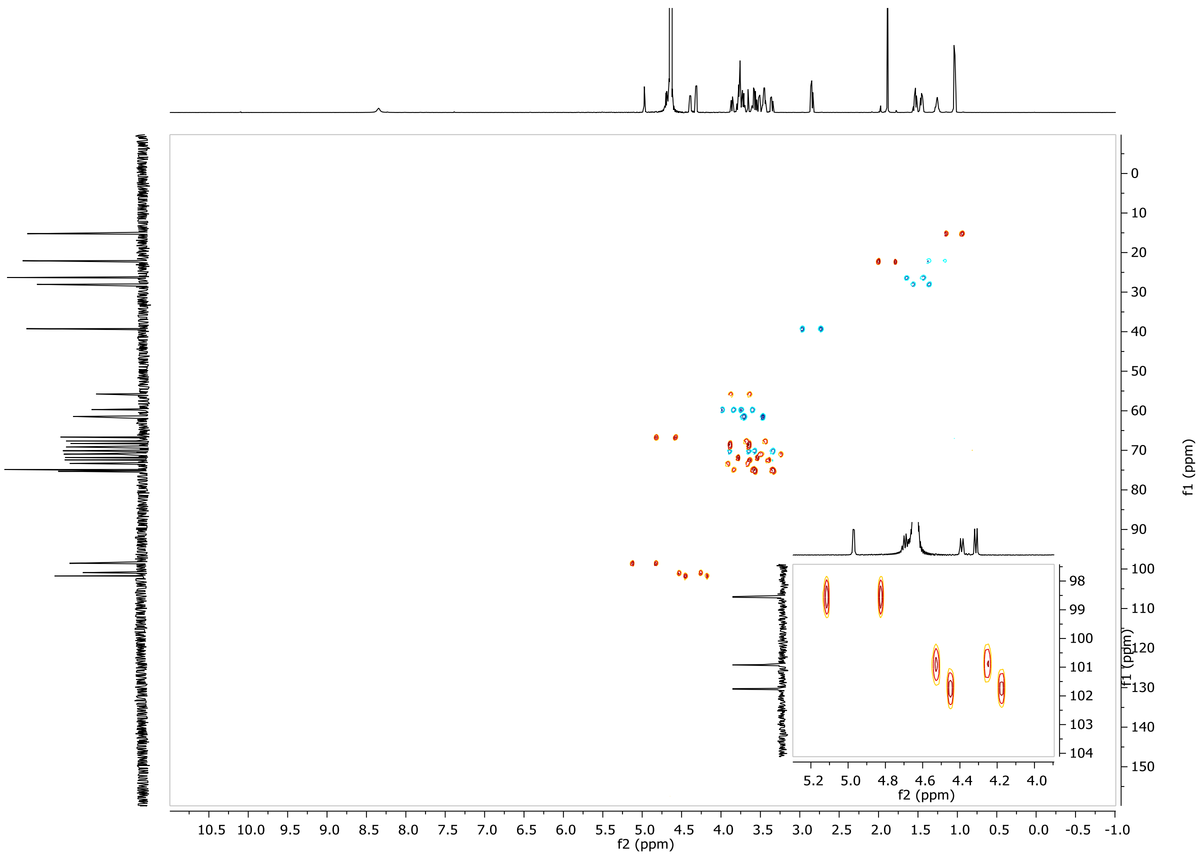

^{1}H NMR: **33**

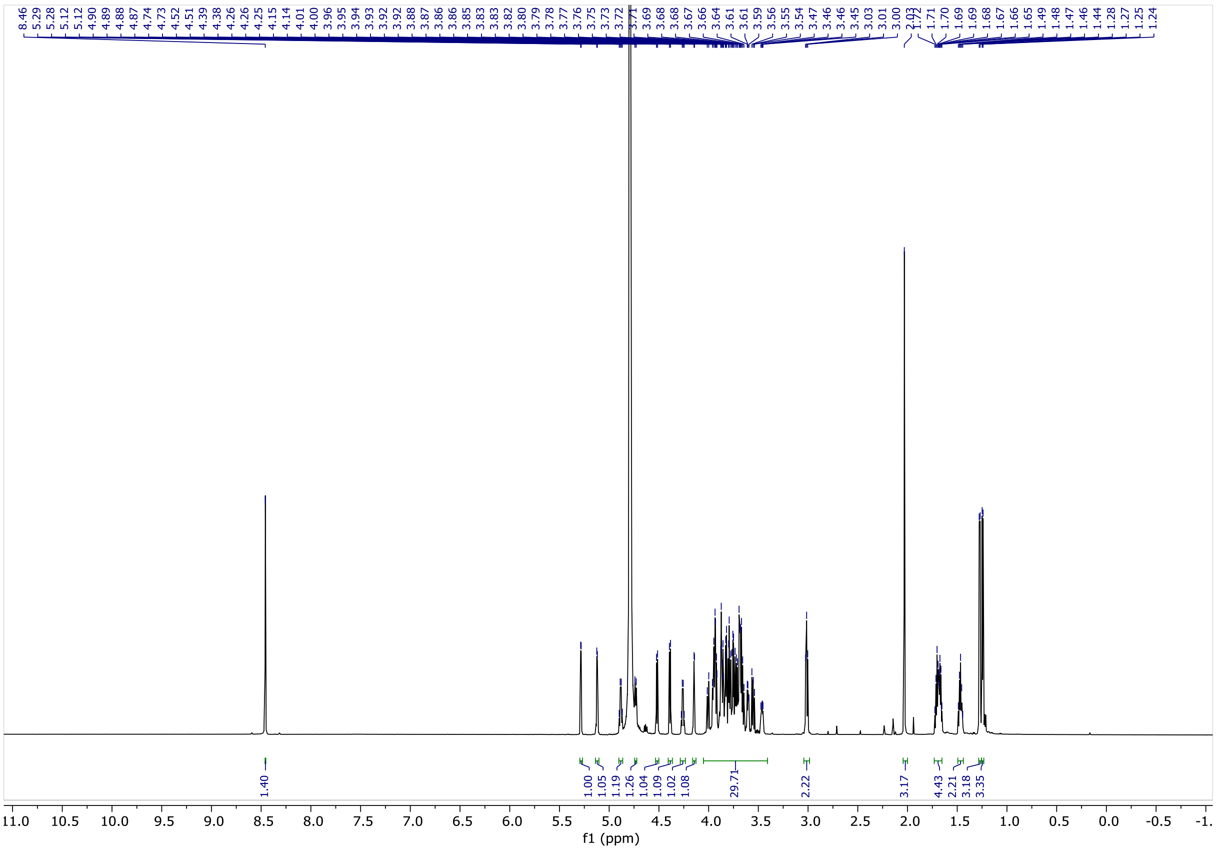

^{13}C NMR: **33**

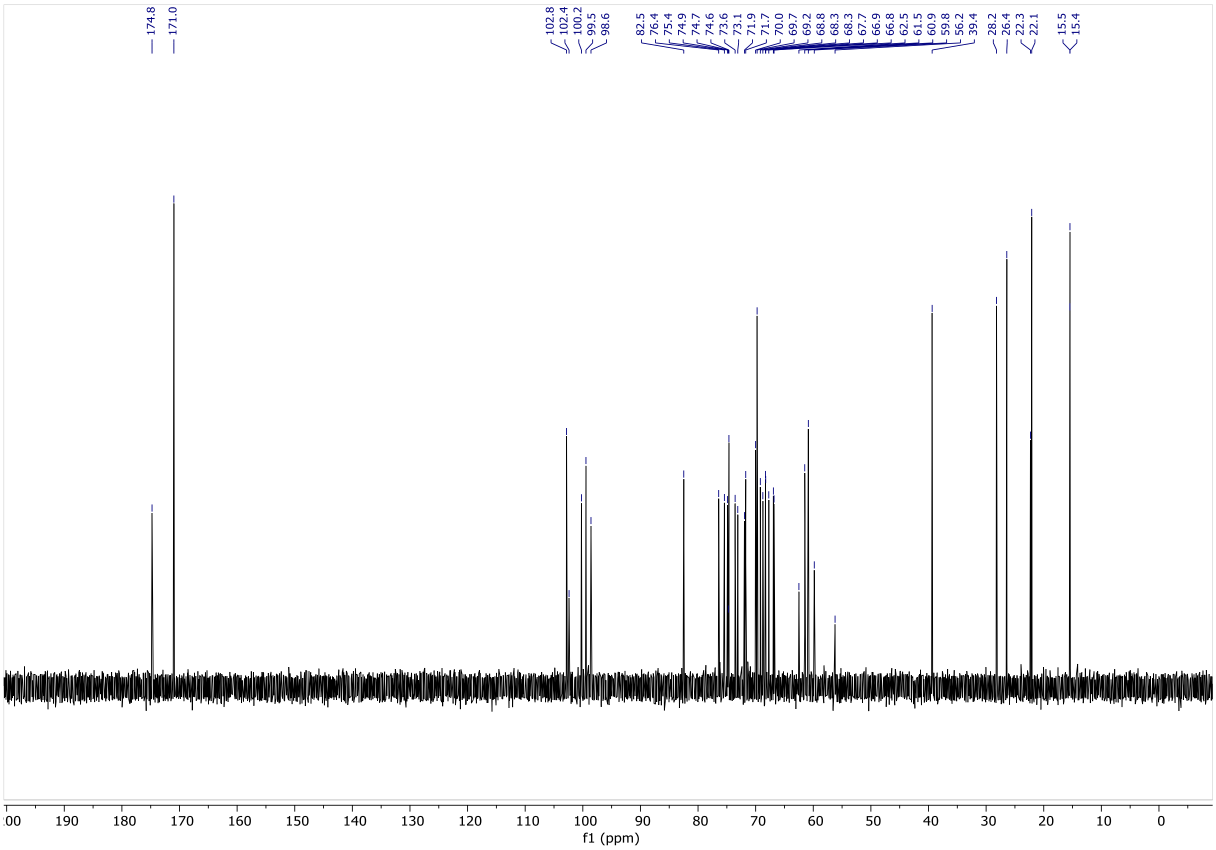

COSY: **33**

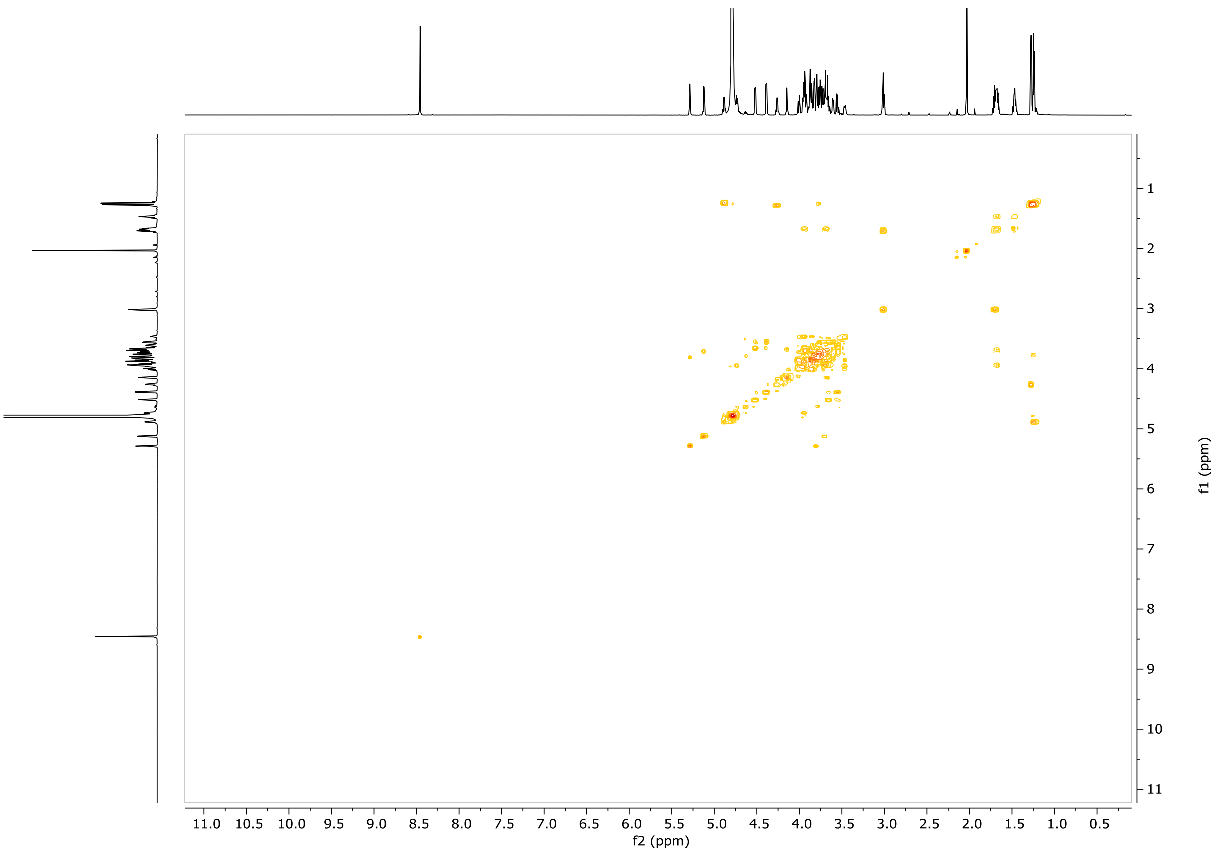

HSQC: **33**

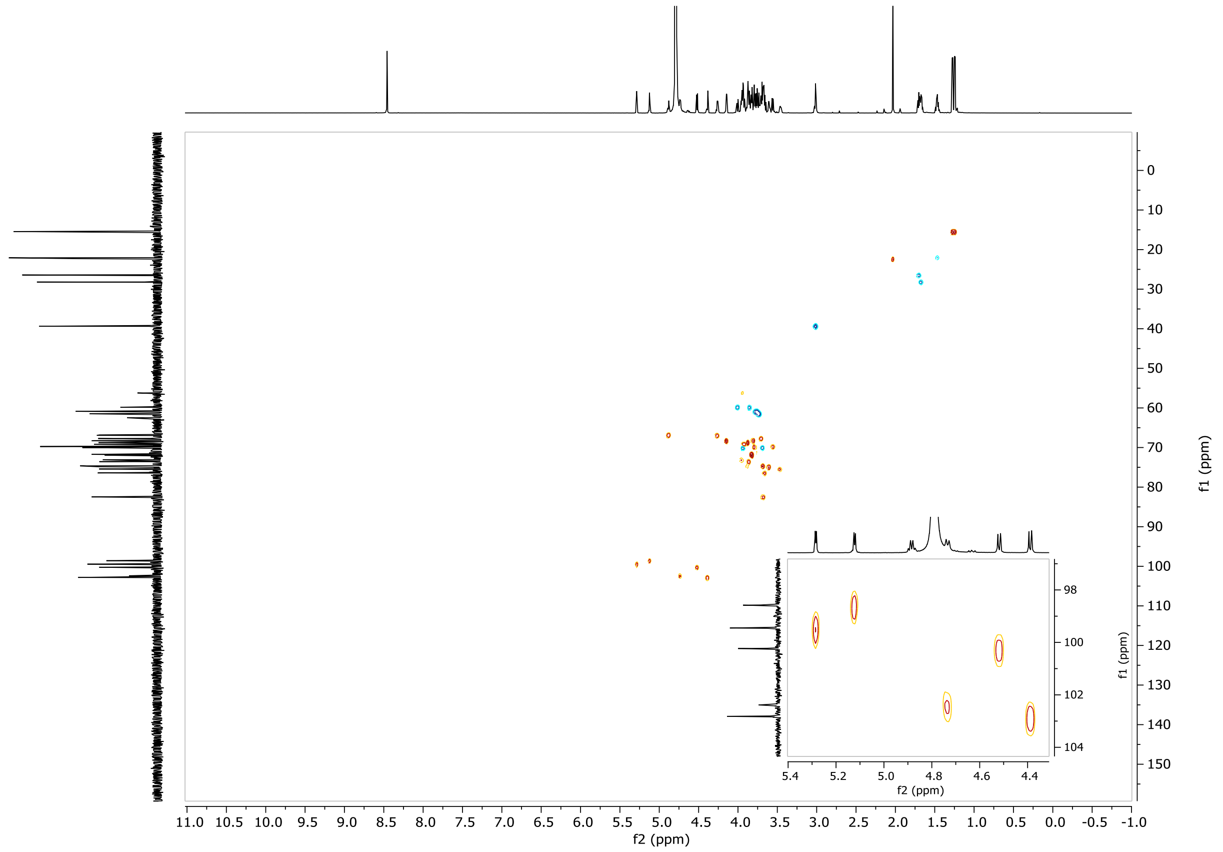

Coupled HSQC: **33**

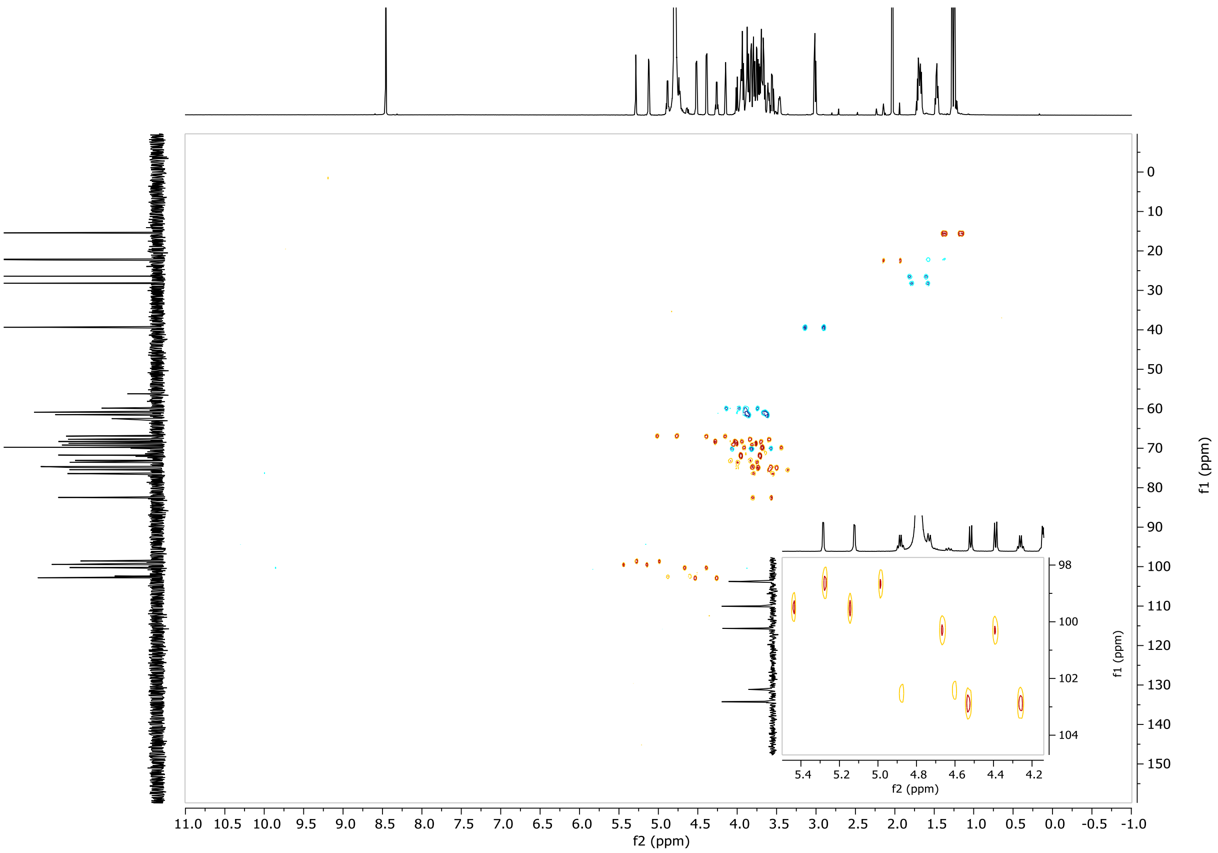

^{1}H NMR: **34**

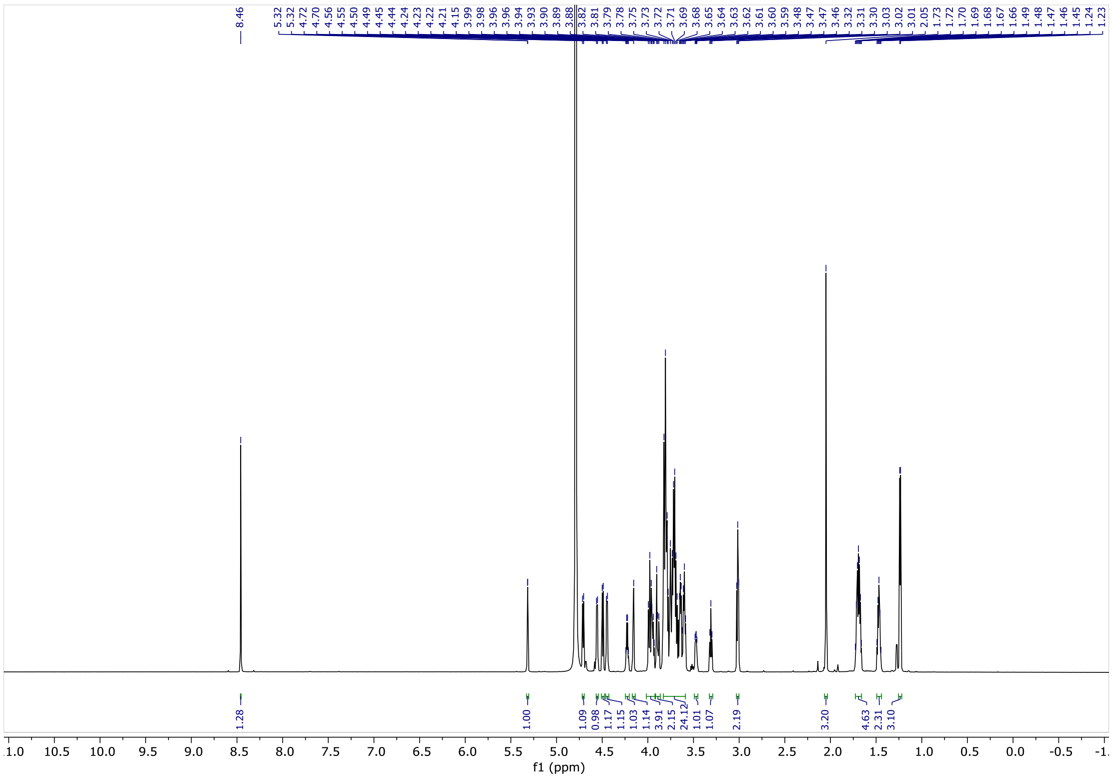

^{13}C NMR: **34**

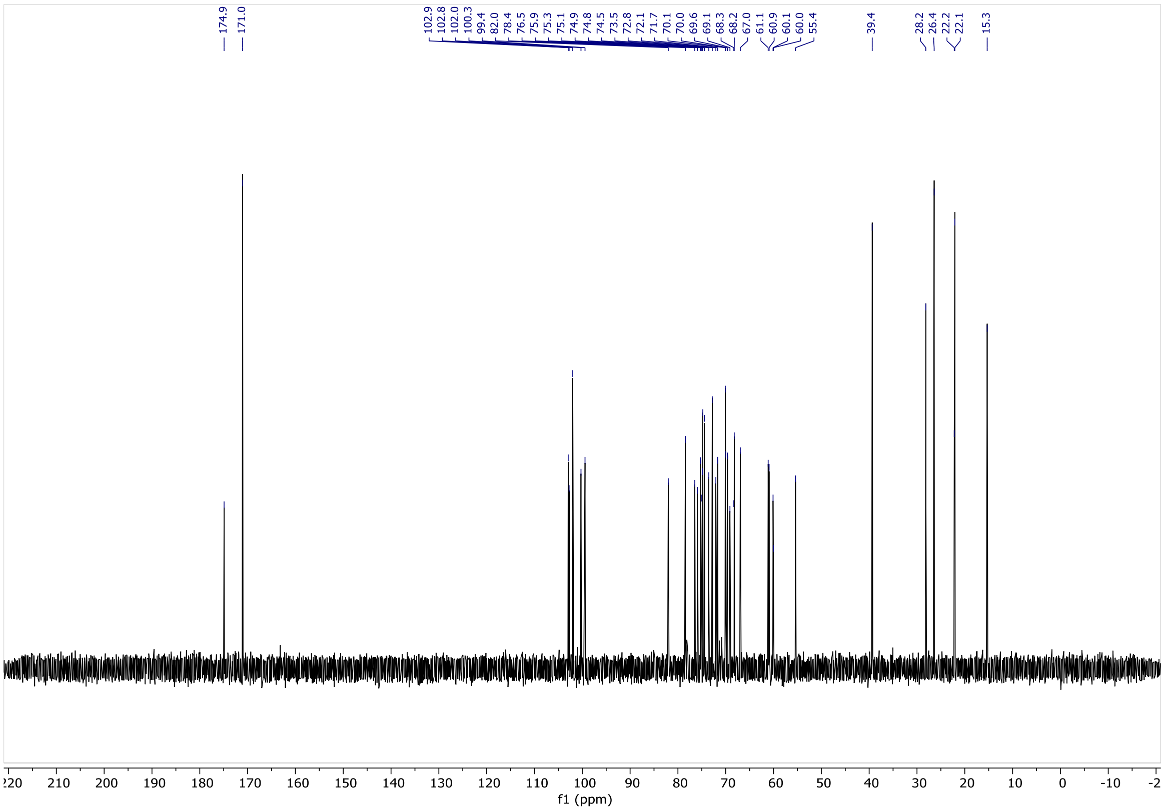

COSY: **34**

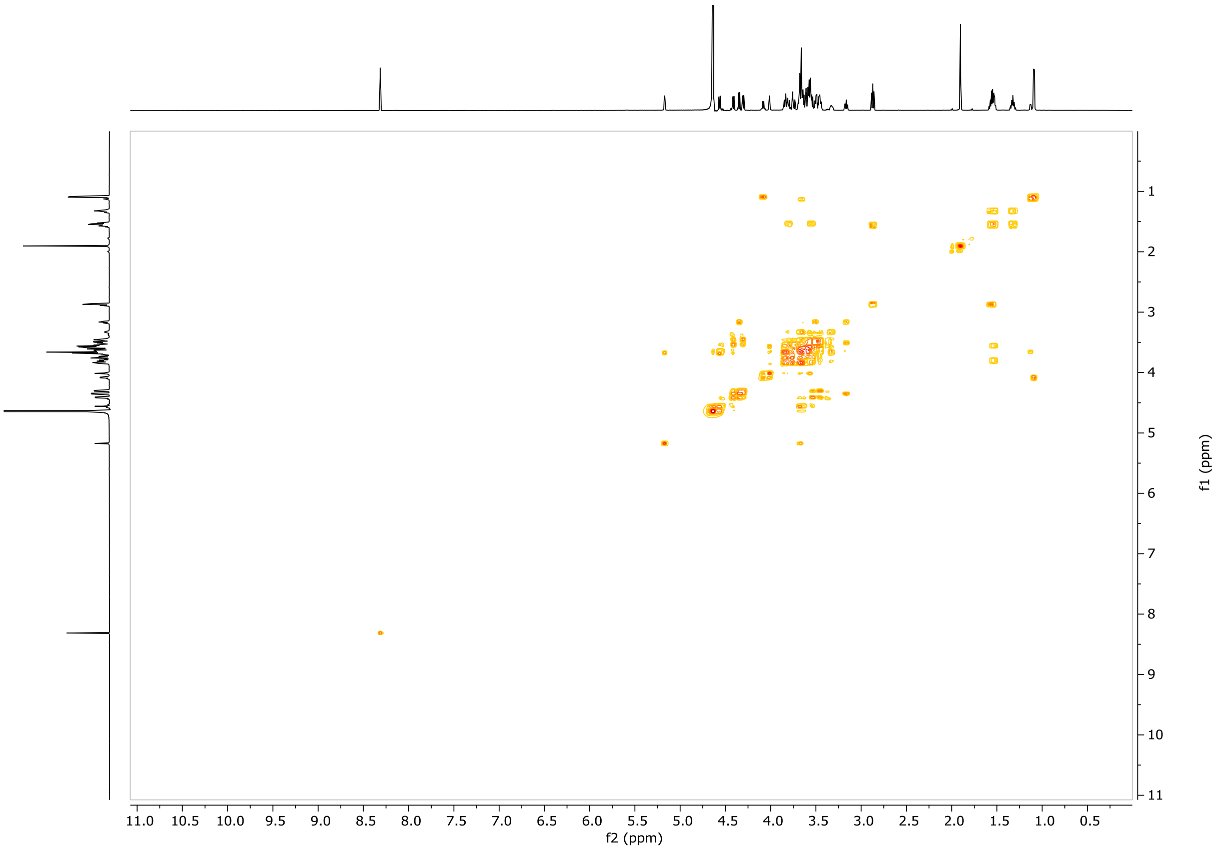

HSQC: **34**

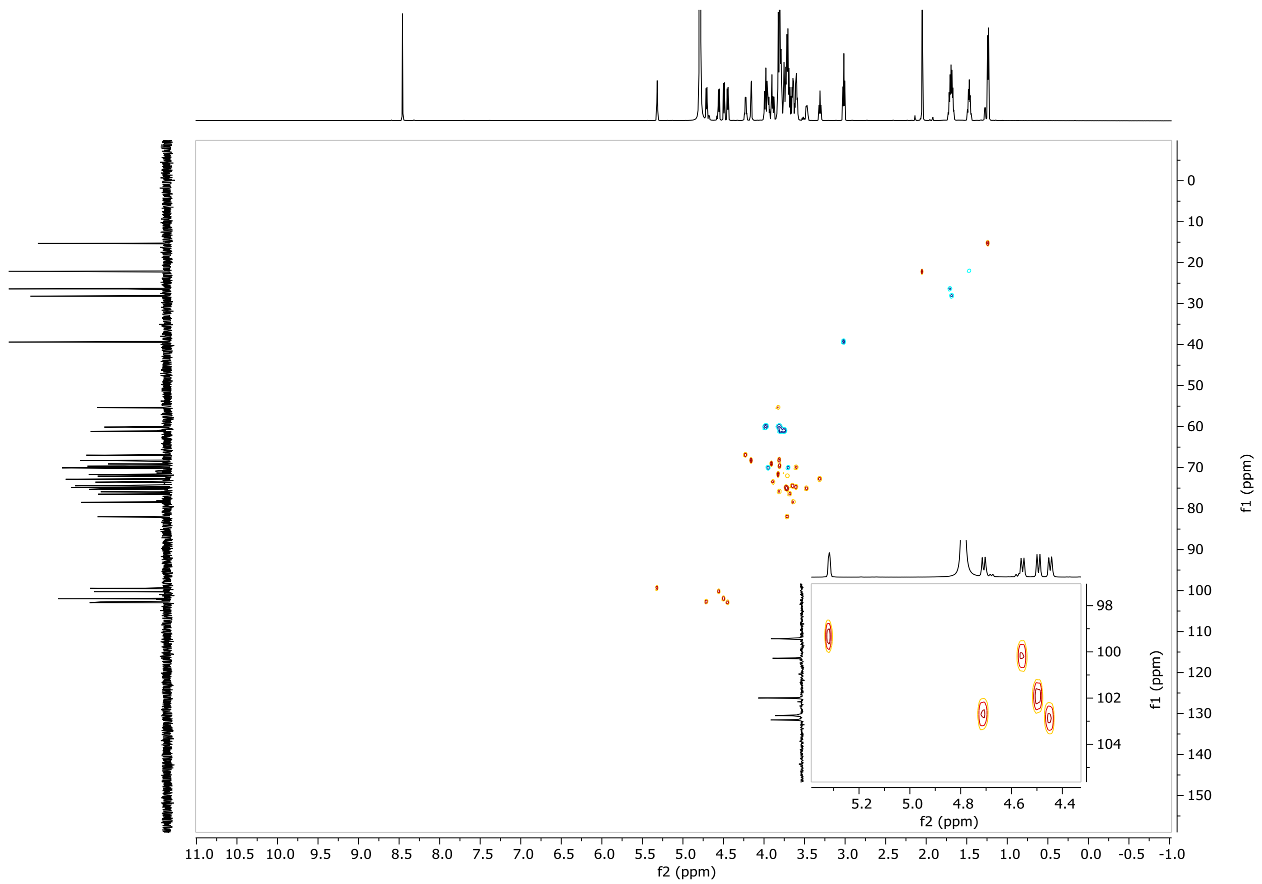

Coupled HSQC: **34**

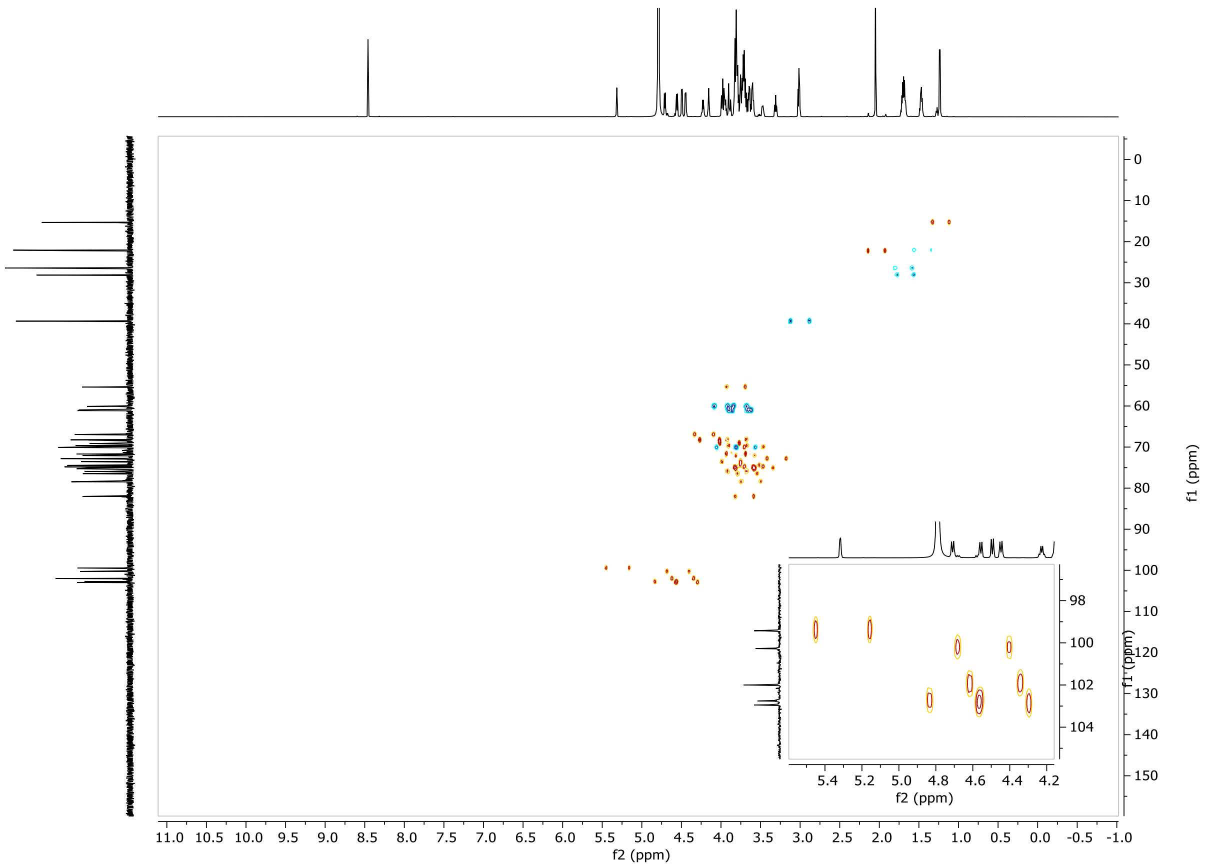

^{1}H NMR: **35**

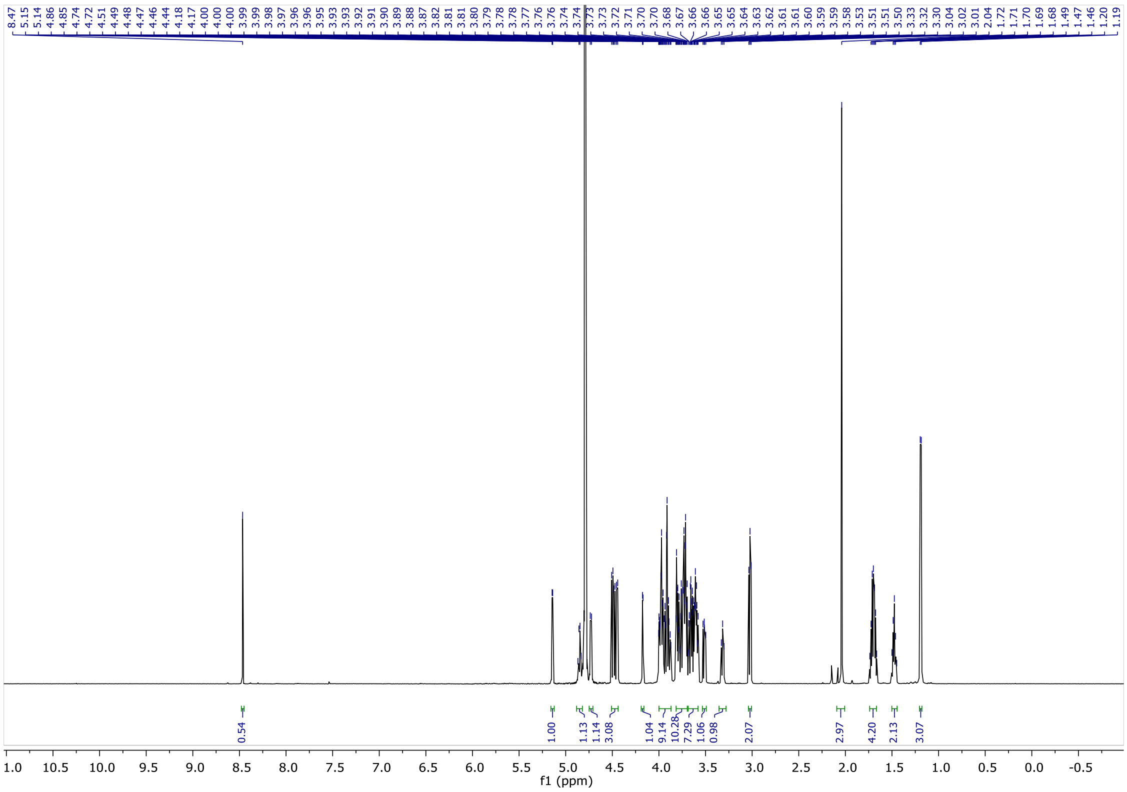

^{13}C NMR: **35**

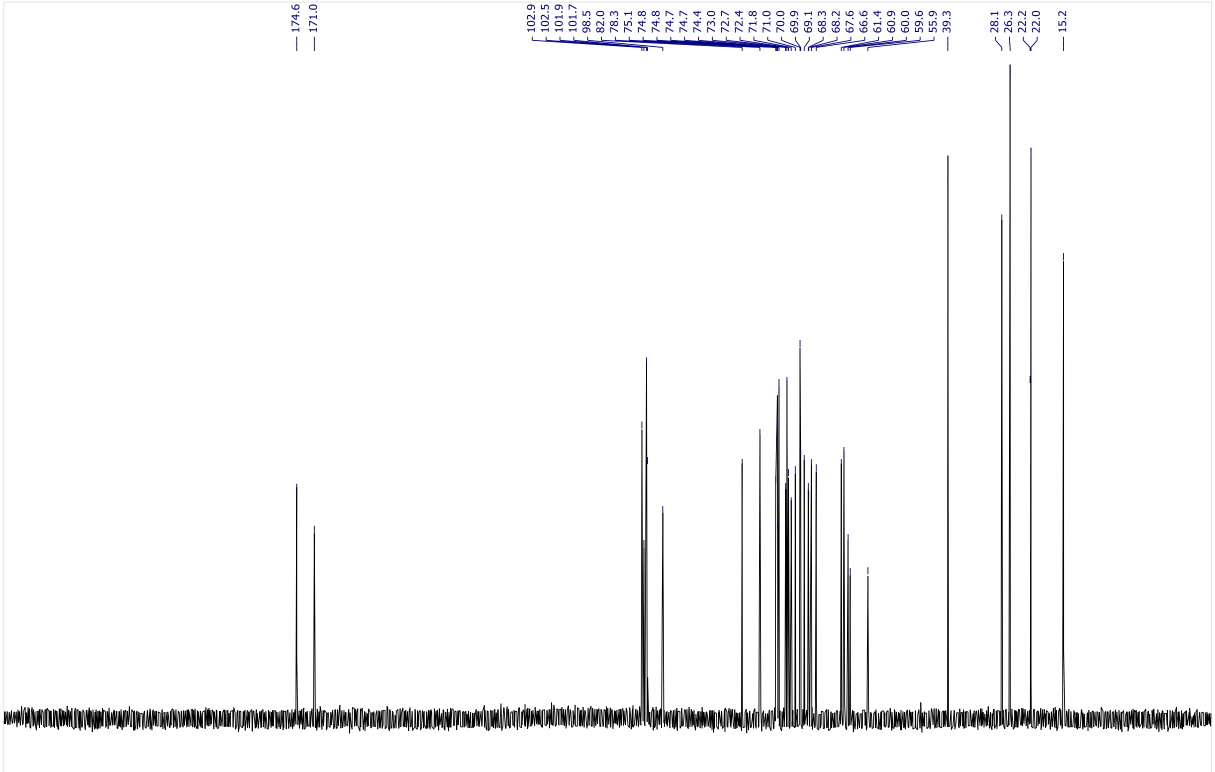

COSY: **35**

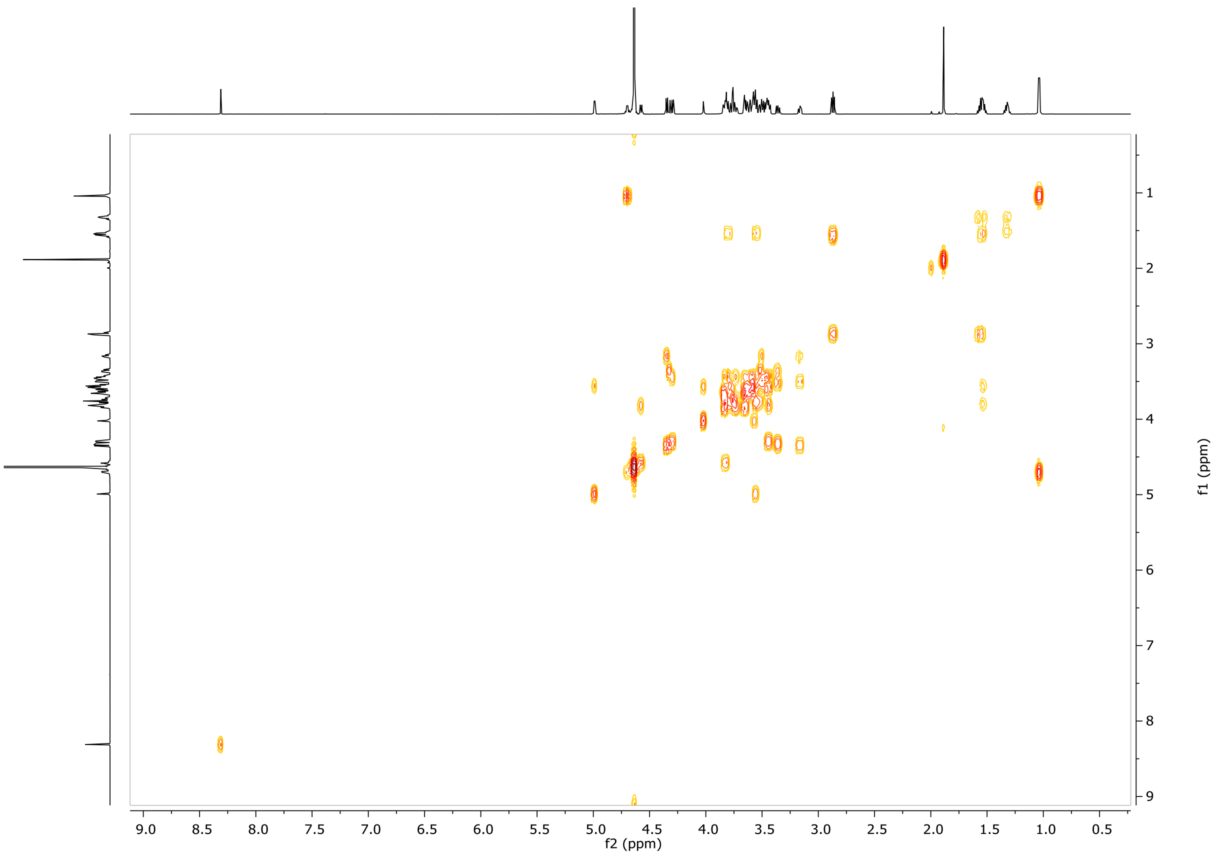

HSQC: **35**

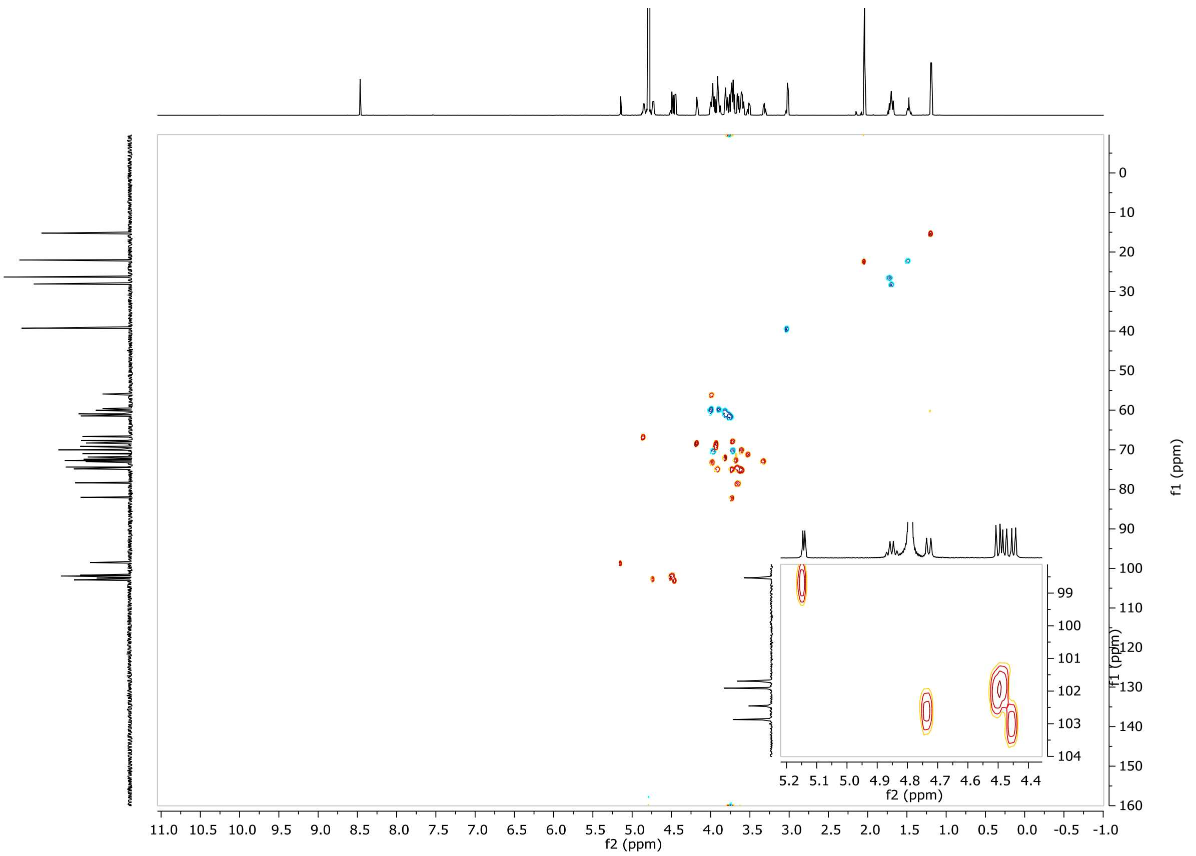

Coupled HSQC: **35**

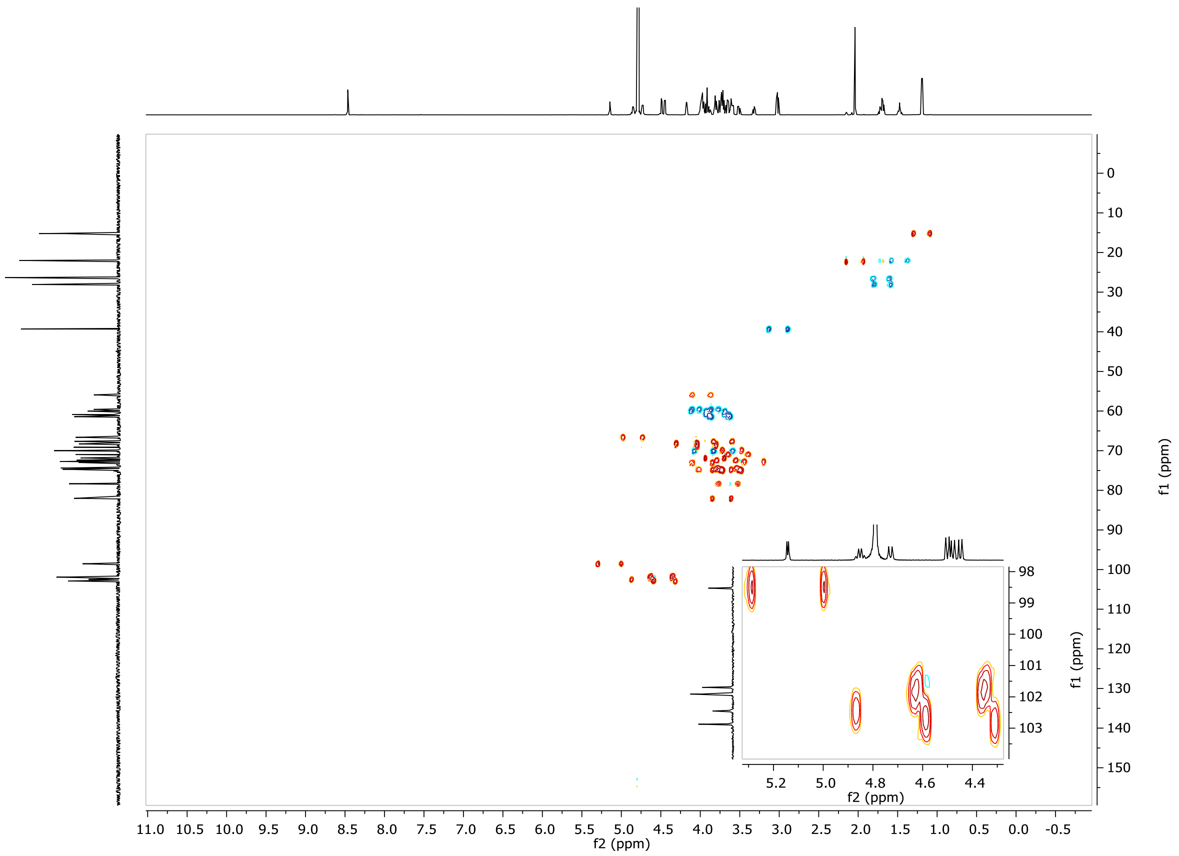

^{1}H NMR: **36**

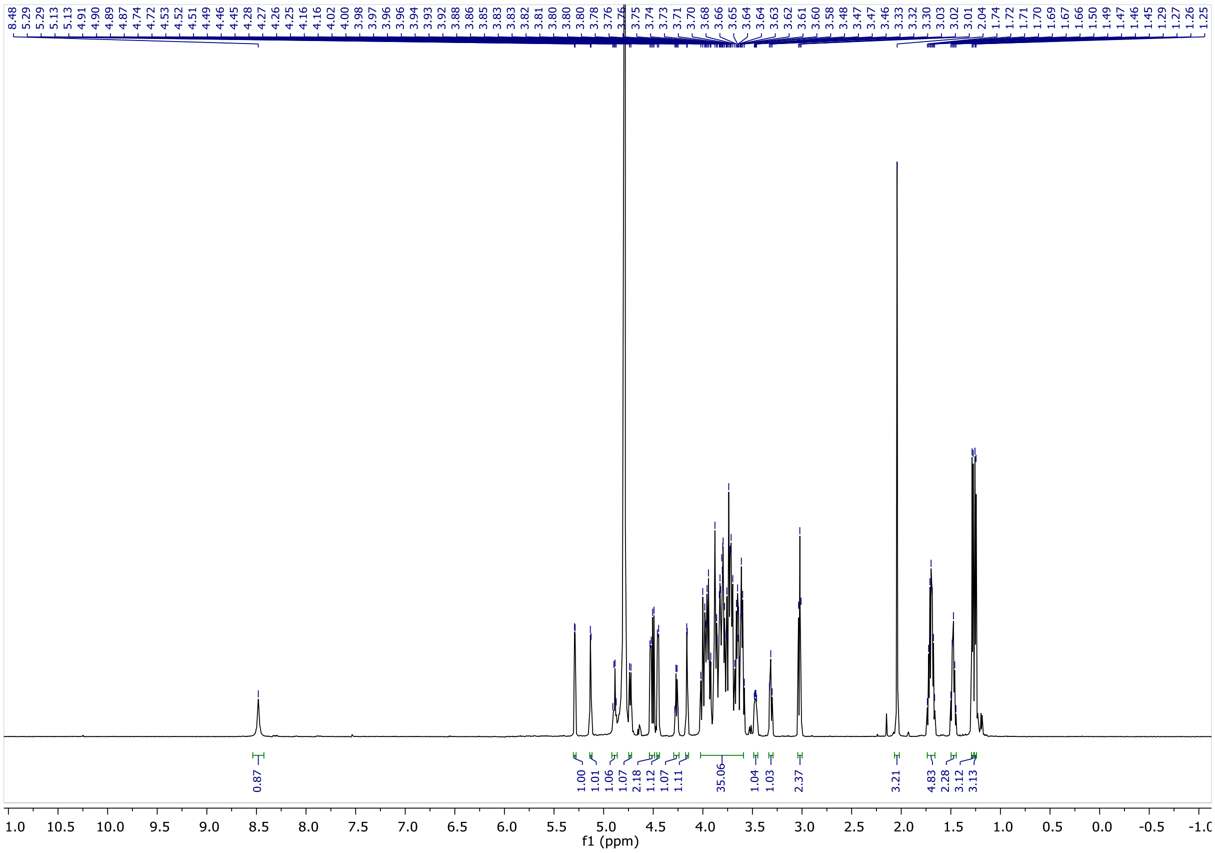

^{13}C NMR: **36**

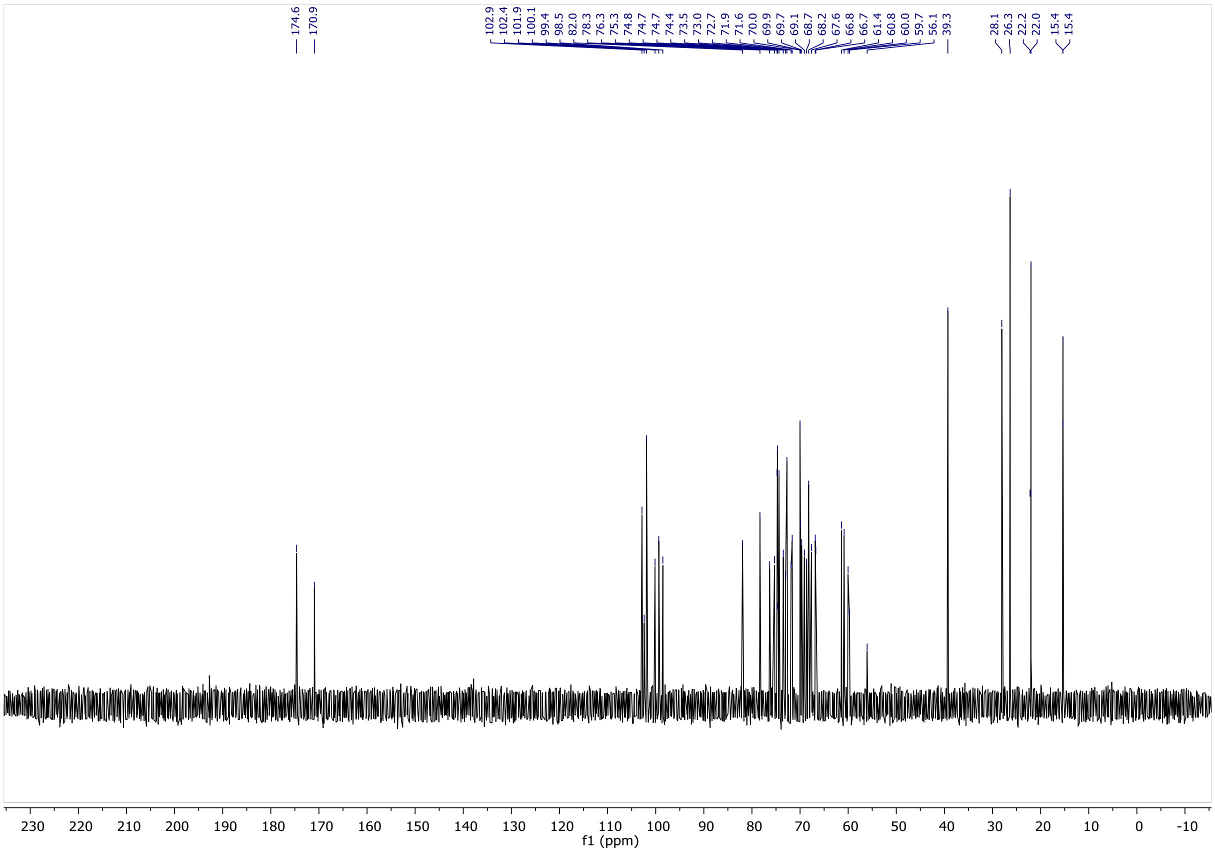

COSY: **36**

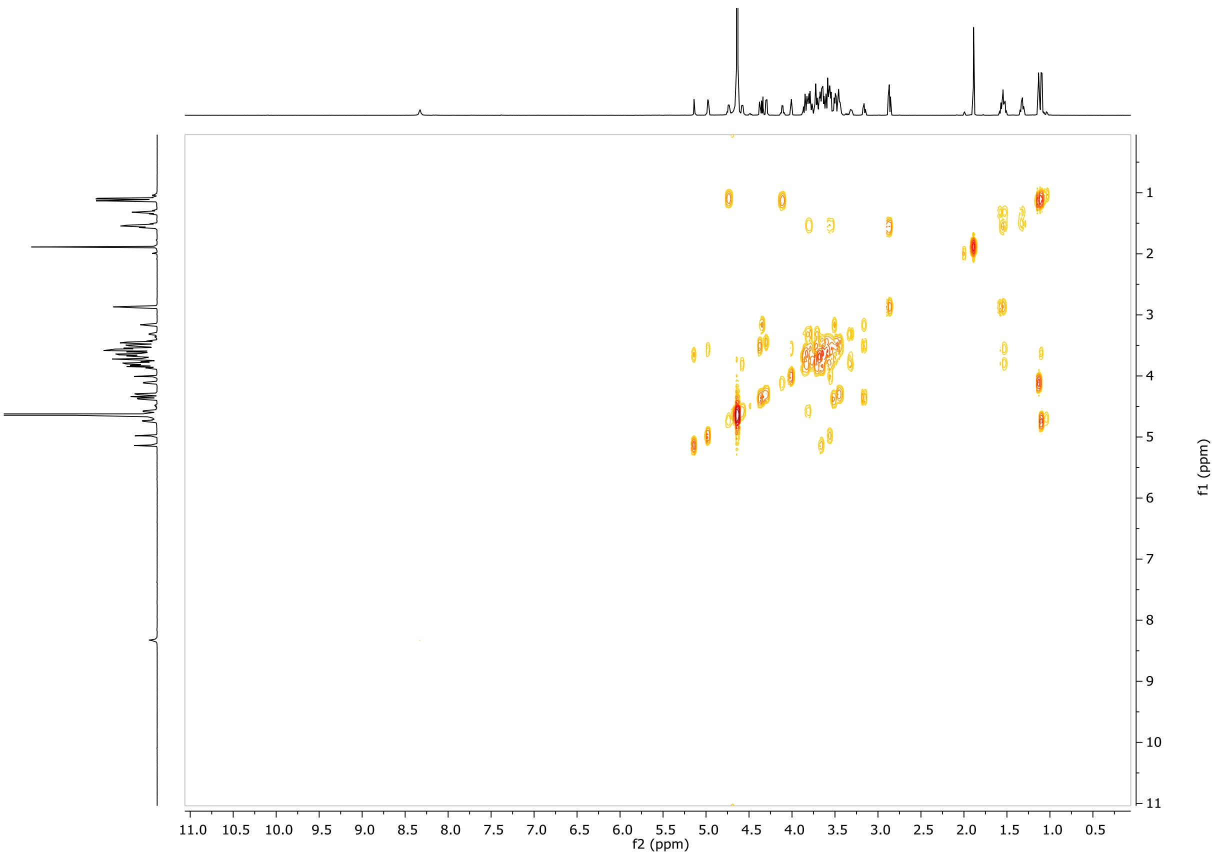

HSQC: **36**

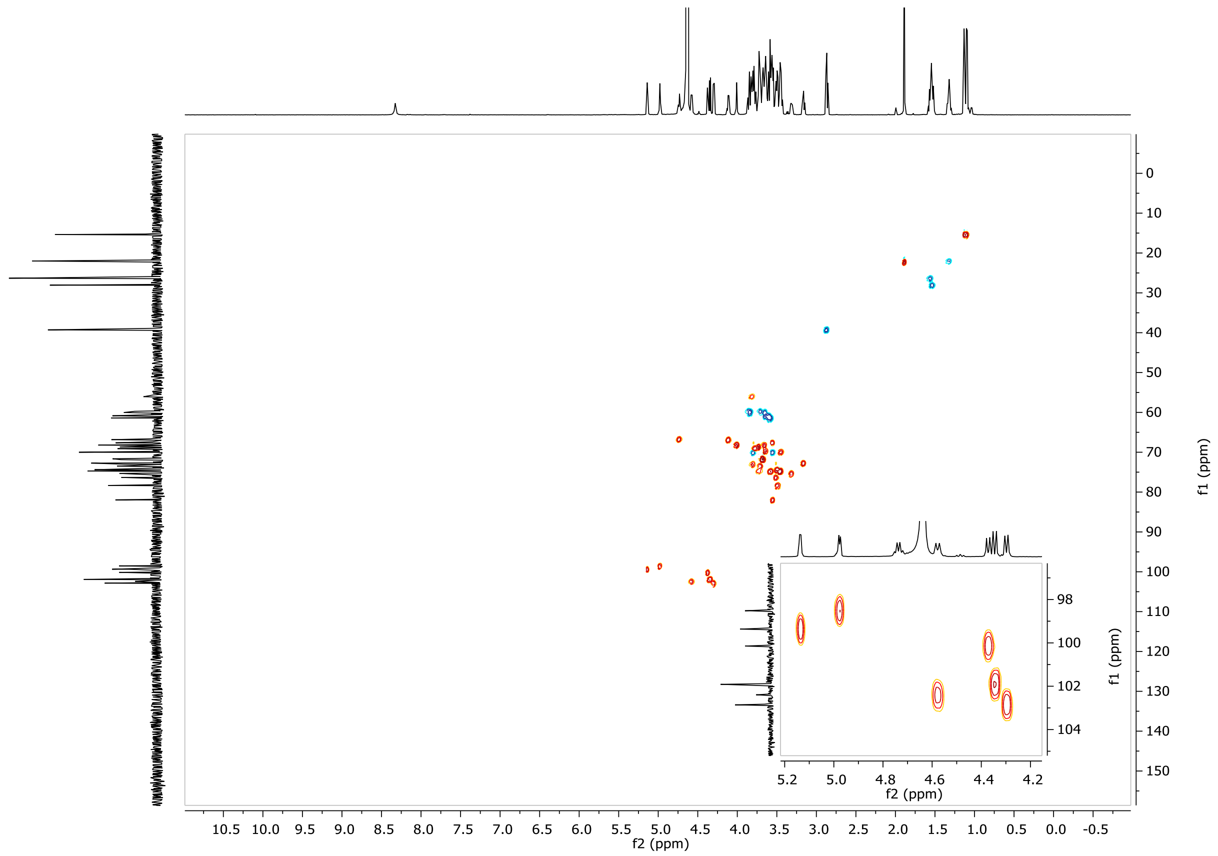

Coupled HSQC: **36**

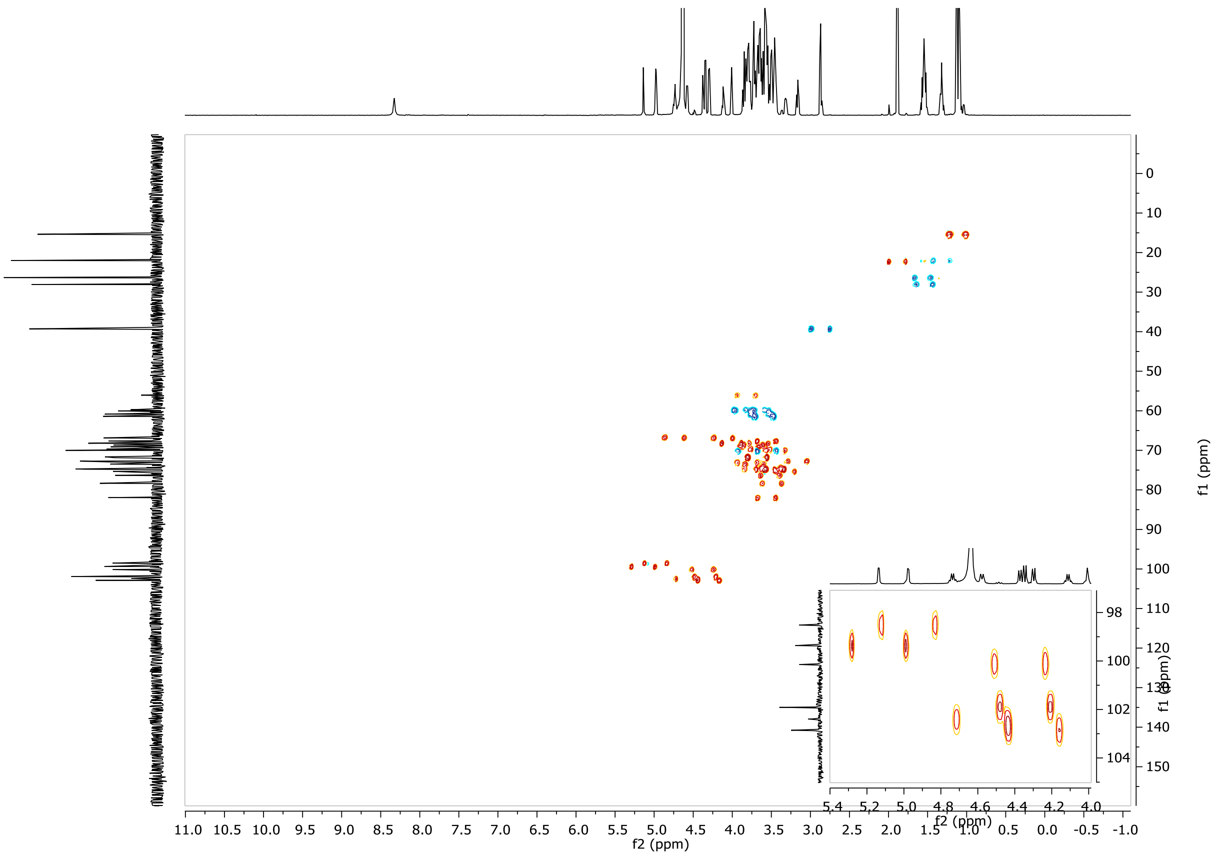

^{1}H NMR: **37**

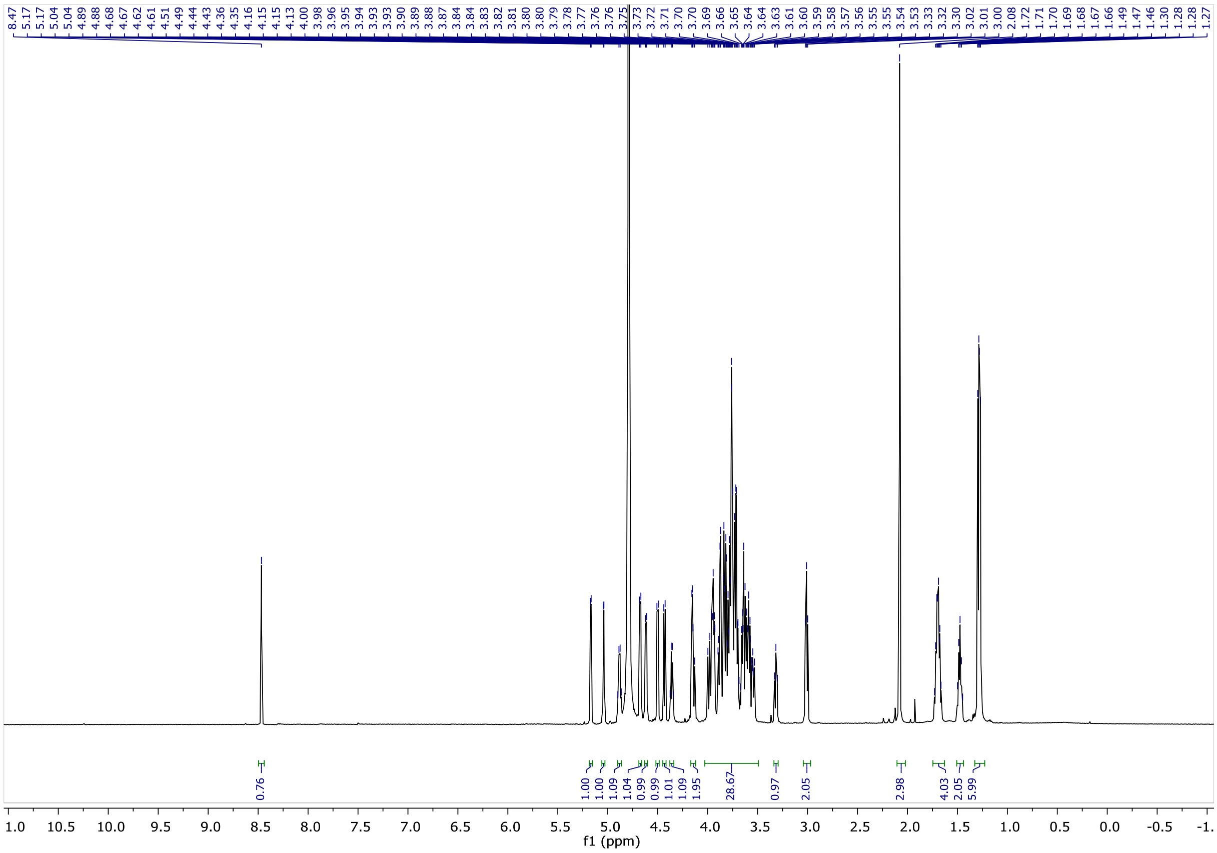

^{13}C NMR: **37**

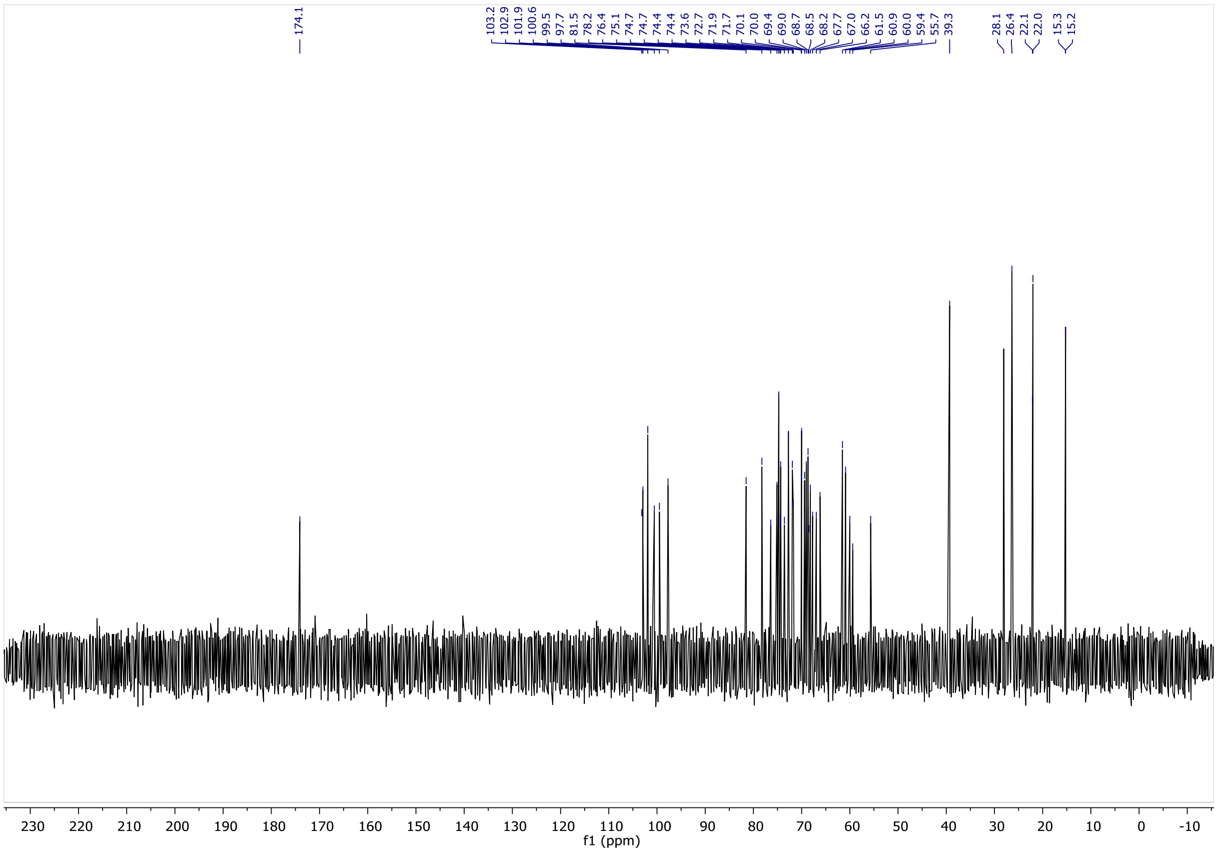

COSY: **37**

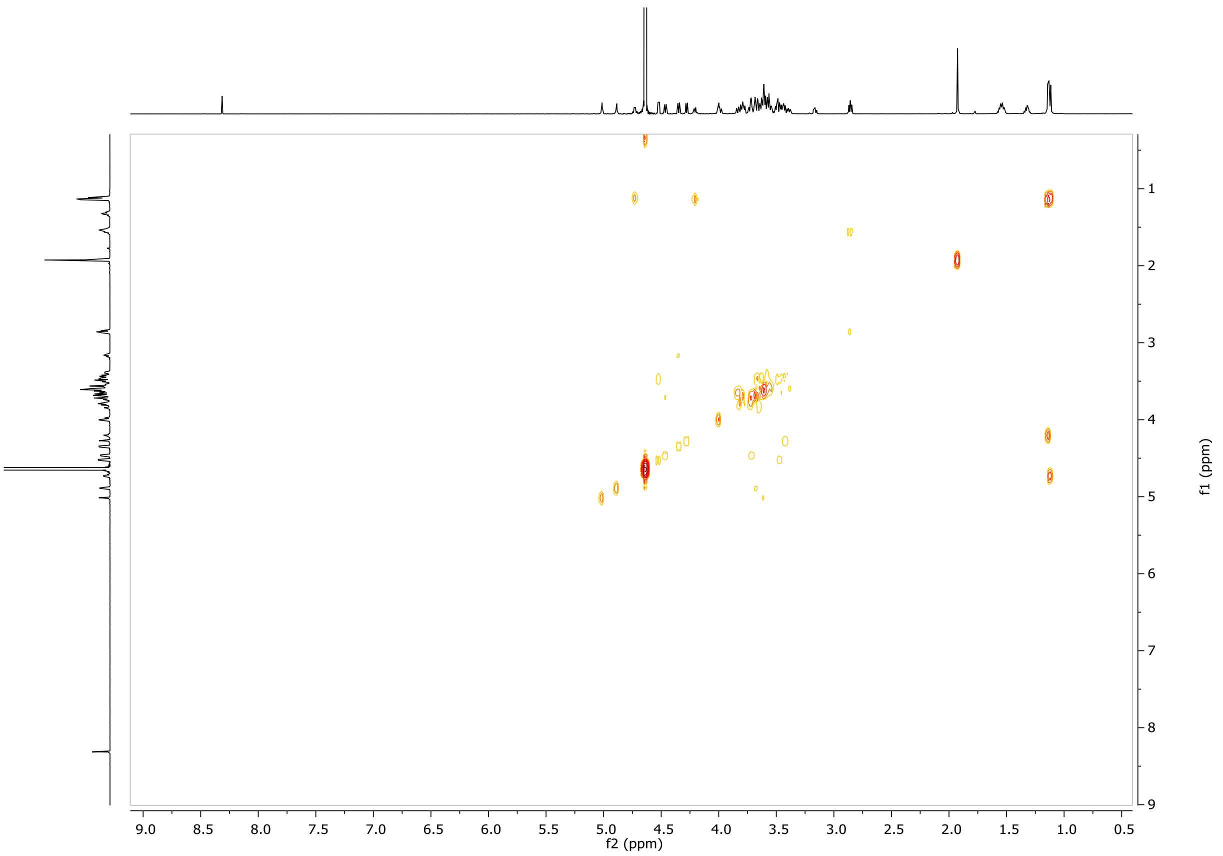

HSQC: **37**

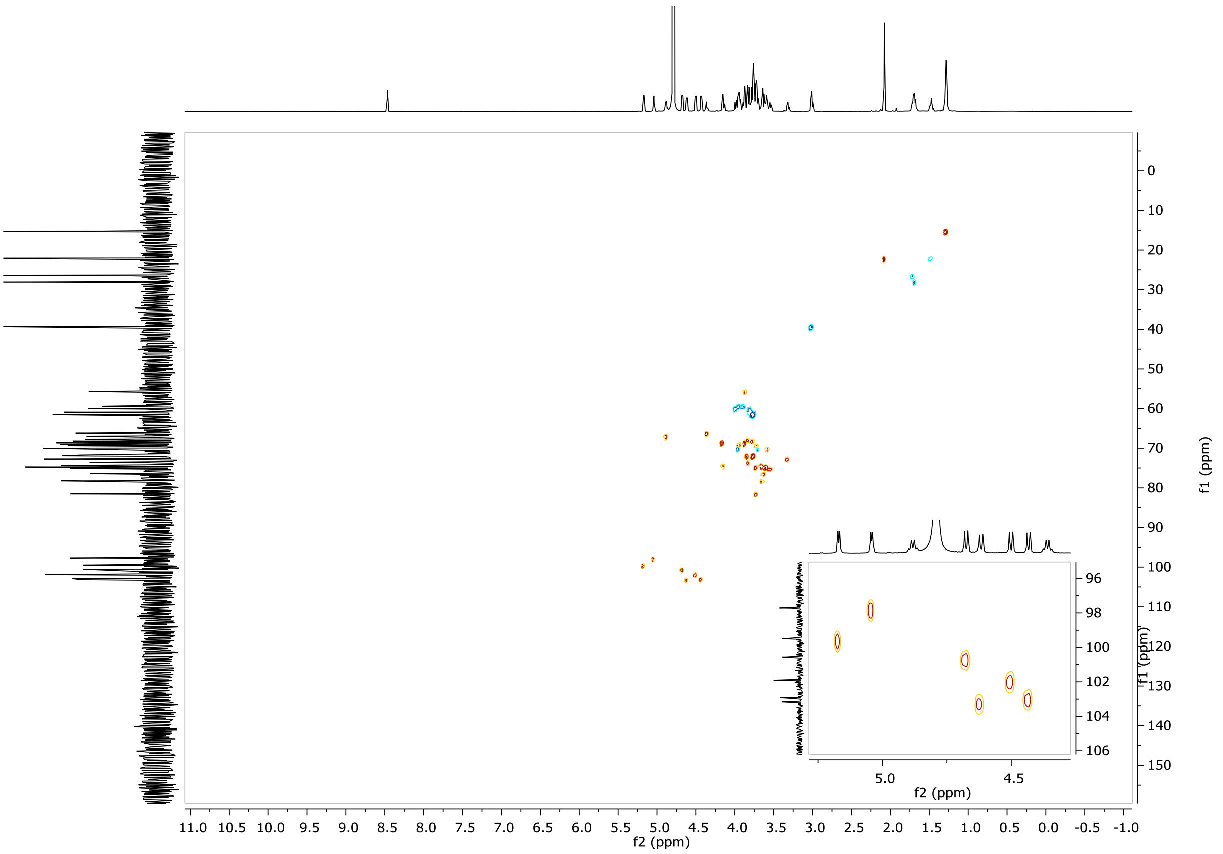

Coupled HSQC: **37**

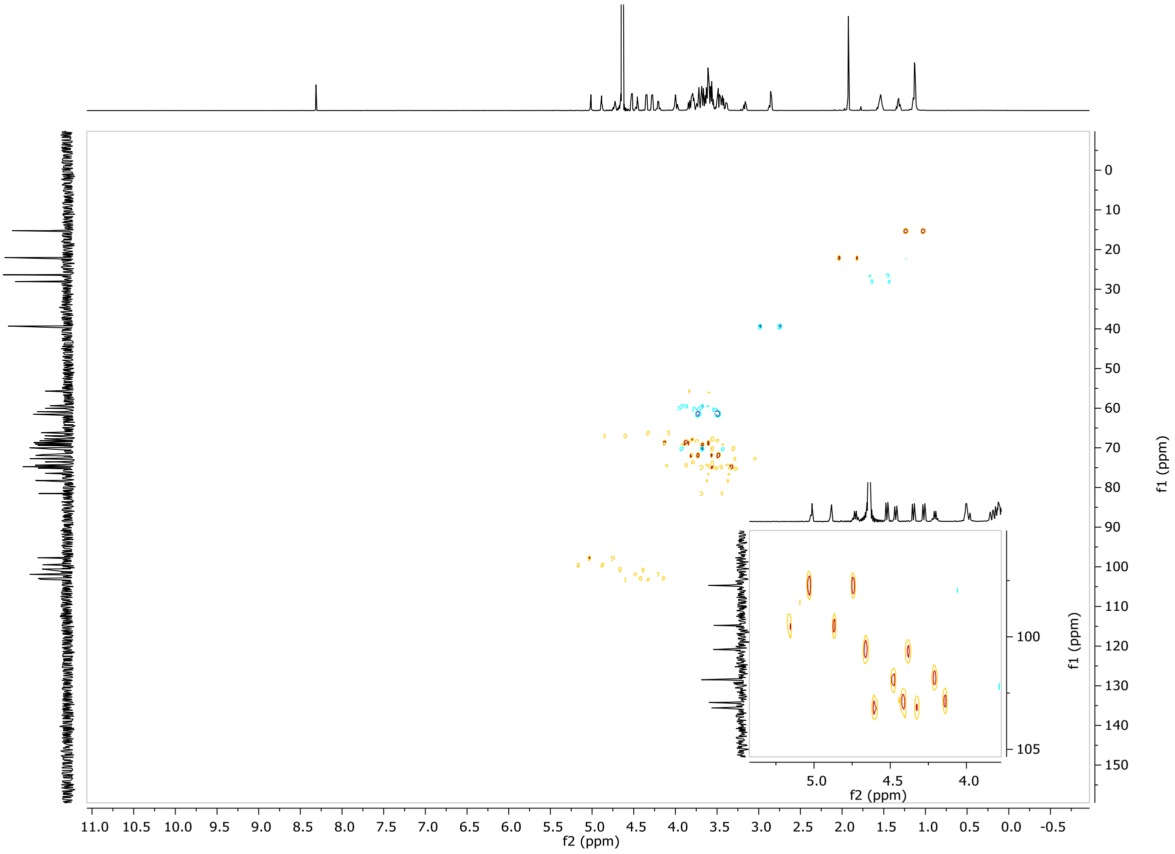

^{1}H NMR: **38**

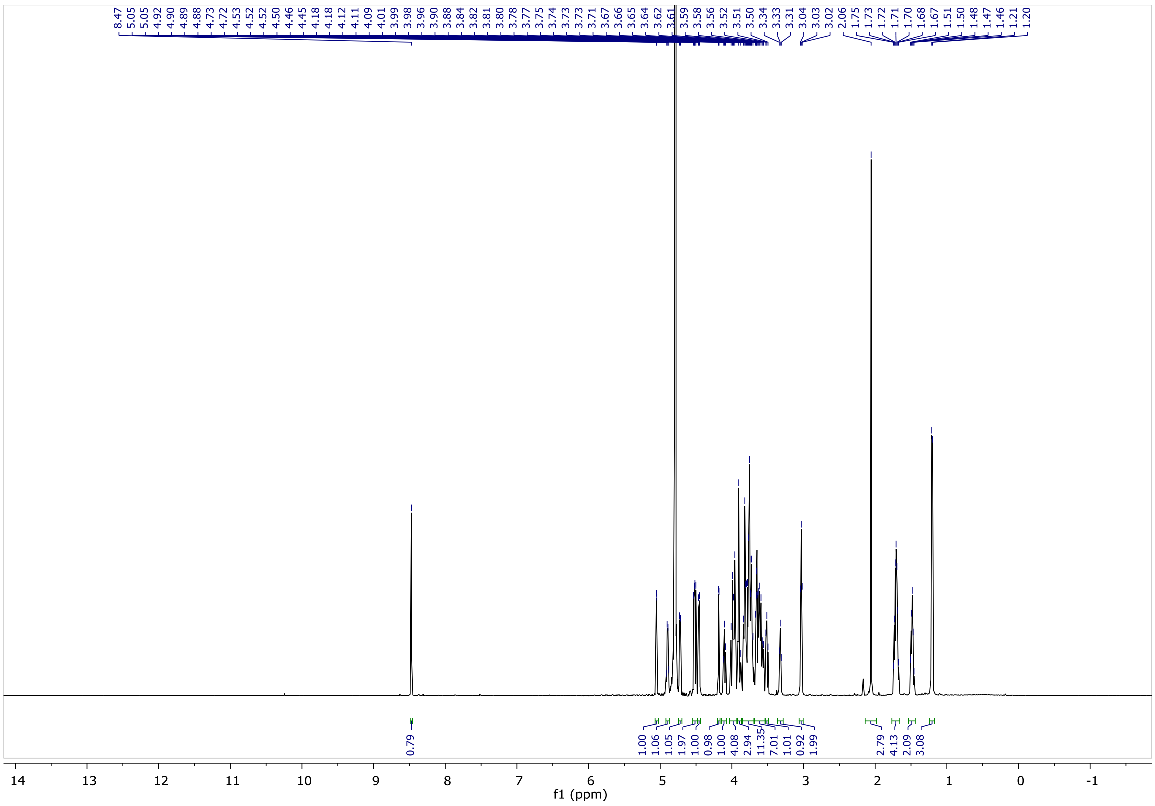

^{13}C NMR: **38**

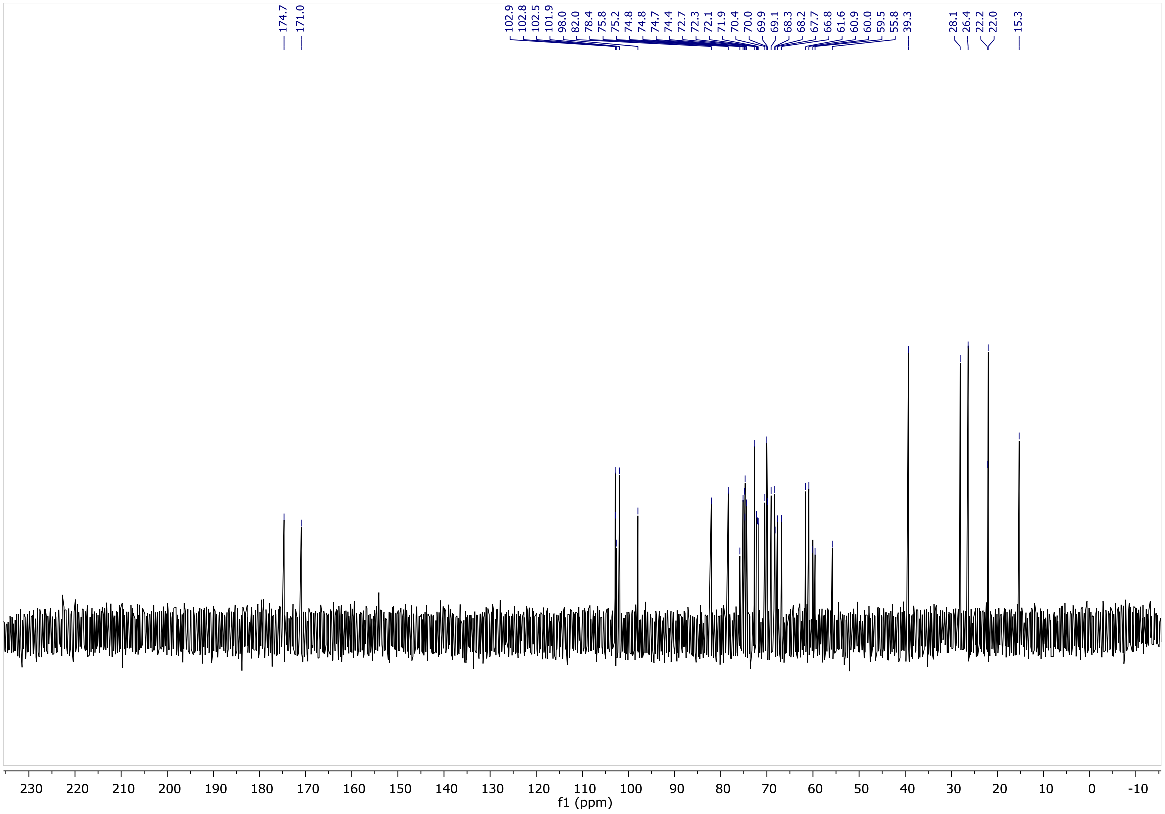

COSY: **38**

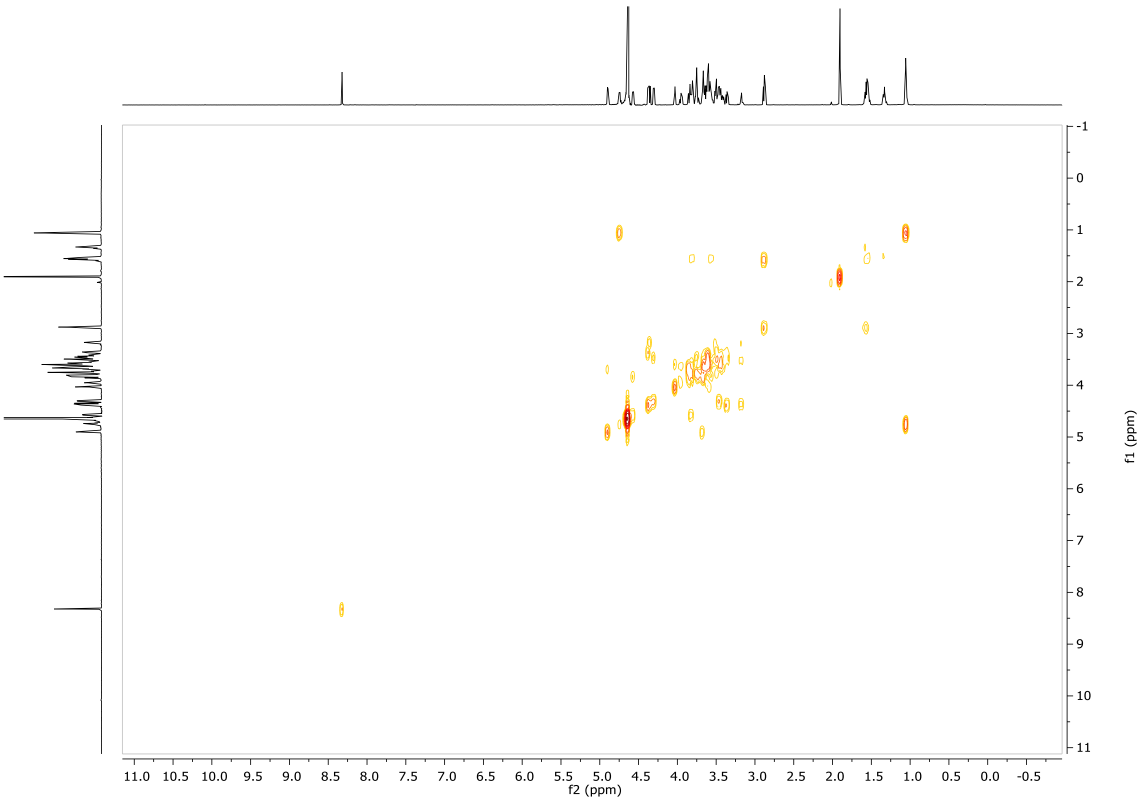

HSQC: **38**

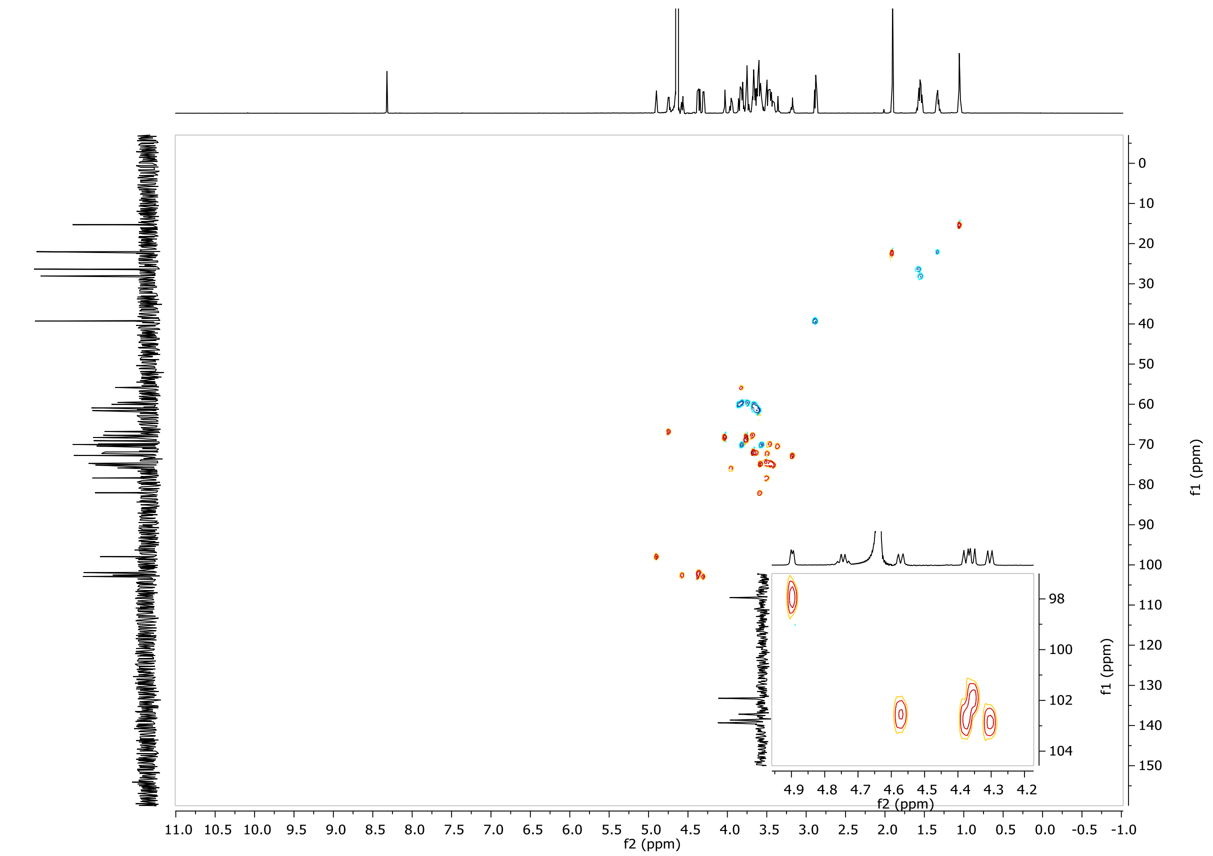

Coupled HSQC: **38**

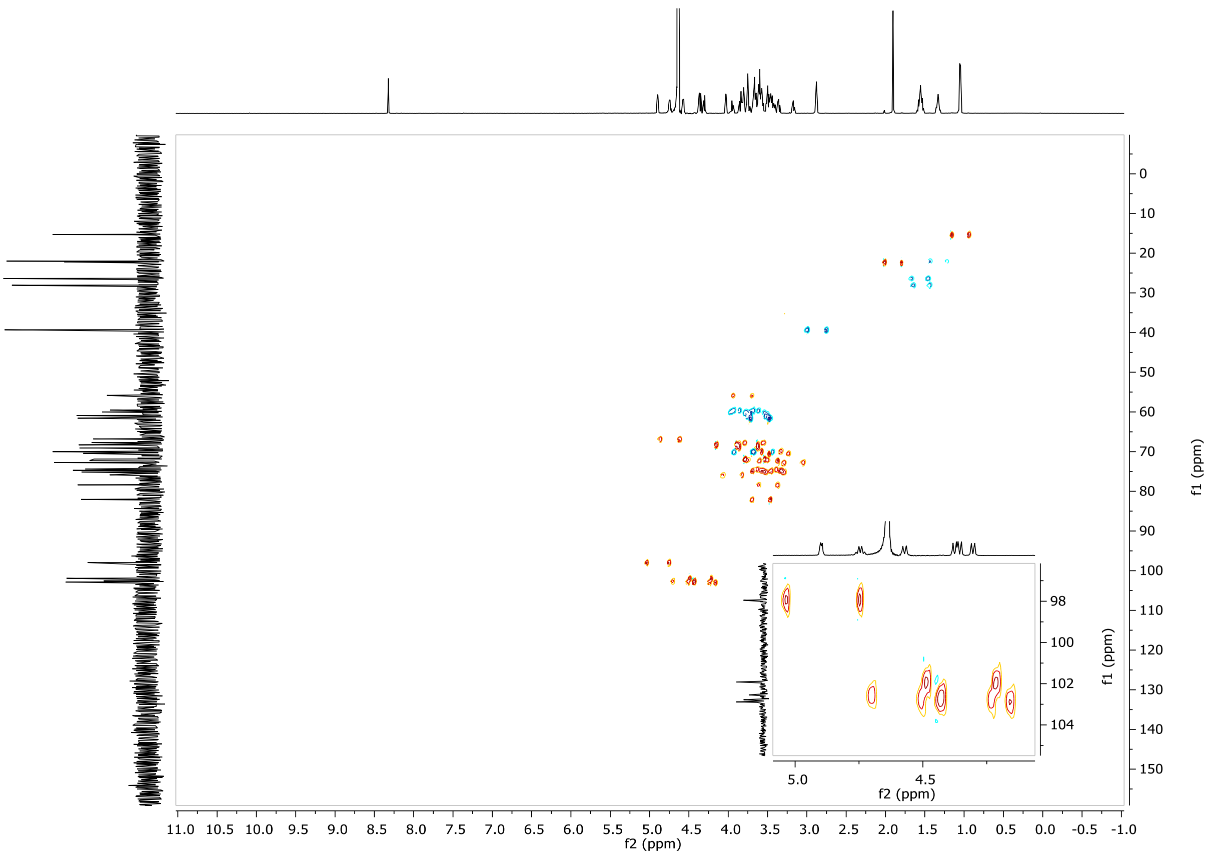

^{1}H NMR: **39**

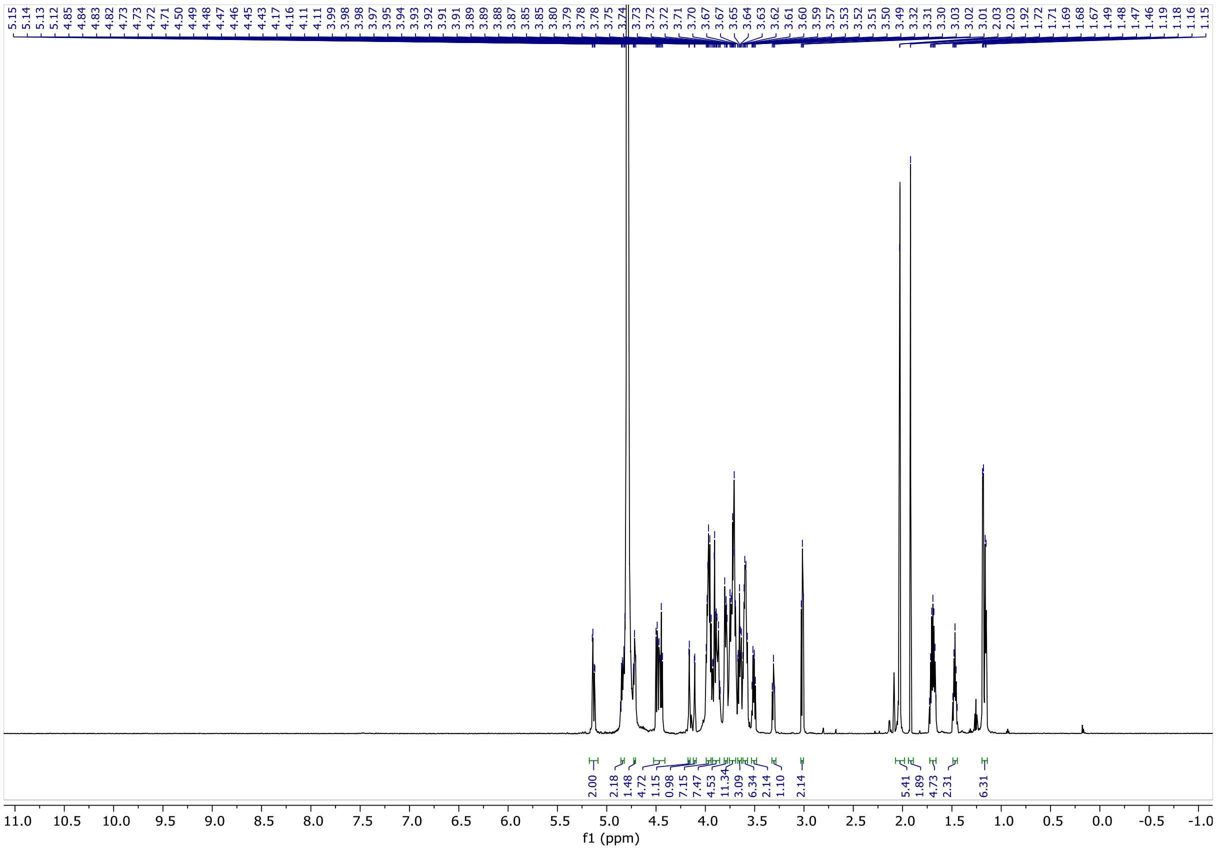

^{13}C NMR: **39**

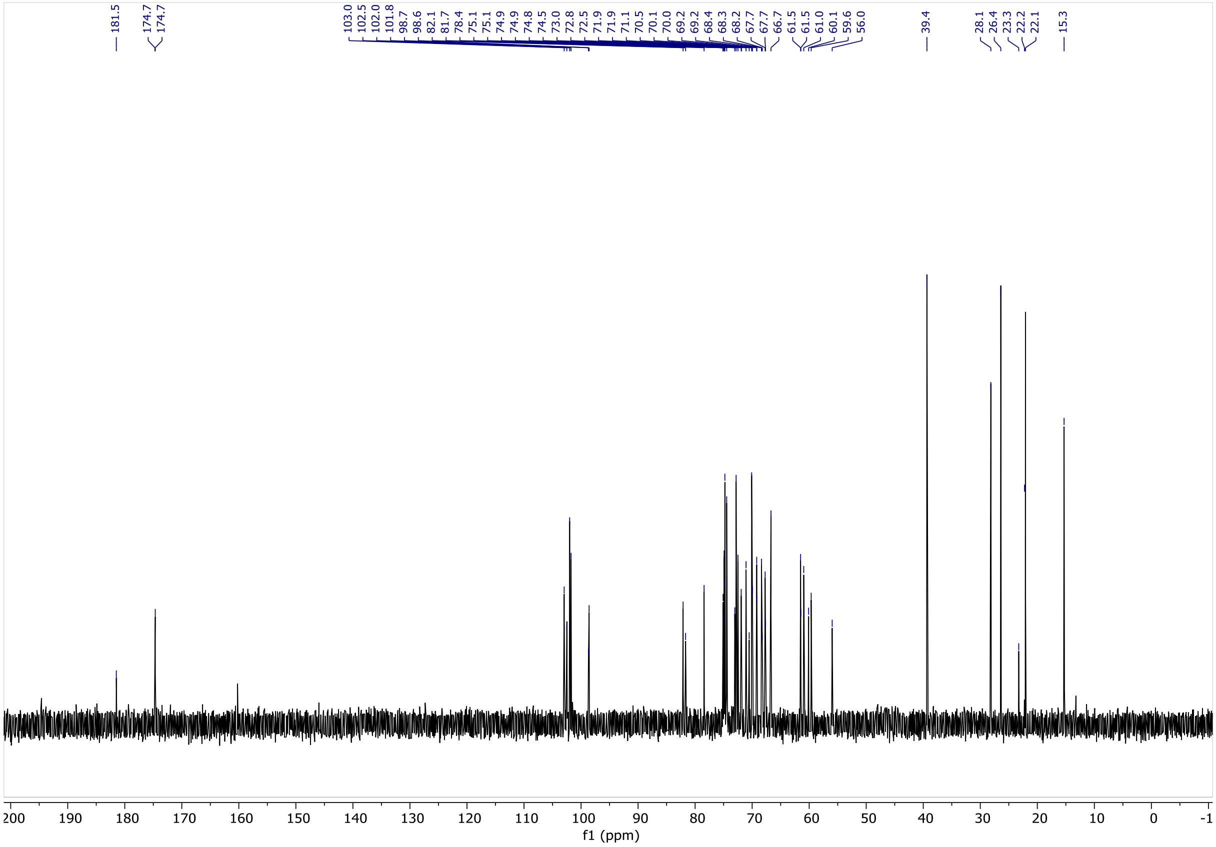

COSY: **39**

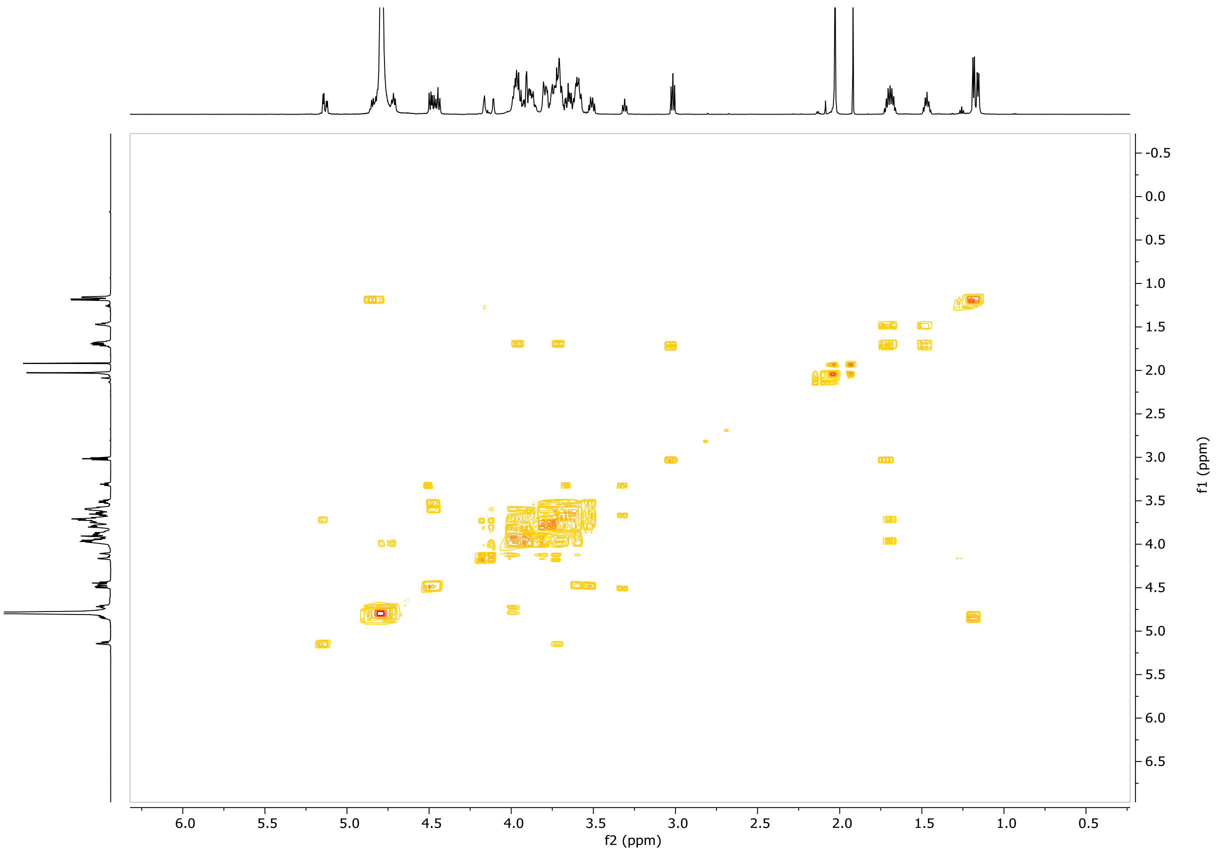

HSQC: **39**

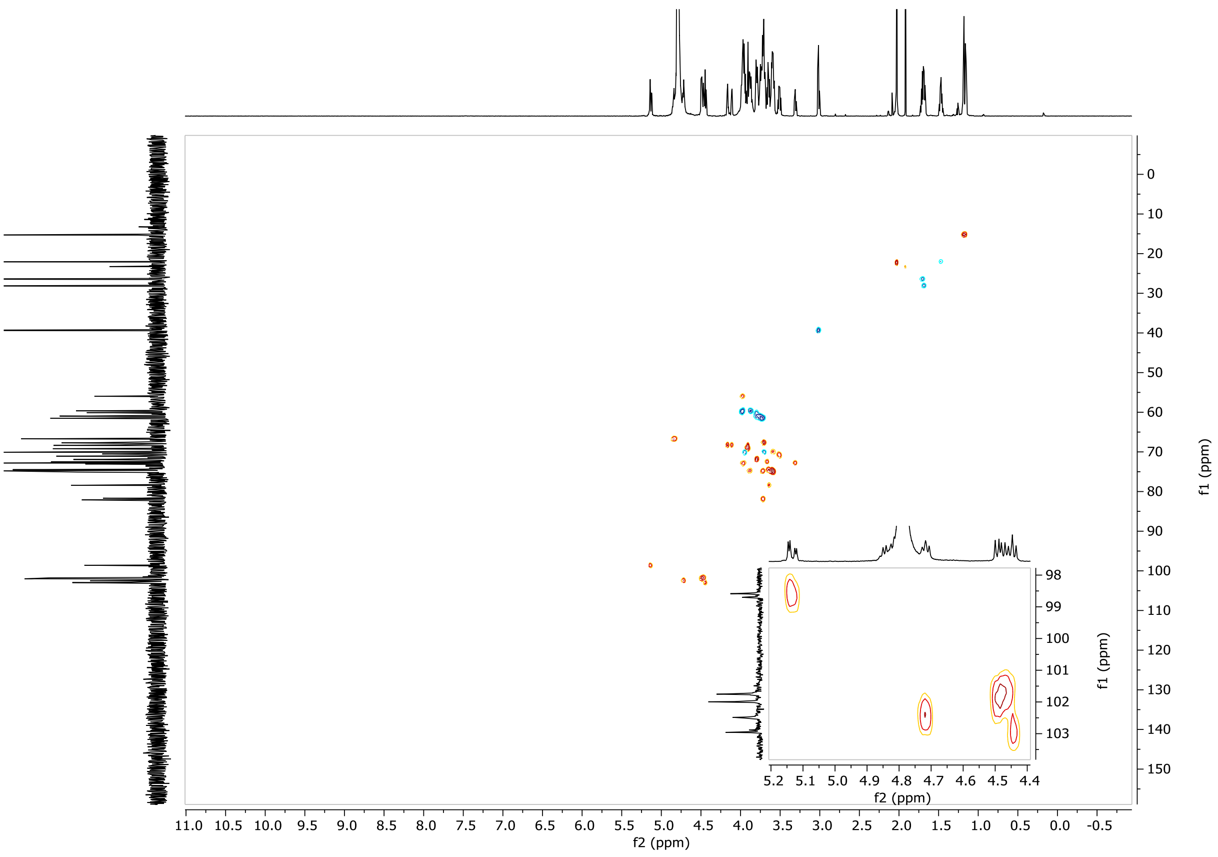

Coupled HSQC: **39**

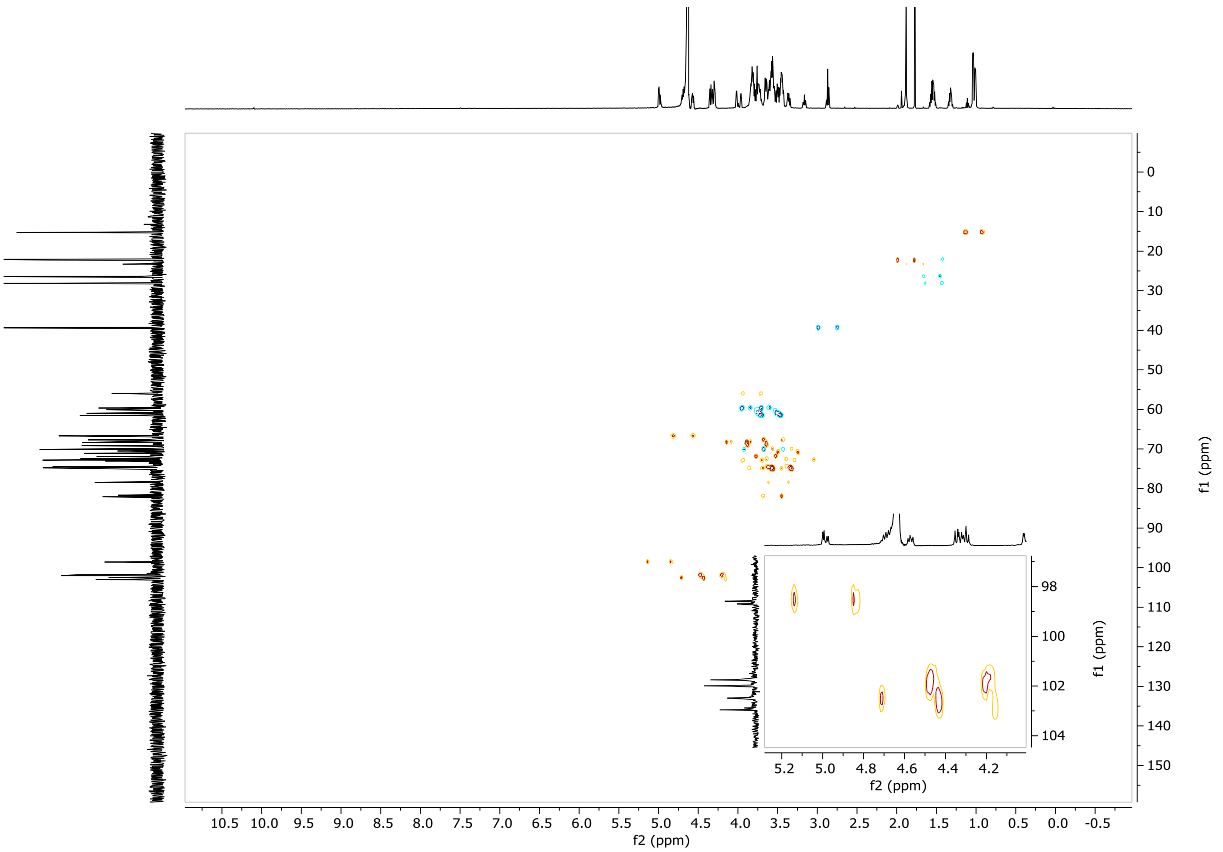

^{1}H NMR: **40**

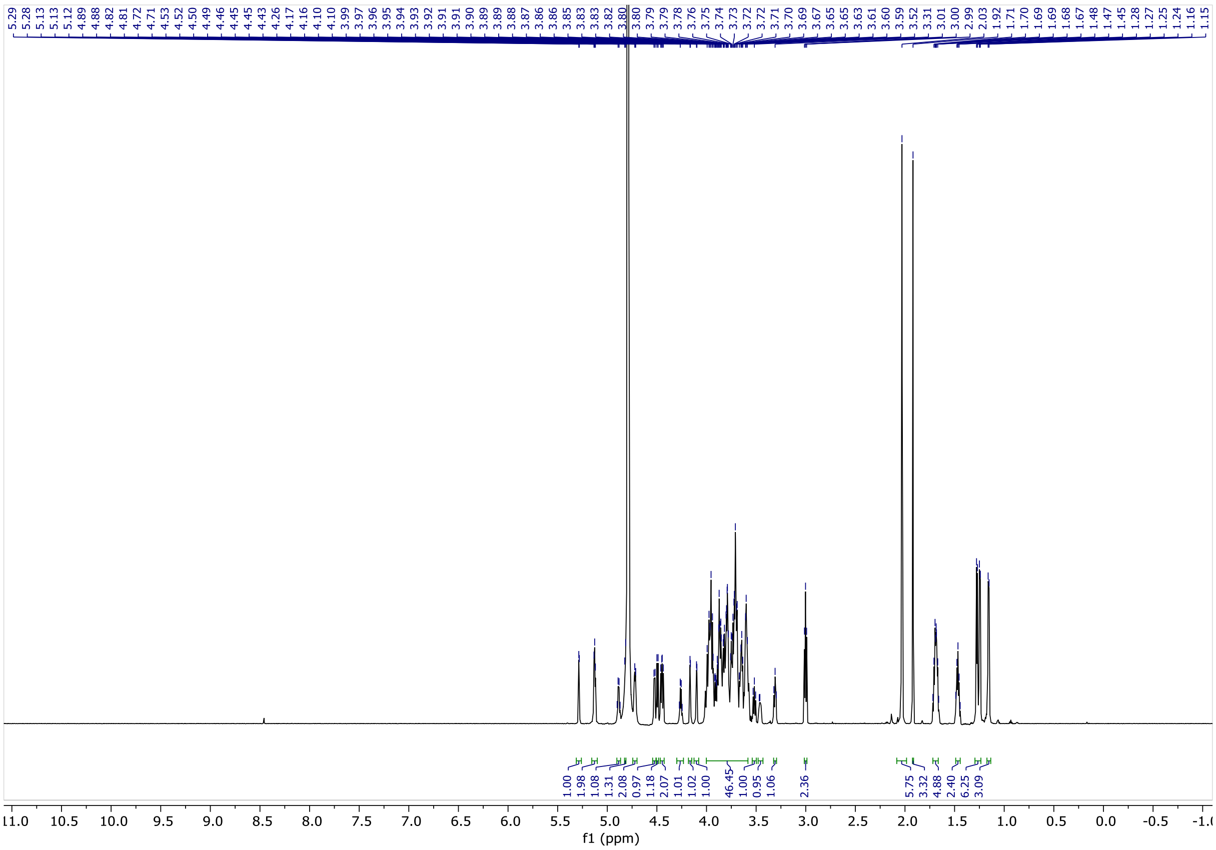

^{13}C NMR: **40**

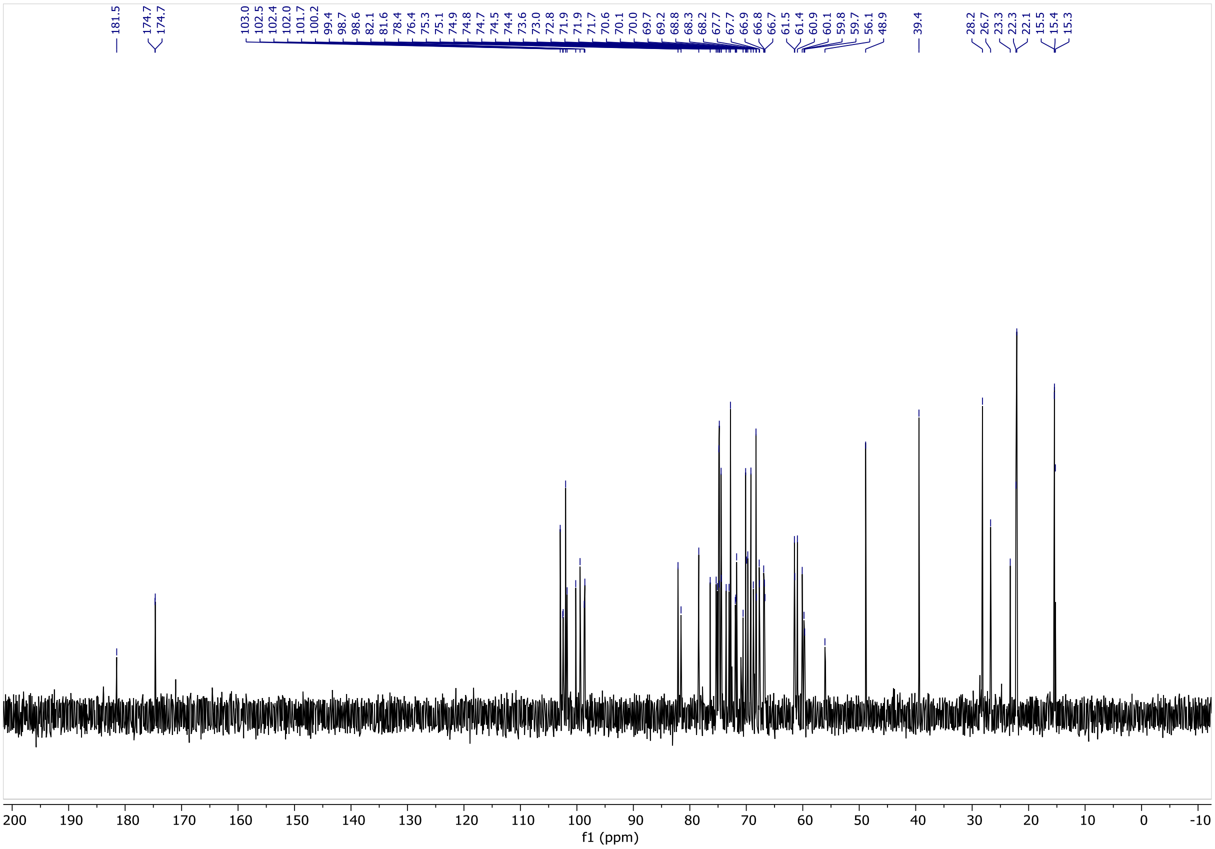

181.5
174.7
174.7
103.0
102.5
102.4
102.0
101.7
100.2
99.4
98.7
98.6
82.1
81.6
78.4
76.4
75.3
75.1
74.9
74.8
74.7
74.5
74.4
73.6
73.0
72.8
71.9
71.9
71.7
70.6
70.1
70.0
69.7
69.2
68.8
68.3
68.2
67.7
67.7
66.9
66.8
66.7
61.5
61.4
60.9
60.1
59.8
59.7
56.1
48.9
39.4
28.2
26.7
23.3
22.3
22.1
15.5
15.4
15.3
f1 (ppm)

COSY: **40**

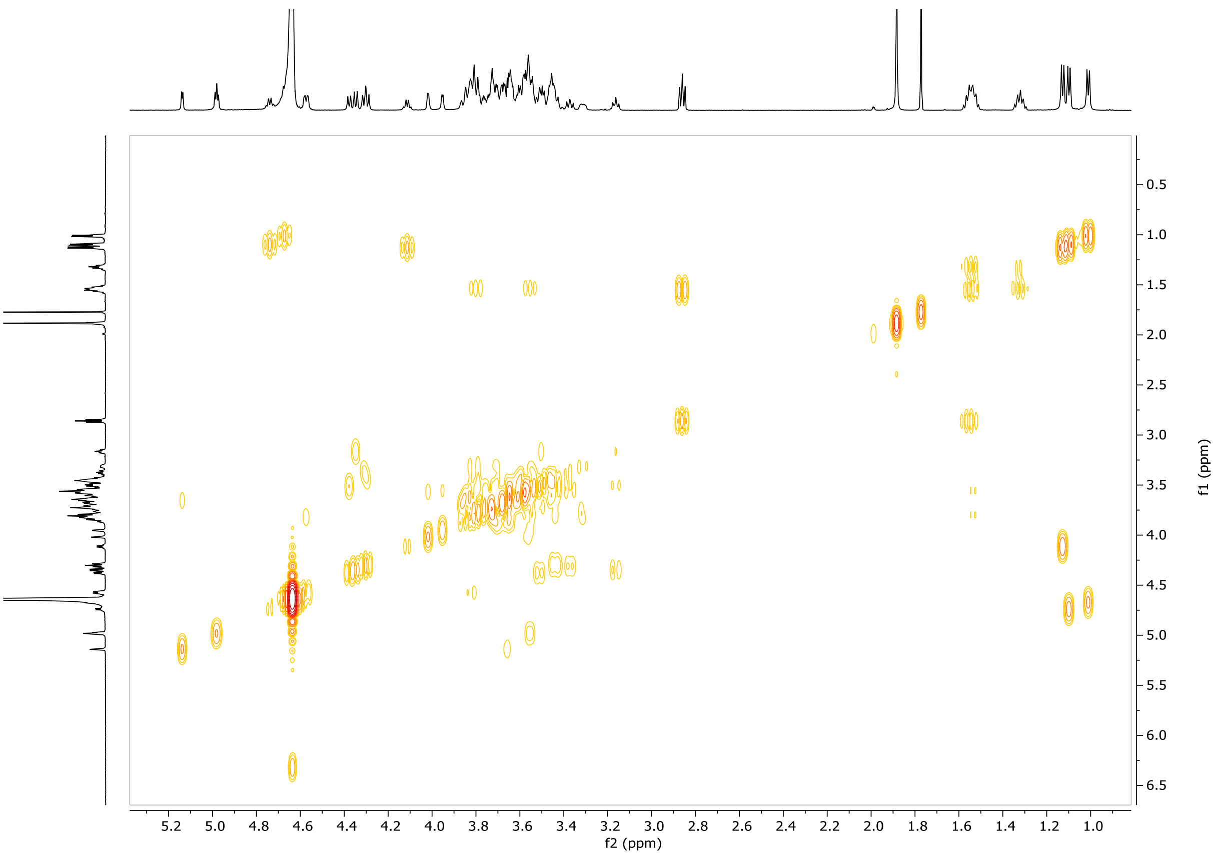

f2 (ppm)
f1 (ppm)

HSQC: **40**

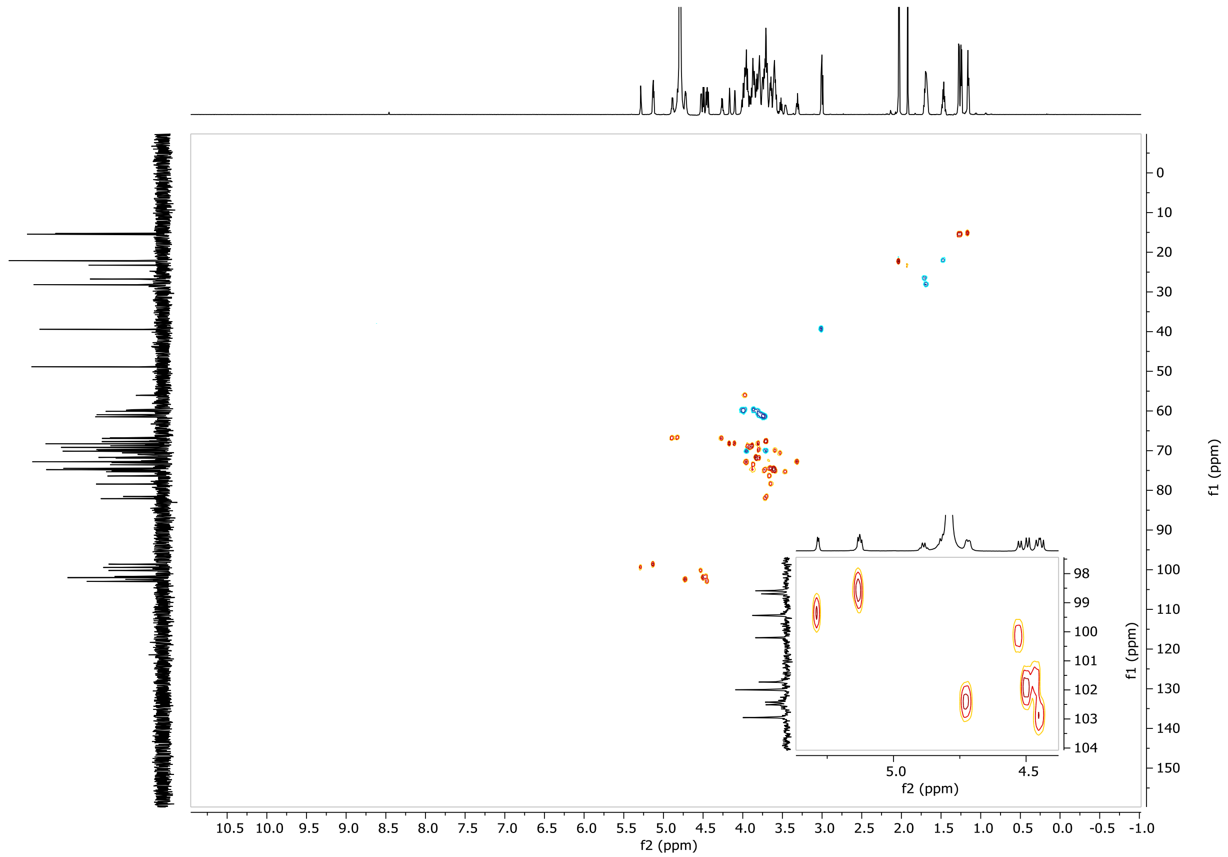

Coupled HSQC: **40**

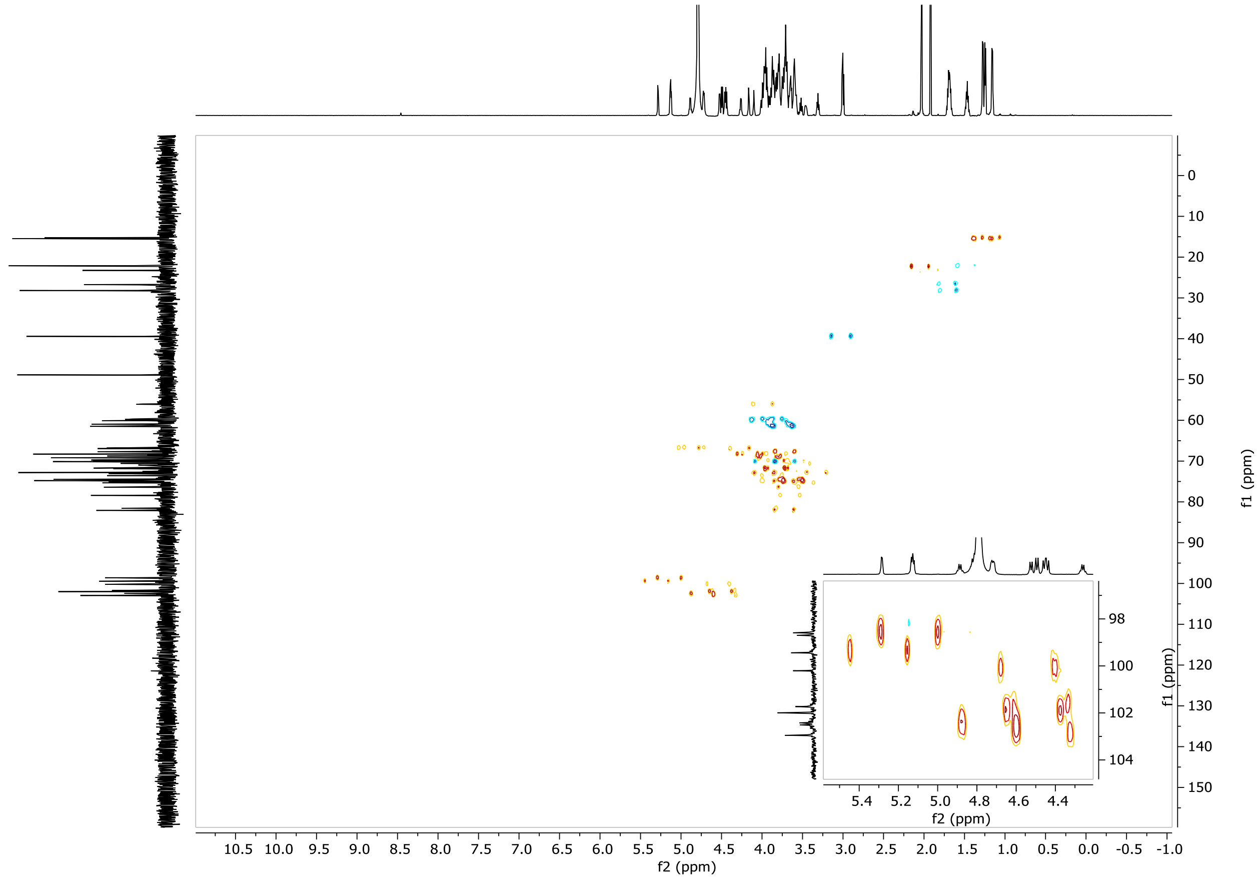

Supplement: Supplementary file 1 [file SC-010-C9SC00768G-s001.pdf]
